# A Hemilabile NHC‐Gold Complex and its Application to the Redox Neutral 1,2‐Oxyarylation of Feedstock Alkenes

**DOI:** 10.1002/anie.202301526

**Published:** 2023-05-02

**Authors:** Samuel C. Scott, Jamie A. Cadge, Grace K. Boden, John F. Bower, Christopher A. Russell

**Affiliations:** ^1^ School of Chemistry University of Bristol Cantock's Close Bristol BS8 1TS UK; ^2^ Department of Chemistry University of Liverpool Crown Street Liverpool L69 7ZD UK

**Keywords:** Ethylene, Gold, NHC, Oxidative Addition, Oxyarylation

## Abstract

We describe a Au^I^ complex of a hemi‐labile (C^N) N‐heterocyclic carbene ligand that is able to mediate oxidative addition of aryl iodides. Detailed computational and experimental investigations have been undertaken to verify and rationalize the oxidative addition process. Application of this initiation mode has resulted in the first examples of “exogenous oxidant‐free” Au^I^/Au^III^ catalyzed 1,2‐oxyarylations of ethylene and propylene. These demanding yet powerful processes establish these commodity chemicals as *nucleophilic‐electrophilic* building blocks in catalytic reaction design.

## Graphical abstract

A hemi‐labile (C^N) N‐heterocyclic carbene ligand mediates oxidative addition of aryl iodides to a Au^I^ center. The oxidative addition process has been studied and analyzed computationally. This process allows the first examples of “exogenous oxidant‐free” Au^I^/Au^III^ catalyzed 1,2‐oxyarylations of ethylene and propylene.

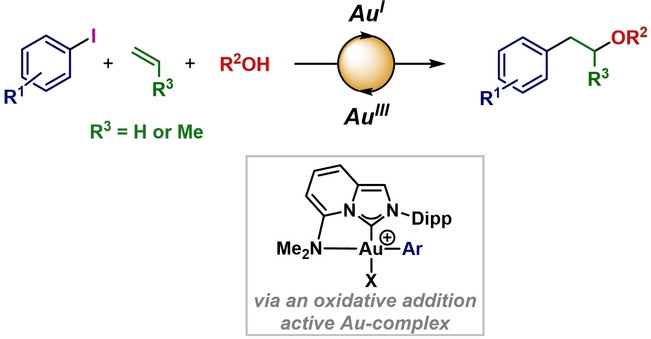

## Introduction

**Scheme 1:**
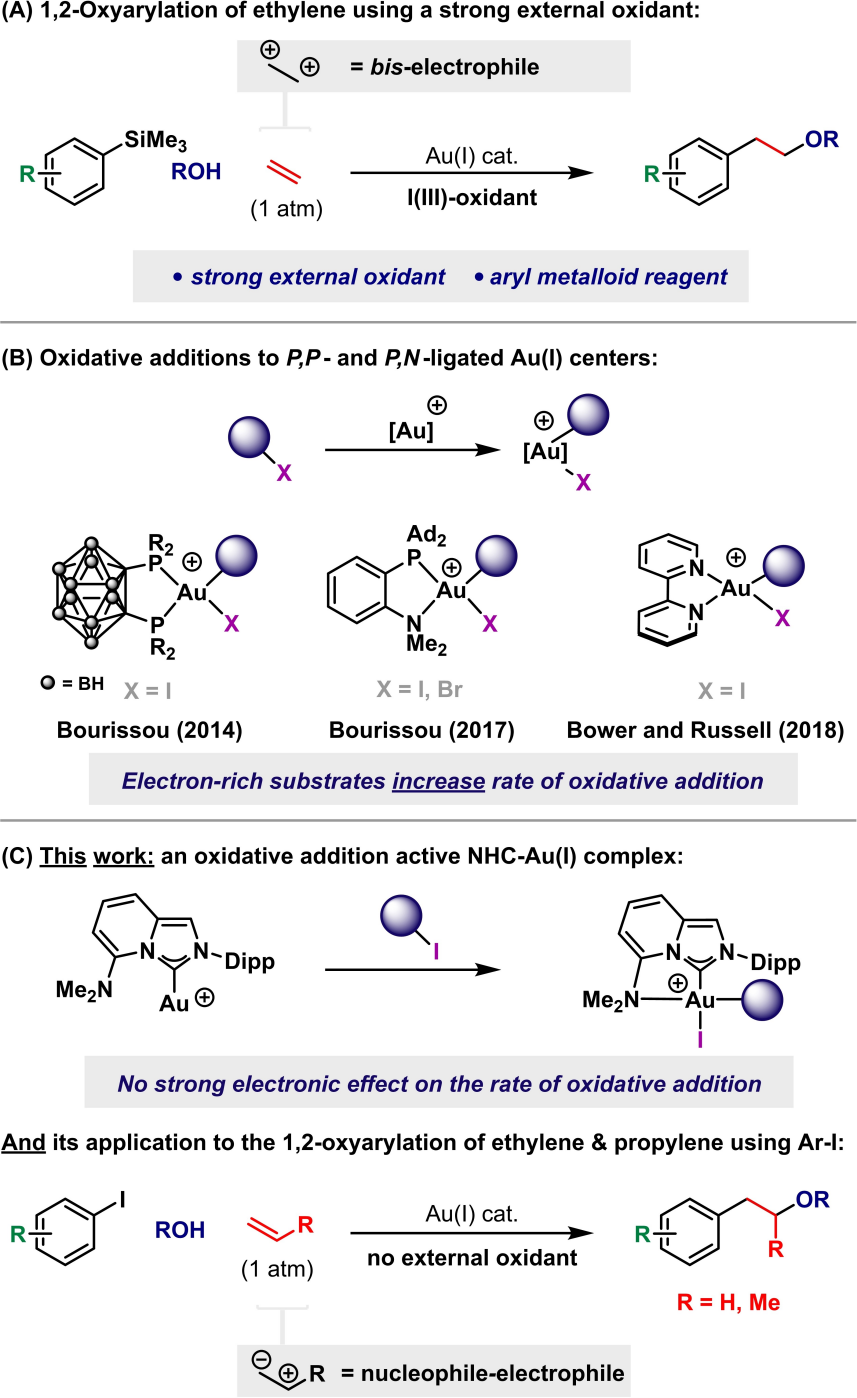
Alkene 1,2‐heteroarylations and oxidative addition active Au‐complexes.

One of the most attractive characteristics of gold catalysis is its ability to construct molecular complexity from simple organic substrates.^[1]^ In many gold catalyzed reactions the gold center acts as a strong but benign Lewis acid to activate π‐systems.^[2]^ Although isohypsic processes of this type are significant, π‐activations of increasing synthetic scope and power can be facilitated by instead harnessing the redox activity of the gold center.[^3^, ^4^] Because of the distinct reactivity profile of gold, this approach can deliver striking new catalytic transformations, with processes that allow the valorization of commodity chemicals (e.g., ethylene and propylene) being especially significant.^[5]^ Indeed, we reported a gold‐catalyzed oxyarylation where ethylene acts as a *bis*‐*electrophilic* two carbon building block (Scheme 1A).^[6]^ This offers a rare method for the catalytic difunctionalization of this feedstock chemical, but the requirement of a strong I^III^‐based oxidant to drive the Au^I/III^ redox couple has repercussions for atom economy, functional group tolerance and safety. To address this, we and others have sought to develop ligand systems that attenuate the natural reluctance of Au^I^ complexes to participate in conventional oxidative addition processes (Scheme 1B).^[7]^ Bourissou et al. reported a diphosphino‐carborane Au^I^ complex that, upon activation, readily undergoes oxidative addition of C(*sp* ^2^)−I and strained C−C bonds.^[8]^ Similar observations were made using the hemi‐labile *P*,*N*‐ligand, MeDalPhos,^[9]^ whereas we utilized cationic bipyridyl gold complexes to enable the oxidative addition of aryl‐, vinyl‐ and alkynyl‐iodides to gold.^[10]^ For these systems, the lability of the N‐donor plays a key role in facilitating reactivity.[^9a^, ^10c^]

These recently discovered oxidative addition processes have the potential to underpin catalytic methodologies where strong external oxidants (e.g., I^III^ reagents) are replaced with weak internal variants (e.g., aryl halides). P^N ligands have dominated this nascent area of chemistry, allowing a range of new external oxidant free processes,[^9^, ^11^] including an emerging class of alkene 1,2‐carbo‐ and hetero‐arylation reactions.^[12]^ Clearly varying the ligand donor atoms has great potential for expanding the scope of reactions that can be delivered. In particular, the use of singlet carbenes as one of the donor arms is underexplored. Bourissou and co‐workers reported Au^I^ complexes modified with mesoionic carbene ligands bearing hemilabile *N*‐groups (MIC^N); however, these systems were not effective for oxidative addition of iodobenzene.^[13]^ Recently, Valdés, Guisado‐Barrios, Ribas and co‐workers reported two new (MIC^N)Au(I) complexes bearing hemilabile (pyridine or pyrimidine) groups.^[14]^ The viability of oxidative addition of aryl iodides was demonstrated and applied to catalytic arylation‐lactonization reactions of γ‐alkenoic acids. In this study, we (1) outline the development of a (C^N)Au(I) complex bearing a hemilabile amine, (2) demonstrate that it is competent for the oxidative addition of aryl iodides and (3) show that it is highly effective for the demanding catalytic oxyarylations of both ethylene and propylene (Scheme 1C). During the preparation of this manuscript, Zhang, Szostak and co‐workers reported overlapping studies, and applied the catalyst system to aryl C−H arylation reactions.^[15]^ Our fundamental insights are complementary to their observations, offering, *inter alia*, unequivocal demonstration of the oxidative addition process. Our methodology application is also both distinct and significant, because it establishes ethylene and propylene as *nucleophilic‐electrophilic* building blocks in catalytic reaction design.

## Results and Discussion

**Scheme 2:**
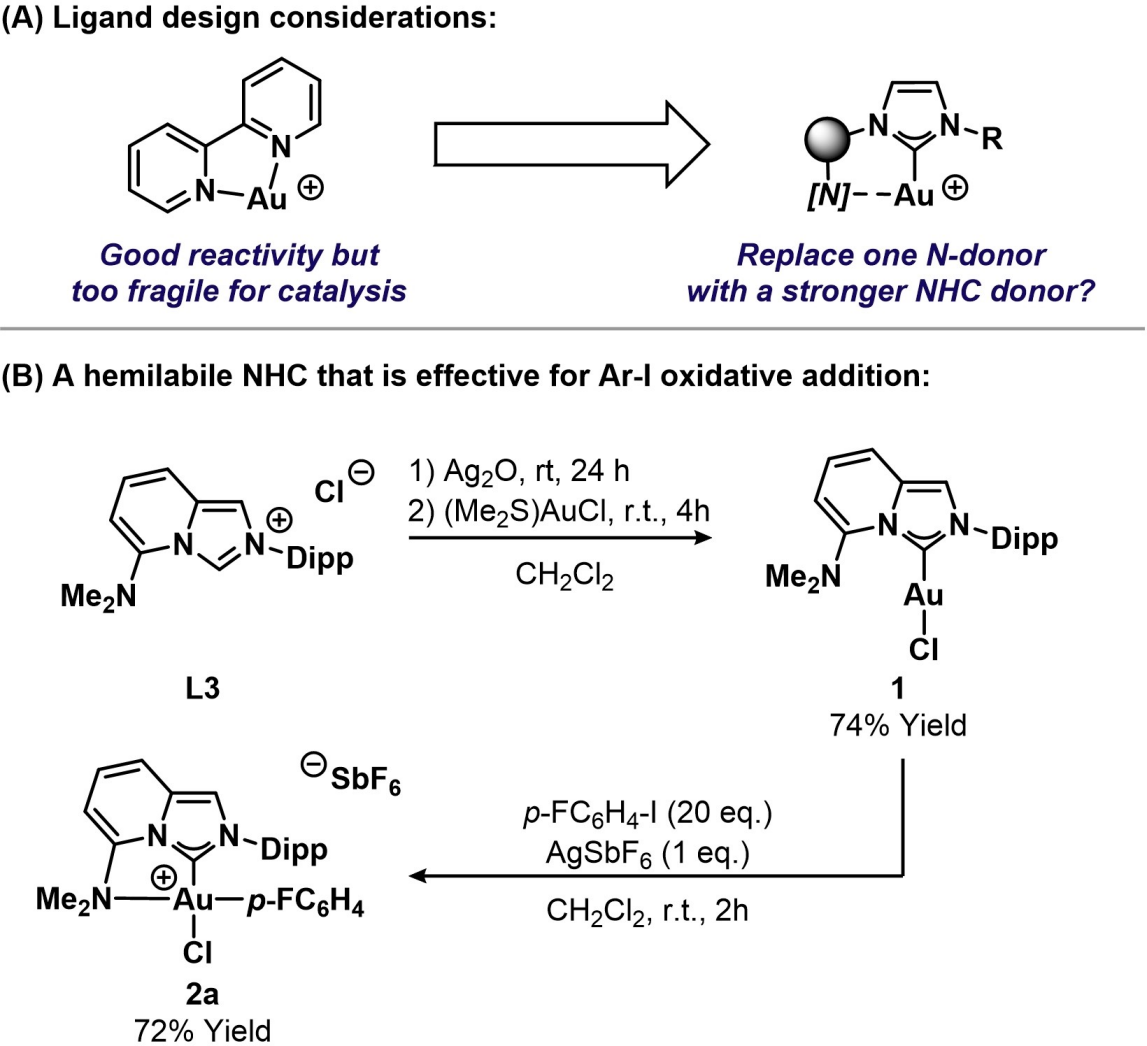
Ligand design considerations and the synthesis and evaluation of NHC‐gold(I) complex **1**.

A number of key ligand design properties have been identified that enable aryl iodide oxidative addition at Au^I^ centers. Principally, a bidentate ligand with a small bite angle is required to give a bent ML_2_ complex, and facilitate oxidative addition.^[8a]^ This observation previously led us to explore the chemistry of [(bipy)Au(I)]^+^ and related complexes.^[10]^ Although this approach was effective for aryl iodide oxidative addition and downstream mechanistic steps, we were unable to achieve catalysis, likely due to the lability of the N‐donor units, which imparts substantial fragility upon the complex. DFT analysis of the aryl iodide oxidative addition process indicates the importance of retaining one hemilabile N‐donor,^[10a]^ an observation consistent with efficacy of MeDalPhos in Bourissou and co‐workers’ studies.^[9]^ Accordingly, we considered ligand designs where one of the bipy *N*‐donors is replaced by an *N*‐heterocyclic carbene (Scheme 2A). It was hoped that the more sterically demanding and stronger NHC donor unit would stabilize the complex sufficiently for catalysis, while the retention of a hemilabile *N*‐donor would facilitate oxidative addition.

Based on the broad outline in Scheme 2A, a variety of different hemilabile NHC‐Au^I^ complexes were designed and synthesized, and then evaluated for aryl iodide oxidative addition (see the Supporting Information). Amongst these, we prepared complex **1**, which is modified with a ligand based on the Lassaletta NHC framework (Scheme 2B).^[16]^ The requisite imidazolium salt **L3** was easily prepared in two steps from 6‐(dimethylamino)picolinaldehyde **L1** (see the Supporting Information). Conversion of this to complex **1** was achieved in 74 % yield via transmetalation from the corresponding Ag^I^ complex.

The behavior of **1** towards oxidative addition with aryl iodides was examined by reaction with 4‐fluoroiodobenzene in the presence of AgSbF_6_ at room temperature (Scheme 2B). Analysis of the reaction mixture by ^19^F NMR spectroscopy showed a single F‐containing product, **2 a**, formed in 72 % yield with a new signal at −116.3 ppm. The ^13^C{^1^H} NMR spectrum of **2 a** showed a significant upfield shift of the carbenic carbon from 164.7 to 141.0 ppm, which is consistent with binding to a Au^III^ center. An analogous reaction using AgOTf provided only 28 % conversion to the oxidative addition product after 5 hours, whereas the process was completely inhibited using AgNTf_2_. Accordingly, a weakly coordinating anion is required, presumably to enable interaction of the aryl iodide with the gold(I) center.

**Figure 1:**
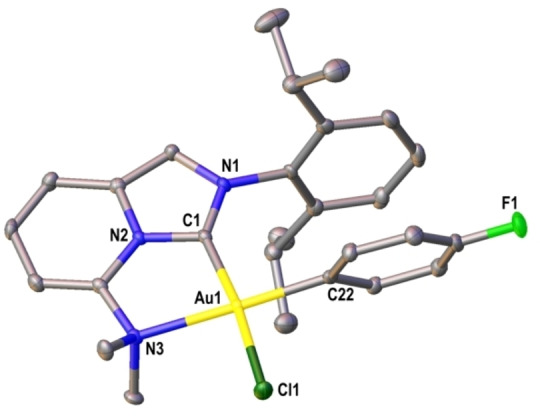
Thermal ellipsoid plot (50 % probability level) of the cation of **2 a**; counterion and H atoms omitted for clarity. Selected bond lengths [Å] and angles [°]: C1−Au1 2.007(2), N3−Au1 2.230(2), Cl1−Au1 2.3029(6), C22−Au1 2.026(3), C1−Au1−N3 80.13(9).

Crystals of **2 a** suitable for single‐crystal X‐ray diffraction were grown from a saturated CH_2_Cl_2_ solution layered with Et_2_O.^[17]^ The molecular structure is ion‐separated, with the cation showing the ligand κ^2^‐(*C*,*N*) mode on a distorted square planar Au^III^ center with the aryl unit *trans* to the N‐center (Figure 1).^[6]^ Interestingly, instead of the expected iodide ligand, **2 a** possesses a chloride *trans* to the carbene. Mass spectrometry confirmed that this is the predominant species in solution (calc. *m*/*z* 648.1656), with a likely pathway involving salt metathesis between the initially generated Au^III^ iodide complex and AgCl. Iodide‐chloride exchanges have been reported for other Au‐complexes,[^9a^, ^10b^] and these are likely driven by the relative stability of the Au^III^−Cl bond.^[18]^ Here, the exchange process is especially beneficial because it also stabilizes the oxidative addition product. This means that oxidative addition is not reversible (see below), which contrasts e.g., [(bipy)Au(*p*‐FC_6_H_4_)I][NTf_2_], where facile C−I reductive elimination was observed.^[10a]^ The C−Au−N bite angle of **2 a** is 80.13(9)°, which is similar to reported aryl Au^III^ complexes possessing *N*,*N*‐ and *P*,*N*‐donors (79° and 85°, respectively).[^9a^, ^10a^]

**Scheme 3:**
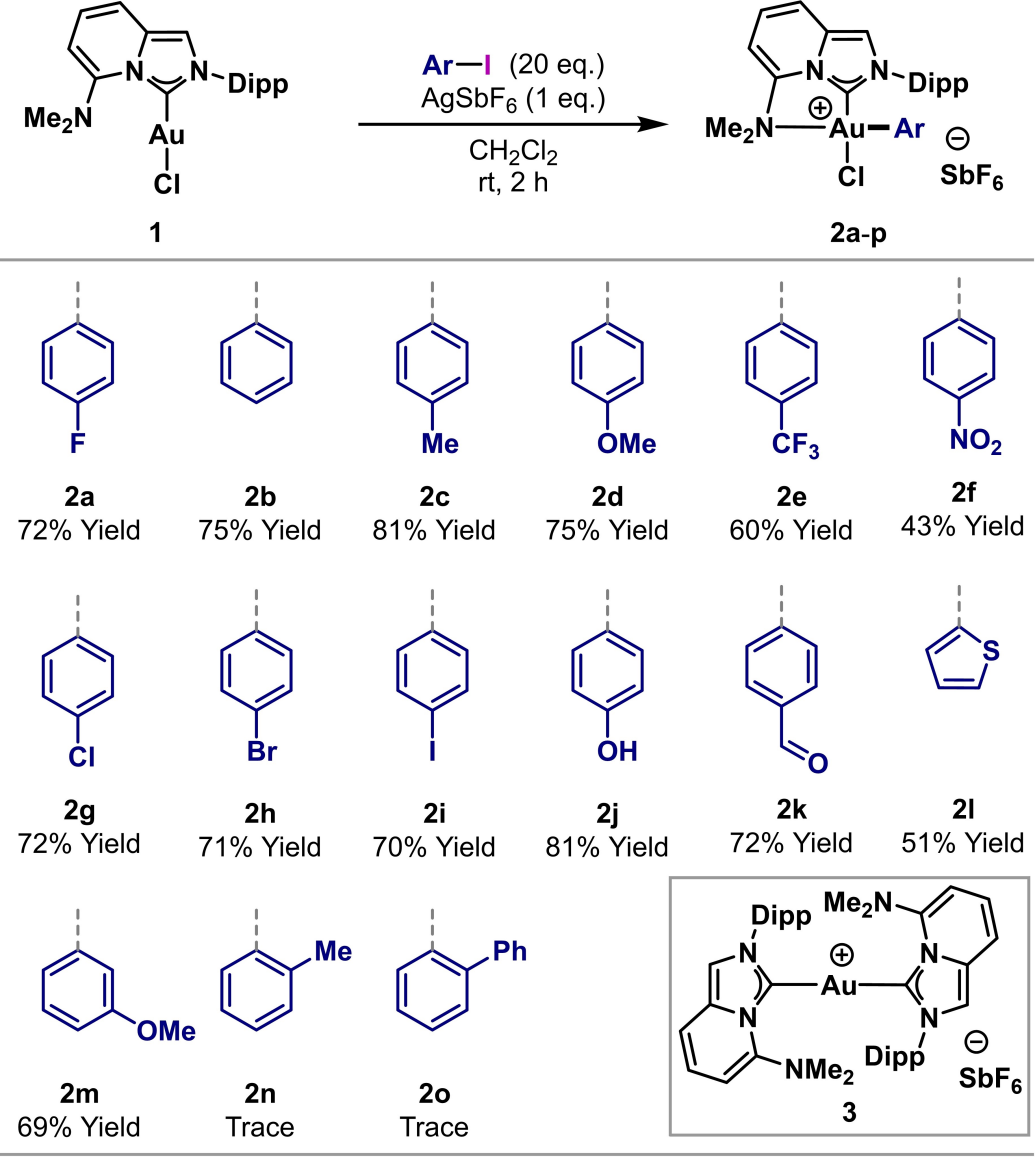
Scope of oxidative addition to NHC AuCl complex **1**. Dipp=2,6‐diisopropylphenyl. Yields were determined by ^1^H NMR spectroscopy using 1,3,5‐trimethoxybenzene as an internal standard. Isolated yields are reported in the Supporting Information. The structures of **2 a**, **2 d**, **2 f** and **2 i** were confirmed by single crystal X‐ray diffraction.

The scope of the oxidative addition using **1** was examined further (Scheme 3). The process tolerates a wide electronic window, such that a range of *p*‐substituted aryl iodides participated, giving complexes **2 b**–**i** in 43–81 % yield. Notably, aryl iodides with oxidatively sensitive functionality, e.g., alcohols and aldehydes, are well tolerated; for example, complexes **2 j** and **2 k** were formed in 81 % and 72 % yield, respectively. Heterocyclic and *m*‐substituted systems also underwent oxidative addition in 51 % (**2 l**) and 69 % (**2 m**) yield, respectively. The process is, however, sensitive to steric hindrance, such that *o*‐substituted iodoarenes provided only trace amounts of complexes **2 n** and **2 o**. In both cases, mass spectrometric analysis indicated that the dominant product was the bis‐NHC cationic complex **3** [calc. *m*/*z*=839.4076, found: *m*/*z*=839.4036]. Reaction of **1** with 4‐iodoaniline did not give the corresponding oxidative addition product, presumably because of competing coordination of the NH_2_ unit to the Au‐center. Attempted oxidative addition of an aryl bromide (4‐fluorobromobenzene) was not efficient, resulting in only traces of product.

**Figure 2:**
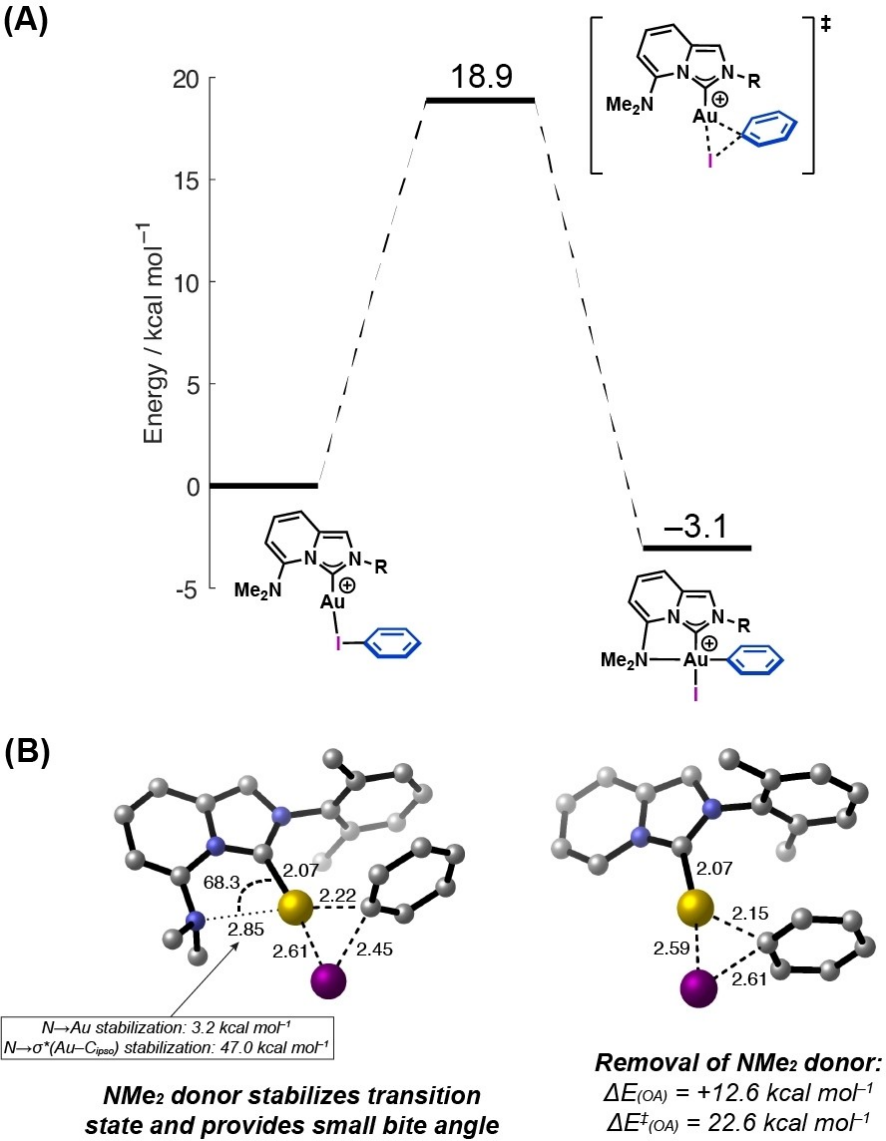
A) Calculated potential energy surface for the oxidative addition of iodobenzene. Ligand with R=2,6‐dimethylphenyl was used as a model system for ligand **L3**. B) Transition state geometries for oxidative addition with (left) and without (right) NMe_2_ donor group. Bond lengths are given in [Å] and bond angles are given in [°]. Calculations performed using the ωB97‐XD functional, a def2‐TZVP basis set with an associated 60‐election pseudopotential on Au, def2‐SVP with an associated 28‐electron pseudopotential on I, def2‐SVP on C and N and def2‐SV on all other atoms. The effects of solvation were modelled using the SMD solvation model (CH_2_Cl_2_). Energies shown include zero‐point energy corrections.

Previous reports have demonstrated that oxidative addition of aryl iodides to Au^I^‐centers is faster for more electron‐rich substrates.[^8a^, ^9b^, ^10c^] Significantly, this contrasts trends that are typical of Pd‐mediated processes, where electron poor aryl halides undergo oxidative addition more rapidly.^[19]^ The unusual behavior of Au^I^ systems has been rationalized on the basis that electron donation from the C_*ipso*_−I unit of the aryl iodide to the electropositive Au‐center is a key factor in facilitating oxidative addition.^[10c]^ To probe this, the potential energy surface for the oxidative addition of iodobenzene to a cationic [NHC−Au]^+^ fragment was investigated computationally using DFT at the ωB97‐XD level of theory with a CH_2_Cl_2_ solvation model (Figure 2A). Following κ‐*I*‐coordination of iodobenzene, oxidative addition is moderately exothermic (Δ*E*=−3.1 kcal mol^−1^). The barrier for the forward reaction is 18.9 kcal mol^−1^, with the microscopic reverse being 22.0 kcal mol^−1^. These barriers are larger than seen in the equivalent oxidative addition with MeDalPhosAuCl, at 8.2 kcal mol^−1^ and 19.2 kcal mol^−1^, respectively.^[9a]^ The increased σ‐donor capacity of the NHC likely stabilizes the intermediate and product, raising the energy barrier. The small computed thermodynamic driving force for oxidative addition indicates that it could be reversible; however, as noted earlier, salt metathesis with AgCl converts the initially formed Au^III^‐iodide complex into the more stable Au^III^‐chloride complex **2 a**. We did not observe C−Cl reductive elimination from this species. This stabilization mechanism is potentially significant and has implications for catalysis (see below).

To probe electronic effects, the oxidative addition barriers for 4‐iodoanisole versus 4‐trifluoromethyliodobenzene were compared (see Supporting Information). This revealed a very small energy difference (0.4 kcal mol^−1^), which is lower than that computed for the Au‐bipy system in Scheme 1B (2.7 kcal mol^−1^).^[10a]^ The computational analysis was validated via a competition experiment, wherein equimolar quantities of 4‐iodoanisole and 4‐trifluoromethyl‐iodobenzene were exposed to a CH_2_Cl_2_ solution of **1** and AgSbF_6_ for 2 hours. This resulted in a 1 : 1 ratio of **2 d** : **2 e**, supporting the idea that the electronics of the aryl unit have minimal influence on the rate of oxidative addition to **1**. Initial rate data were also consistent with this (see the Supporting Information). This distinct behavior versus previously reported “oxidative addition active” Au^I^‐complexes (see Scheme 1B) may reflect the strong σ‐donor properties of the NHC ligand. This is expected to reduce the electropositivity at the Au‐center, such that electron donation from the C_*ipso*_−I unit of the aryl iodide is a less prominent factor, and therefore aryl electronic effects are less pronounced.

The role of the hemi‐labile NMe_2_ group of complex **1** has also been investigated computationally (Figure 2B). In the absence of this unit, the barrier to oxidative addition is slightly higher (Δ*E* ^≠^=22.6 kcal mol^−1^ vs 18.9 kcal mol^−1^). However, the most pronounced difference is that the overall reaction is significantly more endothermic (Δ*E*=12.6 kcal mol^−1^ vs −3.1 kcal mol^−1^). Additionally, for complex **1**, which bears the NMe_2_ group, Natural Bond Orbital (NBO) analysis revealed N→Au {Δ*E*(2)=3.2 kcal mol^−1^} and N→σ*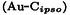 {Δ*E*(2)=47.0 kcal mol^−1^} stabilizing interactions in the transition state and Au^III^ oxidative addition product, respectively. This shows that the second donor atom has an influence on both the barrier and the overall thermodynamics of the process. Indeed, the results mirror previous studies where oxidative addition requires a second donor to provide a small bite angle (here, ∠C_NHC_−Au−N=68.3° and 79.3° for the transition state and Au^III^ product, respectively).[^8^, ^9^, ^10^, ^14^]

To probe the potential of complex **1** for catalysis, we explored the feasibility of 1,2‐oxyarylations of ethylene. As noted in our earlier work, catalytic 1,2‐difunctionalizations of this feedstock chemical are highly challenging.[^6^, ^20^] Within the context of Au‐catalyzed 1,2‐oxyarylation, key issues include the facts that gaseous ethylene (a) is expected to be present in solution in low concentrations compared with other reaction partners, and (b) lacks the stabilizing substituents on the alkene that have been employed in previous oxidative 1,2‐oxyarylations.^[21]^ These issues have a direct impact on turnover numbers; indeed, for the process in Scheme 1A, very careful selection of oxidant and ancillary ligand was required to prolong the life of the catalyst. Accordingly, the possibility of oxidant free 1,2‐oxyarylations of ethylene with complex **1** was considered tentative. It was hoped that the strong donor properties of the NHC unit would stabilize the complex sufficiently for high turnover numbers, and the aforementioned observations regarding the halide metathesis driven irreversibility of Ar−I oxidative addition would facilitate catalysis.

**Scheme 4:**
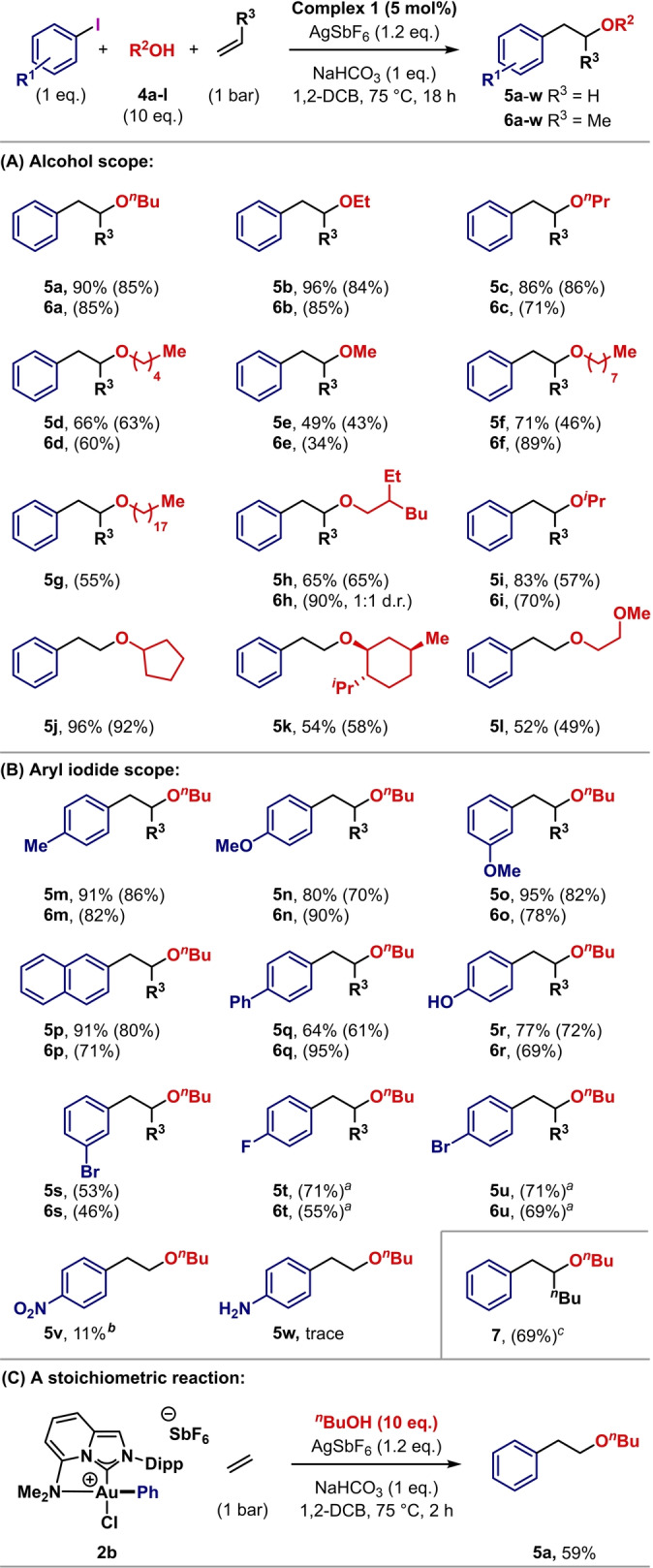
1,2‐Oxyarylation of ethylene and propylene catalyzed by **1**. Yields were determined by ^1^H NMR spectroscopy using 1,3,5‐trimethoxybenzene as an internal standard. Isolated yields are denoted in parentheses. ^a^ **1** (10 mol %) was used. ^*b*^ The product was not isolated. ^*c*^ Using hex‐1‐ene (2 equiv).

In the event we established simple and highly effective conditions for the target process. Ethylene (1 bar) was exposed to iodobenzene (1 equiv) and *n*‐butanol in the presence of AgSbF_6_ (1.2 equiv), NaHCO_3_ (1 equiv) and complex **1** (5 mol %). At 75 °C in 1,2‐dichlorobenzene (1,2‐DCB), these conditions delivered target **5 a** in 90 % yield, as determined by GC analysis (Scheme 4A). Full optimization studies are detailed in the Supporting Information. To provide a point of comparison to the most established “oxidative addition active” Au^I^‐complex, **1** was replaced with (MeDalPhos)AuCl,^[9]^ and this gave **5 a** in only 32 % yield. The protocol using **1** is very simple, and a range of other primary and secondary alcohols (**4 b**–**l**), including structurally complex variants (e.g., **4 k**), participated efficiently to deliver targets **5 b**–**l** in 34–96 % yield. The scope of the aryl iodide component mirrors the oxidative addition studies presented in Scheme 3. Electron rich (e.g., **5 b**) and moderately electron poor (e.g., **5 t**) systems participate efficiently (Scheme 4B), whereas very electron poor systems, such as 4‐iodonitrobenzene (**5 v**), are less suitable, and basic nitrogen centers inhibit catalysis (**5 w**). The very high selectivity of complex **1** for Ar−I bonds means that aryl bromide substituents are inert and this allows access to products (e.g., **5 s** and **5 u**) that have a handle for further diversification. Significantly, the process offers comparable efficiencies for 1,2‐oxyarylations of propylene. This is demonstrated by an analogous scope evaluation leading to products **6 a**–**u**. In each case complete selectivity for the indicated regioisomer was observed, wherein the new C−C bond forms at the less hindered end of the alkene. To demonstrate the potential of using higher alkenes, 1,2‐oxyarylation of hex‐1‐ene was explored, and this gave **7** in 69 % yield and as a single regioisomer. In this case, the lower volatility of the alkene meant that only two equivalents of this component were required.

To probe the mechanism of these oxyarylation reactions, we undertook a stoichiometric study, wherein oxidative addition complex **2 b** was exposed to *n*‐butanol and ethylene under the optimized catalysis conditions, but in the absence of aryl iodide and complex **1** (Scheme 4C). This experiment delivered 1,2‐oxyarylation product **5 a** in 59 % yield. **2 b** was also a competent precatalyst for the conversion of iodobenzene to **5 a**. Accordingly, under catalytic conditions, it is likely that Ar−I oxidative addition precedes π‐coordination of the alkene. The π‐complex is then attacked in an outer sphere process by the alcohol, prior to C−C reductive elimination.^[22]^ For substituted alkenes, attack of the alcohol is expected to exhibit Markovnikov‐like selectivity due to the polarizing effects of the electropositive Au‐center. This accounts for the regioselectivities observed for propylene and hex‐1‐ene. An analogous sequencing was proposed in our earlier work.^[6]^ In this case, the combination of an aryl silane and an I^III^‐oxidant provided the key aryl‐Au^III^ intermediate—the protocol described in Scheme 4 is evidently preferable.^[23]^

## Conclusion

The present study outlines our efforts to develop a hemi‐labile NHC system that can mediate aryl halide oxidative addition at Au^I^ centers. In the event, very specific ligand structural features were required, leading to the identification of easily accessible complex **1**. The nominal cation of this system is effective for the oxidative addition of a wide electronic range of aryl iodides, and the resulting complexes have been isolated and characterized by single crystal X‐ray diffraction. In contrast to previous studies, the rate of oxidative addition is not subject to strong electronic effects.[^8a^, ^9b^, ^10c^] Interestingly, the initially formed Au^III^‐iodide complex undergoes facile halide exchange to deliver the more stable Au^III^‐chloride complex. At this stage, the delivery of the aryl unit onto the Au‐center becomes irreversible and this may have implications for catalysis. The mechanism of the oxidative addition process has been probed computationally, and this has confirmed the importance of the hemilabile NMe_2_ unit. The utility and robustness of **1** has been demonstrated by its application to the 1,2‐oxyarylation of ethylene and propylene. These are particularly demanding substrates, yet complex **1** performs admirably. The new protocol offers a rare method for the catalytic difunctionalization of these feedstock alkenes, and also addresses issues associated with our previously reported oxidative protocol, which was suboptimal because it required a strong I^III^‐oxidant.^[6]^ More broadly, these studies demonstrate the value in developing ligand systems that enable Pd‐like reactivity at Au^I^‐centers. With respect to the 1,2‐oxyarylation processes, an appealing balance is struck, wherein the Au^I^ system promotes Ar−I oxidative addition, but does not suffer from competing downstream β‐hydride elimination pathways. The latter would likely thwart attempts to promote similar difunctionalizations using Pd^0^‐catalysis.

## Conflict of interest

The authors declare no conflict of interest.

1

## Supporting information

As a service to our authors and readers, this journal provides supporting information supplied by the authors. Such materials are peer reviewed and may be re‐organized for online delivery, but are not copy‐edited or typeset. Technical support issues arising from supporting information (other than missing files) should be addressed to the authors.

Supporting Information

## Data Availability

The data that support the findings of this study are available in the Supporting Information of this article.
