# Supplementary material for: A Hemilabile NHC‐Gold Complex and its Application to the Redox Neutral 1,2‐Oxyarylation of Feedstock Alkenes
Source: Angew Chem Int Ed Engl. 2023 May 2;62(23):e202301526. doi: 10.1002/anie.202301526 (PMC10962591; doi:10.1002/anie.202301526)
Supplement: Supplementary file 1 — Supporting Information [file ANIE-62-0-s001.pdf]

Angewandte Chemie
Eine Zeitschrift der Gesellschaft Deutscher Chemiker

Supporting Information

A Hemilabile NHC-Gold Complex and its Application to the Redox Neutral 1,2-Oxyarylation of Feedstock Alkenes

S. C. Scott, J. A. Cadge, G. K. Boden, J. F. Bower, C. A. Russell**

Supporting Information

1. General information 2

2. Development of NHC gold complexes 2

3. Synthesis and characterization 4

3.1. Gold complex synthesis 4

3.2. Oxidative addition of aryl iodides 9

3.3. Oxidative addition of biphenylene 18

4. Oxyarylation catalysis 20

4.1. Reaction optimization 20

4.2. Oxyarylation of ethylene 24

4.3. Oxyarylation of propylene 34

4.4. Mechanistic studies on the alkene oxyarylation reactions 44

5. Mechanism of oxidative addition 48

5.1. NMR studies 48

5.2. Computational studies 51

6. Crystallographic information 65

7. NMR spectra 70

8. References 118

1. General information

All reactions conducted under inert conditions were done so under N_2 using standard Schlenk-link techniques. Anhydrous solvents (CH_2Cl_2, Et_2O and hexane) were acquired from an Anhydrous Engineering alumina column drying system and stored over 4 Å molecular sieves. Anhydrous EtOAc was attained by trying over freshly activated 4 Å molecular sieves overnight, distilled and stored over 4 Å molecular sieves. All other reagents were used as purchased from commercial suppliers and used as received. Reaction monitoring was conducted by TLC on Silica Gel F254 plates and were visualized using UV light and/or with potassium permanganate solution and heat. NMR spectra were obtained using Bruker Nano400, Jeol ECS400, Jeol ECZ400 and the Bruker Avance III HD Cryo. Chemical shifts are recorded in ppm (δ) and are referenced to the solvent peak. Coupling constants (*J*) are given in Hz and multiplicities are abbreviated as: br (broad), s (singlet), d (doublet), t (triplet), q (quartet), quintet (quin), hextet (h), m (multiplet) and combinations thereof. Assignment of novel compounds was determined with assistance of 2D NMR experiments. NMR yields were determined with the addition of a known amount of standard. Mass spectrometry was performed by the university of Bristol mass spectrometry servicey (EI^+) using a Thermo Scientific QExactive mass spectrometer with a TRACE 1310 GC attachment or (ESI^+) with either a Bruker Daltonics MicrOTOF II mass spectrometer or a Waters SYNAPT G2-S mass spectrometer. GC-FID/MS analysis was performed on an Agilent 7820A equipped with a 5977B MSD. Quantitative analysis of reaction components was determined the integral of the peak area compared to an internal standard (dodecane) using a known calibration curve. Infrared spectra were obtained using a Perkin Elmer Spectrum Two FT-IR spectrometer.

2. Development of NHC gold complexes

A range of Au-NHC complexes were evaluated for Ar-I oxidative addition, including systems that lack a pendent N-donor. The synthesis of these complexes is described later. The viability of oxidative addition with each complex was evaluated on 4-fluoroiodobenzene using the General Procedure given in Section 3.2. In most cases, no oxidative addition was evident. For complex **S5**, some evidence for oxidative addition was observed. ^{19}F NMR spectroscopy of the reaction mixture identified a new signal at -115.20 ppm, and ESI-HRMS detected a mass of m/z = 656.1556, which corresponded to $[C_{28}H_{27}N_3FClAu]^+$ (calc. m/z = 656.1543). However, compared to **1**, the process was not clean and an analytical sample of the product could not be isolated.

Gold Complex	Known?	^{19}F NMR Spectroscopic Evidence of Oxidation?	Suspected Outcome?	Changes
IPrAuCl	Yes	No	No reaction	Increase donation from ligand
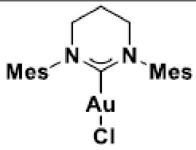 S1	Nechaev *et al.* [1]	No	No reaction	Hemi-labile group required
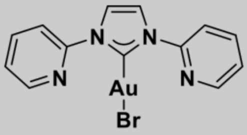 S2	No	No	Au-Ag aggregation e.g. 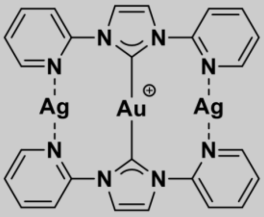 See Catalano *et al.*[2]	Remove one donating pendent group
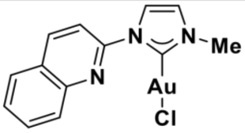 S3	Gornitzka *et al.*[3]	No	Au-Ag aggregation See above	Replacement of quinolyl group
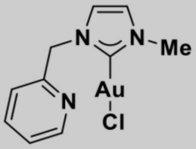 S4	No	No	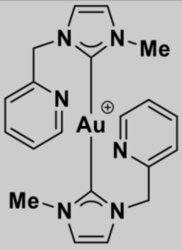	Introduction of rigid backbone; forces orientation towards gold
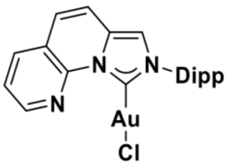 S5	No	Yes	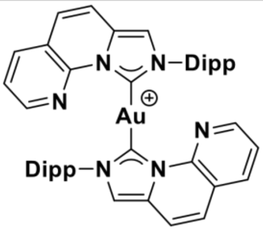	Replacement of pyridyl group with more flexible NMe_2 group
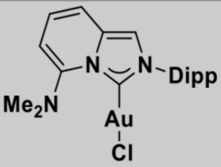 1	No	Yes	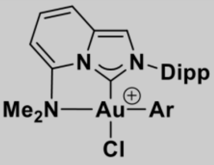 2	N/a

3. Synthesis and characterization

3.1. Gold complex synthesis

N-(2-6-diisopropylphenyl)-1-(1,8-naphthyridin-2-yl)methanimine (**S5a**) (2-dimethylaminopyridin-6-carboxyaldehyde (**L1**) were synthesized according to literature procedures.[4]

(2-Imidazol-1-yl)pyridyl (S2a)

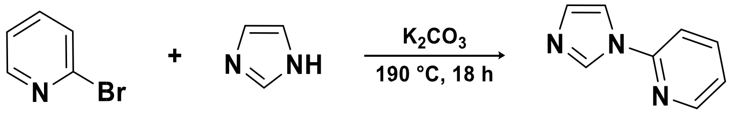

2-Bromopyridine (2.18 g, 13.8 mmol), imidazole (2.81 g, 41.3 mmol) and K_2CO_3 (3.81 g, 27.6 mmol) were added to a J. Youngs tube, under inert conditions. The neat reaction mixture was heated to 190 °C for 18 hours. After cooling to rt, the mixture was diluted with H_2O and extracted with 3×30 mL $CHCl_3$. The organic layer was collected and washed 3×30 mL saturated Na_2CO_3 solution and dried over $MgSO_4$. The solution was filtered, and the solvent was removed under reduced pressure. **S2a** was isolated as a colorless solid in quantitative yield.

^{1}H NMR (400 MHz; $CDCl_3$) δ: 8.42 (ddd, *J* = 4.9, 1.9, 0.9 Hz, 1H), 8.30 (t, *J* = 1.2 Hz, 1H), 7.76 (ddd, *J* = 8.2, 7.5, 1.9, 1H), 7.59 (t, *J* = 1.4 Hz, 1H), 7.30 (dt, *J* = 8.2, 0.9 Hz, 1H), 7.18 (ddd, *J* = 7.4, 4.9, 0.9 Hz, 1H), 7.15 (t, *J* = 1.2 Hz, 1H). The spectroscopic data is consistent with literature precedent.[5]

1,3-Bis(pyridin-2-yl)imidazolium bromide (S2b)

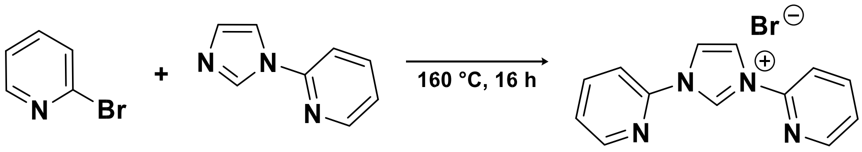

Under an N2 atmosphere, (**S2a**) (0.48 g, 3.3 mmol) was dissolved in 2-bromopyridine (0.78 g, 4.95 mmol) and sealed in a J. Youngs tube which was heated at 160 °C for 16 hours. The resulting precipitate was washed with Et_2O, filtered, and recrystallized from MeOH/Et_2O repeatedly until pure. A beige solid of **S2b** was isolated (0.61 g, 60%).

^{1}H NMR (400 MHz; DMSO-d_6) δ: 10.81 (t, *J* = 1.7 Hz, 1H), 8.75 (d, *J* = 1.7 Hz, 2H), 8.72 (dt, *J* = 4.8, 1.5 Hz, 2H), 8.36-8.18 (m, 4H), 7.71 (ddd, *J* = 7.2, 4.8, 2.6 Hz, 2H). The spectroscopic data is consistent with literature precedent.[6]

[1,3-Bis(aminopyridyl-2-yl)imidazol-2-ylidene]gold(I) bromide (S2)

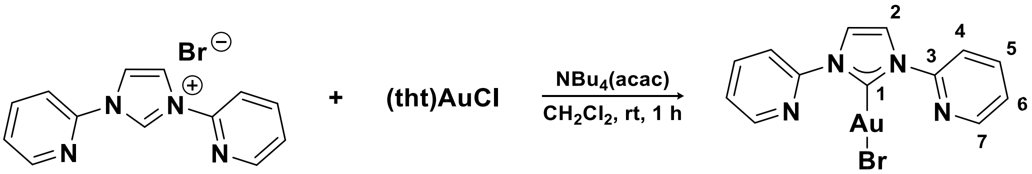

(**S2b**) (103.0 mg, 0.33 mmol), (Me_2S)AuCl (103.0 mg, 0.35 mmol) and NBu_4(acac) (0.12 g, 0.34 mmol) were dissolved in CH_2Cl_2 (10 mL) and the reaction mixture was stirred for 1 hour at rt. The reaction mixture was filtered through a silica plug and washed with 100 mL CH_2Cl_2. The solvent was removed under reduced pressure and the resulting residue was recrystallized using CH_2Cl_2. **S2** was obtained as a white solid (95.5 mg, 58%).

^{1}H NMR (400 MHz; $CDCl_3$) δ: 8.69 (d, J = 8.1 Hz, 2H, C7-H), 8.57 (d, J = 4.2 Hz, 2H, C4-H), 8.007.93 (m, 4H, C2-H and (C6-H), 7.46 (dd, J = 7.5, 4.9 Hz, 2H, C5-H), 8.68 (d, J = 8.3 Hz, 2H, C7-H), 8.58 (C4-H), 8.01-7.95 (m, 4H, C2-H and (C6-H), 7.46 (dd, J = 7.4, 4.8 Hz, 2H, C5-H). **^{13}C{^{1}H} NMR** (101 MHz; $CDCl_3$) δ: 171.9 (C1, observed by HMBC), 150.7 (C3), 149.1 (C4), 139.3 (C6), 124.6 (C5), 121.0 (C2), 117.9 (C7).

HRMS:(ESI)+ Calculated for $[C_{13}H_{11}AuBrN_4]^+$ $[M+H]^+$: 498.9827. Found: 498.9823.

3-Methyl-1-(2-picolyl)imidazolium chloride (S4a)

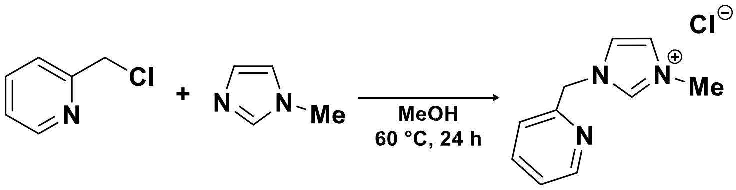

2-(Chloromethyl)pyridine (1.17 g, 9.2 mmol) was dissolved in dry MeOH (7 mL) followed by the dropwise addition of 1-methylimidazole (0.6 mL, 8.3 mmol). The reaction mixture was then heated to 60 °C for 24 hours. The solvent was removed under reduced pressure, and the resulting residue was triturated with a CH_2Cl_2/pentane (2:1) solvent mix. A hygroscopic brown solid of the desired product was isolated (1.09 g, 63%).

^{1}H NMR (400 MHz; DMSO-d_6) δ: 9.27 (s, 1H), 8.55 (d, J = 4.7 Hz, 1H), 7.89 (tt, J = 7.7, 1.6 Hz, 1H), 7.78 (app t, J = 1.8 Hz, 1H), 7.73 (app t, J = 1.8 Hz, 1H), 7.49 (d, J = 7.8 Hz, 1H), 7.44-7.36 (m, 1H), 5.57 (s, 2H), 3.89 (s, 3H). The spectroscopic data is consistent with literature precedent.[7]

[3-Methyl-1-(2-picolyl)imidazol-2-ylidene]gold(I) (S4)

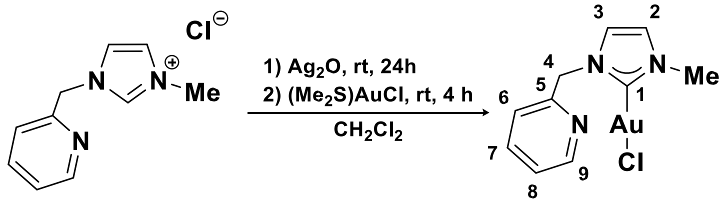

Ag_2O (78.6 mg, 0.34 mmol) and (**S4a**) (70.4 mg, 0.34 mmol) were dissolved in CH_2Cl_2 (10 mL) and stirred in the absence of light. After 24 hours, $(Me_2S)AuCl$ (110.0 mg, 0.37 mmol) was added and stirred for a further 4 hours. Upon completion, the reaction mixture was filtered through Celite and solvent reduced in volume. A precipitate formed upon layering the mixture with hexane and cooling within a fridge. A white solid of the desired product was isolated (71.9 mg, 52%). Crystals of suitable quality for X-ray diffraction analysis were obtained via layering a solution of (**S4**) in CH_2Cl_2 with pentane.

^{1}H NMR (400 MHz; $CDCl_3$) δ: 8.56 (d, *J* = 4.9 Hz, 1H, C9-H), 7.70 (app td, *J* = 7.7, 1.8 Hz, 1H, C7-H), 7.44 (d, *J* = 7.8 Hz, 1H, C6-H), 7.28-7.22 (m, 1H, C8-H), 7.17 (d, *J* = 1.9 Hz, 1H, C3-H), 6.94 (d, *J* = 1.9 Hz, 1H, C2-H), 5.44 (s, 2H, C4-H_2), 3.83 (s, 3H, C10-H_3). **^{13}C{^{1}H} NMR** (101 MHz; $CDCl_3$) δ: 171.7 (C1), 154.8 (C5), 149.9 (C9), 137.5 (C7), 123.6 (C8), 123.1 (C6), 122.1 (C2), 121.5 (C3), 56.6 (C4), 38.5 (C10).

HRMS:$(ESI)^+$ Calculated for $[C_{10}H_{11}N_3NaClAu]^+$ $[M+H]^+$: 428.0204. Found: 484.0205.

1,8-Naphthyrido[1,2-*a*]-(2′,6′-diisopropylphenyl)imidazolium chloride (S5b)

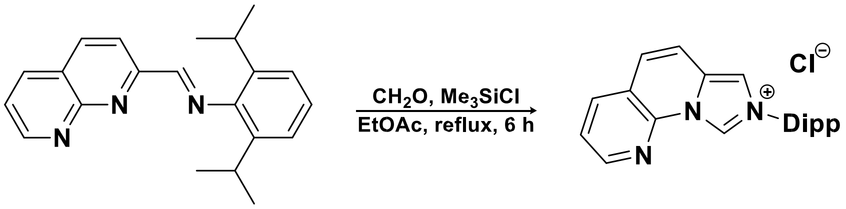

Paraformaldehyde (94.7 mg, 3.2 mmol) and (**S5a**) (1.00 g, 3.2 mmol) were dissolved in EtOAc (50 mL) and the solution was refluxed for a few minutes. Trimethylchlorosilane (0.53 mL, 5.7 mmol) was added dropwise and the reaction mixture was further stirred under reflux for an additional 6 hours. The reaction was cooled to rt, the precipitate was collected by filtration, washed with cold EtOH and dried under reduced pressure. An orange solid of the desired product was isolated (0.68 g, 58%).

^{1}H NMR (400 MHz; DMSO-d_6) δ: 10.89 (d, *J* = 1.7 Hz, 1H), 8.88 (dd, *J* = 4.7, 1.7 Hz, 1H), 8.63-8.57 (m, 2H), 7.91 (dd, *J* = 7.9, 4.7 Hz, 1H), 7.87-7.80 (m, 2H), 7.68 (t, *J* = 7.8 Hz, 1H), 7.51 (d, *J* = 7.8 Hz, 2H), 1.15 (dd, *J* = 9.0, 6.8 Hz, 12H**)**. **^{13}C {^{1}H} NMR** (101 MHz; $CDCl_3$) δ: 149.6, 145.1, 141.2, 138.5, 131.6, 131.1, 129.9, 129.5, 126.1, 125.3, 124.4, 119.9, 118.7, 116.7, 27.8, 24.1, 23.9. The spectroscopic data is consistent with literature precedent.[8]

(1,8-Naphthyrido[1,2-*a*]-(2′,6′-diisopropylphenyl)imidazolium)gold(I) chloride (S5)

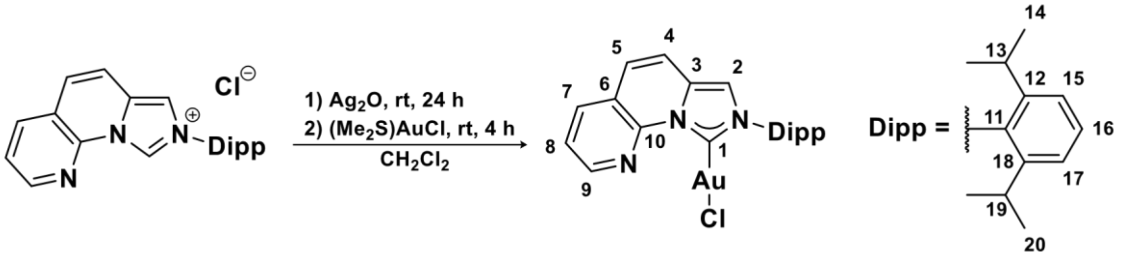

Ag_2O (129 mg, 0.56 mmol) and (**S5b**) (200 mg, 0.55 mmol) were dissolved in CH_2Cl_2 (20 mL) and stirred at rt for 24 hours. (Me_2S)AuCl (161 mg, 0.55 mmol) was added and stirred for a further 4 hours. Upon completion, the solution was filtered through Celite and concentrated under reduced pressured. Hexane (20 mL) was added, forming a precipitate. The solvent was decanted and remaining residue was washed with 3×20 mL of hexane and dried. A white solid of the desired product was isolated (231.8 mg, 75%).

^{1}H NMR (500 MHz; CD_2Cl_2) δ: 8.78 (dd, *J* = 4.8, 1.7 Hz, 1H, C9-H), 8.10 (dd, *J* = 7.7, 1.8 Hz, 1H, C7-H), 7.63 (dd, *J* = 7.8, 4.6 Hz, 1H, C8-H), 7.60 (t, *J* = 7.8 Hz, 1H, C16-H), 7.43-7.28 (m, 5H, C2-H, C4-H, C5-H, C15-H and C17-H), 2.36 (hept, *J* = 6.9 Hz, 2H, C13-H or C19-H), 1.31 (d, *J* = 6.9 Hz, 6H, C14-H_3 or C20-H_3), 1.14 (d, *J* = 6.9 Hz, 6H, C14-H_3 or C20-H_3). **^{13}C{^{1}H} NMR** (126 MHz; CD_2Cl_2**)** δ: 168.4 (C1), 148.8 (C9), 146.0 (C12 and C18), 144.9 (C10), 137.8 (C7), 136.3 (C11), 131.3 (C16), 130.8 (C3), 124.8 ((C4, C5) or (C15 and (C17)), 124.8 ((C4, C5) or (C15 and C17)), 124.3 (C8), 120.4 (C6), 117.2 (C2), 29.0 (C13 and C19), 24.6 (C14 or C20), 24.5 (C14 or C20).

HRMS:$(ESI)^+$ Calculated for $[C_{22}H_{23}N_3ClAuNa]^+$ $[M+Na]^+$: 584.1144. Found: 584.1128.

2-Dimethylaminopyridine-6-(2′,6′-diisopropylphenyl)imine (L2)

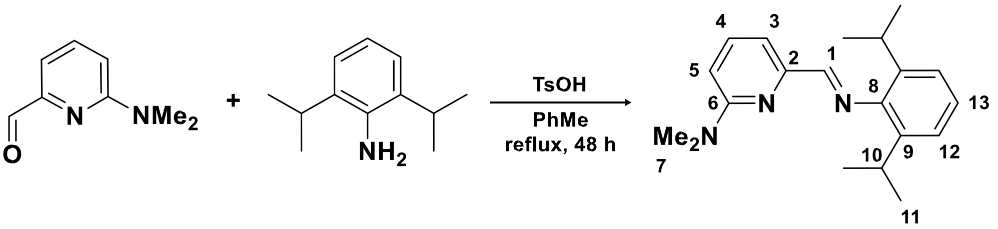

(**L1**) (2 g, 13.3 mmol) and diisopropylaniline (2.5 mL, 13.3 mmol) and tosic acid (100 mg, 0.6 mmol) was dissolved in toluene (150 mL). The reaction mixture was refluxed using a Dean-Stark apparatus for 48 hours. The reaction mixture was cooled to rt, filtered and the solvent was removed. The resulting brown solid (3.85 g, 94%) was used without further purification.

^{1}H NMR (500 MHz; $CDCl_3$) δ: 8.16 (s, 1H, C1–H), 7.60 (app t, *J* = 7.8 Hz, C4–H), 7.53 (d, *J* = 7.3 Hz, 1H, C3–H), 7.17 (d, *J* = 7.2 Hz, 2H, C12–H), 7.12, (dd, *J* = 7.2 Hz, 1H, C13–H) 6.63 (d, *J* = 8.4 Hz, 1H, C5–H) 3.14 (s, 6H, C7–H_3), 3.01 (heptet, *J* = 6.9 Hz, 2H, C10–H), 1.19 (d, *J* = 6.9 Hz, 12H, C11–H_3) **^{13}C{^{1}H} NMR** (126 MHz; $CDCl_3$) δ: 164.4 (C1), 159.3 (C6), 152.6 (C2), 149.0 (C9), 137.7 (C4 or

C8), 137.4 (C4 or C8), 124.1 (C13), 122.9 (C12), 108.8 (C3), 107.3 (C5), 37.9 (C7), 27.9 (C10), 23.5 (C11).

HRMS: $(ESI)^+$ Calculated for $[C_{20}H_{28}N_3]^+$: 310.2278. Found 310.2277.

2-(2′,6′-Diisopropylphenyl)-5-(imidazol[1,5-*a*]pyridine-2-ium) (L3)

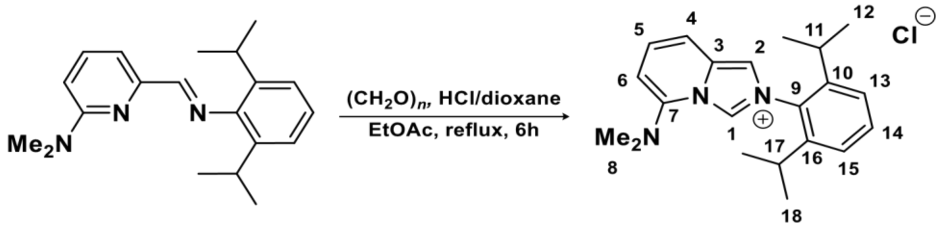

L2 (400 mg, 1.29 mmol) and paraformaldehyde (38.8 mg, 1.29 mmol) dissolved in 20 mL of dry EtOAc. Solution was refluxed for a few minutes followed by the dropwise addition of 4M HCl in dioxane (0.33 mL, 1.32 mmol). Upon this addition a precipitate began to form. After refluxing for 6 hours, the solution was cool, filtered and the precipitate was washed with EtOAc. **L3** was isolated as a beige solid (270 mg, 58%).

^{1}H NMR (500 MHz; $CDCl_3$) δ: 9.81 (d, *J* = 1.7 Hz, 1H, C1-H), 8.39 (d, *J* = 1.7 Hz, 1H, C2-H) 8.00 (d, *J* = 9.2 Hz, 1H, C4-H), 7.56 (t, *J* = 7.9 Hz, 1H, C14-H), 7.37 (dd, *J* = 9.2, 7.3 Hz, 1H, C5-H), 7.33 (app d, *J* = 7.9 Hz, 2H, C13-H and C15-H), 6.69 (d, *J* = 7.3 Hz, 1H, C6-H) 2.98 (s, 6H, C8-H_3), 2.15 (heptet, *J* = 6.8 Hz, 2H, C11-H and C17-H), 1.20 (d, *J* = 6.8 Hz, 6H, C12-H_3 or C18-H_3), 1.17 (d, *J* = 6.8 Hz, 6H, C12-H_3 or C18-H_3) **^{13}C{^{1}H} NMR** (126 MHz; $CDCl_3$) δ: 145.1 (C10 and C16), 144.3 (C7), 132.5 (C3), 132.1 (C14), 130.9 (C9), 127.4 (C5), 124.7 (C13 and C15), 124.1 (C1), 117.3 (C2), 113.7 (C4), 105.3 (C6), 42.1 (C8), 28.9 (app s, C11 and C17), 24.7 (C12 or C18), 24.4 (C12 or C18).

HRMS: $(ESI)^+$ Calculated for $[C_{21}H_{28}N_3]^+$ $[M]^+$: 322.227308, Found 322.227774.

2-(2′,6′-Diisopropylphenyl)-5-(imidazol[1,5-*a*]pyridine-2-ium)gold(I) chloride (1)

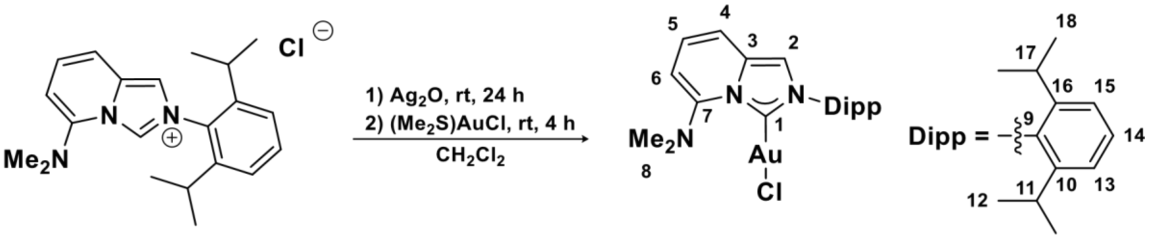

L3 (400 mg, 1.12 mmol) and Ag_2O (258.8 mg, 1.12 mmol) were dissolved in 40 mL dry CH_2Cl_2 in the absence of light at room temperature. After 24 hours, $(Me_2S)AuCl$ (329.2 mg, 1.12 mmol) was added and the reaction stirred for a further 4 hours. The solution was then filtered through Celite, washed with CH_2Cl_2 and reduced under vacuum. The product was precipitated on the addition of hexane. The

solution was then removed, the resulting solid was washed twice with hexane and dried under vacuum. was isolated as a beige solid (460 mg, 74%).

^{1}H NMR (500 MHz; $CDCl_3$) δ: 7.50 (t, J = 7.8 Hz, 1H, C14-H) 7.27 (app d, J =7.8 Hz, 2H, C13H and C15-H), 7.25 (s, 1H, C2-H), 7.17 (dd, J = 9.1, 1.0 Hz, 1H, C4-H), 6.98 (dd, J = 9.1, 7.0 Hz, 1H, C5-H), 6.27 (dd, J = 7.0, 1.0 Hz, 1H, C6-H), 2.95 (s, 6H, C8-H_3), 2.22 (hept, J = 6.8 Hz, 2 H, C11-H and C17-H), 1.32 (d, J = 6.8 Hz, 6H, C12-H_3 or C18-H_3), 1.11 (d, J = 6.8 Hz, 6H, C12-H_3 or C18-H_3). **^{13}C{^{1}H} NMR** (126 MHz; $CDCl_3$) δ: 164.7 (C1), 148.8 (C7), 145.3 (app s, C10 and C16), 135.9 (C9), 132.7 (C3), 130.7 (C14), 124.4 (C5 or (C13 and C15)), 124.3 (C5 or (C13 and C15)), 113.9 (C2), 112.6 (C4), 102.6 (C6), 44.4 (C8), 28.6 (app s, C11 and C17), 24.7 (C12 or C18), 24.5 (C12 or C18).

HRMS: $(ESI)^+$ Calculated for $[C_{12}H_{28}AuClN_3]^+$ $[M]^+$: 554.1637, Found 554.1654.

3.2. Oxidative addition of aryl iodides

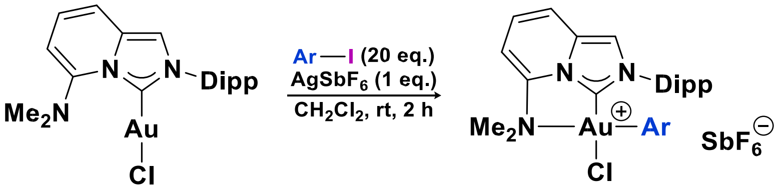

General procedure: (**1**) (20 mg, 36.1 μmol, 1 eq.) and the desired aryl iodide (0.72 mmol, 20 eq.) were dissolved in 2.5 mL dry CH_2Cl_2. Separately $AgSbF_6$ (12.4 mg, 36.1 μmol, 1 eq.) was dissolved in 2.5 mL dry CH_2Cl_2 in the absence of light. The gold solution was then added to the silver solution and stirred at room temperature for 2 hours. Afterwards, the solution was filtered through a frit, reduced in volume, and precipitated with 20 mL hexane or Et_2O. The solution was decanted, the precipitate was washed with hexane or Et_2O (2 × 20 mL) and dried.

(2a)

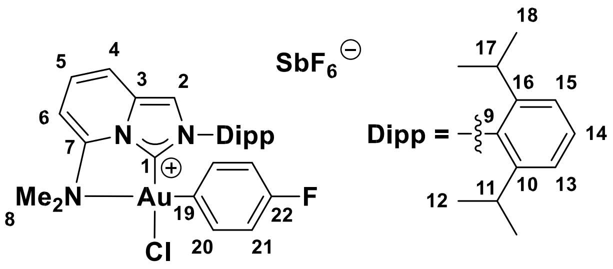

(**1**) (20 mg, 36.1 μmol, 1 eq.) and 4-fluoroiodobenzene (83 μL, 0.72 mmol, 20 eq.) were dissolved in 2.5 mL dry CH_2Cl_2. Separately $AgSbF_6$ (12.4 mg, 36.1 μmol, 1 eq.) was dissolved in 2.5 mL dry CH_2Cl_2 in the absence of light. The gold solution was then added to the silver solution and stirred at room temperature for 2 hours. Afterwards, the solution was filtered through a frit, reduced in volume, and

precipitated with 20 mL of hexane. The solution was decanted, the precipitate washed with (2 × 20 mL) hexane and dried in *vacuo*, affording the desired compound (**2a**) as a pink solid (16.3 mg, 51%). *Crystals suitable for single-crystal X-ray diffraction analysis were obtained by layering a concentration CH_2Cl_2 solution of (**2a**) with Et_2O. Crystallographic data are given in Section 6.*

^{1}H NMR (500 MHz; CD_2Cl_2) δ: 7.76 (d, *J* = 9.2 Hz, 1H, C4-H or C6-H), 7.57 (s, 1H, C2-H), 7.51 (dd, *J* = 9.2, 7.3 Hz, 1H, C5-H), 7.38-7.33 (m, 2H, (C4-H or C6-H) and C14-H), 7.04 (d, *J* = 7.9 Hz, 2H, C13-H and C15-H), 6.84 (m, 2H, C20-H), 6.55 (m, 2H, C21-H), 3.56 (s, 6H, C8-H_3), 2.20 (hept, *J* = 6.8 Hz, 2H, C11-H and C17-H), 1.25 (d, *J* = 6.8 Hz, 2H, C12-H_3 or C18-H_3), 1.00 (d, *J* = 6.8 Hz, 6H, C12-H_3 or C18-H_3). **^{13}C{^{1}H} NMR** (126 MHz; CD_2Cl_2) δ: 162.1 (d, *J* = 245.9 Hz, C22), 144.7 (C10 and C16), 143.9 (C7), 141.0 (C1), 133.3 (C9), 132.7 (d, *J* = 7.3 Hz, C20), 132.3 (C14), 132.0 (C3), 128.0 (C5), 124.8 (C13 and C15), 121.5 (C19), 119.6 (C2), 119.2 (C4 or C6), 116.8 (d, *J* = 21.9 Hz, C21), 111.7 (C4 or C6), 51.6 (C8), 29.1 (C11 and C17), 26.7 (C12 or C18), 22.3 (C12 or C18). **^{19}F NMR** (377 MHz, CD_2Cl_2) δ: -116.3

HRMS: $(ESI)^+$ Calculated for $[C_{27}H_{31}AuFClN_3]^+$ $[M]^+$: 648.1856, Found 648.1846.

(2b)

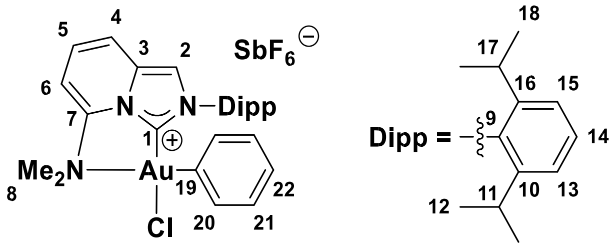

Compound (**2b**) was synthesized following the same procedure as (**2a**), reacting (**1**) (20 mg, 36.1 μmol, 1 eq.) with iodobenzene (81 μL, 0.72 mmol, 20 eq.) in the presence of $AgSbF_6$ (12.4 mg, 36.1 μmol, 1 eq.). A light pink solid of the desired product was obtained (15.6 mg, 50%).

^{1}**H NMR** (500 MHz; CD_2Cl_2) δ: 7.75 (dd, *J* = 9.2, 0.7 Hz, 1H, C4-H or C6-H), 7.56 (s, 1H, C2-H), 7.50 (dd, *J* = 9.2, 7.3 Hz, 1H, C5-H), 7.36 (dd, *J* = 7.3, 0.7 Hz, 1H, C4-H or C6-H), 7.25 (t, *J* = 7.9 Hz, 1H, C14-H), 6.98 (d, *J* = 7.9 Hz, 2H, C13-H and C15-H), 6.83 (m, 3H, C21-H and C22-H), 6.76 (m, 2H, C20-H), 3.54 (s, 6H, C8-H_3), 2.20 (hept, *J* = 6.8 Hz, 2H, C11-H and C17-H), 1.25 (d, *J* = 6.8 Hz, 6H, C12-H_3 or C18-H_3), 0.99 (d, *J* = 6.8 Hz, C12-H_3 or C18-H_3). **^{13}C{^{1}H} NMR** (126 MHz; CD_2Cl_2) δ: 144.5 (C10 and C16), 144.0 (C7), 141.6 (C1), 133.2 (C9), 132.5 (C14), 131.9 (C3), 131.7 (C21), 130.3 (C20), 129.0 (C19), 127.9 (app d, C5 and C22), 124.8 (C13 and C15), 119.7 (C2), 119.2 (C4 or C6), 111.7 (C4 or C6), 51.4 (C8), 29.1 (C11 and C17), 26.6 (C12 or C18), 22.3 (C12 or C18).

HRMS: $(ESI)^+$ Calculated for $[C_{27}H_{32}AuClN_3]^+$ $[M]^+$: 630.1962, Found 630.1962.

(2c)

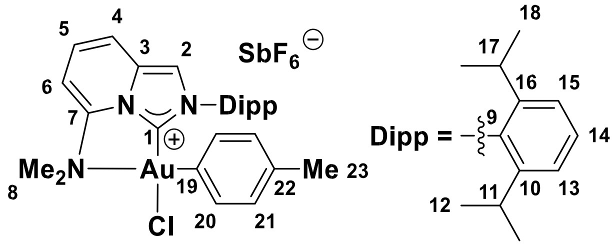

Compound (**2c**) was synthesized following the same procedure as (**2a**), reacting (**1**) (20 mg, 36.1 μmol, 1 eq.) with 4-iodotoluene (157.0 mg, 0.72 mmol, 20 eq.) in the presence of $AgSbF_6$ (12.4 mg, 36.1 μmol, 1 eq.). A light pink solid of the desired product was obtained (11.2 mg, 35%).

^{1}**H NMR** (500 MHz; CD_2Cl_2) δ: 7.75 (d, *J* = 9.2 Hz, 1H, C4-H or C6-H) 7.56 (s, 1H, C2-H), 7.49 (dd, *J* = 9.2, 7.3 Hz, 1H, C5-H), 7.36 (d, *J* = 7.3 Hz, 1H, C4-H or C6-H), 7.29 (t, *J* = 7.9 Hz, 1H, C14-H), 6.98 (d, *J* = 7.9 Hz, 2H, C13-H and C15-H), 6.68 (d, *J* = 8.2 Hz, 2H, C20-H), 6.54 (d, *J* = 8.2 Hz, 2H, C21-H) 3.53 (s, 6H, C8-H_3), 2.20 (p, *J* = 6.8 Hz, 2H, C11-H and C17-H), 2.15 (s, 3H, C23-H_3), 1.25 (d, *J* = 6.8 Hz, 6H, C12-H_3 or C18-H_3), 0.98 (d, *J* = 6.8 Hz, 6H, C12-H_3 or C18-H_3). ^{13}**C{^{1}H} NMR** (126 MHz; CD_2Cl_2) δ: 144.5 (C10 and C16), 144.0 (C7), 141.9 (C1), 137.6 (C22), 133.4 (C9), 131.9 (C3), 131.7 (C14), 131.2 (C20), 130.9 (C21), 127.8 (C5), 125.6 (C19), 124.7 (C13 and C15), 119.7 (C2), 119.1 (C4 or C6), 111.6 (C4 or C6), 51.4 (C8), 29.1 (C11 and C17), 26.7 (C12 or C18), 22.3 (C12 or C18), 20.6 (C23).

HRMS: $(ESI)^+$ Calculated for $[C_{28}H_{34}AuClN_3]^+$ $[M]^+$: 644.2107, Found 644.2101.

(2d)

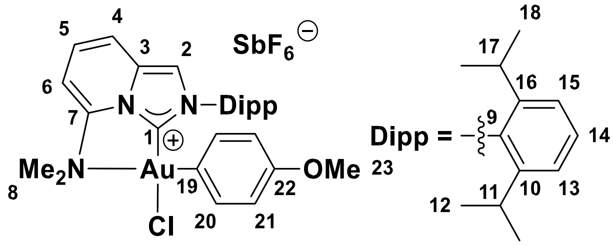

(**1**) (20 mg, 36.1 μmol, 1 eq.) and 4-iodoanisole (168.5 mg, 0.72 mmol, 20 eq.) were dissolved in 2.5 mL dry CH_2Cl_2. Separately $AgSbF_6$ (12.4 mg, 36.1 μmol, 1 eq.) was dissolved in 2.5 mL dry CH_2Cl_2 in the absence of light. The gold solution was then added to the silver solution and stirred at room temperature for 2 hours. Afterwards, the solution was filtered through a frit, reduced in volume, and precipitated with 20 mL of Et_2O. The solution was decanted, the precipitate washed with 2 × 20 mL Et_2O and dried *in vacuo,* affording the desired compound (**2d**) as a beige solid (12.0 mg, 37%). *Crystals suitable for single-crystal X-ray diffraction analysis were obtained by slow evaporation of a CD_2Cl_2 solution of (**2d**). Crystallographic data are given in Section 6.*

^{1}H NMR (500 MHz; CD_2Cl_2) δ: 7.75 (d, *J* = 9.2 Hz, 1H, C4-H or C6-H) 7.56 (s, 1H, C2-H), 7.50 (dd, *J* = 9.2, 7.3 Hz, 1H, C5-H), 7.35 (d, *J* = 7.3 Hz, 1H, C4-H or C6-H), 7.30 (t, *J* = 7.9 Hz, 1H, C14-H), 7.03 (d, *J* = 7.9 Hz, 2H, C13-H and C15-H), 6.74-6.71 (m, 2H, C20-H), 6.35-6.32 (m, 2H, C21-H), 3.68 (s, 3H, C23-H_3), 3.54 (s, 6H, C8-H_3), 2.21 (sept, *J* = 6.8 Hz, 2H, C11-H and C17-H), 1.26 (d, *J* = 6.8 Hz, 6H, C12-H_3 or C18-H_3), 0.99 (d, *J* = 6.8 Hz, 6H, C12-H_3 or C18-H_3). **^{13}C{^{1}H} NMR (**126 MHz; CD_2Cl_2) δ: 159.0 (C19), 144.6 (C10 and C16), 144.0 (C7), 141.5 (C1), 133.5 (C9), 132.1 (C14), 131.9 (C3), 131.8 (C20), 127.9 (C5), 124.8 (C13 and C15), 119.8 (C2), 119.2 (C4 or C6), 118.7 (C19), 115.7 (C21), 111.6 (C4 or C6), 55.9 (C23), 51.5 (C8), 29.2 (C11 and C17), 26.6 (C12 or C18), 22.4 (C12 or C18).

HRMS: $(ESI)^+$ Calculated for $[C_{28}H_{34}AuClN_3O]^+$ $[M]^+$: 660.2054, Found 660.2056

(2e)

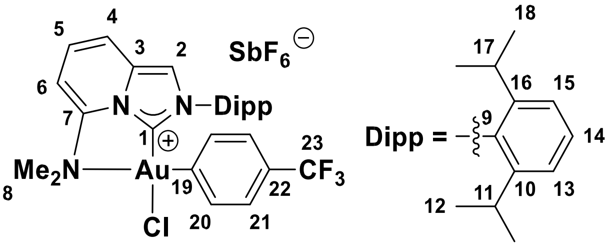

Compound (**2e**) was synthesized following the same procedure as (**2d**), reacting (**1**) (20 mg, 36.1 µmol, 1 eq.) with 4-iodotrifluorotoluene (105.8 µL, 0.72 mmol, 20 eq.) in the presence of $AgSbF_6$ (12.4 mg, 36.1 µmol, 1 eq.). A pink solid of the desired product was obtained (6.1 mg, 18%).

^{1}H NMR (500 MHz; CD_2Cl_2) δ: 7.76 (d, *J* = 9.2 Hz, 1H, C4-H or C6-H), 7.58 (s, 1H, C2-H), 7.52 (dd, *J* = 9.2, 7.3 Hz, 1H, C5-H) 7.38 (d, *J* = 7.3 Hz, 1H, C4-H or C6-H), 7.27 (t, *J* = 7.8 Hz, 1H, C14-H), 7.03 (s, 4H, C20-H and C21-H), 6.99 (d, *J* = 7.9 Hz, 2H, C13-H and C15-H), 3.58 (s, 6H, C8-H), 2.20 (hept, *J* = 6.8 Hz, 2H, C11-H and C17-H), 1.25 (d, *J* = 6.8 Hz, 6H, C12-H_3 or C18-H_3), 0.99 (d, *J* = 6.8 Hz, 6H, C12-H_3 or C18-H_3). **^{13}C{^{1}H} NMR (**126 MHz; CD_2Cl_2) δ: 144.7 (C10 and C16), 143.8 (C7), 140.9 (C1), 133.1 (C9), 132.7 (C14), 132.5 (q, *J* = 1.5 Hz, C20), 132.1 (C3 or C19), 131.9 (C3 or C19), 129.7 (m, observed by HMBC, C22 or C23), 128.1 (C5), 126.4 (q, *J* = 4.0 Hz, C21), 124.8 (C13 and C15) 119.6 (C2), 119.3 (C4 or C6), 111.8 (C4 or C6), 51.8 (C8), 29.1 (C11 and C17), 26.7 (C12 or C18), 22.3 (C12 or C18). *No signal corresponding to C23 was resolved.* **^{19}F NMR** (377 MHz, CD_2Cl_2) δ: -63.3.

HRMS: $(ESI)^+$ Calculated for $[C_{28}H_{31}AuClN_3F_3]^+$ $[M]^+$ 698.1824, Found 698.1808.

(2f)

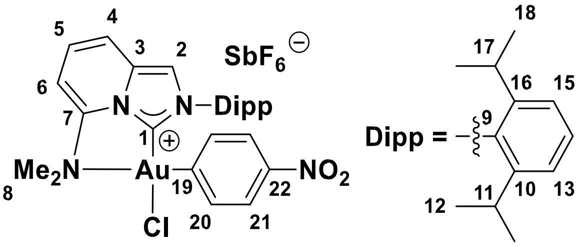

Compound (**2f**) was synthesized following the same procedure as (**2d**), reacting (**1**) (20 mg, 36.1 µmol, 1 eq.) with 1-bromo-4-iodobenzene (179.3 mg, 0.72 mmol, 20 eq.) in the presence of $AgSbF_6$ (12.4 mg, 36.1 µmol, 1 eq.). A beige solid of the desired product was obtained (11.0 mg, 33%). *Crystals suitable for single-crystal X-ray diffraction analysis were obtained by slow evaporation of a CD_2Cl_2 solution of (**2f**). Crystallographic data are given in Section 6.*

^{1}**H NMR** (500 MHz; CD_2Cl_2) δ: 7.77 (d, *J* = 9.2 Hz, 1H, C4-H or C6-H), 7.65-7.60 (m, 2H, C20-H), 7.59 (s, 1H, C2-H), 7.53 (dd, *J* = 9.2, 7.3 Hz, 1H, C5-H), 7.39 (d, *J* = 7.3 Hz, 1H, C4-H or C6-H), 7.27, (t, *J* = 7.8 Hz, 1H, C14-H), 7.15-7.09 (m, *J* =, 2H, C21-H), 6.99, (d, *J* = 7.8 Hz, 2H, C13-H and C15-H), 3.60 (s, 6H, C8-H), 2.21 (hept, *J* = 6.8 Hz, 2H, C11-H and C17-H), 1.25, (d , *J* = 6.8 Hz, 6H, C12-H_3 or C18-H_3), 1.00 (d, *J* = 6.8 Hz, 6H, C12-H_3 or C18-H_3) ^{13}**C{^{1}H} NMR** (126 MHz; CD_2Cl_2) δ: 147.4 (C22), 145.0 (C10 and C17), 143.8 (C7), 140.5 (C1), 135.1 (C19), 133.1 (C9), 132.9 (C21), 132.4 (C14), 132.2 (C3), 128.2 (C5), 124.8 (C13 and C15), 124.0 (C20), 119.5 (C2 or C4), 119.3 (C2 or C4), 111.9 (C6), 52.0 (C8), 29.1 (C11 and C17), 26.7 (C12 or C18), 22.3 (C12 or C18).

HRMS: (ESI)$^{+}$ Calculated for $[C_{27}H_{32}AuClN_4O_2]^+$ $[M]^+$: 675.1801, Found 675.1804.

(2g)

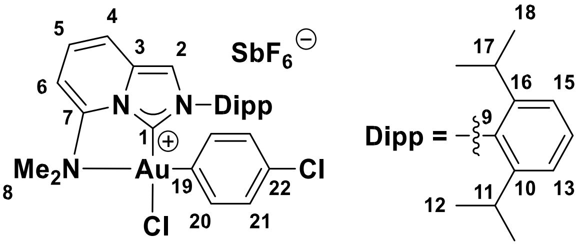

Compound (**2g**) was synthesized following the same procedure as (**2d**), reacting (**1**) (20 mg, 36.1 µmol, 1 eq.) with 1-chloro-4-iodobenzene (171.2 mg, 0.72 mmol. 20 eq.) in the presence of $AgSbF_6$ (12.4 mg, 36.1 µmol). A pink solid of the desired product was obtained (6.5 mg, 20%).

^{1}**H NMR** (500 MHz; CD_2Cl_2) δ: 7.75 (d, *J* = 9.2 Hz, 1H, C4-H or C6-H), 7.58 (s, 1H, C2-H), 7.51 (dd, *J* = 9.2, 7.3 Hz, 1H, C5-H), 7.38 (m, 2H, (C4-H or C6-H) and C14-H), 7.03 (d, *J* = 7.9 Hz, 2H, C13-H and C15-H), 6.77 (m, 4H, C20-H and C21-H), 3.56 (s, 6H, C8-H_3), 2.20 (hept, *J* = 6.8 Hz, 2H, C11-H and C17-H) 1.25 (d, *J* = 6.7 Hz, 6H, C12-H_3 or C18-H_3), 1.00 (d, *J* = 6.8 Hz, 6H, C12-H_3 or C18-H_3). ^{13}**C{^{1}H} NMR (**126 MHz; CD_2Cl_2) δ: 144.7 (C10 and C16), 143.9 (C7), 141.1 (C1), 133.7 (C22), 133.3

(C9), 132.8 (C20 or C21), 132.1 (C3 or C14), 132.0 (C3 or C14), 129.8 (C20 or C21), 128.0 (C5), 125.6 (C19), 124.8 (C13 and C15), 119.6 (C2), 119.2 (C4 or C6), 111.8 (C4 or C6), 51.7 (C8), 29.1 (C11 and C17), 26.7 (C12 or C18), 22.3 (C12 or C18).

HRMS: $(ESI)^+$ Calculated for $[C_{27}H_{31}AuCl_2N_3]^+$ $[M]^+$: 664.1561, Found 664.1546.

(2h)

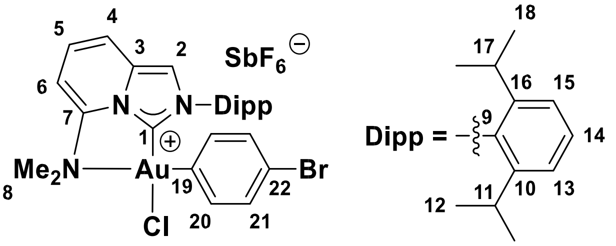

Compound (**2h**) was synthesized following the same procedure as (**2d**), reacting (**1**) (20 mg, 36.1 µmol, 1 eq.) with 1-bromo-4-iodobenzene (203.7 mg, 0.72 mmol, 20 eq.) in the presence of $AgSbF_6$ (12.4 mg, 36.1 µmol, 1 eq.). A pink solid of the desired product was obtained (14.0 mg, 39%).

^{1}**H NMR** (500 MHz; CD_2Cl_2) δ: 7.76 (d, J = 9.2 Hz, 1H, C4-H or C6-H), 7.58 (s, 1H, C2-H), 7.51 (dd, J = 9.2, 7.3 Hz, 1H, C5-H), 7.41 (t, J = 7.9 Hz, 2H, C14-H), 7.37 (d, J = 7.3 Hz, 1H, C4-H or C6-H), 7.04 (d, J = 7.9 Hz, 2H, C13-H and C15-H), 6.90-6.87 (m, 2H, C21-H) 6.75-6.72 (m, 2H, C20-H), 3.56 (s, 6H, C8-H_3), 2.19 (sept, d, J = 6.7 Hz, 2H, C11-H and C17-H), 1.25 (d, J = 6.7 Hz, 6H, C12-H_3 or C18-H_3), 1.00 (d, J = 6.7 Hz, 6H, C12-H_3 or C18-H_3). **^{13}C{^{1}H} NMR (**126 MHz; CD_2Cl_2) δ: 144.7 (C10 and C16), 143.9 (C7), 141.2 (C1), 133.2 (C9), 133.1 (C20), 132.8 (C21), 132.1(C14), 132.0 (C3), 128.0 (C5), 126.5 (C19), 124.9 (C13 and C15), 122.0 (C22), 119.6 (C2), 119.2 (C4 or C6), 111.8 (C4 or C6), 51.7 (C8), 29.1 (C11 and C17), 26.7 (C12 or C18), 22.3 (C12 or C18).

HRMS: $(ESI)^+$ Calculated for $[C_{27}H_{32}AuBrClN_3]^+$ $[M]^+$: 708.1055, Found 708.1039.

(2i)

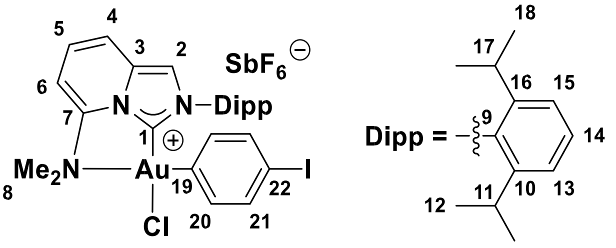

Compound (**2i**) was synthesized following the same procedure as (**2d**), reacting (**1**) (20 mg, 36.1 µmol, 1 eq.) with 1,4-diiodobenzene (237.5 mg, 0.72 mmol, 20 eq.) in the presence of $AgSbF_6$ (12.4 mg, 36.1 µmol, 1 eq.). A beige solid of the desired product was obtained (9.5 mg, 27%). *Crystals suitable for*

*single-crystal X-ray diffraction analysis were obtained by slow evaporation of a CD_2Cl_2 solution of (**2i**). Crystallographic data are given in Section 6.*

[1]**H NMR** (500 MHz; CD_2Cl_2) δ: 7.75 (d, *J* = 9.2 Hz, 1H, C4-H or C6-H), 7.58 (s, 1H, C2-H), 7.51 (dd, *J* = 9.2, 7.3 Hz, 1H, C5-H), 7.44 (d, *J* = 9.0 Hz, 1H, C14-H), 7.37 (d, *J* = 7.3 Hz, 1H, C4-H or C6-H), 7.07-7.03 (m, 4H (C13-H and C15-H) and (C20-H or C21-H)), 6.60 (app dd, *J* = 9.0, 2.8 Hz, 2H, (C20-H or C21-H), 3.55 (s, 6H, C8-H_3), 2.19 (hept, *J* = 6.8 Hz, 2H, C11-H and C17-H), 1.24 (d, *J* = 6.7 Hz, 6H, C12-H_3 or C18-H_3), 0.99, (d, *J* = 6.7 Hz, 6H, C12-H_3 or C18-H_3). **^{13}C{^{1}H} NMR (**126 MHz; CD_2Cl_2) δ: 144.7 (C10 and C16), 143.9 (C7), 141.3 (C1), 138.7 (C20 or C21), 133.4 (C20 or C21), 133.2 (C9), 132.1 (C14), 132.0 (C3), 128.0 (C5), 127.9 (C19), 124.9 (C13 and C15), 119.7

(C2), 119.2 (C4 or C6), 111.8 (C4 or C6), 93.7 (C22), 51.7 (C8), 29.1 (C11 and C17), 26.7 (C12 or C18), 22.3 (C12 or C18).

HRMS: $(ESI)^+$ Calculated for $[C_{27}H_{31}AuClIN_3]^+$ $[M]^+$: 756.0917, Found 756.0901.

(2j)

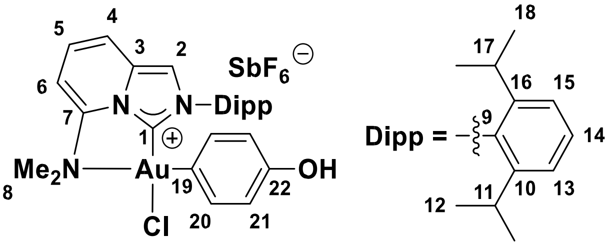

Compound (**2j**) was synthesized following the same procedure as (**2d**), reacting (**1**) (20 mg, 36.1 μmol, 1 eq.) with 4-iodophenol (158.4 mg, 0.72 mmol, 1 eq.) in the presence of $AgSbF_6$ (12.4 mg, 36.1 μmol, 1 eq.). A light pink solid of the desired product was obtained (25.5 mg, 79%).

[1]**H NMR** (500 MHz; CD_2Cl_2) δ: 7.74 (d, *J* = 9.2 Hz, 1H, C4-H or C6-H), 7.56 (s, 1H, C2-H), 7.49 (dd, *J* = 9.2, 7.3 Hz, 1H, C5-H), 7.36 (m, 2H, (C4-H or C6-H) and C14-H), 7.05 (d, *J* = 7.9 Hz, 2H, C13-H and C15-H), 6.69-6.64 (m, 2H, C20-H or C21-H), 6.29-6.26 (m, 2H, C20-H or C21-H), 4.85, (s, 1H, OH), 3.53 (s, 6H, C8-H_3), 2.20 (hept, *J* = 6.8 Hz, 2H, C11-H and C17-H), 1.25 (d, *J* = 6.8 Hz, 6H, C12-H_3 or C18-H_3), 0.99 (d, *J* = 6.8 Hz, 6H, C12-H_3 or C18-H_3). **^{13}C{^{1}H} NMR (**126 MHz; CD_2Cl_2) δ: 155.30 (C22), 144.5 (C10 and C16), 144.0 (C7), 141.6 (C1), 133.4 (C9), 132.0 (C14 or (C20 or C21)), 132.0 (C14 or (C20 or C21)), 131.9 (C3), 127.9 (C5), 124.9 (C13 and C15), 119.7 (C2), 119.2 (C4 or C6), 118.4 (C19), 117.0 (C20 or C21), 111.6 (C4 or C6), 51.5 (C8), 29.2 (C11 and C17), 26.6 (C12 or C18), 22.3 (C12 or C18).

HRMS: $(ESI)^+$ Calculated for $[C_{27}H_{32}AuClN_3O]^+$ $[M]^+$: 646.1899, Found 646.1902.

(2k)

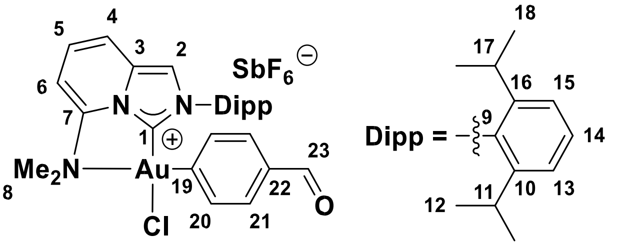

Compound (**2k**) was synthesized following the same procedure as (**2d**), reacting (**1**) (20 mg, 36.1 μmol, 1 eq.) with 4-iodobenzaldehyde (167.1 mg, 0.72 mmol, 1 eq.) in the presence of $AgSbF_6$ (12.4 mg, 36.1 μmol, 1 eq.). A beige solid of the desired product was obtained (12.0 mg, 37%).

^{1}H NMR (500 MHz; CD_2Cl_2) δ: 9.81 (s, 1H, C23-H), 7.76 (d, *J* = 9.2 Hz, 1H, C4-H or C6-H), 7.58 (s, 1H, C2-H), 7.52 (dd, *J* = 9.2, 7.3 Hz, 1H, C5-H), 7.38 (d, *J* = 7.3 Hz, 1H, C4-H or C6-H), 7.28-7.25 (m, 2H, C21-H), 7.21 (t, *J* = 7.9 Hz, 1H, C14-H), 7.11-7.07 (m, 2H, C20-H), 6.96 (d, *J* = 7.9 Hz, 2H, C13-H and C15-H), 3.58 (s, 6H, C8-H_3), 2.21, (hept, *J* = 6.8 Hz, 2H, C11-H and C17-H), 1.26 (d, *J* = 6.8 Hz, 6H, C12-H_3 or C18-H_3), 0.99 (d, *J* = 6.8 Hz, 6H, C12-H_3 or C18-H_3). **^{13}C{^{1}H} NMR (**126 MHz; CD_2Cl_2) δ: 191.5 (C23), 144.8 (C10 and C16), 143.8 (C7), 140.9 (C1), 135.7 (C19 or C22), 135.6 (C19 or C22, 133.1 (C9), 132.8 (C20), 132.4 (C14), 132.1 (C3), 130.4 (C21), 128.1 (C5), 124.8 (C13 and C15), 119.6 (C2), 119.3 (C4 or C6), 111.8 (C4 or C6), 51.8 (C8), 29.1 (C11 and C17), 26.7 (C12 or C18), 22.3 (C12 or C18).

HRMS: $(ESI)^+$ Calculated for $[C_{28}H_{32}AuClN_3O]^+$ $[M]^+$: 658.1899, Found 658.1914.

ν_{max} (neat)/cm^{-1}: 2969, 2179, 1688, 1660, 1580, 1468, 1364, 1260, 1065, 799, 659.

(2l)

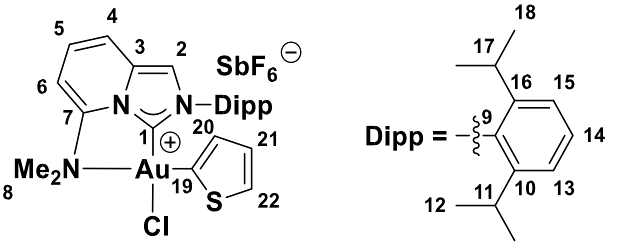

Compound (**2l**) was synthesized following the same procedure as (**2d**) reacting (**1**) (20 mg, 36.1 μmol, 1 eq.) with 2-iodothiophene (79.5 μL, 0.72 mmol, 20 eq.) in the presence of $AgSbF_6$ (12.4 mg, 36.1 μmol, 1 eq.). A yellow solid of the desired product was obtained (14.8 mg, 47%).

^{1}H NMR (500 MHz; CD_2Cl_2) δ: 7.77 (d, *J* = 9.2 Hz, 1H C4-H), 7.61 (s, 1H, C2-H), 7.52 (dd, *J* = 9.2, 7.3 Hz, 1H, C5-H), 7.42 (d, *J* = 7.4, Hz, 1H, C6-H), 7.35 (t, *J* = 7.9 Hz, 1H, C14-H), 7.20 (dd, *J* = 5.3, 1.2 Hz, 1H, (C20-H or C22-H), 7.08 (d, *J* = 7.9 Hz, 2H, C13-H and C15-H), 6.62 (dd, *J* = 5.3, 3.7 Hz, 1H, C21-H), 6.38 (dd, *J* = 3.7, 1.2 Hz, 1H, C20-H or C22-H), 3.62 (s, 6H, C8-H_3), 2.16 (hept, *J* = 7.4

Hz, 2H, C11-H and C17-H), 1.27 (d, *J* = 7.4 Hz, 6H, C12-H_3 or C18-H_3), 1.01 (d, *J* = 7.4 Hz, 6H, C12-H_3 or C18-H_3). **^{13}C{^{1}H} NMR (**126 MHz; CD_2Cl_2) δ: 144.7 (C10 and C16), 143.7 (C7), 138.8 (C1), 133.1 (C9), 132.4 (C14), 132.0 (C3), 129.0 (C20 or C22), 128.6 (C20 or C22), 128.2 (C5), 127.5 (C21), 124.8 (C13 and C15), 120.1 (C2), 119.5 (C4 or C6), 114.2 (C19), 111.9 (C4 or C6), 52.3 (C8), 29.2 (C11 and C17), 26.4 (C12 or C18), 22.7 (C12 or C18).

HRMS: $(ESI)^+$ Calculated for $[C_{25}H_{30}AuClN_3S]^+$ $[M]^+$: 636.1509, Found 636.1492.

(2m)

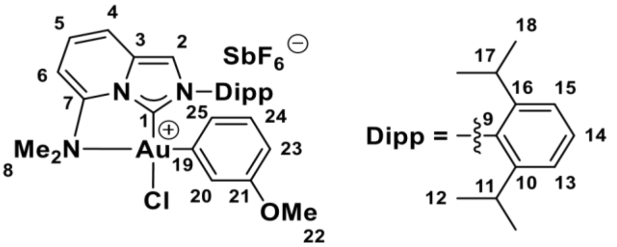

Compound (**2m**) was synthesized following the same procedure as (**2d**), reacting (**1**) (20 mg, 36.1 μmol, 1 eq.) with 3-iodoanisole (85.8 μL, 0.72 mmol, 20 eq.) in the presence of $AgSbF_6$ (12.4 mg, 36.1 μmol, 1 eq.). A light pink solid of the desired product was obtained (9.5 mg, 29%).

^{1}H NMR (500 MHz; CD_2Cl_2) δ: 7.75 (d, *J* = 9.2 Hz, 1H, C4-H or C6-H), 7.57 (s, 1H, C2-H), 7.50 (dd, *J* = 9.2, 7.3 Hz, 1H, C5-H), 7.36 (d, *J* = 7.3 Hz, 1H, C4-H or C6-H), 7.26 (t, *J* = 7.9 Hz, 1H, C14-H), 7.02 (d, *J* = 7.9 Hz, 1H, C13-H or C15-H) 6.99 (d, *J* = 7.9 Hz, 1H, C13-H or C15-H), 6.68 (app t, *J* = 8.1 Hz, 1H, C24-H), 6.43-6.38 (m, 2H, C23-H and C25-H) 6.37-6.35 (m, 1H, C20-H), 3.67 (s, 3H, C22-H), 3.53 (s, 6H, C8-H_3), 2.19 (hept, *J* = 6.8 Hz, 2H, C11-H and C17-H), 1.26 (d, *J* = 6.8 Hz, 6H, C12-H_3 or C18-H_3), 0.99 (d, *J* = 6.8 Hz, 6H, C12-H_3 or C18-H_3). **^{13}C{^{1}H} NMR** (126 MHz; CD_2Cl_2) δ: 159.7 (C21), 144.5 (C10 and C16), 143.9 (C7), 141.5 (C1), 133.1 (C9), 132.1 (C14), 131.9 (C3), 130.2 (C24), 128.7 (C19), 127.9 (C5), 124.8 (C13 or C15), 124.6 (C13 or C15), 123.9 (C23 or C25), 119.7 (C2), 119.2 (C4 or C6), 118.3 (C20), 112.2 (C23 or C25), 111.6 (C4 or C6), 55.8 (C22), 51.5 (C8), 29.2 (app d, C11 and C17), 26.7 (C12 or C18), 22.3 (app d, C12 or C18).

HRMS: $(ESI)^+$ Calculated for $[C_{28}H_{34}AuClN_3O]^+$ $[M]^+$: 660.2056, Found 660.2058.

(2n)

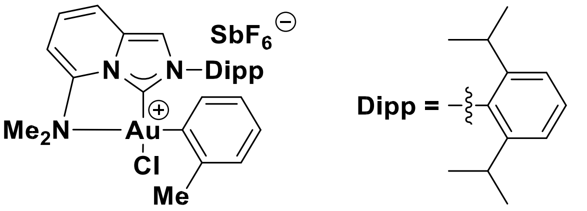

Attempted synthesis of compound (**2n**) occurred following the same procedure as (**2d**), reacting (**1**) (20 mg, 36.1 μmol, 1 eq.) with 2-iodothiophene (79.5 μL, 0.72 mmol, 20 eq.) in the presence of $AgSbF_6$

(12.4 mg, 36.1 µmol, 1 eq.). Only trace quantities of the product were observed, as supported by HRMS analysis.

HRMS: (ESI)$^+$ Calculated for $[C_{28}H_{34}AuClN_3]^+$ $[M]^+$: 644.2109, Found 644.2107.

(2o)

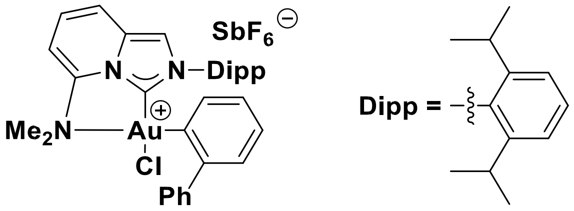

Attempted synthesis of compound (**2o**) occurred following the same procedure as (**2d**), reacting (**1**) (20 mg, 36.1 µmol, 1 eq.) with 2-iodothiophene (79.5 µL, 0.72 mmol, 20 eq.) in the presence of $AgSbF_6$ (12.4 mg, 36.1 µmol, 1 eq.). Only trace quantities of the product were observed, as supported by HRMS analysis.

HRMS: (ESI)$^+$ Calculated for $[C_{33}H_{36}AuClN_3]^+$ $[M]^+$: 706.2253, Found 706.2263.

3.3. Oxidative addition of biphenylene

(2p)

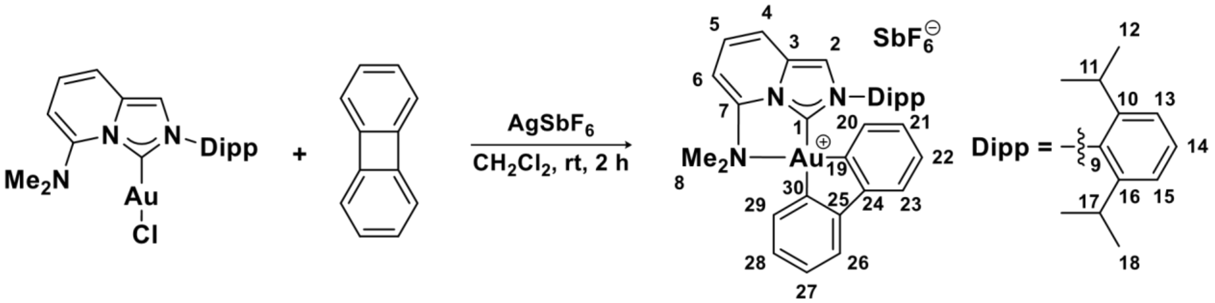

(**1**) (20.0 mg, 36.1 µmol, 1 eq.) and biphenylene (55 mg, 0.72 mmol, 20 eq.) were dissolved in 2.5 mL dry CH_2Cl_2. Separately $AgSbF_6$ (12.4 mg, 36.1 µmol, 1 eq.) was dissolved in dry CH_2Cl_2 (2.5 mL) in the absence of light. The solution containing gold was then added to the silver solution and stirred at rt for 2 hours. Afterwards, the solution was filtered through a syringe sinter frit, the volume decreased under reduced pressure, and precipitated with 20 mL of Et_2O. The solution was decanted, the precipitate washed with 2 × 20 mL Et_2O and dried under reduced pressure. An off-white solid of the desired product **(2p)** was isolated (12.0 mg, 37%).

^{1}H NMR (500 MHz; CD_2Cl_2) δ: 7.70 (d, *J* = 9.1 Hz, 1H, C6-H), 7.66–7.59 (m, 2H, C2-H and C14H), 7.56 (d, *J* = 7.3 Hz, 1H), 7.47 (dd, *J* = 9.1, 7.2 Hz, 1H, C5H), 7.42 (d, *J* = 6.9 Hz, 1H), 7.40–7.33 (m, 3H), 7.30-7.17 (m, 3H), 6.95 (t, *J* = 6.4 Hz, 1H), 6.36 (d, *J* = 7.4 Hz, 1H), 6.02 (t, *J* = 7.1 Hz, 1H), 3.73

(s, 3H, C8-H), 2.41 (hept, J = 6.9 Hz, 2H, C17-H and C11-H), 1.14 (d, J = 6.9 Hz, 6H), 1.09 (d, J = 6.9 Hz, 6H). **^{13}C{^{1}H} NMR** (126 MHz; CD_2Cl_2) δ: 175.1 (C1), 162.2, 154.0, 152.7, 149.0, 147.2 (C7), 145.4 (C10 and C16), 134.7, 134.6 (C9), 132.5, 132.4 (C3), 130.5, 129.5, 128.8, 127.5, 127.4 (C5), 126.7, 125.8 (C13 and C15), 122.9, 122.7, 119.3, 118.7 (C6), 110.3 (C4), 52.6 (C8), 29.2, 25.7, 23.3.

HRMS: $(ESI)^+$ Calculated for $[C_{33}H_{35}N_3Au]^+$ $[M]^+$: 670.2496, Found 670.2486.

4. Oxyarylation catalysis

4.1. *Reaction optimization*

General Optimization Procedure

A Youngs tube was charged with the desired base and silver salt and dissolved in the solvent. Separately **1** (0.05 eq.), iodobenzene (22.4 µL, 0.2 mmol, 1 eq.) and ^{n}BuOH (183.0 µL, 2.0 mmol, 10 eq.) were dissolved in the corresponding solvent. Under a flow of ethylene, the gold solution was added to the Youngs tube at rt and the vessel was pressurized to 1 bar. The reaction was evaluated over various times and temperatures.

(2-butoxyethyl)benzene (5a)

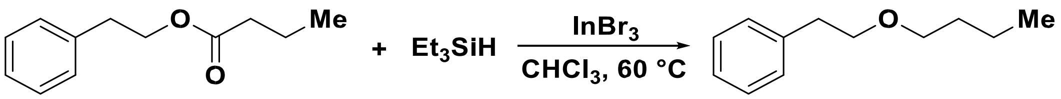

An authentic sample of (2-butoxyethyl)benzene was required for gas-chromatography calibrations. The compound was synthesized using literature precedent.[2] Under a N_2 atmosphere, $InBr_3$ (31.8 mg, 0.09 mmol, 5 mol%) was dissolved in distilled $CHCl_3$ (1.8 mL). Subsequently, phenylethylbutyrate (0.34 mL, 1.8 mmol, 1 eq.) and triethylsilane (1.14 mL, 7.2 mmol, 4 eq.) were added and the vessel was sealed. The reaction mixture was heated at 60 °C for approx. 4 hours. After this time, the mixture was quenched with H_2O (27 mL). The aqueous layer was extracted with $CHCl_3$ (45 mL) a the resulting organic phase was dried with Na_2SO_4 and dried *in vacuo*. Following this, the crude product was purified using flash column chromatography (1% EtOAc/ *n*-hexane) to afford desired compound (150 mg, 47%) as a colorless oil.

^{1}H NMR (400 MHz; $CDCl_3$) δ: 7.32 – 7.26 (m, 2H, Ar–H), 7.25 – 7.18 (m, 3H, Ar–H), 3.63 (t, *J* = 7.3 Hz, 2H, OCH$_2$), 3.44 (t, *J* = 6.6 Hz, 2H, OCH$_2$), 2.89 (t, *J* = 7.3 Hz, 2H, CH$_2$), 1.60 – 1.55 (m, 2H, CH$_2$), 1.41 – 1.31 (m, 2H, CH$_2$), 0.91 (t, *J* = 7.3 Hz, 3H, CH$_3$). **^{13}C{^{1}H} NMR** (101 MHz; $CDCl_3$) δ: 139.3 (Ar–C), 129.1 (Ar–CH), 128.5 (Ar–CH), 126.3 (Ar–CH), 72.0 (OCH$_2$), 70.9 (OCH$_2$), 36.6 (CH$_2$), 32.0 (CH$_2$), 19.5 (CH$_2$), 14.1 (CH$_3$). The spectroscopic data is consistent with literature precedent.[9]

Equivalents of ^{n}BuOH

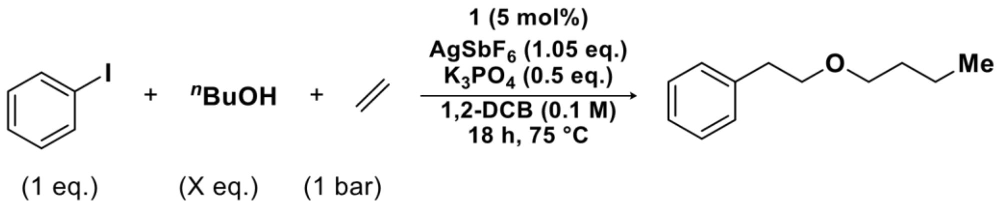

^{n}BuOH	Yield^a
1 eq.	27%
2 eq.	33%
4 eq.	60%
6 eq.	57%
8 eq.	61%
10 eq.	66%

^{a}Yield determined by GCMS using dodecane as internal standard.

Concentration

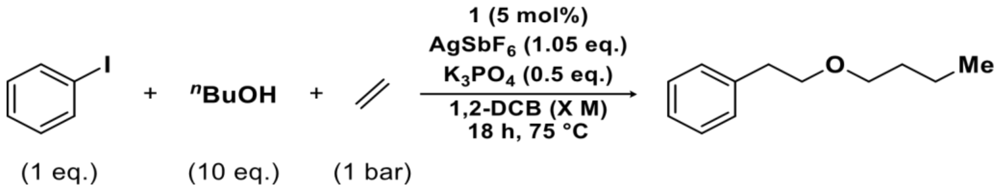

Concentration	Yield^a
0.1 M	66%
0.2 M	68%
0.4 M	62%
0.6 M	59%
0.8 M	50%

^{a}Yield determined by GCMS using dodecane as internal standard.

Solvent Screen

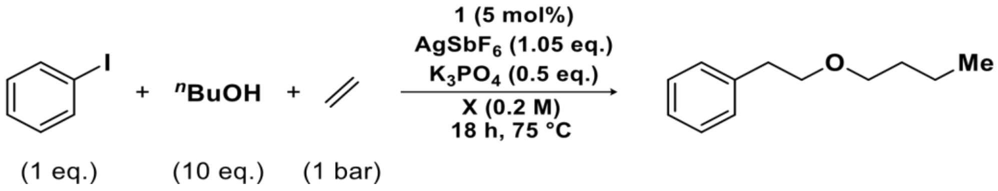

Solvent	Yield[a]
1,2-DCB	68%
Chlorobenzene*	56%
DCE	36%
^{n}BuOH	3%
1,2-DCB: ^{n}BuOH (1:1)	19%
THF	0%
Dioxane	0%
Chloroform	30%
MeCN	0%
PhCN	0%
DMF	0%
TCE	30%
TBME	11%

[a]Yield determined by GCMS using dodecane as internal standard.

*1.2 eq. $AgSbF_6$ and $NaHCO_3$ (1 eq.) used

Silver salt screen

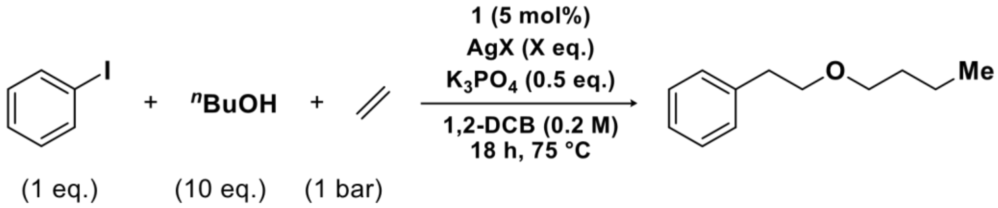

Silver salt	Equivalents	Yield[a]
N/a	N/a	0%
$AgSbF_6$	1.05 eq	68%
$AgSbF_6$	1.1 eq	75%
$AgSbF_6$	1.2 eq	78%
AgOTf	1.05 eq	0%
$AgNTf_2$	1.05 eq	0%

[a]Yield determined by GCMS using dodecane as internal standard.

Base screen

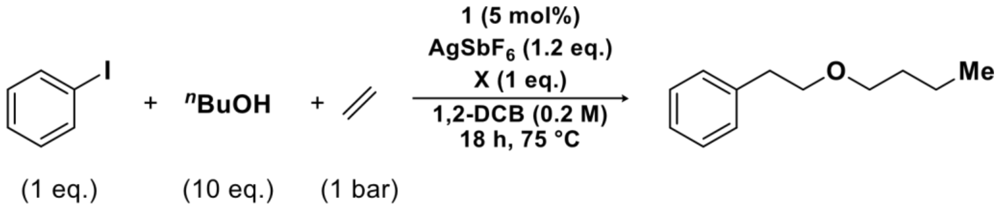

Base	Yield[α]
K_3PO_4 (0.5 eq)	77%
K_3PO_4 (1 eq)	71%
$NaHCO_3$	88%
K_2CO_3	87%
Cs_2CO_3	0%
Li_2CO_3	75%
NaOAc	0%
LiOAc	81%
KO*t*Bu	1%
KHMDS	10%
NEt_3	0%
Proton sponge®	0%
DABCO	0%

[α]Yield determined by GCMS using dodecane as internal standard.

Control reactions

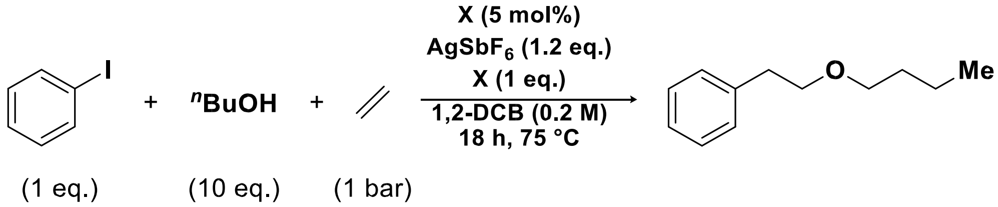

Control	Yield[α]
**1**	90%
No gold	0%
$Pd(PPh_3)_4$	0%
IPrAuCl	0%
MeDalphosAuCl	32%
**2b**	70%

[α]Yield determined by ^1H NMR using trimethoxybenzene as internal standard.

Mercury drop-test and Filtration

Control[α]	Yield[β,γ]
Standard	80%
Hg	80%
Filtration	80%

[α]Reaction vessel depressurized after 10 hours, control conducted then repressurized with 1 bar of ethylene and reacted for a further 8 hours. [β] Yield determined by ^{1}H NMR using trimethoxybenzene as internal standard. [γ] Yield of standard reaction after 10 hours is 21%.

4.2. Oxyarylation of ethylene

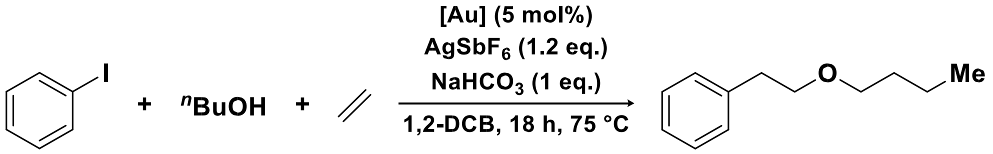

General procedure 1

A Youngs tube was charged with $NaHCO_3$ (1 eq.) and $AgSbF_6$ (1.2 eq.), which were then dissolved in 1,2-dichlorobenzene (0.5 mL). Separately [Au] (0.05 eq.), aryl iodide (1 eq.) and the corresponding alcohol (10 eq.) were dissolved in 1,2-dichlorobenzene (0.5 mL). Under a flow of ethylene, the Au solution was added to the Youngs tube at rt and the vessel was pressurized to 1 bar. The reaction was heated to 75 °C for 18 hours. The reaction mixture was then cooled to rt and the mixture was purified by flash column chromatography under the specified conditions.

General procedure 2 (Solid alcohols)

A Youngs tube was charged with $NaHCO_3$ (1 eq.) $AgSbF_6$ (1.2 eq.) and the chosen alcohol (10 eq.), which were then dissolved in 1,2-dichlorobenzene (0.5 mL). Separately [Au] (0.05 eq.) and the aryl-iodide (1 eq.) were dissolved in 1,2-dichlorobenzene (0.5 mL). Under a flow of ethylene, the Au solution was added to the Youngs tube at rt and the vessel was pressurized to 1 bar. The reaction was heated to 75 °C for 18 hours. The reaction mixture was then cooled to rt and the mixture was purified by flash column chromatography under the specified conditions.

Oxyarylation products

Alcohol scope

(2-Butoxyethyl)benzene (5a)

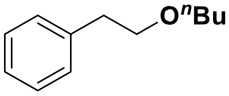

Iodobenzene (22.4 µL, 0.2 mmol, 1 eq.) and ^{n}BuOH (183.0 µL, 2.0 mmol, 10 eq.) were reacted using general procedure 1, resulting in the title compound (27.5 mg, 85%) as a colorless oil (eluent: pentane to 1.5% Et_2O/pentane).

^{1}H NMR (400 MHz; $CDCl_3$) δ: 7.32 – 7.26 (m, 2H, Ar–H), 7.25 – 7.18 (m, 3H, Ar–H), 3.63 (t, *J* = 7.3 Hz, 2H, OCH_2), 3.45 (t, *J* = 6.6 Hz, 2H, OCH_2), 2.90 (t, *J* = 7.3 Hz, 2H, CH_2), 1.60 – 1.51 (m, 2H, CH_2), 1.41 – 1.31 (m, 2H, CH_2), 0.91 (t, *J* = 7.3 Hz, 3H, CH_3). **^{13}C{^{1}H} NMR** (101 MHz; $CDCl_3$) δ: 139.3 (Ar–C), 129.0 (Ar–CH), 128.5 (Ar–CH), 126.3 (Ar–CH), 72.0 (OCH_2), 70.9 (OCH_2), 36.6 (CH_2), 32.0 (CH_2), 19.5 (CH_2), 14.1 (CH_3). The spectroscopic data is consistent with that of literature precedent.[2]

(2-Ethoxyethyl)benzene (5b)

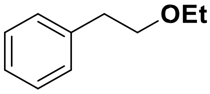

Iodobenzene (22.4 µL, 0.2 mmol, 1 eq.) and 1-propanol (149.5 µL, 2.0 mmol, 10 eq.) were reacted using general procedure 1, giving **5b** (23.0 mg, 84%) as a yellow oil (eluent: pentane to 1% Et_2O/pentane).

^{1}H NMR (400 MHz; $CDCl_3$) δ: 7.31 – 7.27 (m, 2H, Ar–H), 7.24 – 7.18 (m, 3H, Ar–H), 3.64 (t, *J* = 7.4 Hz, 2H, OCH_2), 3.51 (q, *J* = 7.0 Hz, 2H, OCH_2), 2.90 (t, *J* = 7.4 Hz, 2H, CH_2), 1.21 (t, *J* = 7.0 Hz, 3H, CH_3). **^{13}C{^{1}H} NMR** (101 MHz; $CDCl_3$) δ: 138.2, 129.0, 128.5, 126.3, 71.8, 66.4, 36.6, 15.3. The spectroscopic data is consistent with that of literature precedent.[10]

(2-Propoxyethyl)benzene (5c)

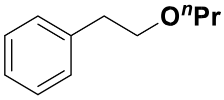

Iodobenzene (22.4 µL, 0.2 mmol, 1 eq.) and ^{n}PrOH (116.8 µL, 2.0 mmol, 10 eq.) were reacted using general procedure 1, giving **5c** (25.6 mg, 86%) as a pale yellow oil (eluent: pentane to 2.5% Et_2O/pentane).

^{1}H NMR (400 MHz; $CDCl_3$) δ: 7.32 – 7.27 (m, 2H, Ar–H), 7.25 – 7.18 (m, 3H, Ar –H), 3.63 (t, *J* = 7.3 Hz, 2H, OCH_2), 3.41 (t, *J* = 6.7 Hz, 2H, OCH_2), 2.90 (t, *J* = 7.4 Hz, 2H, CH_2), 1.60 (h, *J* = 7.3 Hz, 2H, CH_2), 0.91 (t, *J* = 7.4 Hz, 3H, CH_3). **^{13}C{^{1}H} NMR** (101 MHz; $CDCl_3$) δ: 139.2 (Ar–C), 129.1 (Ar–CH),

128.4 (Ar–CH), 126.3 (Ar–CH), 72.8, (OCH$_2$) 71.9 (OCH$_2$), 36.5 (CH$_2$), 23.1 (CH$_2$), 10.7 (CH$_3$). The spectroscopic data is consistent with that of literature precedent.[11]

(2-Pentoxyethyl)benzene (5d)

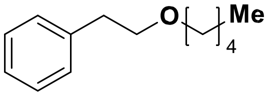

Iodobenzene (22.4 µL, 0.2 mmol, 1 eq.) and pentanol (217.4 µL, 2.0 mmol, 10 eq.) were reacted using general procedure 1, resulting in the title compound (22.2 mg, 63%) as a colorless oil (eluent: pentane to 1.5% Et$_2$O/pentane).

^{1}H NMR (400 MHz; CDCl$_3$) δ: 7.32 – 7.27 (m, 2H, Ar–H), 7.25 – 7.19 (m, 3H, Ar –H), 3.64 (t, *J* = 7.3 Hz, 2H, OCH$_2$), 3.44 (t, *J* = 6.7 Hz, 2H, OCH$_2$), 2.90 (t, *J* = 7.3 Hz, 2H, CH$_2$), 1.59 (m, 2H, CH$_2$), 1.32 (m, 4H, CH$_2$), 0.91 (t, *J* = 7.0 Hz, 3H, CH$_3$). **^{13}C{^{1}H} NMR** (101 MHz; CDCl$_3$) δ: 139.2 (Ar–C), 129.0 (Ar–CH), 128.4 (Ar–CH), 126.3(Ar–CH), 72.0, (OCH$_2$) 71.2 (OCH$_2$), 36.5 (CH$_2$), 29.6 (CH$_2$), 28.5 (CH$_2$), 22.7 (CH$_2$), 14.2 (CH$_3$). The spectroscopic data is consistent with that of literature precedent.[11]

(2-Methoxyethyl)benzene (5e)

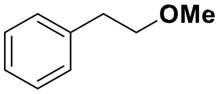

Iodobenzene (22.4 µL, 0.2 mmol, 1 eq.) and MeOH (81.0 µL, 2.0 mmol, 10 eq.) were reacted using a similar method to general procedure 1, resulting in the title compound (11.7 mg, 43%) as a pale yellow oil (eluent: n-hexane to 5% EtOAc/n-hexane).

^{1}H NMR (400 MHz; CDCl$_3$) δ: 7.32 – 7.28 (m, 2H, Ar–H), 7.24 – 7.19 (m, 3H, Ar–H), 3.61 (t, *J* = 7.1 Hz, 2H, CH$_2$), 3.37 (s, 3H, OCH$_3$), 2.89 (t, *J* = 7.1 Hz, 2H, CH$_2$). **^{13}C{^{1}H} NMR** (101 MHz; CDCl$_3$) δ: 139.0 (Ar–C), 128.9 (Ar–CH), 128.5 (Ar–CH), 126.3 (Ar–CH), 73.7 (CH$_2$), 58.8 (OCH$_3$), 36.3 (CH$_2$). The spectroscopic data is consistent with that of literature precedent.[12]

(2-Octoxyethyl)benzene (5f)

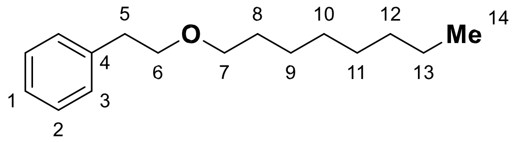

Iodobenzene (22.4 µL, 0.2 mmol, 1 eq.) and octanol (314.9 µL, 2.0 mmol, 10 eq.) were reacted using general procedure 1, resulting in the title compound (20.1 mg, 46%) as a colorless oil (eluent: pentane to 1.5% Et$_2$O/pentane).

^{1}H NMR (400 MHz; CDCl$_3$) δ: 7.32 – 7.27 (m, 2H, C2–H), 7.25 – 7.18 (m, 3H, C1–H and C3–H), 3.63 (t, *J* = 7.3 Hz, 2H, C6–H), 3.44 (t, *J* = 6.7 Hz, 2H, C7–H), 2.90 (t, *J* = 7.3 Hz, 2H, C5–H), 1.62 – 1.55

(m, 2H, C8–H), 1.36 – 1.25 (m, 10H, C9–H, C10–H, C11–H, C12–H, C13–H), 0.90 (t, J = 6.8 Hz, 3H, C14–H). **^{13}C{^{1}H} NMR** (101 MHz; $CDCl_3$) δ: 139.3 (C4), 129.0 (C3), 128.4 (C2), 126.3 (C1), 71.9 (C6), 71.3 (C7), 36.5 (C5), 32.0 (C8), 29.9 (CH_2), 29.6 (CH_2), 29.4 (CH_2), 26.3 (CH_2), 22.8 (CH_2), 14.2 (C14).

HRMS (EI)$^+$: Calculated for $[C_{16}H_{26}O]^+$ $[M]^+$: 234.1978. Found 234.1979.

(2-Octododecoxyethyl)benzene (5g)

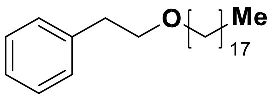

Iodobenzene (22.4 µL, 0.2 mmol, 1 eq.) and 1-octadecanol (541.0 mg, 2.0 mmol, 10 eq.) were reacted using general procedure 2, resulting in the title compound (41.1 mg, 55%) as a colorless solid (eluent: pentane to 20% MePh/pentane).

^{1}H NMR (400 MHz; $CDCl_3$) δ: 7.31 – 7.18 (m, 5H, Ar – H), 3.62, (t, J = 7.3 Hz, 2H, OCH_2), 3.43 (t, J = 6.7 Hz, 2H, OCH_2), 2.89 (t, J = 7.3 Hz, 2H, OCH_2), 1.61 – 1.52 (m, 2H, OCCH_2), 1.26 (s, 30H, 15 × CH_2). **^{13}C{^{1}H} NMR** (101 MHz; $CDCl_3$) δ: 139.3 (Ar – C), 129.1 (Ar – CH), 128.5 (Ar – CH), 126.3 (Ar – CH), 72.0 (OCH_2), 71.3 (OCH_2), 36.6 (CH_2), 32.1 (CH_2), 29.9 – 29.8 (5 × CH_2), 29.6 (CH_2), 29.5 (CH_2), 26.3 (CH_2), 22.9 (CH_2), 14.3 (CH_3). The spectroscopic data is consistent with that of literature precedent.[11]

(2-(2-Ethylhexoxy)ethyl)benzene(5h)

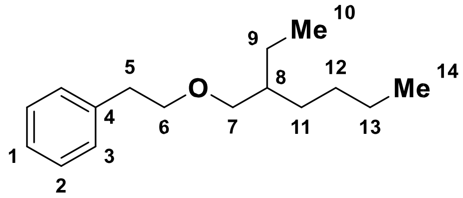

Iodobenzene (22.4 µL, 0.2 mmol, 1 eq.) and 2-ethyl-1-hexanol (312.7 µL, 2.0 mmol, 10 eq.) were reacted using general procedure 1, resulting in the title compound (28.3 mg, 65%) as a colorless oil (eluent: pentane to 2% Et_2O/pentane).

^{1}H NMR (400 MHz; $CDCl_3$) δ: 7.31 – 7.17 (m, 5H, C1 – H, C2 – H and C3 – H), 3.61 (t, J = 7.2 Hz, 2H, C6 – H_2), 3.31 (d, J = 6.0 Hz, 2H, C7 – H_2), 2.88 (t, J = 7.2 Hz, 2H, C5 – H_2) 1.54 – 1.46 (m, 1H, C8 – H), 1.40 – 1.19 (m, 8H, C9 – H_2, C11 – H_2, C12 – H_2 and C13 – H_2). 0.92 – 0.82 (m, 6H, C10 – H_3 and C14 – H_3). **^{13}C{^{1}H} NMR** (101 MHz; $CDCl_3$) δ: 139.5 (C4), 129.1 (C2 or C3), 128.4 (C2 or C3), 126.2 (C1), 74.0 (C7), 72.2 (C6), 39.8 (C8), 36.5 (C5), 30.7 (CH_2), 29.2 (CH_2), 24.0 (CH_2), 23.2 (CH_2), 14.3 (C10 or C14), 11.2 (C10 or C14).

HRMS (EI^+): Calculated or $[C_{16}H_{26}O]^+[M]^+$: 234.1978. Found 234.1979.

(2-Isopropoxyethyl)benzene (5i)

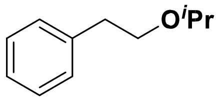

Iodobenzene (22.4 µL, 0.2 mmol, 1 eq.) and ^{i}PrOH (153.1 µL, 2.0 mmol, 10 eq.) were reacted using general procedure 1, resulting in the title compound (17.2 mg, 57%) as a yellow oil (eluent: pentane to 2.5% Et_2O/pentane).

^{1}H NMR (400 MHz; $CDCl_3$) δ: 7.31 – 7.26 (m, 2H, Ar–H), 7.25 – 7.18 (m, 2H, Ar–H), 3.64 – 3.54 (m, 3H, CH_2 and CH(CH_3)$_2$), 2.88 (t, J = 7.5 Hz, 2H, CH_2), 1.15 (d, J = 6.1 Hz, 6H, CH(CH_3)$_2$). **^{13}C{^{1}H} NMR** (101 MHz; $CDCl_3$) δ: 139.3 (Ar–C), 129.1 (Ar–CH), 128.4 (Ar-CH), 126.3 (Ar-CH), 71.7 (CH_2), 69.4 (CH(CH_3)$_2$), 37.0 (CH_2), 22.3 (CH(CH_3)$_2$. The spectroscopic data is consistent with that of literature precedent.[13]

(2-Cyclopentoxyethyl)benzene (5j)

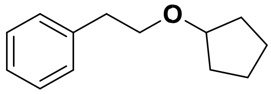

Iodobenzene (22.4 µL, 0.2 mmol, 1 eq.) and cyclopentanol (181.7 µL, 2.0 mmol, 10 eq.) were reacted using general procedure 1, resulting in the title compound (31.9 mg, 92%) as a colorless oil (eluent: pentane to 1.5% Et_2O/pentane).

^{1}H NMR (400 MHz; $CDCl_3$) δ: 7.32 – 7.18 (m, 5H, Ar – H), 3.92 (m, 1H, CH), 3.60 (t, J = 7.5 Hz, 2H, OCH_2), 2.88 (t, J = 7.5 Hz, 2H, Ar – CH_2), 1.80 – 1.46 (m, 8H, 4 × cyclopentyl – CH_2). **^{13}C{^{1}H} NMR** (101 MHz; $CDCl_3$) δ: 139.4 (Ar – C), 129.1 (Ar – CH), 128.4 (Ar – CH), 126.2 (Ar – CH), 81.6 (cyclopentyl – CH), 70.0 (OCH_2), 36.9 (CH_2), 32.4 (cyclopentyl – CH_2), 23.7 (cyclopentyl – CH_2). The spectroscopic data is consistent with that of literature precedent.[14]

***O*-(2-Phenylethyl)menthol (5k)**

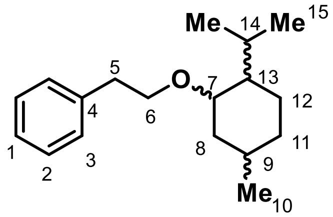

Iodobenzene (22.4 µL, 0.2 mmol, 1 eq.) and (±)-menthol (312.5 mg, 2.0 mmol, 10 eq.) were reacted using general procedure 2, resulting in the title compound (39.3 mg, 75%) as a colorless oil (eluent: pentane to 3% Et_2O/pentane).

^{1}H NMR (400 MHz; $CDCl_3$) δ: 7.32 – 7.16 (m, 5H, Ar – H), 3.89 – 3.81 (m, 1H, C6 – H_2), 3.53 – 3.44 (m, 1H, C6 – H_2), 3.03 (td, J = 10.6, 4.1 Hz, 1H, C7 – H), 2.88 (t, J = 7.5 Hz, C5 – H_2), 2.21 – 2.05 (m, 2H, C8 – H_2 and C9 – H), 1.69 – 1.55 (m, 2H, C11 – H_2 and C12 – H_2) 1.41 – 1.26 (m, 1H, C14 – H), 1.22 (ddt, J = 12.2, 10.3, 3.1 Hz, 1H, C13 – H) 1.03 – 0.77 (m, 9H, C8 – H, C11 – H, C12 – H, C15 – H_3 and C16 – H_3), 0.69 (d, J = 6.9 Hz, 3H, C10 – H). **^{13}C{^{1}H} NMR** (101 MHz; $CDCl_3$) δ: 139.4 (C4), 129.0 (C2 or C3), 128.4 (Ar – CH), 126.2 (Ar – CH), 79.7 (C7), 69.8 (C6), 48.4 (C13), 40.7 (C8), 37.1 (C5), 34.7 (C11), 25.7 (C9), 23.5 (C12), 22.5 (C15 or C16), 21.1 (C15 or C16), 16.3 (C10). The spectroscopic data is consistent with that of literature precedent.[11]

(2-(2-Methoxyethoxy)ethyl)benzene (5l)

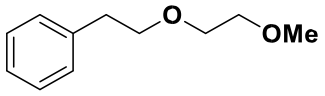

Iodobenzene (22.4 µL, 0.2 mmol, 1 eq.) and 2-methoxyethanol (157.7 µL, 2.0 mmol, 10 eq.) were reacted using general procedure 1, resulting in the title compound (16.2 mg, 49%) as a colorless oil (eluent: pentane to 20% Et_2O/pentane).

^{1}H NMR (400 MHz; $CDCl_3$) δ: 7.33 – 7.18 (m, 5H, Ar – H), 3.70 (t, J = 7.6 Hz, 2H, OCH_2) 3.63 – 3.59 (m, 2H, OCH_2), 3.58 – 3.53 (m, 2H, $MeOCH_2$), 3.40 (s, 3H, OCH_3) 2.94 (t, 7.5 Hz, 2H, CH_2) **^{13}C{^{1}H} NMR** (101 MHz; $CDCl_3$) δ: 139.0, (Ar–C), 129.1 (Ar–CH), 128.5 (Ar–CH), 126.3 (Ar–CH), 72.6 OCH_2), 72.1 (CH_2OMe), 70.3 (OCH_2), 59.2 (OCH_3), 36.4 (CH_2). The spectroscopic data is consistent with literature precedent.[11]

(2-Butoxyethyl)toluene (5m)

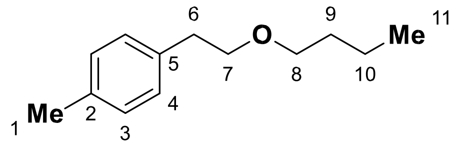

4-Iodotoluene (43.8 mg, 0.2 mmol, 1 eq.) and ^{n}BuOH (183.0 µL, 2.0 mmol, 10 eq.) were reacted using general procedure 1, resulting in the title compound (30.4 mg, 86%) as a yellow oil (eluent: pentane to 1.5% Et_2O/pentane).

^{1}H NMR (400 MHz; $CDCl_3$) δ: 7.15 – 7.07 (m, 4H, C3 – H and C4 – H), 3.60 (t, J = 7.4 Hz, 2H, C7– H_2), 3.44 (t, J = 6.7 Hz, 2H, C8 – H_2), 2.85 (t, J = 7.4 Hz, 2H, C6 – H_2), 2.32 (s, 3H. C1 – H_3), 1.59 – 1.53 (m, 2H, C9 – H_2 or C10 – H_2), 1.41 – 1.32 (m, 2H, C9 – H_2 or C10 – H_2), 0.92 (t, J = 7.4 Hz, 3H,

C11 – H$_3$). **^{13}C{^{1}H} NMR** (101 MHz; CDCl$_3$) δ: 136.0 (C5), 135.7 (C2), 129.1 (C3 or C4), 128.9 (C3 or C4), 72.1 (C7), 70.9 (C8), 36.0 (C6), 31.9 (C9), 21.1 (C1), 19.4 (C10), 14.0 (C11).

HRMS (EI$^+$): Calculated for $[C_{13}H_{20}O]^+$ $[M]^+$: 192.1509. Found 192.1508.

(2-Butoxyethyl)-4-methoxybenzene (5n)

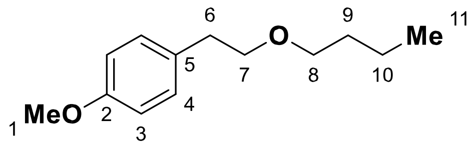

4-Iodoanisole (47.1 mg, 0.2 mmol, 1 eq.) and ^{n}BuOH (183.0 µL, 2.0 mmol, 10 eq.) were reacted using general procedure 1, resulting in the title compound (30.4 mg, 70%) as a yellow oil (eluent: pentane to 1.5% Et$_2$O/pentane).

^{1}H NMR (400 MHz; CDCl$_3$) δ: 7.17 – 7.11 (m, 2H, C4 – H), 6.86 – 6.80 (m, 2H, C3 – H), 3.79 (s, 3H, C1– H$_3$), 3.58 (t, J = 7.4 Hz, 2H, C7 – H$_2$), 3.43, (t, J = 6.9 Hz, 2H, C8 – H$_2$), 2.83 (t, J = 7.4 Hz, 2H, C6 – H$_2$), 1.60 – 1.52 (m, 2H, C9 – H$_2$ or C10 – H$_2$), 1.42 – 1.31 (m, 2H, C9 – H$_2$ or C10 – H$_2$), 0.91 (t, J = 7.4 Hz, 3H, C11 – H$_3$). **^{13}C{^{1}H} NMR** (101 MHz; CDCl$_3$) δ: 158.1 (C2), 131.3 (C5), 129.9 (C4), 113.8 (C3), 72.2 (C7), 70.9 (C8), 55.3 (C1), 35.6 (C6), 31.9 (C9), 19.4, (C10) 14.0 (C11).

HRMS (EI$^+$): Calculated for $[C_{13}H_{20}O_2]^+$ $[M]^+$: 208.1458. Found 208.1458.

(2-Butoxyethyl)-3-methoxybenzene (5o)

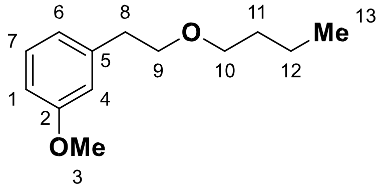

3-Iodoanisole (23.8 µL, 0.2 mmol, 1 eq.) and ^{n}BuOH (183.0 µL, 2.0 mmol, 10 eq.) were reacted using general procedure 1, resulting in the title compound (31.4 mg, 82%) as a colorless oil (eluent: pentane to 2% Et$_2$O/pentane).

^{1}H NMR (400 MHz; CDCl$_3$) δ: 7.20 (app t, J = 7.8 Hz, 1H, C1 – H or C7 – H), 6.84 – 6.73 (m, 3H, (C1 – H or C7 – H), C4 – H and C6 – H), 3.80 (s, 3H, C3 – H$_3$), 3.62 (t, J = 7.3 Hz, 2H, C9 – H$_2$), 3.44 (t, J = 6.7 Hz, 2H, C10 – H$_2$), 2.87 (t, J = 7.3 Hz, 2H, C8 – H$_2$), 1.60 – 1.51 (m, 2H, C11 – H$_2$) 1.42 – 1.31 (m, 2H, C12– H$_2$), 0.91 (t, J = 7.3 Hz, 3H, C13 – H$_3$). **^{13}C{^{1}H} NMR** (101 MHz; CDCl$_3$) δ: 159.7 (C2), 140.9 (C5), 129.4 (C1 or C7), 121.4 (C4 or C6), 114.8 (C4 or C6), 111.7 (C1 or C7), 71.9 (C9), 70.9 (C10), 55.3 (C3), 36.6 (C8), 32.0 (C11), 19.5 (C12), 14.1 (C13).

HRMS (EI^+): Calculated for $[C_{13}H_{20}O_2]^+$ $[M]^+$: 208.1458. Found 208.1458.

2-(2-Butoxyethyl)naphthalene (5p)

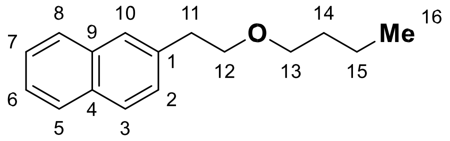

2-Iodonaphthalene (50.8 mg, 0.2 mmol, 1 eq.) and ^{n}BuOH (183.0 μL, 2.0 mmol, 10 eq.) were reacted using general procedure 1, resulting in the title compound (33.6 mg, 80%) as a pale yellow oil (eluent: pentane to 2% Et_2O/pentane).

^{1}H NMR (400 MHz; $CDCl_3$) δ: 7.84 – 7.76 (m, 3H, C2 – H, C3 – H and C5 – H), 7.68 (s, 1H, C10 – H), 7.48 – 7.40 (m, 2H, C6 – H and C7 – H), 7.38 (app dd, J = 8.4, 1.7 Hz, 1H, C8 – H), 3.72 (t, J = 7.3 Hz, 2H, C12 – H_2), 3.47 (t, J = 6.6 Hz, 2H, C13 – H_2), 3.06 (t, J = 7.3 Hz, 2H, C11 – H_2), 1.63 – 1.54 (m, 2H, C14 – H_2), 1.43 – 1.33 (m, 2H, C15 – H_2), 0.92 (t, J = 7.4 Hz, 3H, C16 – H_3). **^{13}C{^{1}H} NMR** (101 MHz; $CDCl_3$) δ: 136.9 (C1), 133.7 (C4), 132.3 (C9), 128.0 (C2, C3, C5 or C8), 127.8 (C2, C3, C5 or C8), 127.7 (C2, C3, C5 or C8), 127.6 (C2, C3, C5 or C8), 127.3 (C10), 126.0 (C6 or C7) 125.4 (C6 or C7), 71.9 (C12), 71.0 (C13), 36.7 (C11), 32.0 (C14), 19.5 (C15), 14.1 (C16).

HRMS (EI^+): Calculated for $[C_{16}H_{20}O_2]^+$ $[M]^+$: 228.1509. Found 228.1507.

(2-Butoxyethyl)biphenyl (5q)

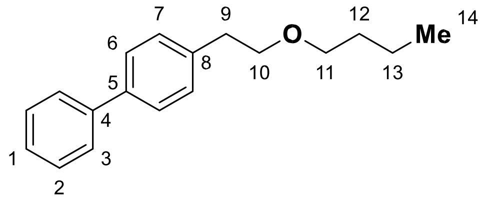

4-Iodobiphenyl (56.0 mg, 0.2 mmol, 1 eq.) and ^{n}BuOH (183.0 μL, 2.0 mmol, 10 eq.) were reacted using general procedure 1, resulting in the title compound (30.4 mg, 61%) as a yellow oil (eluent: pentane to 1.5% Et_2O/pentane).

^{1}H NMR (400 MHz; $CDCl_3$) δ: 7.62 – 7.59 (m, 2H, C3 – H), 7.56 – 7.54 (m, 2H, C6 – H), 7.47 – 7.41 (m, 2H, C2 – H), 7.37 – 7.29 (m, 3H, C1 – H and C7 – H), 3.68 (t, J = 7.2 Hz, 2H, C10 – H_2), 3.48 (t, J = 6.6 Hz, 2H, C11 – H_2), 2.95 (t, J = 7.2 Hz, 2H, C9 – H_2), 1.63 – 1.54 (m, 2H, C12 – H_2), 1.44 – 1.34 (m, 2H, C13 – H_2), 0.94 (t, J = 7.4 Hz, 3H, C14 – H_3). **^{13}C{^{1}H} NMR** (101 MHz; $CDCl_3$) δ: 141.2 (C4 or C5), 139.3 (C4 or C5), 138.4 (C8), 129.5 (C1 or C7), 128.9 (C2), 127.2 ((C1 or C7), C3 and C6), 71.9 (C10), 71.0 (C11), 36.2 (C9), 32.0 (C12), 19.5 (C13), 14.1 (C14).

HRMS (EI$^+$): Calculated for $[C_{18}H_{22}O]^+$ $[M]^+$: 254.1665. Found 254.1665.

(2-Butoxyethyl)-4-hydroxybenzene (5r)

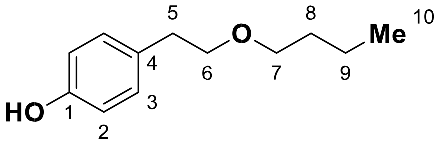

4-Iodophenol (44.0 mg, 0.2 mmol, 1 eq.) and *^{n}*BuOH (183.0 µL, 2.0 mmol, 10 eq.) were reacted using general procedure 1, resulting in the title compound (25.6 mg, 72%) as a pale yellow oil (eluent: pentane to 20% Et_2O/pentane).

^{1}H NMR (400 MHz; $CDCl_3$) δ: 7.10 – 7.04 (m, 2H, C3 – H), 6.77 – 6.70 (m, 2H, C2 – H), 5.40 (br, 1H, OH), 3.61 (t, *J* = 7.3 Hz, 2H, C6 – H_2), 3.46 (t, *J* = 6.7 Hz, C7 – H_2), 2.82 (t, *J* = 7.3 Hz, 2H, C5 – H_2), 1.62 – 1.52 (m, 2H, C8 – H_2), 1.41 – 1.31 (m, 2H, C9 – H_2), 0.91 (t, *J* = 7.4 Hz, C10 – H_3). **^{13}C{^{1}H} NMR** (101 MHz; $CDCl_3$) δ: 154.3 (C1), 131.0 (C4), 130.1 (C3), 115.4 (C2), 72.2 (C6), 71.0 (C7), 35.5 (C5), 31.8 (C8), 19.4 (C9) 14.0 (C10).

HRMS (EI$^+$): Calculated for $[C_{12}H_{18}O_2]^+$ $[M]^+$: 194.1301. Found 194.1301.

(2-Butoxyethyl)-3-bromobenzene (5s)

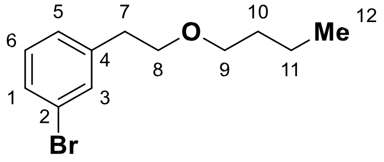

3-Bromoiodobenzene (25.5 µL, 0.2 mmol, 1 eq.) and *^{n}*BuOH (183.0 µL, 2.0 mmol, 10 eq.) were reacted using general procedure 1, resulting in the title compound (27.3 mg, 53%) as a colorless oil (eluent: pentane to 2% Et_2O/pentane).

^{1} H NMR (400 MHz; $CDCl_3$) δ: 7.40 – 7.37 (m, 1H, C3 – H), 7.36 – 7.30 (m, 1H, C6 – H) 7.19 – 7.10 (m, 2H, C1 – H and C5 – H) 3.61 (t, *J* = 7.0 Hz, 2H, C8 – H), 3.43 (t, *J* = 6.6 Hz, 2H, C9 – H), 2.85 (t, *J* = 7.0 Hz, 2H, C7 – H), 0.91 (t, *J* = 7.4 Hz, 3H, C12 – H). **^{13}C{^{1}H} NMR** (101 MHz; $CDCl_3$) δ: 144.8 (C4), 132.1 (C3), 130.0 (C1 or C5), 129.4 (C6), 127.2 (C1 or C5) 122.5 (C2), 71.4 (C8), 71.0 (C9), 36.1 (C7), 31.9 (C10), 19.5 (C11), 14.0 (C12).

HRMS (EI$^+$): Calculated for $[C_{12}H_{17}BrO]^+$ $[M]^+$: 256.0457. Found 256.0457.

(2-Butoxyethyl)-4-fluorobenzene (5t)

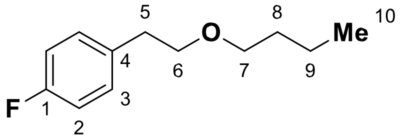

4-Fluoroiodobenzene (23.2 µL, 0.2 mmol, 1 eq.) and ^{n}BuOH (183.0 µL, 2.0 mmol, 10 eq.) were reacted using a modified general procedure 1, with 10 mol% (**1**) used, resulting in the title compound (27.9 mg, 71%) as a pale yellow oil (eluent: pentane to 2% Et_2O/pentane).

^{1}H NMR (400 MHz; $CDCl_3$) δ: 7.21 – 7.14 (m, 2H, C3 – H), 7.00 – 6.93 (m, 2H, C2 – H), 3.59 (t, *J* = 7.1 Hz, 2H, C6 – H), 3.43 (t, *J* = 6.6 Hz, 2H, C7 – H), 2.85 (t, *J* = 7.1 Hz, 2H, C5 – H), 1.58 – 1.50 (m, 2H, C8 – H), 1.40 – 1.29 (m, 2H, C9 – H), 0.91 (t, *J* = 7.4 Hz, 3H, C10 – H). **^{13}C{^{1}H} NMR** (101 MHz; $CDCl_3$) δ: 161.6 (d, *J* = 244.7 Hz, C1), 135.0 (C4), 130.4 (d, *J* = 7.9 Hz, C3), 115.2 (d, *J* = 20.8 Hz, C2), 71.8 (C6), 71.0 (C7), 35.7 (C5), 31.9 (C8), 19.5 (C9), 14.1 (C10). **^{19}F NMR** (376 MHz; $CDCl_3$) δ: -117.3.

HRMS (EI^+): Calculated for $[C_{12}H_{17}FO]^+$ $[M]^+$: 196.1258. Found 196.1258.

(2-Butoxyethyl)-4-bromobenzene (5u)

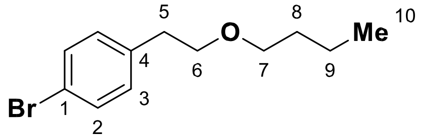

4-Bromoiodobenzene (56.8 mg, 0.2 mmol, 1 eq.) and ^{n}BuOH (183.0 µL, 2.0 mmol, 10 eq.) were reacted using was reacted using a modified general procedure 1, with 10 mol% (**1**) used, resulting in the title compound (14.8 mg, 31%) as a pale yellow oil (eluent: pentane to 2.5% Et_2O/pentane).

^{1}H NMR (400 MHz; $CDCl_3$) δ: 7.42 – 7.37 (m, 2H, C2 – H), 7.13 – 7.07 (m, 2H, C3 – H), 3.59 (t, *J* = 7.0 Hz, 2H, C6 – H), 3.42 (t, *J* = 6.6 Hz, 2H, C7 – H), 2.83 (t, *J* = 7.0 Hz, 2H, C5 – H), 1.54 (m, 2H, C8 – H), 1.36 (m, 2H, C9 – H), 0.90 (t, *J* = 7.4 Hz, 3H, C10 – H). **^{13}C{^{1}H} NMR** (101 MHz; $CDCl_3$) δ: 138.4 (C4), 131.5 (C2), 130.8 (C3), 120.1 (C1), 71.5 (C6), 71.0 (C7), 35.9 (C5), 31.9 (C8), 19.5 (C9), 14.0 (C10).

HRMS (EI^+): Calculated for $[C_{12}H_{17}BrO]^+$ $[M]^+$: 256.0457. Found 256.0457.

4.3. Oxyarylation of propylene

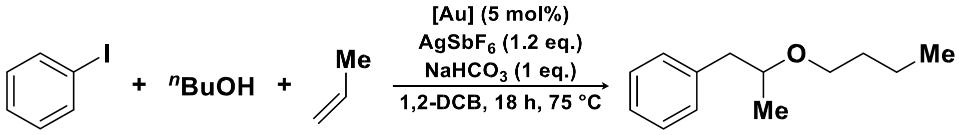

General procedure 3

A Youngs tube was charged with $NaHCO_3$ (1 eq.) and $AgSbF_6$ (1.2 eq.), which were then dissolved in 1,2-dichlorobenzene (0.5 mL). Separately [Au] (0.05 eq.), aryl-iodide (1 eq.) and the corresponding alcohol (10 eq.) were dissolved in 1,2-dichlorobenzene (0.5 mL). Under a flow of propylene, the Au solution was added to the Youngs tube at rt and the vessel was pressurized to 1 bar. The reaction was heated to 75 °C for 18 hours. The reaction mixture was then cooled to rt and purified by flash column chromatography under the conditions noted.

Butyl(1-methyl-2-phenylethyl)ether (6a)

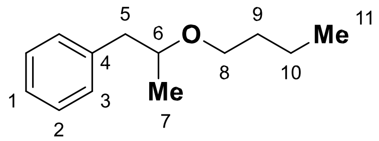

Iodobenzene (22.4 µL, 0.2 mmol, 1 eq.) and ^{n}BuOH (183.0 µL, 2.0 mmol, 10 eq.) were reacted using general procedure 3, resulting in the title compound (30.1 mg, 82%) as a pale yellow oil (eluent: pentane to 1.5% Et_2O/pentane).

^{1}H NMR (400 MHz; $CDCl_3$) δ: 7.30 – 7.23 (m, 2H, C2 – H), 7.22 – 7.15 (m, 3H, C1 – H and C3 – H), 3.57 (app h, J = 6.2 Hz, 1H, C6 – H), 3.51 – 3.41 (m, 1H, C8 – H_2), 3.40 – 3.30 (m, 1H, C8 – H_2), 2.91 (dd, J = 13.4, 6.2 Hz, 1H, C5 – H_2), 2.61 (dd, J = 13.4 Hz, 6.4 Hz, 1H, C5 – H_2), 1.56 – 1.45 (m, 2H, C9 – H), 1.37 – 1.26 (m, 2H, C10 – H), 1.12 (d, J = 6.2 Hz, 3H, C7 – H), 0.87 (t, J = 7.4 Hz, 3H, C11 – H). **^{13}C{^{1}H} NMR** (101 MHz; $CDCl_3$) δ: 139.4 (C4), 129.6 (C1 or C3), 128.3 (C2), 126.1 (C1 or C3), 76.8 (C6), 68.6 (C8), 43.3 (C5), 32.3 (C9), 19.7 (C7), 19.5 (C10), 14.0 (C11).

HRMS (APCI$^+$): Calculated for $[C_{14}H_{23}O]^+$ $[M–H]^+$: 207.1743. Found 207.1742.

Ethyl(1-methyl-2-phenylethyl)ether (6b)

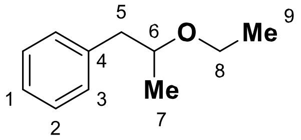

Iodobenzene (22.4 µL, 0.2 mmol, 1 eq.) and EtOH (116.8 µL, 2.0 mmol, 10 eq.) were reacted using general procedure 3, resulting in the title compound (22.6 mg, 76%) as a pale yellow oil (eluent: pentane to 2% Et_2O/pentane).

^{1}H NMR (400 MHz; $CDCl_3$) δ: 7.31 – 7.24 (m, 2H, C2 – H), 7.22 – 7.16 (m, 3H, C1 – H and C3 – H), 3.60 (app h, *J* = 6.2 Hz, 1H, C6 – H), 3.57 – 3.49 (m, 1H, C8 – H_2), 3.48 – 3.38 (m, 1H, C8 – H_2), 2.92 (dd, *J* = 13.50, 6.0 Hz, 1H, C5 – H_2), 2.61 (dd, *J* = 13.50, 6.8 Hz, 1H, C5 – H_2), 1.16 (t, *J* = 7.0 Hz, 3H, C9 – H_3), 1.12 (d, *J* = 6.2 Hz, 3H, C9 – H_3). **^{13}C{^{1}H} NMR** (101 MHz; $CDCl_3$) δ: 139.3 (C4), 129.6 (C1 or C3), 128.3 (C2), 126.1 (C1 or C3), 76.6 (C6), 64.1 (C8), 43.2 (C5), 19.8 (C7), 15.7 (C9).

HRMS (APCI$^+$) : Calculated for $[C_9H_{11}]^+$ $[M+H-EtOH]^+$: 119.0855. Found: 119.0852.

***n*-Propyl(1-methyl-2-phenylethyl)ether (6c)**

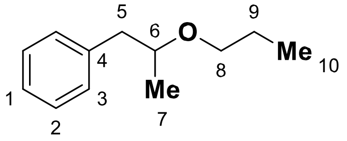

Iodobenzene (22.4 µL, 0.2 mmol, 1 eq.) and ^{n}PrOH (149.5 µL, 2.0 mmol, 10 eq.) were reacted using general procedure 3, resulting in the title compound (23.2 mg, 71%) as a colorless oil (eluent: pentane to 2.5% Et_2O/pentane).

^{1}H NMR (400 MHz; $CDCl_3$) δ: 7.30 – 7.23 (m, 2H, C2 – H), 7.22 – 7.16 (m, 3H, C1 – H and C3 – H), 3.58 (app h, *J* = 6.2 Hz, 1H, C6 – H), 3.47 – 3.39 (m, 1H, C8 – H_2), 3.35 – 3.27 (m, 1H, C8 – H_2), 2.91 (dd, *J* = 13.6, 6.3 Hz, 1H, C5 – H_2), 2.61 (dd, *J* = 13.6, 6.5 Hz, 1H, C5 – H_2), 1.54 (h, *J* = 7.1 Hz, 2H, C9 – H_2), 1.12 (d, *J* = 6.3 Hz, 3H, C7 – H_3), 0.87 (t, *J* = 7.4 Hz, 3H, C10 – H_3). **^{13}C{^{1}H} NMR** (101 MHz; $CDCl_3$) δ: 139.4 (C4), 129.6 (C1 or C3), 128.3 (C2), 126.1 (C1 or C3), 76.8 (C6), 70.6 (C8), 43.3 (C5), 23.4 (C9), 19.7 (C7), 10.8 (C10).

HRMS (APCI$^+$): Calculated for $[C_{12}H_{19}O]^+$ $[M + H]^+$: 179.1430. Found 179.1429.

Pentyl(1-methyl-2-phenylethyl)ether (6d)

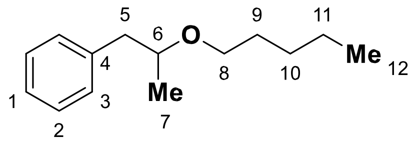

Iodobenzene (22.4 µL, 0.2 mmol, 1 eq.) and pentanol (217.4 µL, 2.0 mmol, 10 eq.) were reacted using general procedure 3, resulting in the title compound (24.7 mg, 60%) as a colorless oil (eluent: pentane to 2% Et_2O/pentane).

^{1}H NMR (400 MHz; $CDCl_3$) δ: 7.31 – 7.25 (m, 2H, C2 – H), 7.23 – 7.17 (m, 3H, C1 – H and C3 – H) 3.59 (h, J = 6.2 Hz, 1H, C6 – H), 3.47 (dt, J = 9.2, 6.6 Hz, 1H, C8 – H_2), 3.35 (dt, J = 9.2, 6.7 Hz, 1H, C8 – H_2), 2.92 (dd, J = 13.4 Hz, 6.2 Hz, 1H, C5 – H_2), 2.62 (dd, J = 13.4, 6.6 Hz, 1H, C5 – H), 1.53 (m, 2H, C9 – H_2), 1.33 – 1.24 (m, 4H, C10– H_2 and C11– H_2) ,1.13 (d, J = 6.2 Hz, 3H, C7 – H_3), 0.92 – 0.86 (m, 3H, C12 – H_3). **^{13}C{^{1}H} NMR** (101 MHz; $CDCl_3$) δ: 139.4 (C4), 129.6 (C1 or C3), 128.3 (C2), 126.1 (C1 or C3), 76.8 (C6), 69.0 (C8), 43.3 (C5), 29.9 (C9), 28.5 (C10 or C11), 22.7 (C10 or C11), 19.7 (C7), 14.2 (C12).

HRMS (APCI$^+$): Calculated for $[C_{14}H_{23}O]^+$ $[M + H]^+$: 207.1743. Found 207.1745.

Methyl(1-methyl-2-phenylethyl)ether (6e)

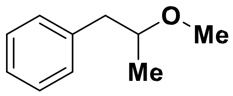

Iodobenzene (22.4 µL, 0.2 mmol, 1 eq.) and MeOH (81.0 µL, 2.0 mmol, 10 eq.) were reacted using general procedure 3, resulting in the title compound (9.5 mg, 34%) as a pale yellow oil (eluent: pentane to 2% Et_2O/pentane).

^{1}H NMR (400 MHz; $CDCl_3$) δ: 7.33 – 7.26 (m, 2H, Ar – H), 7.24 – 7.17 (m, 3H, Ar – H), 3.58 – 3.49 (m, 1H, HCOMe), 3.34 (s, 3H, OCH_3), 2.93 (dd, J = 13.5, 6.0 Hz, 1H, Ar – CH_2), 2.62 (dd, J = 13.5, 6.8 Hz, 1H $ArCH_2$), 1.13 (d, J = 6.1 Hz, 3H, $HCCH_3$). **^{13}C{^{1}H} NMR** (101 MHz; $CDCl_3$) δ: 139.2 (Ar – C), 129.6 (Ar – CH), 128.4 (Ar – CH), 126.2 (Ar – CH), 78.2 (HCOMe), 56.4 (OCH_3), 42.9 (CH_3), 19.0 ($HCCH_3$). The spectroscopic data is consistent with that of literature precedent.[15]

Octyl(1-methyl-2-phenylethyl)ether (6f)

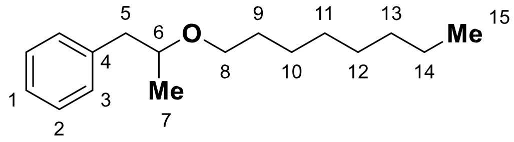

Iodobenzene (22.4 µL, 0.2 mmol, 1 eq.) and octanol (314.9 µL, 2.0 mmol, 10 eq.) were reacted using general procedure 3, resulting in the title compound (44.0 mg, 89%) as a colorless oil (eluent: pentane to 1.5% Et_2O/pentane).

^{1}H NMR (400 MHz; $CDCl_3$) δ: 7.30 – 7.23 (m, 2H, C2 – H), 7.22 – 7.15 (m, 3H, C1 – H and C3 – H), 3.57 (h, *J* = 6.4 Hz, 1H, C6 – H), 3.46 (dt, *J* = 9.1, 6.6 Hz, 1H, C8 – H_2), 3.33 (dt, *J* = 9.1, 6.7 Hz, 1H, C8 – H_2), 2.90 (dd, *J* = 13.5, 6.2 Hz, 1H, C5 – H_2), 2.61 (dd, *J* = 13.5, 6.6 Hz, 1H, C5 – H_2), 1.51 (m, 2H, C9 – H), 1.26 (m, 10H, C10 – H, C11 – H, C12 – H, C13 – H and C14 – H), 1.12 (d, *J* = 6.2 Hz, 3H, C7– H), 0.88 (t, *J* = 6.9 Hz, 3H, C15 – H). **^{13}C{^{1}H} NMR** (101 MHz; $CDCl_3$) δ: 139.4 (C4), 129.6 (C1 or C3), 128.3 (C2), 126.1 (C1 or C3), 76.8 (C6), 69.0 (C8), 43.3 (C5), 32.0 (C9), 30.3 (CH_2), 29.6 (CH_2), 29.4 (CH_2), 26.3 (CH_2), 22.8 (CH_2), 19.7 (C7), 14.2 (C15).

HRMS (APCI$^+$): Calculated for $[C_{17}H_{29}O]^+$ $[M + H]^+$: 249.2213. Found 249.2220.

2-Ethylhexanyl(1-methyl-2-phenylethyl)ether (6h)

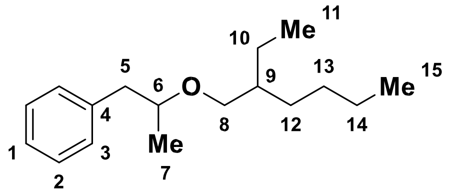

Iodobenzene (22.4 µL, 0.2 mmol, 1 eq.) and 2-ethylhexanol (312.7 µL, 2.0 mmol, 10 eq.) were reacted using general procedure 3, resulting in the title compound (41.5 mg, 90%, 1:1 d.r.) as a colorless oil (eluent: pentane to 1.5% Et_2O/pentane).

^{1} H NMR (400 MHz; $CDCl_3$) δ: 7.30 – 7.24 (m, 2H, C2 – H), 7.22 – 7.16 (m, 3H, C1 – H and C3 – H), 3.55 (h, *J* = 6.2 Hz, 1H, C6 – H), 3.36 (ddd, *J* = 9.1, 5.8, 0.7 Hz, 1H, C8 – H_2), 3.19 (dt, *J* = 9.1, 5.8 Hz, 1H, C8 – H_2) 1.46 – 1.15 (m, 9H, C9 – H, C10 – H, C12 – H, C13 – H and C14 – H), 1.13 (d, *J* = 6.1 Hz, 3H, C7 – H), 0.88 (t, *J* = 7.1 Hz, 3H, C15 – H), 0.82 (td, *J* = 7.4, 2.4 Hz, 3H, C11 – H). **^{13}C{^{1}H} NMR** (101 MHz; $CDCl_3$) δ: 139.6 (C4), 129.6 (C1 or C3), 128.2 (C2), 126.1 (C1 or C3), 77.0 (C6), 71.6 (C8), 71.5 (C8), 43.4 (C5), 40.1 (C9), 30.7 (CH_2), 30.6 (CH_2), 29.3 (CH_2), 29.1 (CH_2), 23.9 (CH_2), 23.2 (CH_2), 19.7 (C7), 14.3 (C15), 11.2 (C11), 11.1 (C11).

HRMS (APCI$^+$): Calculated for $[C_{17}H_{29}O]^+$ $[M + H]^+$: 249.2213. Found 249.2216.

***i*-Propyl(1-methyl-2-phenylethyl)ether (6i)**

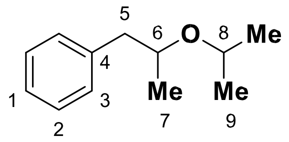

Iodobenzene (22.4 μL, 0.2 mmol, 1 eq.) and ^{i}PrOH (153.1 μL, 2.0 mmol, 10 eq.) were reacted using general procedure 3, resulting in the title compound (22.8 mg, 70%) as a pale yellow oil (eluent: pentane to 2.5% Et_2O/pentane).

^{1}H NMR (400 MHz; $CDCl_3$) δ: 7.30 – 7.24 (m, 2H, C2 – H), 7.22 – 7.16 (m, 3H, C1– H and C3 – H), 3.64 (app h, *J* = 6.3 Hz, 1H, C6 – H), 3.54 (sept, *J* = 6.1 Hz, 1H, C8 – H), 2.84 (dd, *J* = 13.4, 6.4 Hz, 1H, C5 – H_2), 2.61 (dd, *J* = 13.4, 6.5 Hz, 1H, C5 – H_2), 1.12 (d, *J* = 6.1 Hz, 6H, 2 × C9 – H_3), 1.01 (d, *J* = 6.1 Hz, 3H, C7 – H_3). **^{13}C{^{1}H} NMR** (101 MHz; $CDCl_3$) δ: 139.5 (C4), 129.7 (C1 or C3), 128.2 (C2), 126.1 (C1 or C3) 74.4 (C6), 69.7 (C8), 44.1 (C5), 23.0 (C9), 22.7 (C9), 20.9 (C7).

HRMS (APCI^{+}): Calculated for $[C_{12}H_{19}O]^{+}$ $[M + H]^{+}$: 179.1430. Found 179.1431.

1-(2-Butoxypropyl)-4-methylbenzene (6m)

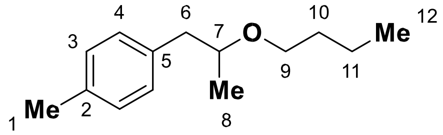

4-Iodotoluene (43.8 mg, 0.2 mmol, 1 eq.) and ^{n}BuOH (183.0 μL, 2.0 mmol, 10 eq.) were reacted using general procedure 3, resulting in the title compound (31.2 mg, 82%) as a colorless oil (eluent: pentane to 2% Et_2O/pentane).

^{1}H NMR (400 MHz; $CDCl_3$) δ: 7.09 (s, 4H, C3 – H and C4 – H), 3.56 (h, *J* = 6.1 Hz, 1H, C7 – H), 3.48 (dt, *J* = 9.2, 6.6 Hz, 1H, C9 – H), 3.38 (dt, *J* = 9.1, 6.6 Hz, 1H, C9 – H), 2.89 (dd, *J* = 13.5, 6.0 Hz, 1H, C6 – H), 2.58 (dt, *J* = 13.5, 6.9 Hz, 1H, C6 – H), 2.33 (s, 3H, C1 – H), 1.57 – 1.48 (m, 2H, C10 – H), 1.40 – 1.31 (m, 2H, C11 – H), 1.12 (d, *J* = 6.2 Hz, 3H, C8 – H), 0.90 (t, *J* = 7.3 Hz, 3H, C12 – H). **^{13}C{^{1}H} NMR** (101 MHz; $CDCl_3$) δ: 136.3 (C5), 135.5 (C2), 129.5 (C3 or C4), 129.0 (C3 or C4), 76.9 (C7), 68.6 (C9), 42.8 (C6), 32.4 (C10), 21.1 (C1), 19.7 (C8), 19.5 (C11), 14.0 (C12).

HRMS (APCI^{+}): Calculated for $[C_{14}H_{23}O]^{+}$ $[M + H]^{+}$: 207.1743. Found 207.1742.

1-(2-Butoxypropyl)-4-methoxybenzene (6n)

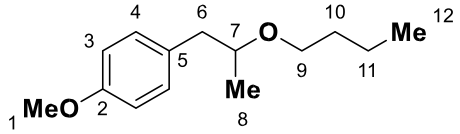

4-Iodoanisole (46.8 mg, 0.2 mmol, 1 eq.) and ^{n}BuOH (183.0 µL, 2.0 mmol, 10 eq.) were reacted using general procedure 3, resulting in the title compound (36.7 mg, 90%) as a colorless oil (eluent: pentane to 4% Et_2O/pentane).

^{1}H NMR (400 MHz; $CDCl_3$) δ: 7.15 – 7.07 (m, 2H, C4 – H), 6.85 – 6.78 (m, 2H, C3 – H), 3.79 (s, 3H, C1– H_3), 3.54 (app p, *J* = 6.2 Hz, 1H, C7 – H), 3.47 (dt, *J* = 9.3, 6.4 Hz, 1H, C9 – H_2), 3.36 (dt, *J* = 9.3, 6.7 Hz, 1H, C9 – H_2) , 2.85 (dd, *J* = 13.6, 6.1 Hz, 1H, C6 – H_2), 2.56 (dd, *J* = 13.6, 6.6 Hz, 1H, C6 – H_2) 1.56 – 1.46 (m, 2H, C10 – H_2), 1.39 – 1.29 (m, 2H, C11 – H_2), 1.11 (d, *J* = 6.1 Hz, C8 – H_3), 0.89 (t, *J* = 7.2 Hz, C12 – H_3). **^{13}C{^{1}H} NMR** (101 MHz; $CDCl_3$) δ: 158.1 (C2), 131.5 (C5), 130.5 (C4), 113.7 (C3), 76.9 (C7), 68.6 (C9), 55.4 (C1), 42.3 (C6), 32.4 (C10), 19.6 (C8 or C11),
19.5 (C8 or C11), 14.0 (C12).

HRMS (EI^+): Calculated for $[C_{14}H_{22}O_2]^+$ $[M]^+$: 222.1614. Found 222.1614.

1-(2-Butoxypropyl)-3-methoxybenzene (6o)

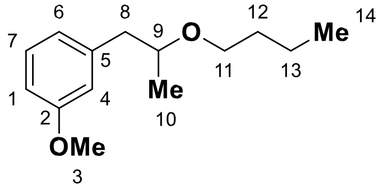

3-Iodoanisole (23.8 µL, 0.2 mmol, 1 eq.) and ^{n}BuOH (183.0 µL, 2.0 mmol, 10 eq.) were reacted using general procedure 3, resulting in the title compound (31.9 mg, 78%) as a colorless oil (3% Et_2O/pentane to 6% Et_2O/pentane).

^{1}H NMR (400 MHz; $CDCl_3$) δ: 7.19 (t, *J* = 7.7 Hz, 1H, C7 – H), 6.81 – 6.73 (m, 3H, C1 – H, C4 – H and C6 – H), 3.80 (s, 3H, C3 – H_3), 3.59 (h, *J* = 6.3 Hz, 1H, C9 – H), 3.48 (dt, *J* = 9.1, 6.5 Hz, C11 – H_2), 3.36 (dt, *J* = 9.1, 6.6 Hz, C11 – H_2), 2.89, (dd, *J* = 13.4, 6.3 Hz, 1H, C8 – H_2), 2.59 (dd, *J* = 13.4, 6.6, 1H, C8 – H_2), 1.56 – 1.47 (m, 2H, C12 – H_2), 1.39 – 1.29 (m, 2H, C13 – H_2), 1.13 (d, *J* = 6.1 Hz, 3H, C10 – H_3), 0.89 (t, *J* = 7.4 Hz, 3H, C14 – H_3). **^{13}C{^{1}H} NMR** (101 MHz; $CDCl_3$) δ: 159.6, (C2) 141.0 (C5), 129.2 (C1 or C7), 122.0 (C4 or C6), 115.3 (C4 or C6), 111.5 (C1 or C7), 76.7 (C9), 68.7 (C11), 55.3 (C3), 43.3 (C8), 32.4 (C12), 19.8 (C10), 19.5 (C13), 14.0 (C14).

HRMS (EI^+): Calculated for $[C_{14}H_{22}O_2]^+$ $[M]^+$: 222.1614. Found: 222.1614.

1-(2-Butoxypropyl)-2-naphthalene (6p)

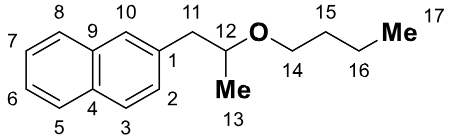

2-Iodonaphthalene (50.8 mg, 0.2 mmol, 1 eq.) and ^{n}BuOH (183.0 µL, 2.0 mmol, 10 eq.) were reacted using general procedure 3, resulting in the title compound (31.4 mg, 71%) as a colorless oil (pentane to 2% Et_2O/pentane).

^{1}H NMR (400 MHz; $CDCl_3$) δ: 7.83 – 7.74 (m, 3H, C2 – H, C3 – H and C5 – H), 7.65 (s, 1H, C10 – H), 7.48 – 7.40 (m, 2H, C6 – H and C7 – H), 7.37 (dd, *J* = 8.4, 1.7 Hz, 1H, C8 – H), 3.69 (h, *J* = 6.2 Hz, 1H, C12 – H), 3.50 (dt, *J* = 9.2, 6.5 Hz, 1H, C14 – H_2), 3.38 (dt, *J* = 9.2, 6.7 Hz, 1H, C14 – H_2), 3.08 (dd, *J* = 13.5, 6.2 Hz, 1H, C11 – H_2), 2.79 (dd, *J* = 13.5, 6.6 Hz, 1H, C11 – H_2), 1.57 – 1.48 (m, 2H, C15 – H_2), 1.39 – 1.28 (m, 2H, C16 – H_2), 1.17 (d, *J* = 6.1 Hz, 3H, C13 – H_3), 0.87 (t, *J* = 7.4 Hz, 3H, C17 – H_3). **^{13}C{^{1}H} NMR** (101 MHz; $CDCl_3$) δ: 137.0 (C1), 133.7 (C4), 132.2 (C9), 128.3 (C8), 127.9 (C2, C3, C5 or C8), 127.8 (C2, C3, C5 or C8), 127.7 (C2, C3, C5 or C8), 127.6 (C2, C3, C5 or C8), 126.0 (C6 or C7), 125.3 (C6 or C7), 76.8 (C12), 68.7 (C14), 43.5 (C11), 32.4 (C15), 19.8 (C13), 19.5 (C16), 14.0 (C17).

HRMS (EI^+): Calculated for $[C_{17}H_{22}O]^+$ $[M]^+$: 242.1665. Found: 242.1665.

1-(2-Butoxypropyl)-4-biphenyl (6q)

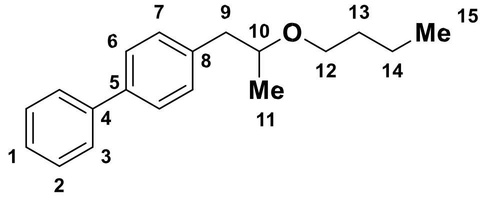

4-Iodobiphenyl (56.0 mg, 0.2 mmol, 1 eq.) and ^{n}BuOH (183.0 µL, 2.0 mmol, 10 eq.) was reacted using general procedure 3, resulting in the title compound (51,1 mg, 90%) as a colorless oil (eluent: pentane to 1.5% Et_2O/pentane).

^{1} H NMR (400 MHz; $CDCl_3$) δ: 7.61 – 7.56 (m, 2H, C3 – H), 7.54 – 7.49 (m, 2H, C6 – H), 7.46 – 7.39 (m, 2H, C2 – H) 7.33 (tt, *J* = 7.3, 1.2 Hz, 1H, C1 – H), 7.30 – 7.26 (m, 2H, C7 – H), 3.62 (h, *J* = 6.2 Hz, 1H, C10 – H), 3.50 (dt, *J* = 9.2, 6.5 Hz, 1H, C12 – H_2), 3.39 (dt, *J* = 9.2, 6.6 Hz, 1H, C12 – H_2) 2.94 (dd, *J* = 13.5, 6.2 Hz, 1H, C9 – H_2), 2.67 (dd, *J* = 13.5, 6.5 Hz, 1H, C9 – H_2) 1.57 – 1.49 (m, 2H, C13 – H_2), 1.39 – 1.29 (m, 2H, C14 – H_2), 1.16 (d, *J* = 6.1 Hz, 3H, C11 – H_3), 0.89 (t, *J* = 7.4 Hz, 3H, C15 – H_3). **^{13}C{^{1}H} NMR** (101 MHz; $CDCl_3$) δ: 141.2 (C4 or C5), 139.0 (C4 or C5), 138.5 (C8), 130.0 (C7),

128.8 (C2), 127.1 (C1, C3 and/or C6), 127.0 (C1, C3 and/or C6), 76.7 (C10), 68.6 (C12), 42.9 (C9), 32.3 (C13), 19.8 (C11), 19.5 (C14), 14.0 (C15).

HRMS (EI^+): Calculated for $[C_{19}H_{24}O]^+$ $[M]^+$: 269.1822. Found 269.1823.

1-(2-Butoxypropyl)-4-hydroxybenzene (6r)

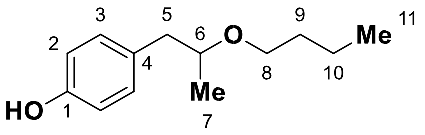

4-Iodophenol (44.0 mg, 0.2 mmol, 1 eq.) and ^{n}BuOH (183.0 µL, 2.0 mmol, 10 eq.) were reacted using general procedure 3, resulting in the title compound (26.4 mg, 69%) as a colorless oil (10% Et_2O/pentane to 20% Et_2O/pentane).

^{1}H NMR (400 MHz; $CDCl_3$) δ: 7.08 – 7.00 (m, 2H, C3 – H), 6.74 (m, 2H, C2 – H), 5.28, (br, 1H, OH), 3.61 – 3.43 (m, 2H, C6 – H, and C8 – H_2), 3.38 (dtd, J = 8.8, 6.6, 2.1 Hz, 1H, C8 – H_2) 2.84 (dd, J = 13.7, 6.2 Hz, 1H, C5 – H_2), 2.56 (dd, J = 13.7, 6.7 Hz, 1H, C5 – H_2), 1.57 – 1.46 (m, 2H, C9 – H_2), 1.38 –1.26 (m, 2H, C10 – H_2), 1.12 (d, J = 6.2 Hz, 3H, C7 – H_3) , 0.88 (t, J = 7.3, 3H, C11– H_3). **^{13}C{^{1}H} NMR** (101 MHz; $CDCl_3$) δ: 154.1 (C1), 131.3 (C4), 130.7 (C3), 115.2 (C2), 77.1 (C6), 68.7 (C9), 42.3 (C5), 32.2 (C10), 19.6 (C7 or C10), 19.5 (C7 or C10), 14.0 (C11).

HRMS (EI^+): Calculated for $[C_{13}H_{20}O_2]^+$ $[M]^+$: 208.1458. Found 208.1456

1-(2-Butoxypropyl)-3-bromobenzene (6s)

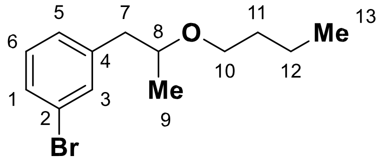

3-Bromoiodobenzene (25.5 µL, 0.2 mmol, 1 eq.) and ^{n}BuOH (183.0 µL, 2.0 mmol, 10 eq.) were reacted using general procedure 3, resulting in the title compound (25.2 mg, 46%) as a pale yellow oil (pentane to 2% Et_2O/pentane).

^{1}H NMR (400 MHz; $CDCl_3$) δ: 7.40 – 7.35 (m, 1H, C3 – H) , 7.35 – 7.29 (m, 1H, C1– H or C6 – H), 7.17 – 7.08 (m, 2H, C1– H or C6 – H, and C5 – H), 3.55 (h, J = 6.2 Hz, 1H, C8 – H), 3.47 (dt, J = 9.1, 6.5 Hz, 1H, C10 – H_2), 3.31 (dt, J = 9.2, 6.6 Hz, 1H, C10 – H_2), 2.83 (dd, J = 13.6, 6.6 Hz, 1H, C7 – H_2), 2.60 (dd, J = 13.6, 6.0 Hz, 1H, C7 – H_2), 1.54 – 1.45 (m, 2H, C11 – H_2), 1.37 – 1.28 (m, 2H, C12 – H_2), 1.13 (d, J = 6.1 Hz, 3H, C9 – H_3), 0.88 (t, J = 7.3 Hz, 3H, C13 – H_3). **^{13}C{^{1}H} NMR** (101 MHz; $CDCl_3$) δ: 141.8 (C4), 132.6 (C3), 129.8 (C1 or C6), 129.2 (C1 or C6), 128.3 (C5), 122.3 (C2), 76.4 (C8), 68.7 (C14), 42.9 (C7), 32.3 (C11), 19.8 (C9), 19.5 (C12), 14.0 (C13).

HRMS (APCI$^+$): Calculated for $[C_{13}H_{20}BrO]^+$ $[M + H]^+$: 271.0692. Found: 271.0697.

1-(2-Butoxypropyl)-4-fluorobenzene (6t)

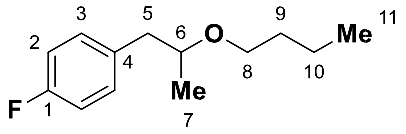

4-fluoroiodobenzene (23.2 µL, 0.2 mmol, 1 eq.) and ^{n}BuOH (183.0 µL, 2.0 mmol, 10 eq.) were reacted using a modified general procedure 3, with 10 mol% (**1**) used, resulting in the title compound (23.0 mg, 55%) as a colorless oil (pentane to 2% Et_2O/pentane).

^{1}H NMR (400 MHz; $CDCl_3$) δ: 7.18 – 7.13 (m, 2H, C3 – H), 6.99 – 6.93 (m, 2H, C2 – H), 3.54 (h, *J* = 6.1 Hz, 1H, C6 – H), 3.47 (dt, *J* = 9.2, 6.5 Hz, 1H, C8 – H_2), 3.31 (dt, *J* = 9.2, 6.7 Hz, 1H, C8 – H_2) , 2.83 (dd, *J* = 13.7, 6.5 Hz, 1H, C5 – H_2), 2.61 (dd, *J* = 13.7, 6.1 Hz, 1H, C5 – H_2), 1.53 – 1.46 (m, 2H, C9 – H_2), 1.36 – 1.26 (m, 2H, C10– H_2), 1.12 (d, *J* = 6.1 Hz, 3H, C7– H_3), 0.88 (t, *J* = 7.4 Hz, 3H, C11– H_3). **^{13}C{^{1}H} NMR** (101 MHz; $CDCl_3$) δ: 161.6 (d, *J* = 243.6 Hz, C1), 135.1 (d, *J* = 3.3 Hz, C4), 130.9 (d, *J* = 7.7 Hz, C3), 115.0 (d, *J* = 21.0 Hz, C2), 76.6 (C6), 68.7 (C8), 42.4 (C5), 32.3 (C9), 19.7 (C7 or C10), 19.5 (C7 or C10), 14.0 (C11). **^{19}F NMR** (376 MHz; $CDCl_3$) δ: 117.6.

HRMS (APCI$^+$): Calculated for $[C_{13}H_{20}FO]^+$ $[M + H]^+$: 211.1491. Found: 211.1493.

1-(2-Butoxypropyl)-4-bromobenzene (6u)

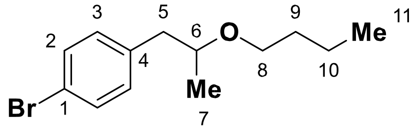

4-bromoiodobenzene (56.8 mg, 0.2 mmol, 1 eq.) and ^{n}BuOH (183.0 µL, 2.0 mmol, 10 eq.) were reacted using a modified general procedure 3, with 10 mol% (**1**) used, resulting in the title compound (34.4 mg, 69%) as a colorless oil (pentane to 2% Et_2O/pentane).

^{1}H NMR (400 MHz; $CDCl_3$) δ: 7.44 – 7.33 (m, 2H, C2 – H), 7.11– 7.03 (m, 2H, C3 – H), 3.54 (h, *J* = 6.2 Hz, 1H, C6 – H), 3.46 (dt, *J* = 9.2, 6.5 Hz, 1H, C8 – H_2), 3.31 (dt, *J* = 9.2, 6.6 Hz, 1H, C8 – H_2), 2.81 (dd, *J* = 13.6, 6.6 Hz, 1H, C5 – H_2), 2.59 (dd, *J* = 13.6, 6.0 Hz, 1H, C5 – H_2), 1.54 – 1.44 (m, 2H, C9 – H_2), 1.36 – 1.27 (m, 2H, C10 – H_2), 1.12 (d, *J* = 6.1 Hz, 3H, C7 – H_3), 0.88 (t, *J* = 7.4 Hz, 3H, C11 – H_3). **^{13}C{^{1}H} NMR** (101 MHz; $CDCl_3$) δ: 138.4 (C4), 131.4 (C2 or C3), 131.3 (C2 or C3), 120.0 (C1), 76.4 (C6), 68.7 (C8), 42.7 (C5), 32.3 (C9), 19.7 (C7 or C10), 19.5 (C7 or C10), 14.0 (C11).

HRMS (APCI$^+$): Calculated for $[C_{13}H_{20}BrO]^+$ $[M + H]^+$: 271.0692. Found: 271.0693.

_n_-Butyl(1-_n_-butyl-2-phenylethyl)ether (7)

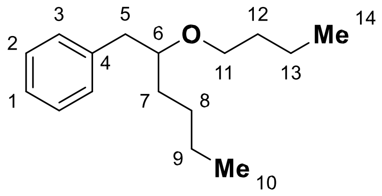

A Youngs flask was charged with $NaHCO_3$ (16.8 mg, 0.20 mmol) and $AgSbF_6$ (82.5 mg, 0.24 mmol), which were then dissolved in 1,2-dichlorobenzene (0.5 mL). Separately, (**1**) (5.5 mg, 5 mol%), iodobenzene (22.4 µL, 0.20 mmol), ^{n}BuOH (183.0 µL, 2.00 mmol) and 1-hexene (50.0 µL, 0.40 mmol) were dissolved in 1,2-dichlorobenzene (0.5 mL). This solution was added to the Youngs flask and the reaction was then heated at 75 °C for 18 hours. The mixture was then cooled to rt and purified by flash column chromatography (hexane to 2% EtOAc:hexane) to provide the desired product (**7**) as a colorless oil (37.0 mg, 69%).

^{1}H NMR (400 MHz; $CDCl_3$) δ: 7.28-7.20 (m, 2H, C2-H), 7.20-7.11 (m, 3H, C1-H and C3-H), 3.51-3.41 (m, 3H, C6-H and C11-H_2), 2.79 (dd, J = 13.6, 6.3 Hz, 1H, C5-H_2), 2.66 (dd, J = 13.6, 6.4 Hz, 6.3 Hz, 1H, C5-H_2), 1.51-1.35 (m, 4H, C7-H_2 and C12-H_2), 1.37-1.26 (m, 6H, C8-H_2, C9-H_2, C13H_2), 0.87 (app tt, J = 7.1, 1.7 Hz, 3H, C10-H_3 and C14-H_3). **^{13}C{^{1}H} NMR** (101 Hz; $CDCl_3$) δ: 139.6 (C4), 129.6 (C3), 128.3 (C2), 126.0 (C1), 81.1 (C6), 69.4 (C11), 41.0 (C5), 34.0 (C7), 32.4 (C12), 27.9 (C8 or C13), 22.9 (C9), 19.5 (C8 or C13), 14.2 (C10 or C14), 14.0 (C10 or C14).

HRMS (APCI)$^{+}$: Calculated for $[C_{16}H_{26}O]^{+}$ $[M+H]^{+}$: 235.2056. Found: 235.2059.

4.4. *Mechanistic studies on the alkene oxyarylation reactions*

The studies in this section provide evidence for the mechanism proposed in the main paper for the 1,2-oxyarylation of ethylene, propylene and higher alkenes using Au(I) NHC complex **1** (see Scheme 4 in the main paper). These experiments highlight the role of the Ag(I) salt and provide evidence for π-coordination of the alkene to the Au-center, in advance of attack by the alcohol.

*Exposure of oxidative addition product **2a** to ^{n}BuOH and $AgSbF_6$ in the absence of alkene*

Following oxidative addition, there is a possibility that the alcohol nucleophile engages the Au(III)-center prior to the alkene. To investigate this, a stoichiometric study was undertaken in the absence of alkene:

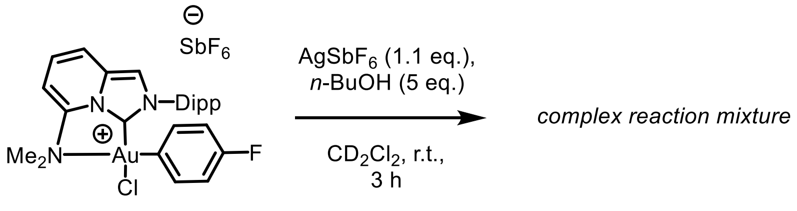

2a

(**2a**) (10 mg, 11.3 μmol, 1 eq.) and $AgSbF_6$ (4 mg, 12.4 mmol, 1.1 eq.) were dissolved in dry CD_2Cl_2 (0.2 mL) in the absence of light at room temperature. A solution of ^{n}BuOH (1 M in dry CH_2Cl_2, 57 μL, 56.5 mmol, 5 eq.) was added, followed by dry CD_2Cl_2 (0.8 mL). After stirring for three hours, an aliquot of the reaction mixture was taken for analysis by ^{1}H NMR spectroscopy. A complex mixture of species was observed by GC-MS and NMR spectroscopy. When the reaction was repeated without $AgSbF_6$, no reaction was observed.

This experiment shows that clean reactivity requires the presence of the alkene reaction partner, as observed for the conversion of **2b** to **5a** (see Scheme 4C in the main paper). Based on these results, we suggest that, following aryl iodide oxidative addition, the alkene reaction partner engages the Au-center prior to attack by the alcohol. Further experiments were undertaken to support this, as described below.

*Exposure of oxidative addition product **2a** to norbornene and $AgSbF_6$ in the absence of alcohol*

To provide evidence for π-coordination of the alkene to the Au(III)-center, **2a** was generated and exposed to increasing equivalents of norbornene in the presence of $AgSbF_6$ (1.1 equivalents):

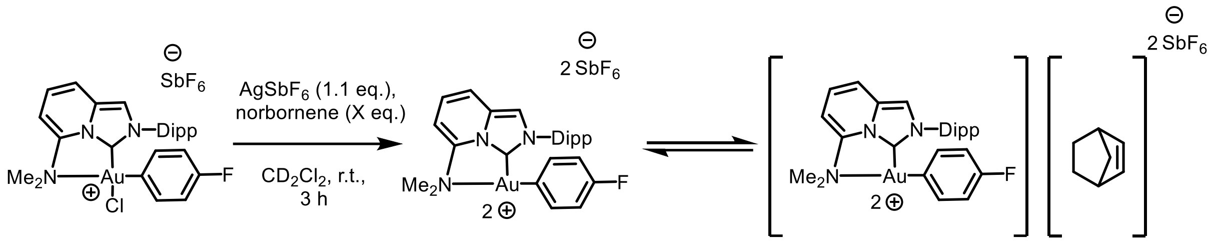

(**2a**) (10 mg, 11.3 μmol, 1 eq.) and $AgSbF_6$ (4 mg, 12.4 mmol, 1.1 eq.) were dissolved in dry CD_2Cl_2 (0.2 mL) in the absence of light at room temperature. A solution of norbornene (1 M in dry CH_2Cl_2) (1, 5 or 10 equivalents) was added, followed by dry CD_2Cl_2 (0.8 mL). After three hours, an aliquot of the reaction mixture was taken and analysed by ^{1}H NMR spectroscopy.

As shown below, use of 1 equivalent of norbornene resulted in a significant downfield shift of the characteristic olefinic signal from ~6.0 ppm to ~6.45 ppm. As the equivalents of norbornene was increased (to 5 and then 10 equivalents), the olefinic signal moved upfield, tending towards the shift of free norbornene. These observations are consistent with reversible coordination of norbornene to the Au(III)-center of the nominal dication of **2a**. Addition of a chloride source (tetrabutylammonium chloride, 1.1 equivalents) returned the olefinic signal to the shift of free norbornene. In these experiments, we did not observe migratory insertion of the alkene into the Au-C(sp^2) bond. Accordingly, for the processes in Scheme 4 of the main paper, we suggest that C-C bond formation occurs after outer sphere attack of the alcohol onto the π-coordinated alkene.

^{1}H NMR spectra for norbornene titration experiments.

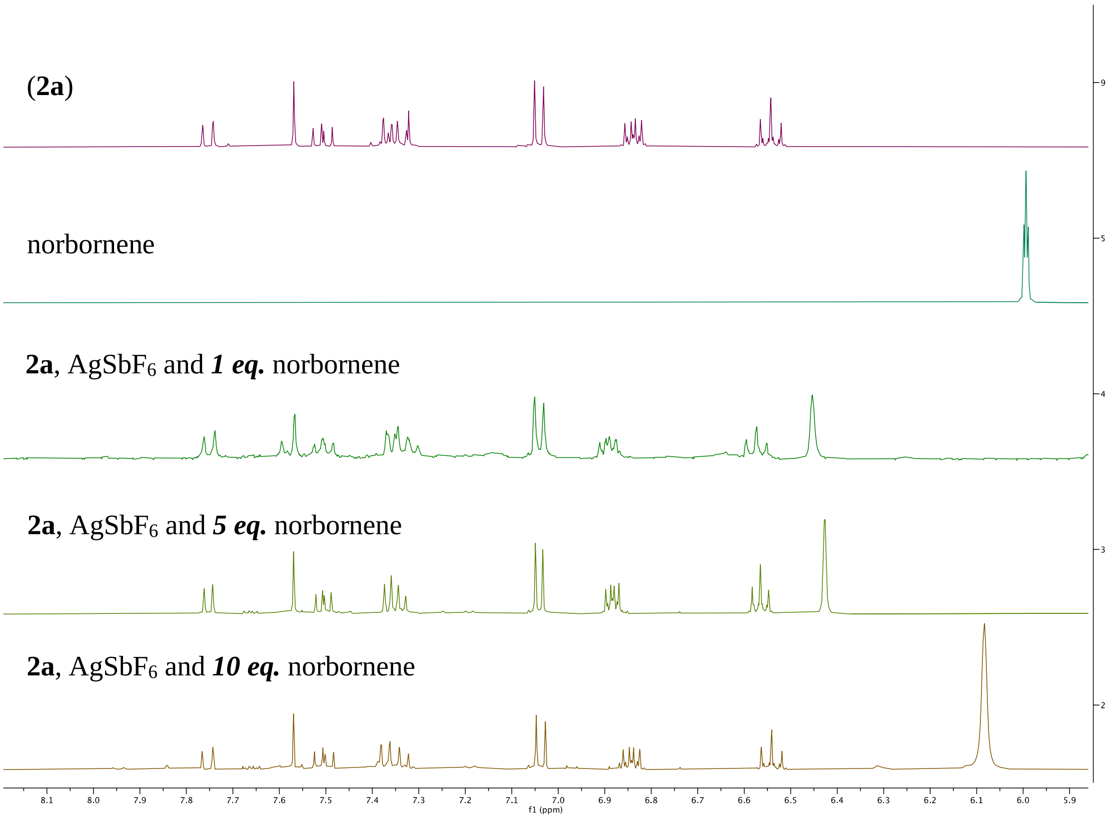

Attempted alkene aminoarylation reaction using p-toluidine

As noted in the main manuscript, replacement of the alcohol nucleophile with *p*-toluidine did not result in alkene 1,2-aminoarylation, with significant formation of colloidal gold being observed.

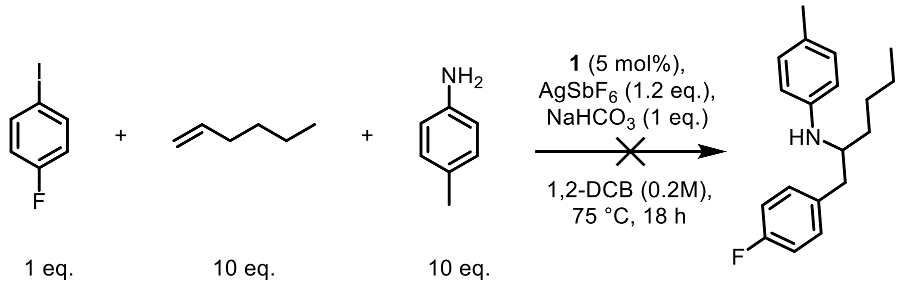

$NaHCO_3$ (1 eq.) and $AgSbF_6$ (1.2 eq.) were dissolved in 1,2-dichlorobenzene (0.5 mL). Separately [Au] (10 mg, 0.018 mmol, 0.05 eq.), 4-fluoroiodobenzene (42 µl, 36.0 µmol, 1 eq.), *p*-toluidine (385 mg, 3.60 mmol, 10 eq.) and 1-hexene (0.45 mL, 3.60 mmol, 10 eq.) were dissolved in 1,2-dichlorobenzene (0.5 mL). The two solutions were mixed at r.t. in the absence of light prior to heating at 75 °C for 18 hours. The reaction mixture was then cooled to r.t. and analysed by GC-MS. The reaction mixture displayed an intense purple coloration characteristic of colloidal gold. The desired alkene aminoarylation product was not detected.

5. Mechanism of oxidative addition

5.1. NMR studies

(1) (10 mg, 18.1 µmol, 1 eq.) and 4-fluoroiodobenzene (42 µL, 0.36 mmol, 10 eq.) were dissolved in 1.3 mL of dry CH_2Cl_2. Separately $AgSbF_6$ (6.2 mg, 18.1 µmol, 1 eq.) was dissolved in 1.3 mL of dry CH_2Cl_2 in the absence of light. Both solutions were cooled to -78 °C, then the solutions were mixed. A sample of the reaction mixture was taken and allowed to warm to room temperature within the NMR spectrometer. The reaction was monitored every 2 mins using ^{19}F NMR spectroscopy in the presence of trifluorotoluene, until completion.

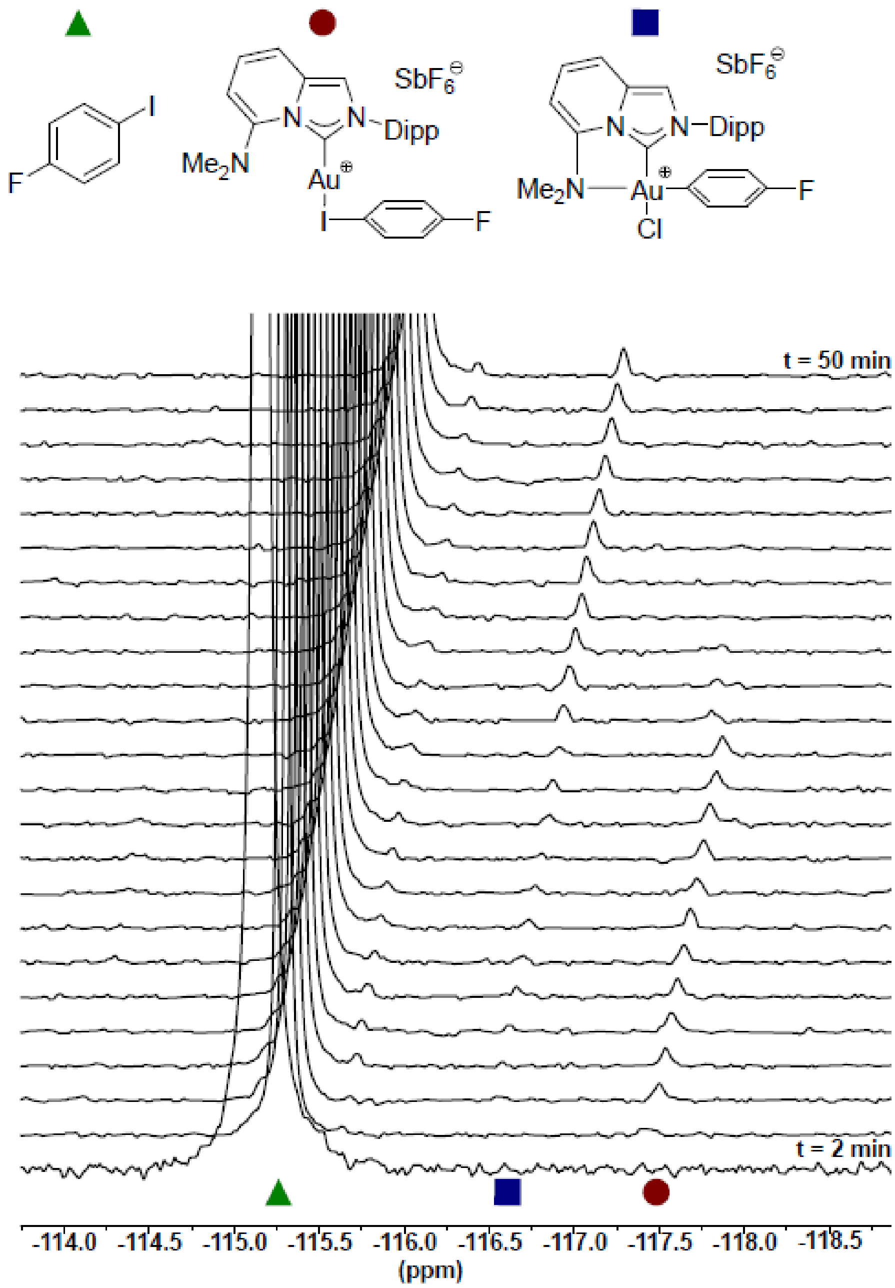

^{1}H NMR spectroscopy was used to monitor the reaction of **1** with different *p*-substituted aryl iodides. This was done by adapting the general oxidative addition procedure in Section 3.2. The reaction mixture was kept cool (-78 °C) until it was placed within the spectrometer. Reaction progress was monitored by integrating the $-NMe_2$ signal of **1** against 1,3,5-trimethoxybenzene as an internal standard. The initial oxidative addition rates for each aryl iodide were very similar, indicating no significant electronic preference.

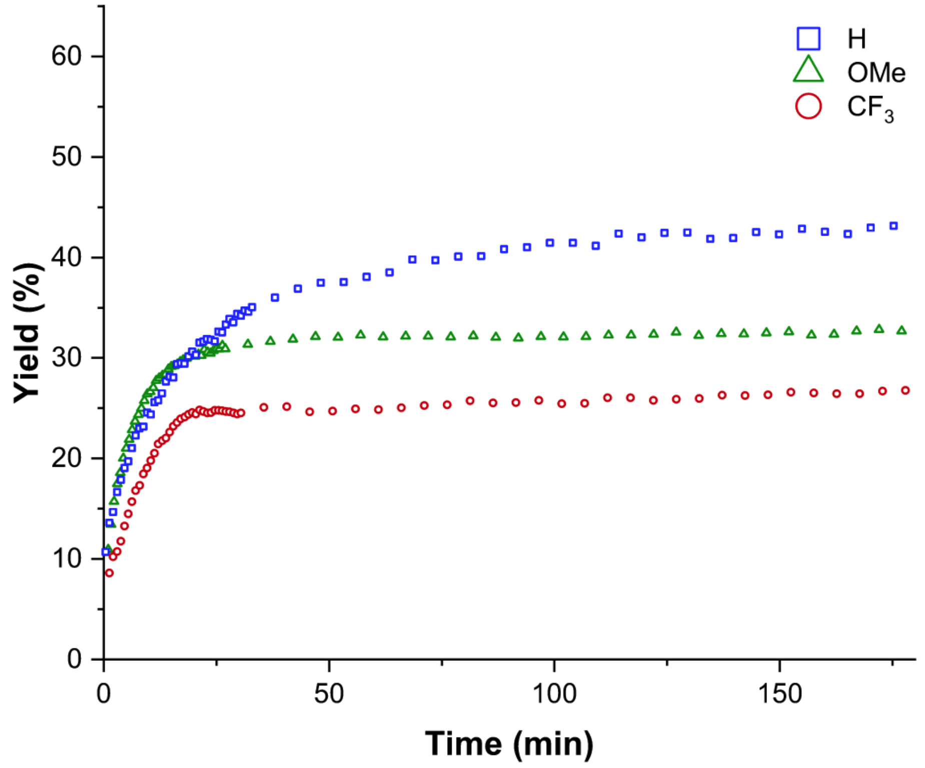

Oxidative addition reactions with *p*-H, *p*-OMe and *p*-CF_3 substituted aryl iodides monitored by ^{1}H NMR spectroscopy, yields determined by an 1,3,5-trimethoxybenzene internal standard

Subsequently, a competition experiment was conducted, as described below. This was conducted in the presence of AgCl (10 equivalents) to ensure fast salt metathesis (see the main manuscript) after the initial oxidative addition process.

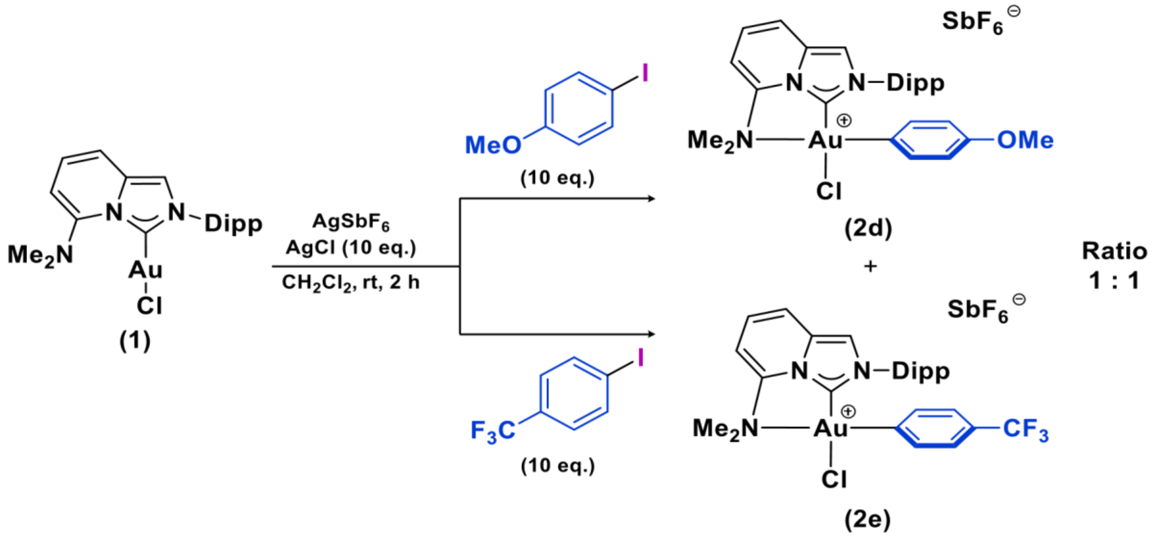

(**1**) (20 mg, 36.1 μmol, 1 eq.), 4-iodoanisole (21.2 mg, 0.36 mmol, 10 eq.) and 4-iodotrifluorotoluene (26.5 μL, 0.36 mmol, 10 eq.) were dissolved in 2.5 mL of dry CH_2Cl_2. Separately $AgSbF_6$ (12.4 mg, 36.1 μmol, 1 eq.) and AgCl (51.7 mg, 0.36 mmol, 10 eq.) were dissolved in 2.5 mL of dry CH_2Cl_2 in the absence of light. The solution were then mixed in the absence of light and stirred at room temperature for 2 hours. A sample of the reaction mixture was taken and analyzed using ^{1}H NMR spectroscopy with a known quantity of 1,3,5-trimethoxybenzene standard. **2d** and **2e** were identified in a ratio of 1:1.

This result supports the aforementioned rate experiments. No strong electronic trend for oxidative addition is evident.

5.2. Computational studies

General considerations

All calculations were performed using *Gaussian 09, Revision D.01*.[16] The ωB97-XD functional[17] with an ultrafine integration grid was used throughout. Ahlrichs' def2-TZVP basis set was used on Au; def2-SVP on C, N, I, Sb, F and def2-SV on all other atoms.[18] The 60-electron def2 pseudopotentials was used for Au; 28-electron def2 pseudopotentials were used for I and Sb.[19] Solvation (CH_2Cl_2) was modelled using the SMD model.[20] The nature of all stationary points was confirmed by analysis of the harmonic vibrational frequencies. Natural bond orbital (NBO) analysis[21] was performed using *NBO Version 3.1* as implemented in *Gaussian 09*.[22] Visualization of ground state and transition state structures were generated in CYLview20.[23]

In all cases, the 2,6-diisopropylphenyl (Dipp) substituent on the NHC complex **1** was replaced with 2,6-dimethylphenyl for simplification. Experimentally, oxidative addition complexes were all determined to be Au(III) chloride complexes (see Section 3.1). The computational modelling here is for the formation of Au(III) iodide complexes, generated as a direct result of a C–I bond cleavage, where any I/Cl exchange is assumed not to be rate-limiting. The SbF_6^- anion was considered to have no significant role in the C–I cleavage step of the overall oxidative addition mechanism; however, an exchange resulting in the formation of the *I*-bound intermediate from the [Au]···SbF_6 adduct (generated from an initial chloride abstraction by $AgSbF_6$) is considered in Figure S1.

Oxidative addition potential energy surface

The full oxidative addition mechanism for iodobenzene as the substrate starting from a Au(I) SbF_6 adduct was modelled (Figure S1). A slightly endergonic ($\Delta\Delta G$ = 4.1 kcal mol^{-1}) exchange with iodobenzene proceeds *via* a small barrier ($\Delta\Delta G^{\ddagger}$ = 5.8 kcal mol^{-1}) to give the η^1-*I*-bound pre-oxidative addition complex. The Au(III) oxidative addition complex is then generated with a much higher barrier than the SbF_6/iodobenzene exchange ($\Delta\Delta G^{\ddagger}$ = 20.1 kcal mol^{-1}). A similar high activation barrier for oxidative addition compared to SbF_6/iodobenzene exchange was reported by Bourissou and Amgoune *et al.* with *P,N*-ligand MeDalPhos.[24]

Figure S1: Oxidative addition potential energy surface for iodobenzene. Electronic energies including zero-point correction, are given in parentheses. Transition state and O.A. product geometry bond lengths are quoted in Å, C_{NHC}–Au–N bond angles are quoted in degrees (°) and hydrogen atoms are omitted for clarity. R = 2,6-dimethylphenyl.

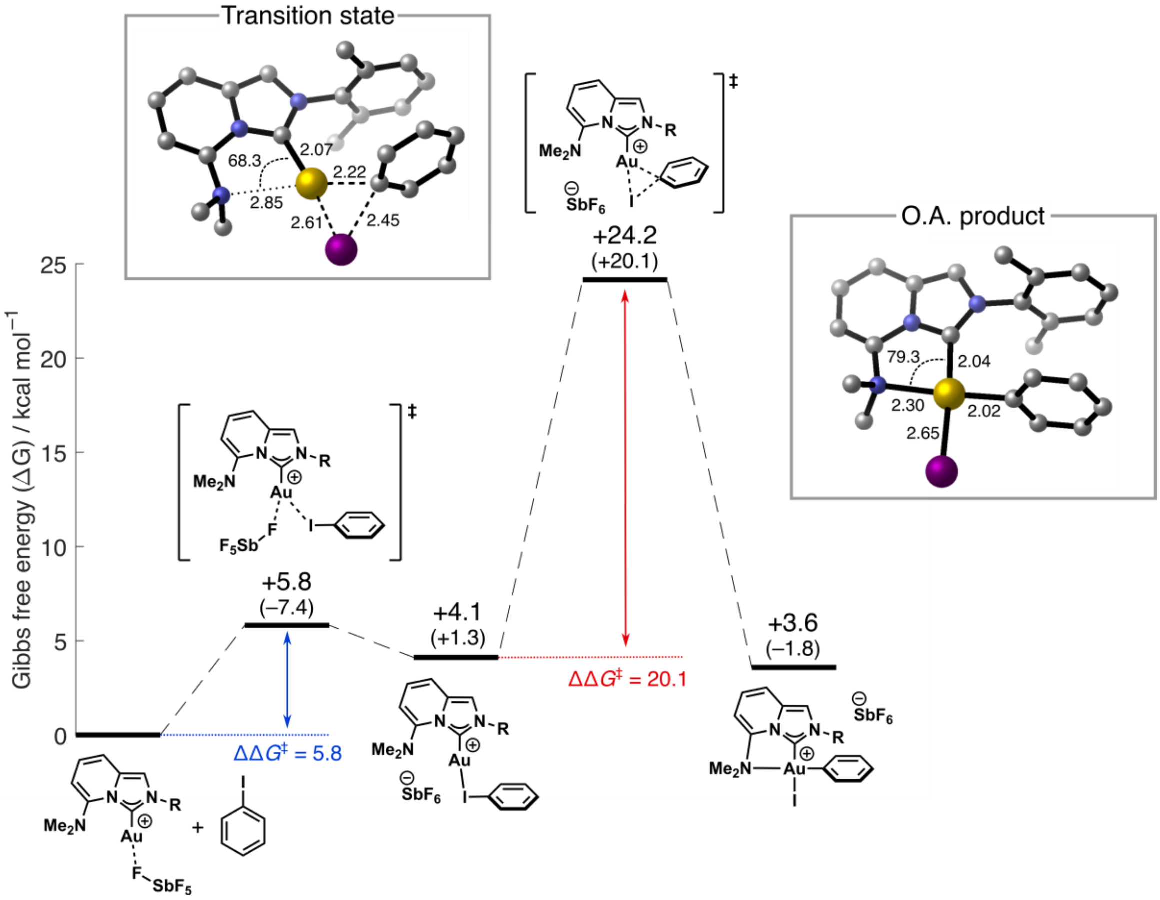

Influence of ligand NMe_2 hemi-labile moiety on oxidative addition

Given that the monodentate Au complex IPrAuCl is inactive towards oxidative addition of aryl iodides or indeed in the 1,2-difunctionalization of ethylene, the role of the hemi-labile NMe_2 group of complex **1** was investigated computationally (Figure S2). The oxidative addition potential energy surface for complexes with and *without* the hemi-labile N donor were compared. In the absence of the hemi-labile NMe_2 group, the barrier to oxidative addition is slightly higher ($\Delta E^‡$ = 22.6 kcal mol^{-1} *vs* 18.9 kcal mol^{-1}). The most pronounced difference is that the overall reaction is significantly more endothermic (ΔE = 12.6 kcal mol^{-1} *vs* –3.1 kcal mol^{-1}). Additionally, NBO analysis revealed N→Au {$\Delta E(2)$ = 3.2 kcal mol^{-1}} and N→$\sigma^*_{(Au–C_{ipso})}$ {$\Delta E(2)$ = 47.0 kcal mol^{-1}} stabilizing interactions in the transition state and Au(III) oxidative addition product, respectively for the complex bearing the NMe_2 group.

Figure S2: Oxidative addition potential energy surfaces for iodobenzene with ligands with and without the hemi-labile NMe_2 group. All energies include zero-point correction. The transition state geometry bond lengths are quoted in Å and hydrogen atoms are omitted for clarity. R^1 = 2,6-dimethylphenyl.

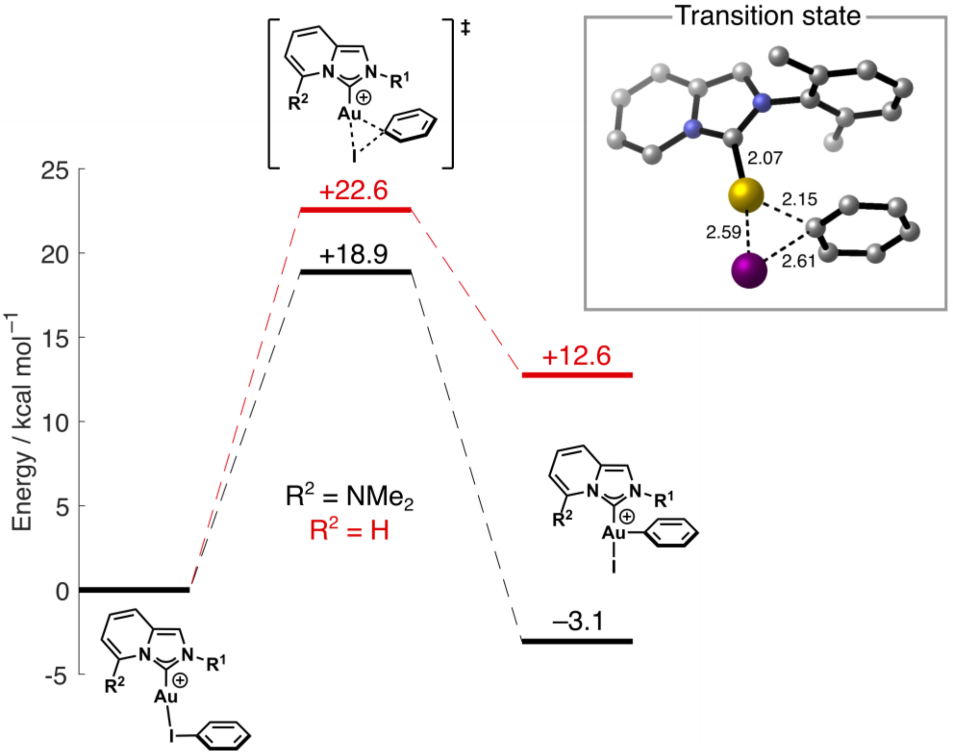

Overall, these results indicate that the role of a second donor atom is crucial for enabling oxidative addition of aryl iodides, having influence on the barrier and overall thermodynamics of the reaction. In

line with previous studies,[24,25] the ML_2 coordination generated as a result of the second donor atom is crucial for providing a small bite angle[26] ($\angle C_{NHC}$–Au–N = 68.3° and 79.3° for the transition state and Au(III) product, respectively) required for oxidative addition.

Arene electronic comparison

The arene electronic effects on the mechanism of oxidative addition were investigated with 4-iodoanisole (*p*-OMe) and 4-iodobenzotrifluroide (*p*-CF_3) chosen as representative examples of electron-rich and electron-poor aryl iodides (Figure S3). The barriers to oxidative addition for the *p*-OMe and *p*-CF_3 aryl iodides are extremely similar (*p*-OMe: $\Delta E^{\ddagger}$ = 19.1 kcal mol^{-1}; *p*-CF_3: $\Delta E^{\ddagger}$ = 18.7 kcal mol^{-1}). The transition state geometries and NBO charges on Au for each of the transition states are also similar, indicating there are limited changes to the Au center with electron-rich or electron-poor arenes.

Furthermore, stabilizing interactions {$\Delta E(2)$} between iodine and the Au–C_{NHC} σ^* orbital in the transition states were also shown to be similar (*p*-OMe: 28.9 kcal mol^{-1}, *p*-CF_3: 32.8 kcal mol^{-1}).[27] These results are in line with qualitative experimental data, where there is a limited aryl iodide electronic effect on the rate of oxidative addition (see Section 5.1).

Figure S3: Oxidative addition potential energy surfaces for 4-iodoanisole and 4-iodobenzotrifluoride. All energies include zero-point correction. Transition state bond lengths are quoted in Å and hydrogen atoms are omitted for clarity. Selected NBO partial charges are given in ***bold italic***. R^1 = 2,6-dimethylphenyl.

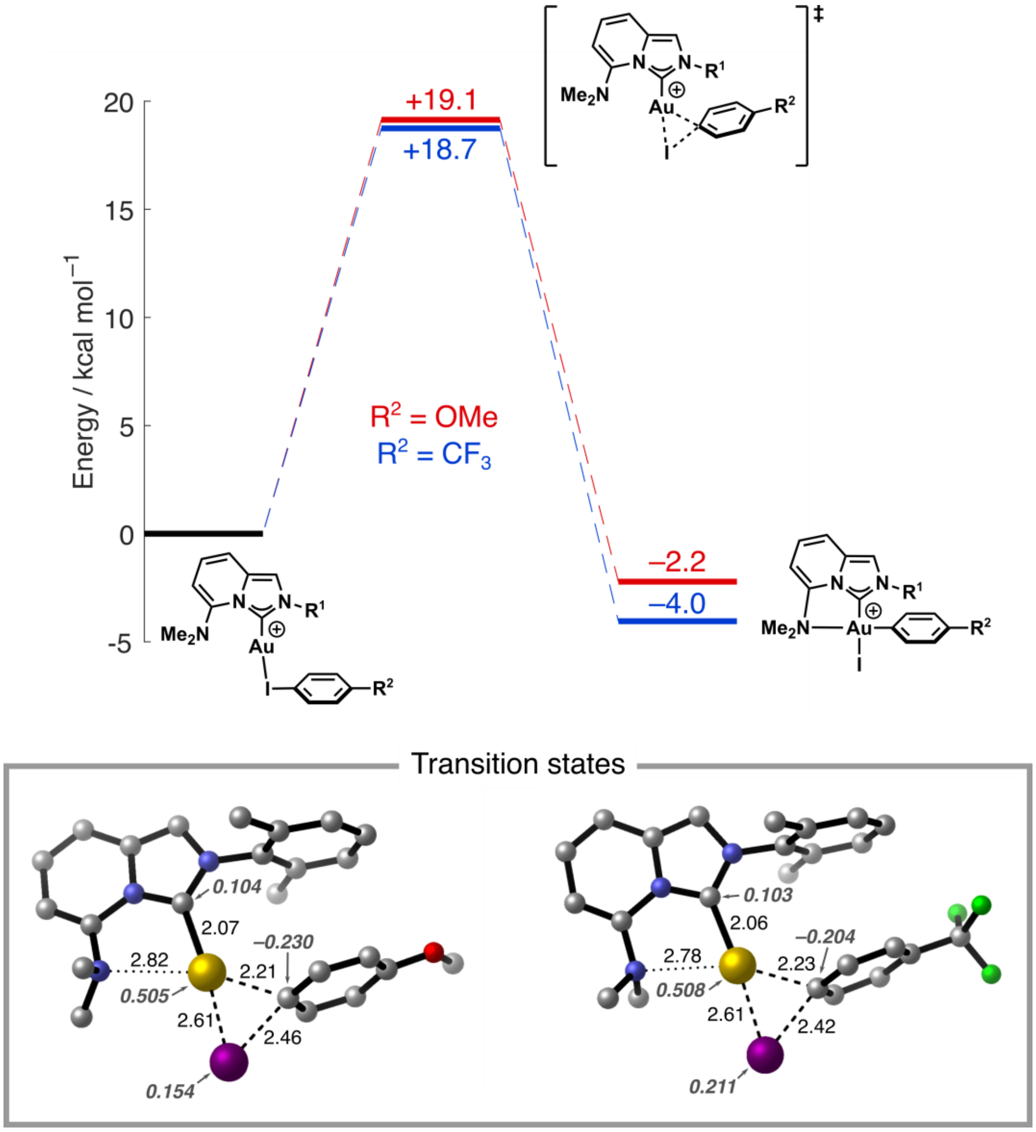

Energies (au) and Cartesian coordinates (Å) for stationary points

(NMe_2-NHC)Au···SbF_6 adduct

SCF = –1797.35973

ZPE = –1797.01375

Δ*G* = –1797.07837

N	-3.05930	-0.38160	0.13200
C	-4.23280	0.32490	0.19650
C	-1.98210	0.42600	0.04280
C	-3.89180	1.65150	0.14370
N	-2.48770	1.69300	0.08240
C	-1.79960	2.92470	0.03450
C	-2.54660	4.05980	-0.08100
C	-3.97940	4.03210	-0.08830
C	-4.64850	2.85840	0.06080
H	-2.02890	5.01930	-0.11650
H	-4.52520	4.97490	-0.17680
H	-5.73790	2.79900	0.09080
H	-5.20380	-0.15960	0.25290
C	-2.77990	-4.56460	0.13810
C	-3.01540	-3.88890	-1.05810
C	-3.11860	-2.49470	-1.08340
C	-2.97680	-1.81720	0.13580
C	-2.73900	-2.46510	1.35560
C	-2.64360	-3.86040	1.33260
H	-2.69970	-5.65550	0.13880
H	-3.11600	-4.44820	-1.99290
H	-2.45610	-4.39740	2.26720
C	-2.55480	-1.68680	2.63020
H	-3.33570	-0.91790	2.75880
C	-3.34340	-1.74370	-2.36770
H	-3.41510	-2.43980	-3.21790
N	-0.41570	2.86850	0.11270
C	0.32610	3.90440	-0.57870
H	1.37590	3.57940	-0.65860
H	-0.07410	4.04280	-1.59530
H	0.30640	4.87960	-0.04770
C	0.12180	2.58350	1.44030
H	1.15330	2.20940	1.34890
H	0.12470	3.49720	2.07110
H	-0.47760	1.81510	1.95220
Au	-0.13970	-0.22510	-0.27340
F	1.84660	-0.98270	-0.68480
Sb	3.54360	-0.19860	0.02560
F	4.05300	-1.96960	0.52570
F	5.15910	0.55690	0.70470
F	2.88500	1.51890	-0.52260
F	2.65060	0.01590	1.70990
F	4.28700	-0.46060	-1.71390
H	-2.51240	-1.04370	-2.56790
H	-4.27270	-1.14850	-2.33410
H	-1.57900	-1.16770	2.63340
H	-2.58250	-2.35700	3.50360

Iodobenzene

SCF = –529.17186

ZPE = –529.08083

Δ*G* = –529.11250

C	1.21410	1.24770	0.00000
C	0.00000	0.55700	0.00000
C	-1.21410	1.24760	0.00000
C	-1.20720	2.64350	0.00000
C	-0.00010	3.34360	0.00000
C	1.20710	2.64360	0.00000
H	2.16340	0.70570	0.00000
I	0.00000	-1.55310	0.00000
H	-2.16340	0.70560	0.00000
H	-2.15760	3.18480	0.00000
H	-0.00010	4.43730	0.00000
H	2.15750	3.18490	0.00000

(NMe_2-NHC)Au SbF_6 / iodobenzene exchange TS

SCF = –2326.54422

ZPE = –2326.10633

Δ*G* = –2326.18159

Au	0.43950	0.26710	-0.05090
I	-0.61420	-2.54780	-0.92700
Sb	-3.45280	1.01160	-0.39260
N	3.41100	0.25160	-0.20960
C	4.56480	0.95700	0.02030
C	2.30470	1.00180	-0.02080
C	4.18160	2.22210	0.38080
N	2.77670	2.23750	0.31910
C	2.05240	3.41090	0.62480
C	2.75490	4.48030	1.09800
C	4.18170	4.46080	1.22700
C	4.89420	3.36890	0.84270
H	2.20760	5.39350	1.33560
H	4.69020	5.35130	1.60530
H	5.98340	3.32380	0.89770
H	5.55100	0.50890	-0.06640

C	3.36980	-3.80190	-1.28230
C	3.45130	-3.44210	0.06160
C	3.45710	-2.09660	0.44360
C	3.38770	-1.13810	-0.57680
C	3.29560	-1.46570	-1.93620
C	3.28690	-2.82380	-2.27090
H	3.36320	-4.85930	-1.56170
H	3.50620	-4.21610	0.83280
H	3.21360	-3.11360	-3.32320
C	3.16890	-0.39640	-2.98710
H	3.27880	-0.82490	-3.99550
C	3.49440	-1.68910	1.89160
H	3.55020	-2.57420	2.54460
N	0.68160	3.37050	0.42350
C	-0.13850	4.16620	1.31510
H	-1.18210	3.82940	1.21140
H	0.18000	4.01030	2.35800
H	-0.10290	5.25260	1.08580
C	0.24960	3.44810	-0.96850
H	-0.77990	3.06960	-1.05750
H	0.27760	4.49590	-1.33550
H	0.89870	2.83910	-1.61580
F	-1.86680	0.45310	0.61250
F	-3.04690	2.83420	0.05840
F	-4.98770	1.57860	-1.38390
F	-3.82370	-0.82250	-0.81800
F	-4.46860	0.83410	1.21940
F	-2.33690	1.14790	-1.95340
H	2.58700	-1.11980	2.16340
H	4.36320	-1.04510	2.11390
H	3.92880	0.39410	-2.86170
H	2.17790	0.09040	-2.93020
C	-1.39600	-2.96920	3.88190
C	-2.43370	-2.56840	3.03900
C	-2.22770	-2.46240	1.66260
C	-0.96870	-2.76490	1.14370
C	0.07860	-3.16780	1.97230
C	-0.14220	-3.26820	3.34710
H	-1.56480	-3.04550	4.95990
H	-3.41740	-2.32550	3.45110
H	-3.03610	-2.13020	1.00830
H	1.06140	-3.39730	1.55290
H	0.67730	-3.58070	4.00110

SbF_6 anion free

SCF = –839.01840

ZPE = –839.00486

Δ*G* = –839.03552

Sb	0.00000	0.00000	0.00000
F	0.00000	0.00000	1.92110
F	0.00000	1.92110	0.00000
F	0.00000	0.00000	-1.92110
F	0.00000	-1.92110	0.00000
F	-1.92110	0.00000	0.00000
F	1.92110	0.00000	0.00000

(NMe_2-NHC)Au(C_6H_5I) η^2 intermediate

SCF = –1487.51178

ZPE = –1487.08771

Δ*G* = –1487.14879

N	1.44420	1.97240	0.00270
C	2.59300	2.72010	0.01150
C	1.69500	0.64680	0.04210
C	3.63480	1.82970	0.05560
N	3.05300	0.55020	0.09810
C	3.85320	-0.60900	0.15350
C	5.20360	-0.44970	0.05890
C	5.81400	0.84380	-0.03770
C	5.05310	1.96960	-0.00870
H	5.83520	-1.33810	0.10130
H	6.90290	0.91060	-0.10330
H	5.48050	2.97280	-0.05520
H	2.58440	3.80600	-0.02930
C	-2.43970	3.53450	-0.24000
C	-1.71840	3.28840	-1.40670
C	-0.41930	2.77500	-1.34870
C	0.11690	2.52120	-0.07810
C	-0.58330	2.75280	1.11390
C	-1.87850	3.26860	1.00700
H	-3.45480	3.93640	-0.30360
H	-2.16780	3.49510	-2.38250
H	-2.45270	3.46150	1.91780
C	0.02180	2.42360	2.45170
H	1.04150	2.83320	2.55290
C	0.36660	2.47910	-2.59750
H	-0.19980	2.77700	-3.49370
N	3.18510	-1.82170	0.29290
C	3.80430	-2.97430	-0.33360
H	3.06370	-3.79040	-0.37530
H	4.10610	-2.72650	-1.36320
H	4.69050	-3.34720	0.22170

C	2.70770	-2.10440	1.64320
H	1.94970	-2.90460	1.60770
H	3.53890	-2.43300	2.30190
H	2.24380	-1.21250	2.09190
Au	0.22050	-0.73520	-0.13030
I	-1.88600	-2.31290	-0.31070
C	-5.09470	1.23270	0.71310
C	-4.38290	0.61940	1.74470
C	-3.45950	-0.38850	1.46250
C	-3.26760	-0.76540	0.13430
C	-3.96100	-0.16000	-0.91150
C	-4.88150	0.84570	-0.60990
H	-5.81650	2.02190	0.94170
H	-4.54350	0.92370	2.78280
H	-2.90320	-0.86970	2.27080
H	-3.79340	-0.46120	-1.94840
H	-5.43170	1.32940	-1.42190
H	0.09270	1.32920	2.59230
H	0.59150	1.40020	-2.67850
H	1.33130	3.01570	-2.60830
H	-0.59520	2.82930	3.26890

(NMe$_2$-NHC)Au(C$_6$H$_5$I) transition state

SCF = –1487.48020

ZPE = –1487.05764

Δ*G* = –1487.11685

Au	0.05540	-0.73300	0.09840
I	-1.26890	-2.97810	0.14010
N	0.99350	2.23220	0.05520
C	2.09660	3.01360	-0.19200
C	1.29480	0.92050	0.07320
C	3.15660	2.15450	-0.34220
N	2.63360	0.86970	-0.14760
C	3.44040	-0.27680	-0.20520
C	4.74140	-0.11730	-0.56340
C	5.29440	1.18290	-0.82370
C	4.53410	2.30240	-0.68850
H	5.38260	-0.99910	-0.61170
H	6.34790	1.26090	-1.10350
H	4.93220	3.30550	-0.85220
H	2.04140	4.09730	-0.25290
C	-2.81630	3.87260	0.65090
C	-2.35890	3.63780	-0.64360
C	-1.10110	3.07050	-0.86630
C	-0.32720	2.76140	0.26070
C	-0.76500	2.96370	1.57630
C	-2.03240	3.52960	1.74990
H	-3.80330	4.31810	0.80520
H	-2.98830	3.89070	-1.50140
H	-2.40300	3.70600	2.76400
C	0.08600	2.56850	2.75330
H	0.14350	1.46860	2.84570
C	-0.59670	2.79390	-2.25670
H	-1.37660	3.01160	-3.00310
N	2.80540	-1.47690	0.14790
C	3.14590	-2.63570	-0.66130
H	2.42000	-3.43870	-0.44860
H	3.07720	-2.37870	-1.72980
H	4.16230	-3.02550	-0.44710
C	2.85530	-1.75150	1.58430
H	2.15820	-2.57170	1.82310
H	3.87590	-2.04460	1.90570
H	2.54920	-0.86060	2.15560
C	-4.29390	0.85010	-1.00500
C	-4.00290	0.60060	0.33700
C	-2.91560	-0.19700	0.69200
C	-2.12150	-0.72490	-0.32800
C	-2.40040	-0.50320	-1.67780
C	-3.49000	0.30550	-2.00580
H	-5.15230	1.47290	-1.27110
H	-4.62780	1.02790	1.12600
H	-2.69800	-0.40200	1.74230
H	-1.78860	-0.95230	-2.46320
H	-3.71180	0.49610	-3.05970
H	-0.33750	2.96400	3.68970
H	1.11970	2.94340	2.65730
H	0.28580	3.41290	-2.49800
H	-0.29750	1.73690	-2.37150

(NMe$_2$-NHC)Au(C$_6$H$_5$)I product

SCF = –1487.51909

ZPE = –1487.09258

Δ*G* = –1487.14964

N	1.55020	1.87800	-0.08400
C	1.57790	3.25060	-0.16410
C	0.28820	1.41440	-0.04650
C	0.27400	3.68200	-0.17200
N	-0.49220	2.51880	-0.09560
C	-1.87550	2.52930	-0.06760
C	-2.51250	3.72250	-0.11910
C	-1.76320	4.94690	-0.20510
C	-0.40220	4.93770	-0.23100
H	-3.60320	3.75220	-0.09220
H	-2.30940	5.89180	-0.24730
H	0.18620	5.85470	-0.29330
H	2.51340	3.80240	-0.20180

C	5.13430	-0.27780	0.13750
C	4.58490	0.27400	1.29270
C	3.37750	0.97580	1.24290
C	2.76120	1.10080	-0.00820
C	3.28480	0.56410	-1.18980
C	4.48840	-0.13980	-1.08910
H	6.07670	-0.83000	0.19450
H	5.09360	0.15360	2.25350
H	4.92340	-0.58290	-1.98950
C	2.58500	0.75040	-2.50770
H	1.53970	0.40040	-2.46550
C	2.75920	1.56720	2.48070
H	3.29920	1.23470	3.38070
N	-2.47960	1.22360	0.02950
C	-3.23010	1.09810	1.30460
H	-3.64180	0.08080	1.37190
H	-2.54640	1.27180	2.14900
H	-4.05550	1.83030	1.33700
C	-3.37310	0.97710	-1.12980
H	-3.77700	-0.04220	-1.05220
H	-4.20780	1.69910	-1.13430
H	-2.79520	1.07160	-2.06130
Au	-0.77510	-0.32140	0.01290
I	-2.30940	-2.47580	0.12680
C	2.95460	-3.35100	-0.13890
C	2.23490	-3.06490	-1.29910
C	1.15700	-2.17830	-1.25970
C	0.81210	-1.57560	-0.04980
C	1.52260	-1.85300	1.11590
C	2.59730	-2.74430	1.06470
H	3.79710	-4.04770	-0.17310
H	2.50560	-3.53930	-2.24710
H	0.59050	-1.97690	-2.17270
H	1.25350	-1.38390	2.06610
H	3.15880	-2.96010	1.97850
H	3.09820	0.19010	-3.30480
H	2.56400	1.81630	-2.79890
H	2.78870	2.67150	2.45850
H	1.70090	1.27120	2.58670

(NHC)Au(C_6H_5I) η^2 intermediate

SCF = –1353.68269

ZPE = –1353.33236

N	-2.03460	1.41140	0.02660
C	-3.31230	1.89450	-0.11460
C	-1.99020	0.06360	-0.03950
C	-4.12490	0.80020	-0.27800
N	-3.27830	-0.31720	-0.22850
C	-3.77130	-1.60710	-0.36270
C	-5.09920	-1.79230	-0.54410
C	-5.99930	-0.67010	-0.59820
C	-5.52720	0.59900	-0.46830
H	-5.48150	-2.80950	-0.64920
H	-7.06720	-0.84990	-0.74430
H	-6.18230	1.47170	-0.50390
H	-3.54030	2.95680	-0.08680
C	1.42870	3.69440	0.64780
C	0.65920	3.29220	1.73770
C	-0.50780	2.54470	1.55240
C	-0.86370	2.22200	0.23500
C	-0.11190	2.61050	-0.88300
C	1.04600	3.35850	-0.64890
H	2.34010	4.27640	0.81170
H	0.96580	3.55790	2.75350
H	1.65670	3.67480	-1.49910
C	-0.51020	2.20280	-2.27550
H	-1.57870	2.39640	-2.47150
C	-1.33500	2.07830	2.72020
H	-0.92060	2.45910	3.66680
Au	-0.28850	-1.01070	0.12010
I	2.10930	-2.12200	0.30460
C	4.41560	1.95970	-1.09310
C	3.73350	1.18810	-2.03510
C	3.07150	0.02270	-1.64590
C	3.10550	-0.34820	-0.30240
C	3.77370	0.41110	0.65500
C	4.43330	1.57240	0.24660
H	4.93310	2.87120	-1.40480
H	3.71330	1.49050	-3.08590
H	2.53830	-0.58170	-2.38420
H	3.78480	0.11160	1.70580
H	4.96180	2.17720	0.98900
H	-0.33830	1.12220	-2.43310
H	-1.35620	0.97500	2.77640
H	-2.38130	2.42190	2.64190
H	0.08220	2.75020	-3.02530
H	-3.02820	-2.40500	-0.31100

(NHC)Au(C_6H_5I) transition state

SCF = –1353.64576

ZPE = –1353.29642

N	-2.13970	1.04110	0.15240
C	-3.50120	1.12210	0.00080
C	-1.69000	-0.22240	0.02340
C	-3.94440	-0.15570	-0.23550
N	-2.80090	-0.96470	-0.21800
C	-2.88030	-2.33450	-0.42780
C	-4.08890	-2.89990	-0.65330

C	-5.28510	-2.10040	-0.67460
C	-5.21880	-0.75700	-0.47100
H	-4.14510	-3.97750	-0.81930
H	-6.24730	-2.58540	-0.85600
H	-6.10540	-0.11980	-0.47990
H	-4.03900	2.06380	0.07030
C	0.23820	4.42760	0.81840
C	-0.18770	3.66160	1.90090
C	-0.97170	2.51930	1.70600
C	-1.29730	2.18400	0.38540
C	-0.86500	2.91860	-0.72790
C	-0.09220	4.05680	-0.48320
H	0.84470	5.32140	0.99130
H	0.08350	3.95420	2.91950
H	0.25970	4.65390	-1.32920
C	-1.21440	2.48890	-2.12700
H	-2.29950	2.57360	-2.31650
C	-1.42750	1.67380	2.86520
H	-1.24780	2.19290	3.81960
Au	0.26710	-0.88540	0.14670
I	2.56290	-2.06720	0.35180
C	2.99270	2.62420	-1.55760
C	2.54290	1.62010	-2.41490
C	2.02010	0.43050	-1.90190
C	1.93110	0.30640	-0.51850
C	2.38660	1.27940	0.36660
C	2.91400	2.45240	-0.17540
H	3.41320	3.54530	-1.97000
H	2.60590	1.74740	-3.49930
H	1.69140	-0.36700	-2.57130
H	2.33860	1.13880	1.44810
H	3.26810	3.23490	0.50110
H	-0.93220	1.43650	-2.30640
H	-0.69130	3.11410	-2.86740
H	-2.50290	1.43470	2.80110
H	-0.87870	0.71480	2.89490
H	-1.93310	-2.87680	-0.39440

(NHC)Au(C_6H_5)I product

SCF = –1353.66350

ZPE = –1353.31204

N	-2.10500	-0.50140	-0.66180
C	-3.42180	-0.12420	-0.68030
C	-1.29740	0.54810	-0.42070
C	-3.45030	1.22600	-0.42070
N	-2.11500	1.61360	-0.25780
C	-1.76750	2.92480	0.03710
C	-2.74660	3.85040	0.16610
C	-4.12820	3.49230	-0.00490

C	-4.47740	2.20890	-0.29400
H	-2.46990	4.88040	0.39950
H	-4.89540	4.26330	0.09870
H	-5.51620	1.90260	-0.43100
H	-4.22600	-0.82880	-0.87420
C	-0.72080	-4.43730	-1.07060
C	-0.54100	-3.53300	-2.11520
C	-1.00210	-2.21690	-2.01070
C	-1.64040	-1.85540	-0.81650
C	-1.83830	-2.73840	0.25480
C	-1.36370	-4.04440	0.10140
H	-0.35410	-5.46270	-1.17110
H	-0.03750	-3.84890	-3.03340
H	-1.49540	-4.75810	0.91960
C	-2.52280	-2.29980	1.52150
H	-2.14580	-1.32330	1.87150
C	-0.79350	-1.22620	-3.12460
H	-0.50730	-1.74160	-4.05460
Au	0.72950	0.55320	-0.32380
I	3.33180	0.70680	-0.43490
C	0.25380	-1.21720	4.07660
C	-0.09380	0.11050	3.82870
C	0.03120	0.64840	2.54370
C	0.51840	-0.18210	1.54470
C	0.87490	-1.50780	1.75000
C	0.73570	-2.02020	3.04280
H	0.14740	-1.63010	5.08330
H	-0.47370	0.74370	4.63550
H	-0.25320	1.68500	2.34930
H	1.25110	-2.13920	0.94260
H	1.01090	-3.06160	3.23230
H	-2.36320	-3.03810	2.32250
H	-3.61260	-2.19860	1.37230
H	-1.70340	-0.63390	-3.32080
H	0.01650	-0.51470	-2.87480
H	-0.69910	3.12340	0.14440

(NMe_2-NHC)Au(4-OMe-C_6H_4I) η^2 intermediate

SCF = –1601.91500

ZPE = –1601.45809

N	1.33650	2.11040	0.05460
C	2.29910	3.05630	-0.18920
C	1.83720	0.85700	0.05120
C	3.47670	2.37600	-0.36340
N	3.17120	1.01590	-0.18060
C	4.17190	0.02760	-0.27460
C	5.41580	0.42360	-0.66730
C	5.73180	1.79850	-0.92210

C	4.79560	2.76750	-0.74200
H	6.20180	-0.32920	-0.74030
H	6.74800	2.05890	-1.22860
H	5.00580	3.82720	-0.89760
H	2.07540	4.11860	-0.23770
C	-2.71800	2.97410	0.69320
C	-2.24720	2.78500	-0.60430
C	-0.90040	2.49710	-0.84380
C	-0.05340	2.41130	0.27040
C	-0.49450	2.59360	1.58850
C	-1.85060	2.87850	1.77910
H	-3.77830	3.18480	0.85860
H	-2.93640	2.85330	-1.45110
H	-2.22840	3.02150	2.79560
C	0.45270	2.45550	2.75010
H	1.35820	3.07180	2.61430
C	-0.38480	2.25590	-2.23650
H	-1.17980	2.42380	-2.97980
N	3.79300	-1.27200	0.04190
C	4.48180	-2.33670	-0.66150
H	3.91680	-3.27320	-0.52030
H	4.51890	-2.11320	-1.73920
H	5.51560	-2.50480	-0.29300
C	3.65020	-1.53580	1.47060
H	3.06030	-2.45550	1.62020
H	4.64080	-1.66640	1.95470
H	3.12810	-0.70730	1.97320
Au	0.66050	-0.79510	0.08980
I	-1.09750	-2.76580	-0.01620
C	-4.83120	0.40370	-0.11350
C	-4.32640	-0.06220	1.11150
C	-3.28030	-0.97270	1.14400
C	-2.73840	-1.43100	-0.05960
C	-3.23460	-0.99290	-1.28160
C	-4.28770	-0.07620	-1.31180
O	-5.81410	1.31500	-0.03960
H	-4.75890	0.32050	2.03930
H	-2.88730	-1.31330	2.10510
H	-2.80890	-1.34920	-2.22300
H	-4.65980	0.26320	-2.27980
H	0.78500	1.40760	2.86470
H	-0.02550	1.21690	-2.35050
H	0.46060	2.92250	-2.48070
H	-0.03360	2.76150	3.68960
C	-6.33970	1.86250	-1.22750
H	-7.10590	2.58840	-0.91910
H	-5.56020	2.38690	-1.81010
H	-6.81210	1.08680	-1.85700

(NMe$_2$-NHC)Au(4-OMe-C$_6$H$_4$I) transition state

SCF = –1601.88329

ZPE = –1601.42708

N	-0.68260	2.37620	0.09120
C	-1.54800	3.42290	-0.11910
C	-1.29990	1.18290	0.00300
C	-2.78180	2.86880	-0.35150
N	-2.59810	1.48320	-0.25540
C	-3.66120	0.58240	-0.41660
C	-4.88000	1.08990	-0.73140
C	-5.09360	2.50380	-0.87350
C	-4.07540	3.38230	-0.67150
H	-5.71640	0.39840	-0.85120
H	-6.09370	2.86390	-1.12690
H	-4.20910	4.46250	-0.75390
H	-1.22440	4.46000	-0.08910
C	3.36430	3.02450	0.97270
C	2.44200	2.89800	2.00870
C	1.09050	2.65900	1.73860
C	0.71250	2.55540	0.39320
C	1.61900	2.65230	-0.67130
C	2.95810	2.89480	-0.35310
H	4.41800	3.20700	1.20100
H	2.77060	2.98570	3.04860
H	3.69320	2.97250	-1.15930
C	1.16520	2.47460	-2.09460
H	2.00890	2.59910	-2.79130
C	0.08470	2.49240	2.84590
H	0.52170	2.77670	3.81590
N	-3.34860	-0.77180	-0.19560
C	-3.70870	-1.23170	1.14370
H	-3.25220	-2.21960	1.32280
H	-3.32310	-0.52990	1.90040
H	-4.80790	-1.31990	1.26730
C	-3.76520	-1.68720	-1.24810
H	-3.27750	-2.66260	-1.08160
H	-4.86280	-1.84820	-1.26740
H	-3.44180	-1.30140	-2.22760
Au	-0.54270	-0.74100	0.07610
I	0.17040	-3.24030	0.26570
C	4.13090	-0.16220	-0.40100
C	3.37680	-0.55370	-1.51360
C	2.11270	-1.12150	-1.33750
C	1.59950	-1.26010	-0.05070
C	2.35540	-0.91100	1.07490
C	3.61350	-0.36080	0.89050
O	5.34180	0.41230	-0.46890
H	3.75220	-0.42530	-2.53030

H	1.53980	-1.43660	-2.21260
H	1.96520	-1.05350	2.08500
H	4.21610	-0.06000	1.75070
H	0.38360	3.20510	-2.36820
H	0.74030	1.46640	-2.24880
H	-0.81550	3.10880	2.67960
H	-0.24720	1.44060	2.91990
C	5.93160	0.63260	-1.72890
H	6.90200	1.11310	-1.53680
H	6.10060	-0.31770	-2.26800
H	5.31590	1.30450	-2.35490

(NMe$_2$-NHC)Au(4-OMe-C$_6$H$_4$)I product

SCF = –1601.92068

ZPE = –1601.46163

N	-0.00260	2.47000	-0.05590
C	-0.70920	3.63800	-0.22490
C	-0.81810	1.40080	-0.05970
C	-2.03570	3.29720	-0.33060
N	-2.06270	1.90730	-0.21680
C	-3.23380	1.16920	-0.25220
C	-4.40370	1.82800	-0.42480
C	-4.42160	3.25960	-0.56480
C	-3.27160	3.98620	-0.51730
H	-5.34040	1.26860	-0.44990
H	-5.38310	3.75830	-0.70640
H	-3.26370	5.07300	-0.61560
H	-0.21700	4.60650	-0.24740
C	4.14140	2.59730	0.54840
C	3.27780	2.72970	1.63360
C	1.89330	2.66330	1.45630
C	1.42420	2.47130	0.15070
C	2.26010	2.33730	-0.96300
C	3.63850	2.39570	-0.73480
H	5.22270	2.63920	0.70660
H	3.68070	2.87470	2.64000
H	4.32360	2.28090	-1.57990
C	1.69350	2.14950	-2.34340
H	0.94470	1.33990	-2.36790
C	0.94240	2.76440	2.61800
H	1.49330	2.89230	3.56270
N	-3.04920	-0.25120	-0.07850
C	-3.68600	-0.70300	1.18300
H	-3.48120	-1.77540	1.31560
H	-3.25830	-0.14380	2.02860
H	-4.77710	-0.54000	1.14360
C	-3.59710	-0.99360	-1.24010
H	-3.40030	-2.06550	-1.09700
H	-4.68590	-0.83290	-1.32240
H	-3.10010	-0.65130	-2.15990
Au	-0.78130	-0.62970	0.04550
I	-0.88930	-3.26550	0.21910
C	4.01720	-1.04930	-0.06740
C	3.26350	-1.20290	-1.23600
C	1.87090	-1.08410	-1.18740
C	1.23310	-0.80810	0.01630
C	1.97700	-0.67430	1.18970
C	3.36010	-0.79600	1.14590
O	5.36130	-1.12000	-0.01260
H	3.73970	-1.41410	-2.19520
H	1.30180	-1.21790	-2.11130
H	1.48970	-0.46950	2.14670
H	3.95830	-0.68190	2.05370
H	2.49040	1.89970	-3.06110
H	1.19420	3.07040	-2.69590
H	0.25190	3.61940	2.50830
H	0.32360	1.85330	2.70610
C	6.08070	-1.39230	-1.19010
H	7.14340	-1.41460	-0.90670
H	5.80360	-2.37320	-1.61880
H	5.93120	-0.60420	-1.95190

(NMe$_2$-NHC)Au(4-CF$_3$-C$_6$H$_4$I) η^2 intermediate

SCF = –1824.21500

ZPE = –1823.78629

N	-1.54020	2.11560	0.12870
C	-2.46880	3.12170	0.06020
C	-2.09390	0.89590	-0.03710
C	-3.68060	2.51710	-0.15510
N	-3.41970	1.13770	-0.23590
C	-4.46170	0.21320	-0.45410
C	-5.73620	0.69440	-0.48230
C	-6.02800	2.09170	-0.34920
C	-5.02260	2.99690	-0.21930
H	-6.55250	-0.00870	-0.65320
H	-7.06990	2.41950	-0.38490
H	-5.20190	4.07060	-0.13830
H	-2.20790	4.16950	0.18300
C	2.55350	2.67360	0.85620
C	1.66760	2.57280	1.92720
C	0.29790	2.39320	1.70870
C	-0.13770	2.31880	0.37750
C	0.72790	2.40950	-0.72130
C	2.08780	2.59170	-0.45390
H	3.62230	2.81150	1.04310
H	2.04080	2.63150	2.95380
H	2.79080	2.66210	-1.28770

C	0.22340	2.26460	-2.13160
H	-0.67370	2.88020	-2.31460
C	-0.67060	2.25470	2.85290
H	-0.14960	2.35120	3.81820
N	-4.08930	-1.11810	-0.61450
C	-5.01800	-2.11410	-0.11430
H	-4.49540	-3.08390	-0.06040
H	-5.35080	-1.84210	0.89970
H	-5.90930	-2.24420	-0.76350
C	-3.56590	-1.44640	-1.93670
H	-3.02080	-2.40410	-1.89280
H	-4.38490	-1.53690	-2.68120
H	-2.86880	-0.66920	-2.28640
Au	-1.01750	-0.81120	0.15990
I	0.67380	-2.82690	0.41170
C	4.54830	0.03300	-0.38470
C	3.75560	-0.37580	-1.46050
C	2.65450	-1.19780	-1.24160
C	2.36170	-1.59540	0.06310
C	3.14340	-1.19900	1.14340
C	4.24730	-0.37830	0.91120
C	5.71800	0.94470	-0.65250
H	3.98970	-0.05030	-2.47780
H	2.03260	-1.51390	-2.08230
H	2.90290	-1.51200	2.16180
H	4.86170	-0.05680	1.75460
H	-0.05120	1.21420	-2.34190
H	-1.17220	1.27030	2.83150
H	-1.46070	3.02480	2.81390
H	0.99910	2.56150	-2.85490
F	6.32960	1.33330	0.46850
F	5.33440	2.05840	-1.29580
F	6.63910	0.35690	-1.42550

(NMe_2-NHC)Au(4-CF_3-C_6H_4I) transition state

SCF = –1824.18425

ZPE = –1823.75656

N	-0.94350	2.38960	0.06390
C	-1.82930	3.43870	0.01060
C	-1.56130	1.19940	-0.05560
C	-3.07750	2.88910	-0.14330
N	-2.87680	1.50400	-0.19510
C	-3.94430	0.60540	-0.33430
C	-5.20270	1.11390	-0.36210
C	-5.44370	2.52820	-0.28440
C	-4.40790	3.40510	-0.19700
H	-6.04230	0.42480	-0.47140
H	-6.47420	2.89020	-0.31470
H	-4.55780	4.48520	-0.14960
H	-1.51130	4.47450	0.09580
C	3.16290	3.03170	0.61300
C	2.32380	2.93340	1.72050
C	0.95500	2.69390	1.56260
C	0.47220	2.56310	0.25330
C	1.29380	2.62460	-0.88030
C	2.65480	2.86790	-0.67350
H	4.23040	3.22040	0.75390
H	2.73280	3.04230	2.72920
H	3.32340	2.92290	-1.53650
C	0.73500	2.40870	-2.26020
H	0.36030	1.37550	-2.37520
C	0.03720	2.56040	2.74810
H	-0.76210	3.32200	2.73430
N	-3.58080	-0.74810	-0.45000
C	-4.34220	-1.67380	0.37620
H	-3.81990	-2.64560	0.38770
H	-4.39370	-1.29470	1.40860
H	-5.37190	-1.83880	-0.00110
C	-3.45850	-1.18830	-1.83980
H	-2.98240	-2.18230	-1.86680
H	-4.44860	-1.25530	-2.33530
H	-2.82740	-0.48470	-2.40660
Au	-0.84970	-0.73100	0.09440
I	-0.16960	-3.23720	0.37260
C	3.85240	-0.33430	-0.27900
C	3.11640	-0.72820	-1.39920
C	1.83630	-1.25110	-1.24890
C	1.29470	-1.34500	0.03810
C	2.02200	-0.96410	1.16810
C	3.30510	-0.45060	0.99850
C	5.25080	0.18880	-0.47020
H	3.53970	-0.63870	-2.40330
H	1.27330	-1.57830	-2.12530
H	1.60090	-1.06080	2.17070
H	3.87380	-0.14210	1.87820
H	1.51000	2.57480	-3.02480
H	-0.10910	3.08820	-2.47190
H	-0.45360	1.57050	2.75980
H	0.59790	2.67410	3.68900
F	6.11080	-0.79580	-0.76900
F	5.72130	0.79860	0.62150
F	5.31990	1.07080	-1.47720

(NMe_2-NHC)Au(4-CF_3-C_6H_4)I product

SCF = –1824.22381

ZPE = –1823.75643

N	0.56260	2.50200	0.08190

C	1.40990	3.58090	0.17750
C	1.24530	1.34360	0.05050
C	2.69170	3.08800	0.20200
N	2.54810	1.70300	0.11920
C	3.62500	0.83440	0.10370
C	4.87320	1.35330	0.17460
C	5.06840	2.77500	0.26790
C	4.00960	3.63050	0.28150
H	5.73590	0.68500	0.15780
H	6.08910	3.15950	0.32580
H	4.13480	4.71250	0.34900
H	1.03560	4.60050	0.21310
C	-3.57250	3.10710	-0.19640
C	-2.78140	3.18930	-1.34020
C	-1.40360	2.96660	-1.27150
C	-0.86550	2.66870	-0.01360
C	-1.62730	2.58170	1.15700
C	-3.00290	2.80050	1.03750
H	-4.65050	3.27750	-0.26810
H	-3.23750	3.42170	-2.30680
H	-3.63290	2.73070	1.92880
C	-0.98680	2.27810	2.48320
H	-0.39950	1.34490	2.44640
C	-0.53300	3.03600	-2.49690
H	-1.14860	3.12400	-3.40540
N	3.26610	-0.55810	-0.00290
C	3.78510	-1.12690	-1.27260
H	3.45970	-2.17440	-1.34780
H	3.37840	-0.55650	-2.12100
H	4.88800	-1.08360	-1.28920
C	3.78450	-1.31880	1.16150
H	3.45190	-2.36300	1.07580
H	4.88750	-1.28950	1.18170
H	3.38420	-0.88240	2.08880
Au	0.96850	-0.67270	-0.01930
I	0.79130	-3.31120	-0.14410
C	-3.83830	-0.65240	0.06440
C	-3.11680	-0.89090	1.23420
C	-1.72070	-0.88830	1.21460
C	-1.05480	-0.63690	0.01480
C	-1.76360	-0.39740	-1.16030
C	-3.16040	-0.40580	-1.12900
C	-5.23810	-0.65860	0.08830
H	-3.64030	-1.08930	2.17420
H	-1.16790	-1.09450	2.13490
H	-1.24470	-0.20220	-2.10260
H	-3.71840	-0.21390	-2.05020
H	-1.75010	2.16750	3.26920
H	-0.29910	3.08820	2.78680
H	0.14370	3.90880	-2.46230
H	0.09940	2.13660	-2.59680
F	-5.67580	-0.92320	1.37000
F	-5.70810	-1.63330	-0.76830
F	-5.71030	0.57430	-0.31340

6. Crystallographic information

X-ray diffraction experiments on **2a**, **2d**, **2f** and **2i**, (Figures S4 to S7) were carried out at 100(2) K on a Bruker APEX II diffractometer using Mo-K_a radiation (l = 0.71073 Å). Intensities were integrated in SAINT[28] and absorption corrections based on equivalent reflections were applied using SADABS.[29]

The structures were solved using Superflip[30] and refined by full matrix least squares against F^2 in ShelXL[31] using Olex2.[32] All of the non-hydrogen atoms were refined anisotropically, while all of the hydrogen atoms were located geometrically and refined using a riding model. Crystal structure and refinement data are given in Tables S1 and S2. In all cases, the structures of the Au(III) complexes were determined to be the chloride adducts, rather than the expected Au(III) iodides. It is speculated that in the synthesis of these complexes, a rapid metathesis occurs with the initially formed iodide with AgCl, generated by reaction of the NHCAuCl complex and $AgSbF_6$. Similar exchanges have been reported in the literature where complete[24] or partial[33] iodide/chloride exchange is observed. The exchanges are likely to be driven by the relative stability of the Au(III) chloride complex over the analogous iodide.[34] Crystallographic data for compounds **2a**, **2d**, **2f** and **2i** have been deposited with the Cambridge Crystallographic Data Centre as a supplementary publication. CCDC deposition numbers: 2238560- 2238563. Copies of data can be obtained free of charge on application to the CCDC, 12 Union Road, Cambridge, CB2 1EZ, U.K. Fax: (+44) 1223 336033. Email: deposit@ccdc.cam.ac.uk.

Figure S4: Thermal ellipsoid plot of cation of **2a** determined by single-crystal X-ray diffraction. Thermal ellipsoids are shown at the 50% probability level with counterion and hydrogen atoms omitted for clarity. Selected bond lengths (Å) and bond angles (°); C1-Au1 2.007(2), N3-Au1 2.230(2), Cl1-Au1 2.3029(6), C22-Au1 2.026(3), C1-Au1-N3 80.13(9).

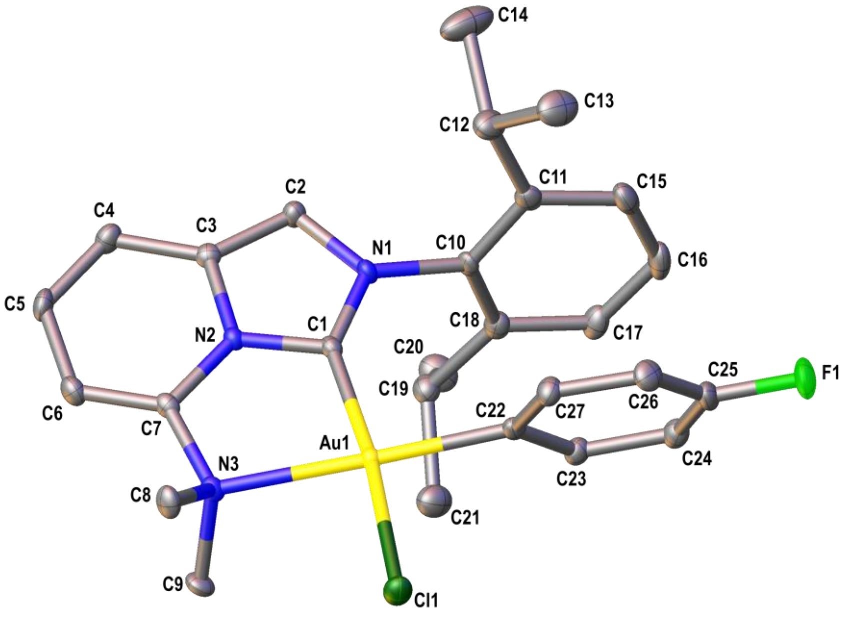

Figure S5: Thermal ellipsoid plot the grown structure of complex **2d** determined by single-crystal X-ray diffraction. Thermal ellipsoids are shown at the 50% probability level with counterion and hydrogen atoms omitted for clarity. Selected bond lengths (Å) and bond angles (°); C1-Au1 1.989(3), N3-Au1 2.221(3), Cl1-Au1 2.2804(9), C22-Au1 2.016(3), C1-Au1-N3 81.32(13).

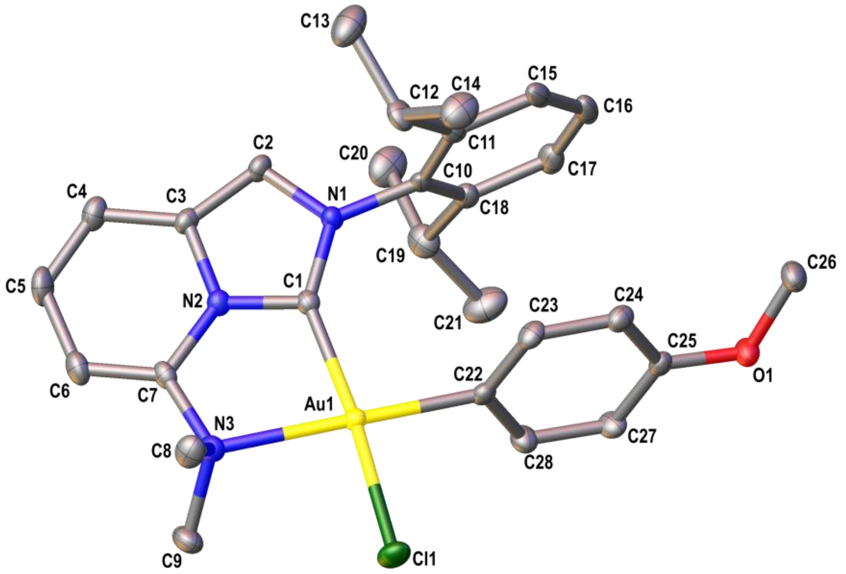

Table S1: Crystal data and structure refinement for **2a** and **2d**

Identification code	2a	2d
**Empirical formula**	$C_{27}H_{31}AuClF_7N_3Sb$	$C_{28}H_{34}AuClF_6N_3OSb$
**Formula weight**	884.71	896.75
**Temperature/K**	100(2)	100(2)
**Crystal system**	Monoclinic	orthorhombic
**Space group**	$P2_1/n$	$P2_12_12_1$
***a*/Å**	12.1579(16)	10.7343(2)
***b*/Å**	15.0557(17)	11.4659(2)
***c*/Å**	16.714(2)	24.9806(5)
**α/°**	90	90
**β/°**	107.979(6)	90
**γ/°**	90	90
**Volume/Å^3**	2910.0(6)	3074.57(10)
**Z**	4	4
**ρ_{calc}g/cm^3**	2.019	1.937
**μ/mm^{-1}**	6.123	5.795
**F(000)**	1696.0	1728.0
**Crystal size/mm^3**	0.443 × 0.324 × 0.06	0.372 × 0.337 × 0.14
**Radiation**	MoKα (λ = 0.71073)	MoKα (λ = 0.71073)
**2θ range for data collection/°**	3.726 to 59.144	3.26 to 59.148
**Index ranges**	$-15 \leq h \leq 16$, $-20 \leq k \leq 20$, $-23 \leq l \leq 18$	$-13 \leq h \leq 14$, $-15 \leq k \leq 15$, $-34 \leq l \leq 34$
**Reflections collected**	47081	50474
**R_{int} / R_{sigma}**	0.0396 / 0.0286	0.034 / 0.0235
**Data/restraints/parameters**	8150/371/431	8599/81/407
**Goodness-of-fit on F^2**	1.021	1.054
**Final R indexes [I>=2σ (I)]**	R_1 = 0.0208, wR_2 = 0.0443	R_1 = 0.0172, wR_2 = 0.0341
**Final R indexes [all data]**	R_1 = 0.0272, wR_2 = 0.0461	R_1 = 0.0187, wR_2 = 0.0345
**Largest diff. peak/hole / e Å^{-3}**	1.31/-0.71	0.76/-0.68

Figure S6: Thermal ellipsoid plot of cation of **2f** determined by single-crystal X-ray diffraction. Thermal ellipsoids are shown at the 50% probability level with counterion and hydrogen atoms omitted for clarity. Selected bond lengths (Å) and bond angles (°); C1-Au1 1.999(3), N3-Au1 2.205(2), Cl1-Au1 2.3021(7), C22-Au1 2.021(3), C1-Au1-N3 80.69(10).

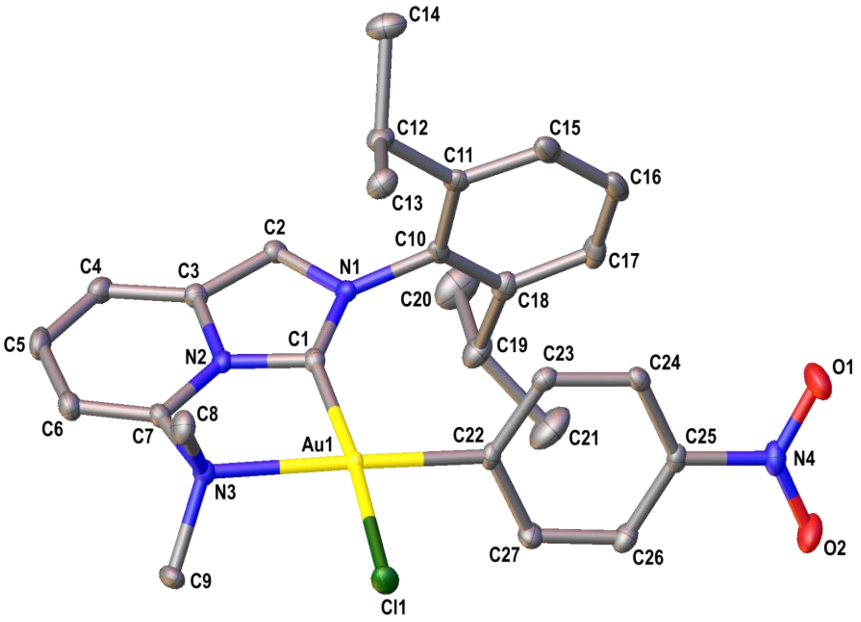

Figure S7: Thermal ellipsoid plot of cation of **2i** determined by single-crystal X-ray diffraction. Thermal ellipsoids are shown at the 50% probability level with counterion, solvent of crystallization and hydrogen atoms omitted for clarity. Selected bond lengths (Å) and bond angles (°); C1-Au1 1.999(11), N3-Au1 2.224(9), Cl1-Au1 2.292(3), C22-Au1 2.020(10), C1-Au1-N3 80.7(4).

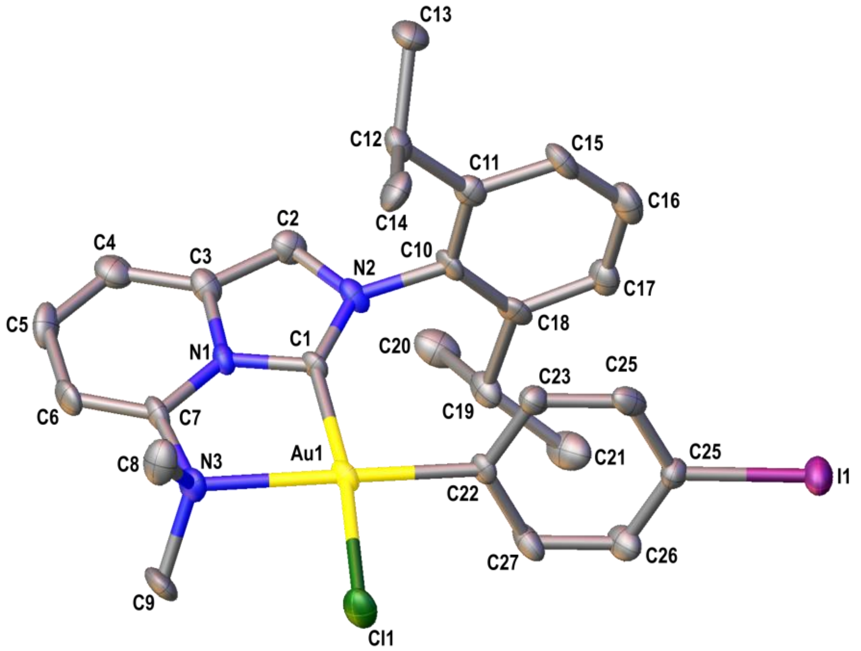

Table S2: Crystal data and structure refinement for **2f** and **2i**

**Identification code**	**2f**	**2i**
**Empirical formula**	$C_{27}H_{31}AuClF_6N_4O_2Sb$	$C_{27.5}H_{32}AuCl_2F_6IN_3Sb$
**Formula weight**	911.72	1035.08
**Temperature/K**	100(2)	100(2)
**Crystal system**	Monoclinic	monoclinic
**Space group**	$P2_1/n$	$P2_1$
***a*/Å**	12.8795(2)	17.1803(4)
***b*/Å**	14.6639(2)	10.5625(3)
***c*/Å**	16.9990(3)	18.9900(5)
**α/°**	90	90
**β/°**	109.0172(9)	108.7073(13)
**γ/°**	90	90
**Volume/Å^3**	3035.27(8)	2364.00(15)
**Z**	4	4
**ρ_{calc}g/cm^3**	1.995	2.106
**μ/mm^{-1}**	5.875	6.484
**F(000)**	1752.0	1956.0
**Crystal size/mm^3**	0.294 × 0.096 × 0.08	0.289 × 0.28 × 0.04
**Radiation**	MoKα (λ = 0.71073)	MoKα (λ = 0.71073)
**2θ range for data collection/°**	4.348 to 60.266	2.264 to 54.204
**Index ranges**	$-18 \leq h \leq 18$, $-20 \leq k \leq 20$, $-22 \leq l \leq 24$	$-22 \leq h \leq 22$, $-13 \leq k \leq 13$, $-24 \leq l \leq 24$
**Reflections collected**	50277	42552
**R_{int} / R_{sigma}**	0.0542 / 0.0393	0.0459 / 0.0590
**Data/restraints/parameters**	8937/324/449	14409/457/883
**Goodness-of-fit on F^2**	1.007	1.041
**Final R indexes [I>=2σ (I)]**	R_1 = 0.0254, wR_2 = 0.0463	R_1 = 0.0363, wR_2 = 0.0764
**Final R indexes [all data]**	R_1 = 0.0374, wR_2 = 0.0492	R_1 = 0.0422, wR_2 = 0.0786
**Largest diff. peak/hole / e Å^{-3}**	0.70/-0.61	1.83/-1.19

7. NMR spectra

S2: ^{1}H, $CDCl_3$, 400 MHz

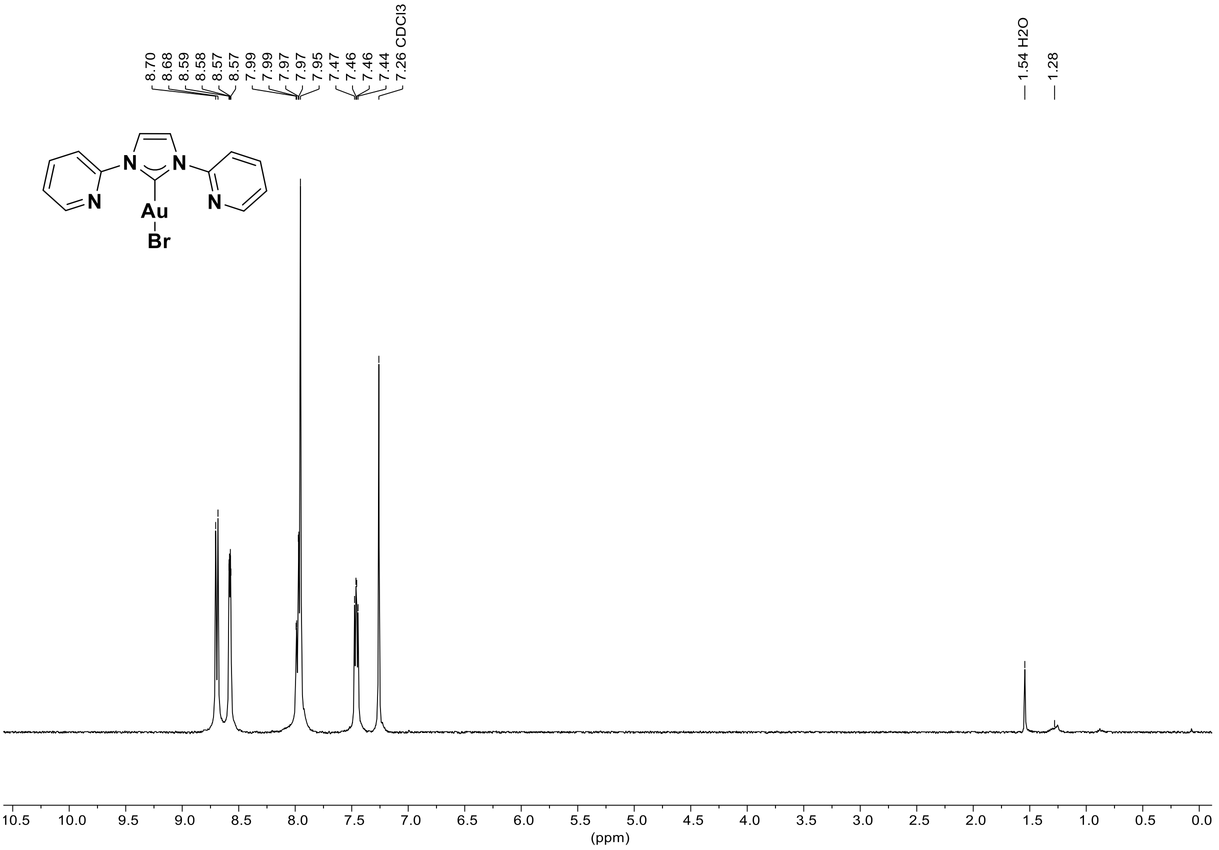

S2: ^{13}C, $CDCl_3$, 101 MHz

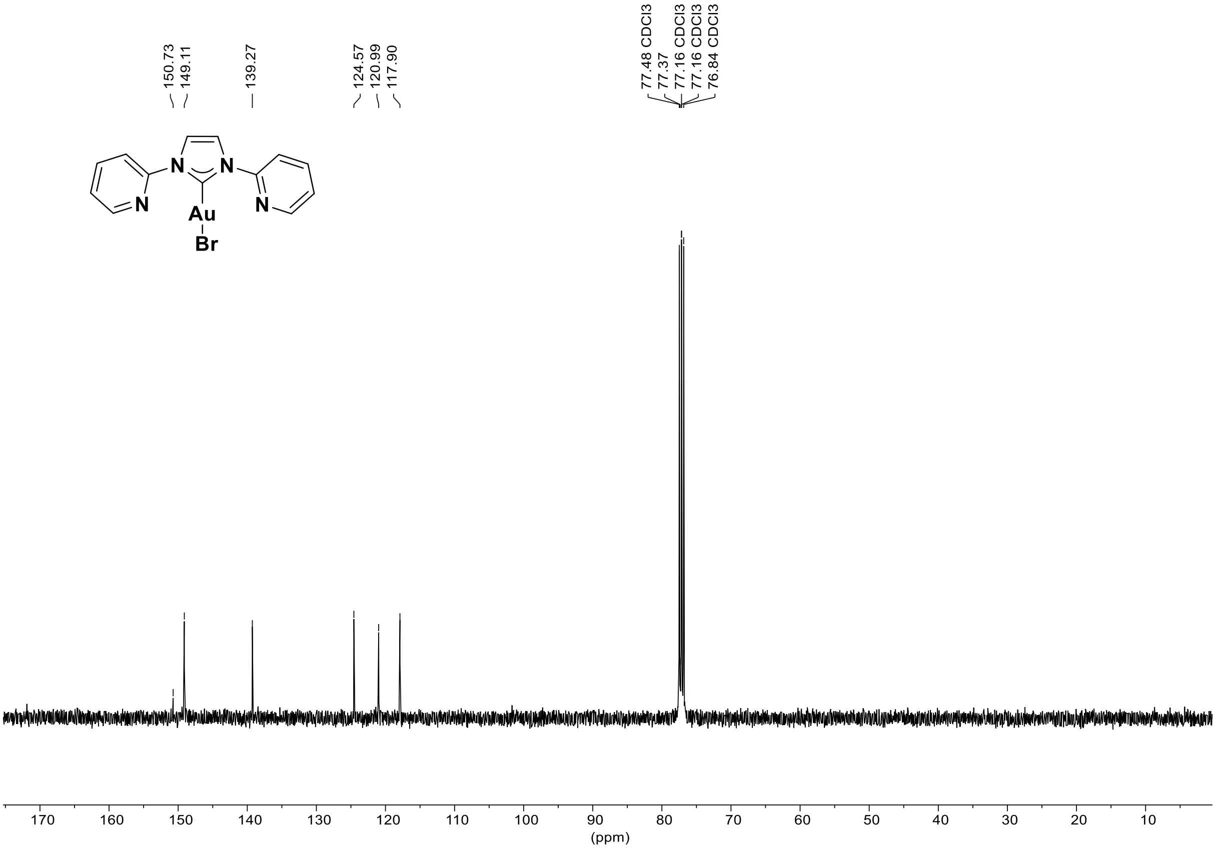

S4: ^{1}H, $CDCl_3$, 400 MHz

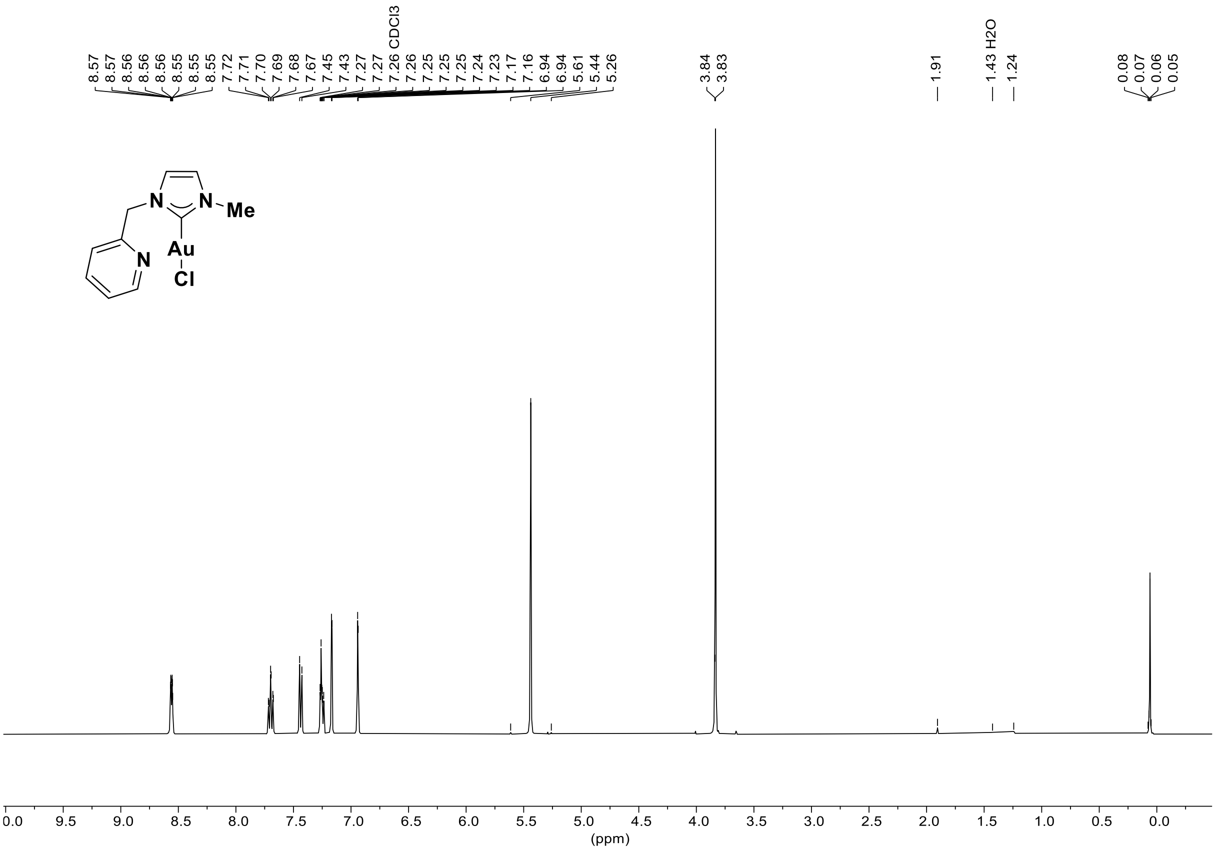

S4: ^{13}C, $CDCl_3$, 101 MHz

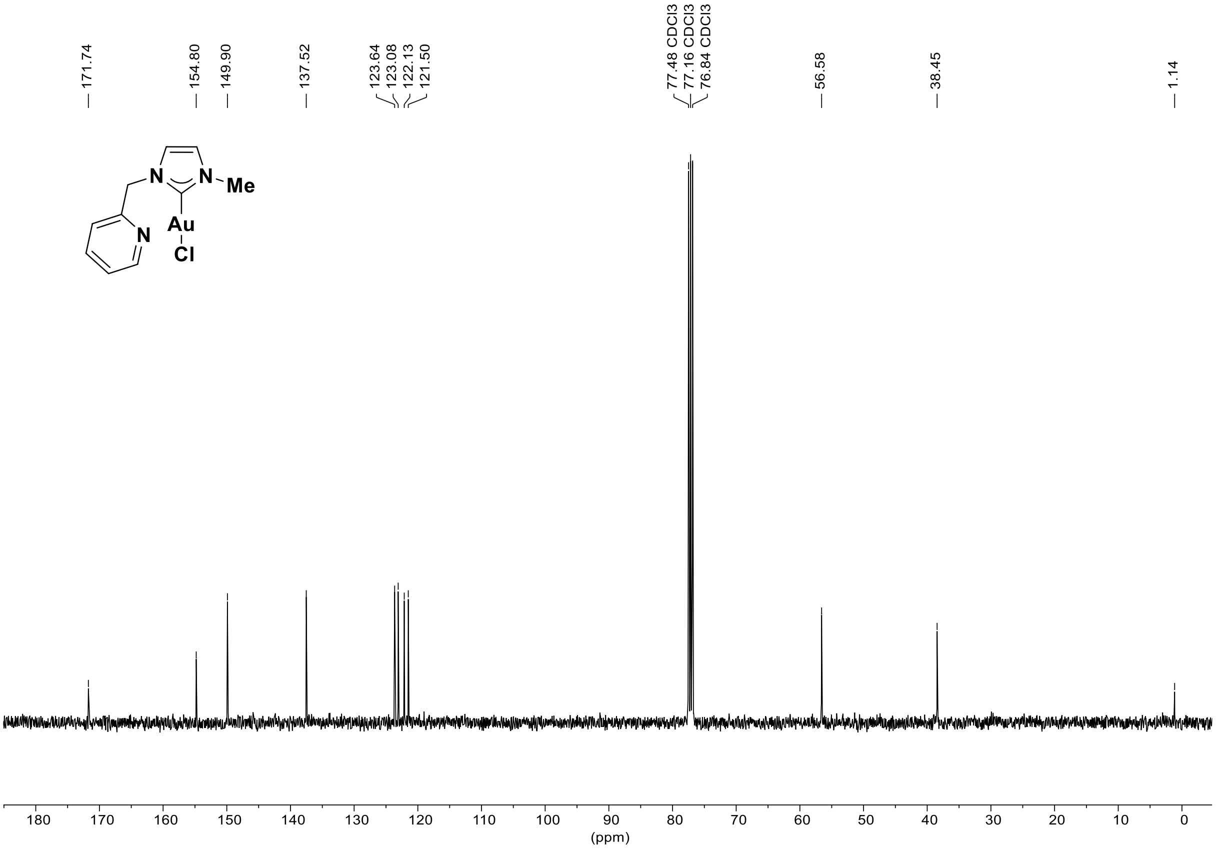

S5: ^{1}H, CD_2Cl_2, 500 MHz

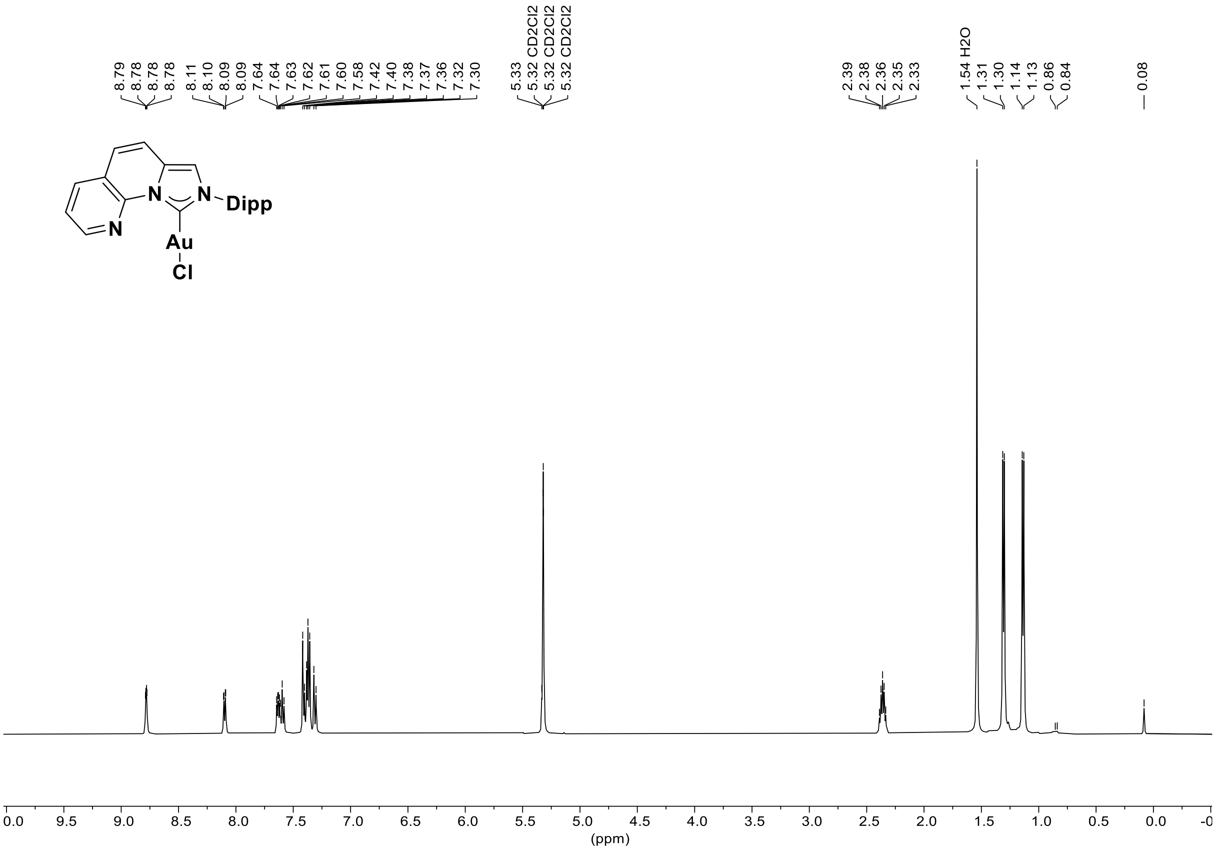

S5: ^{13}C, CD_2Cl_2, 126 MHz

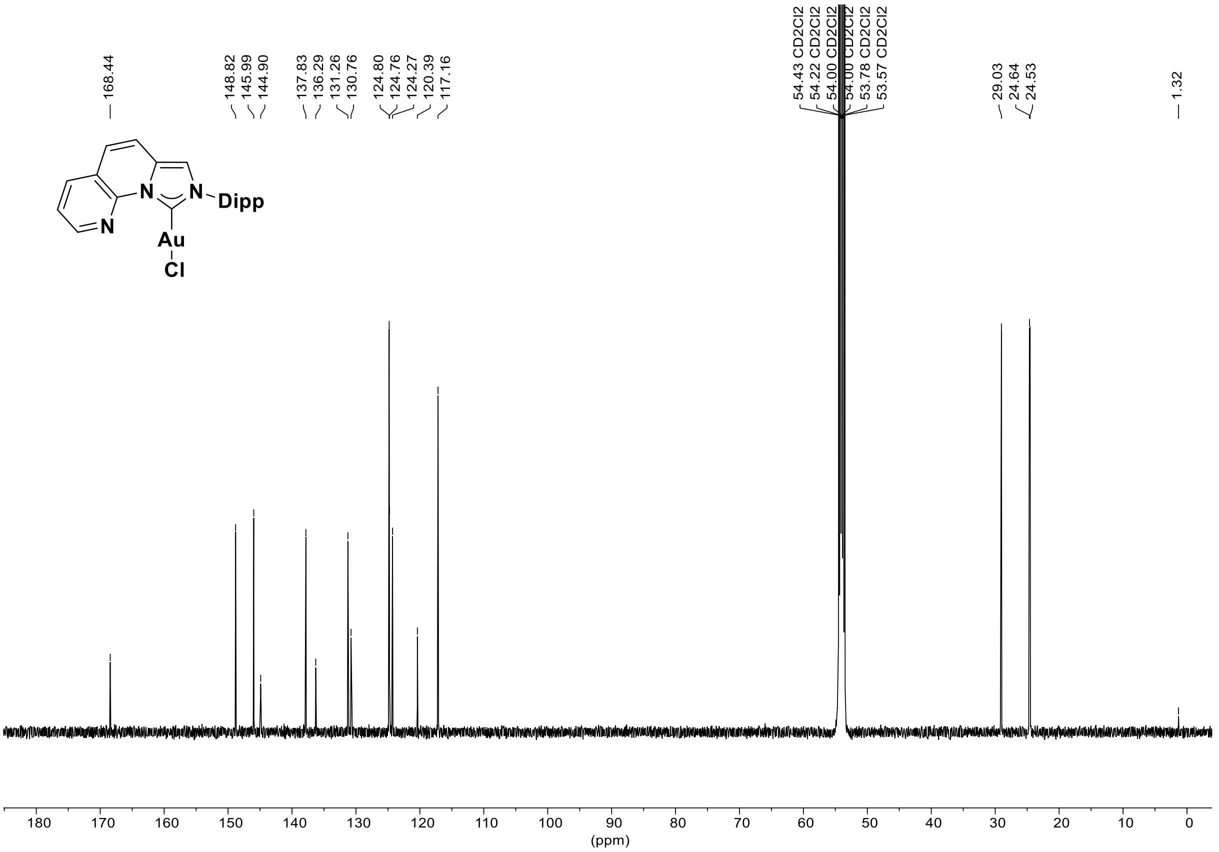

2a: ^{1}H, CD_2Cl_2, 500 MHz

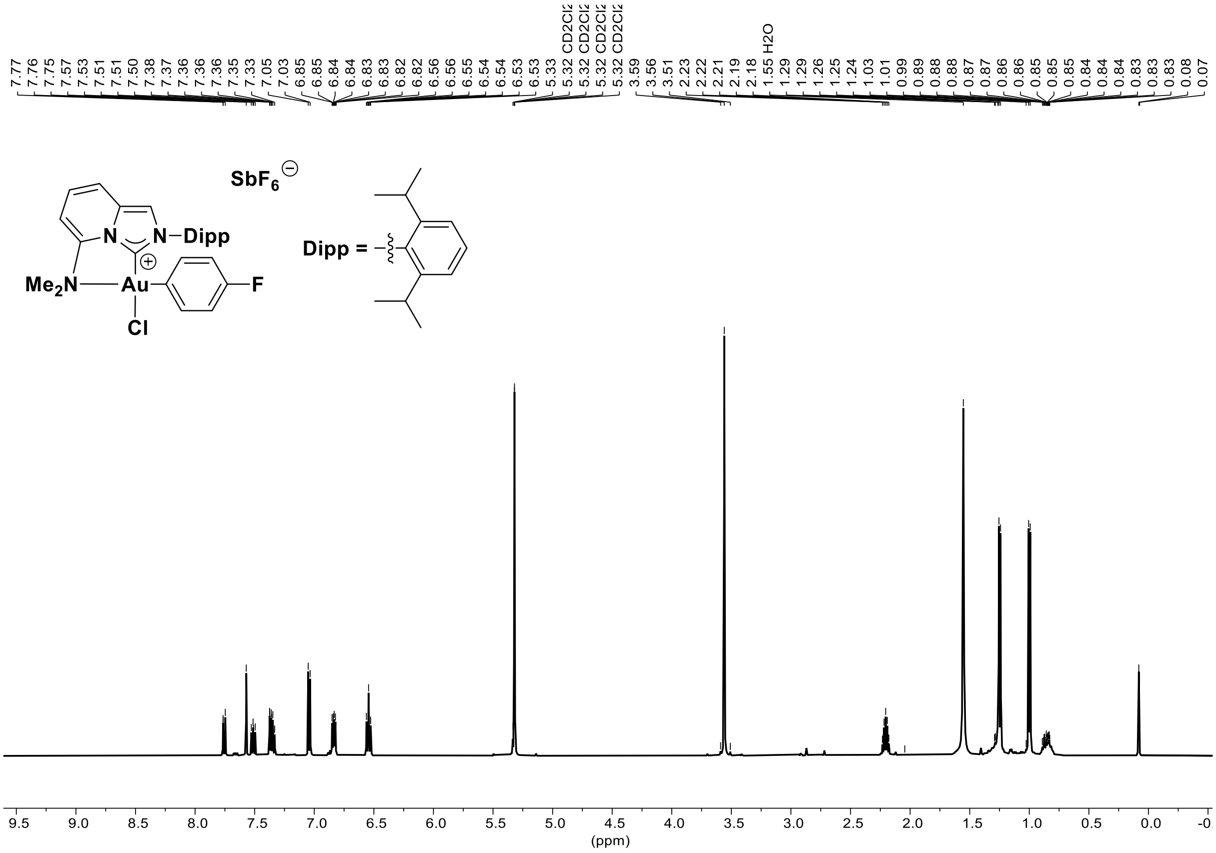

2a: ^{13}C, CD_2Cl_2, 126 MHz

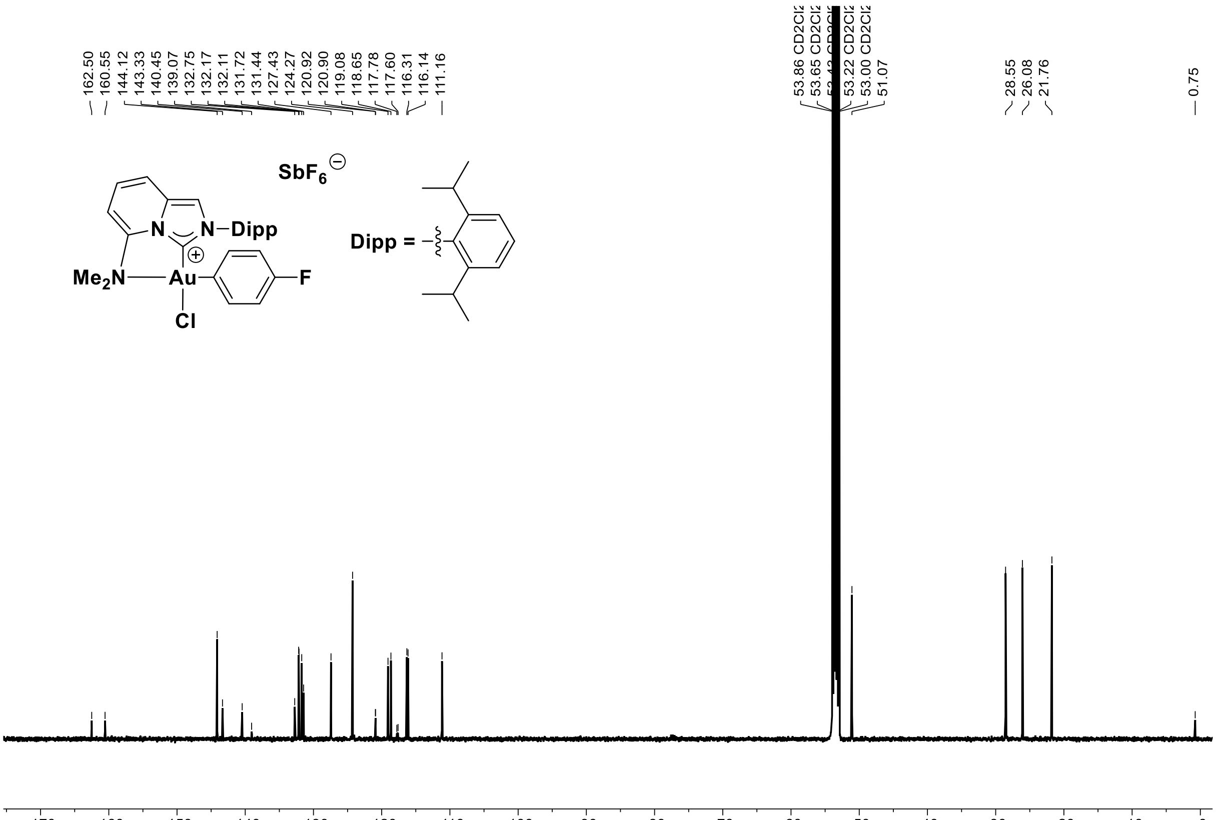

2a: ^{19}F, CD_2Cl_2, 377 MHz

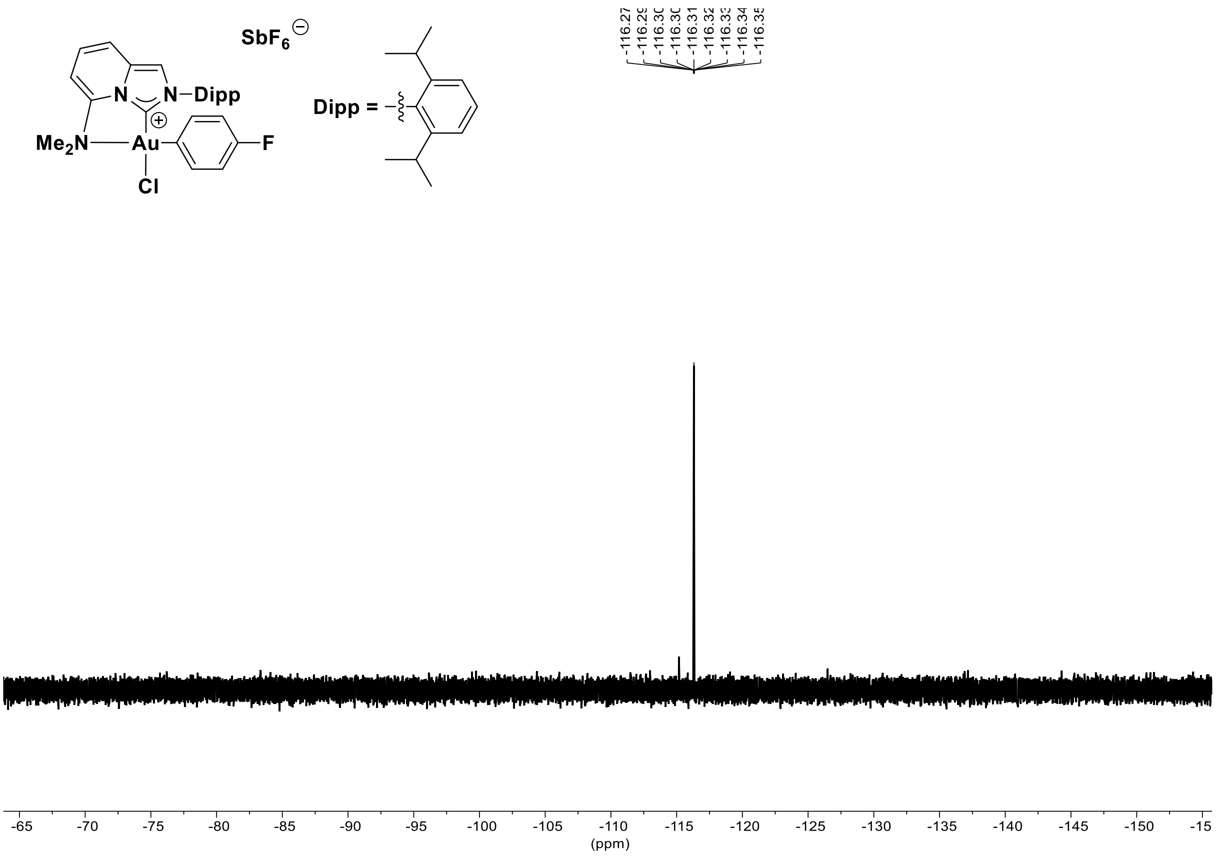

2b: ^{1}H, CD_2Cl_2, 500 MHz

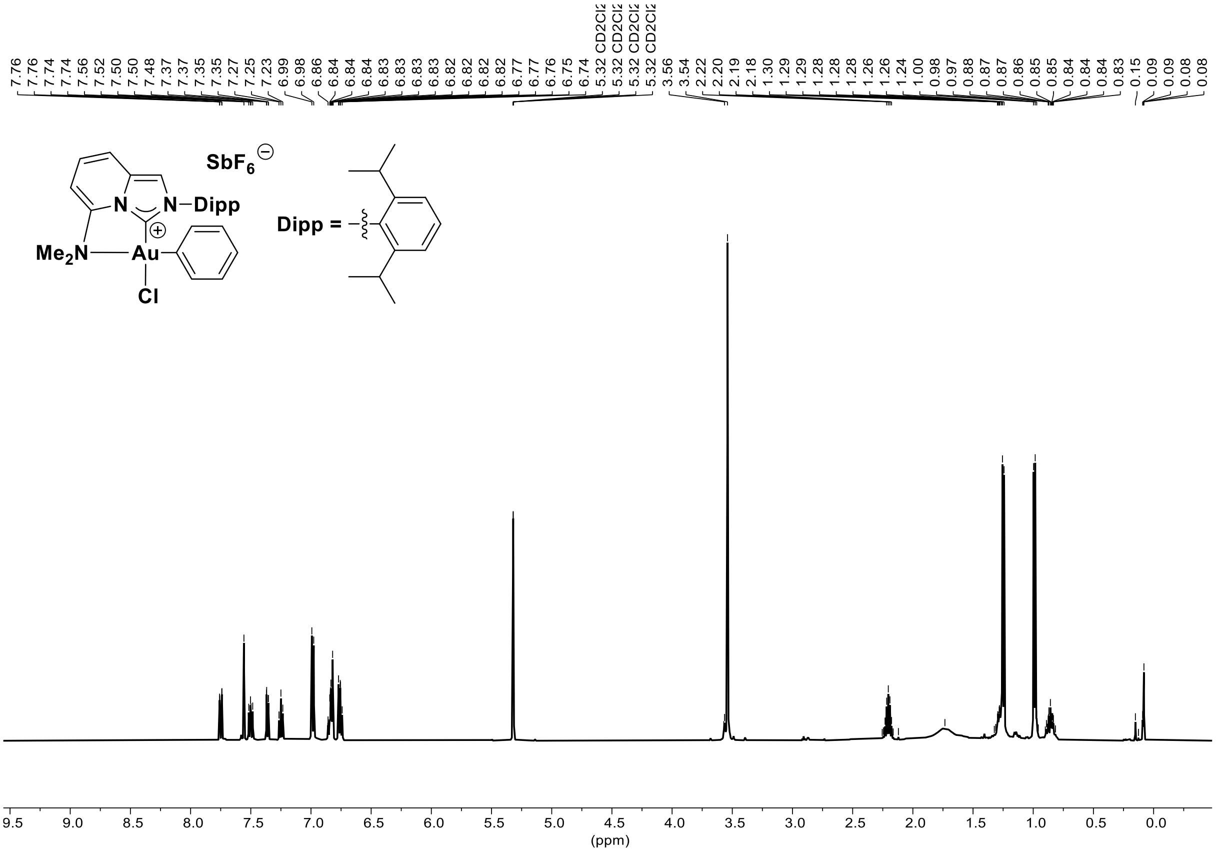

2b: ^{13}C, CD_2Cl_2, 126 MHz

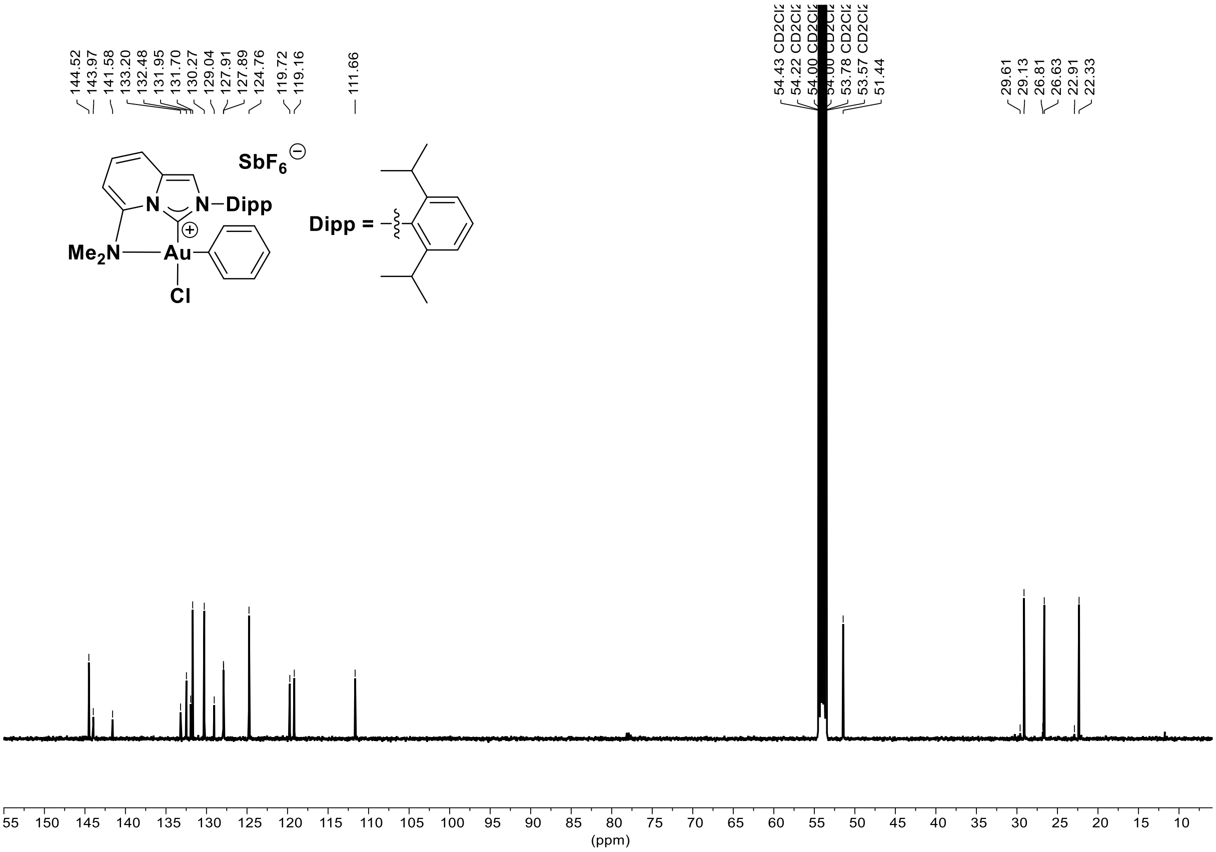

2c: ^{1}H, CD_2Cl_2, 500 MHz

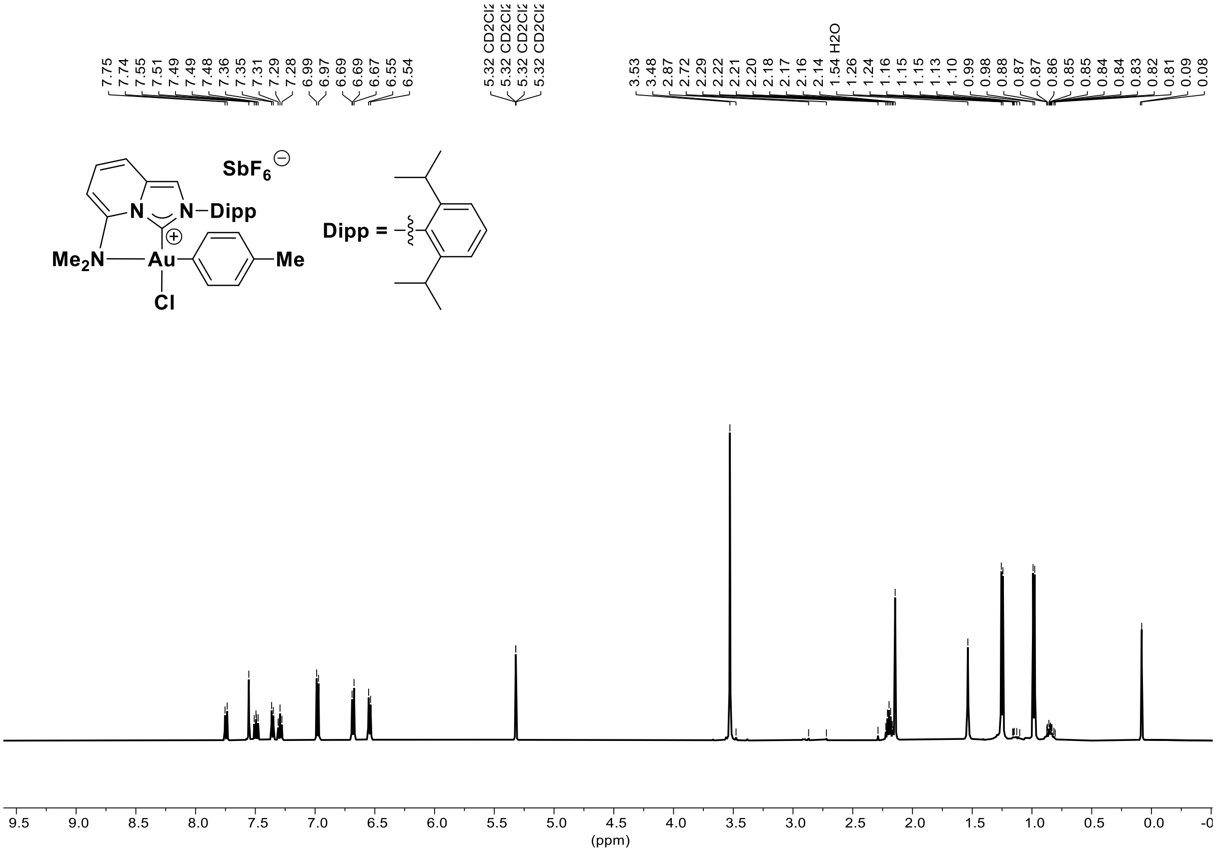

2c: ^{13}C, CD_2Cl_2, 126 MHz

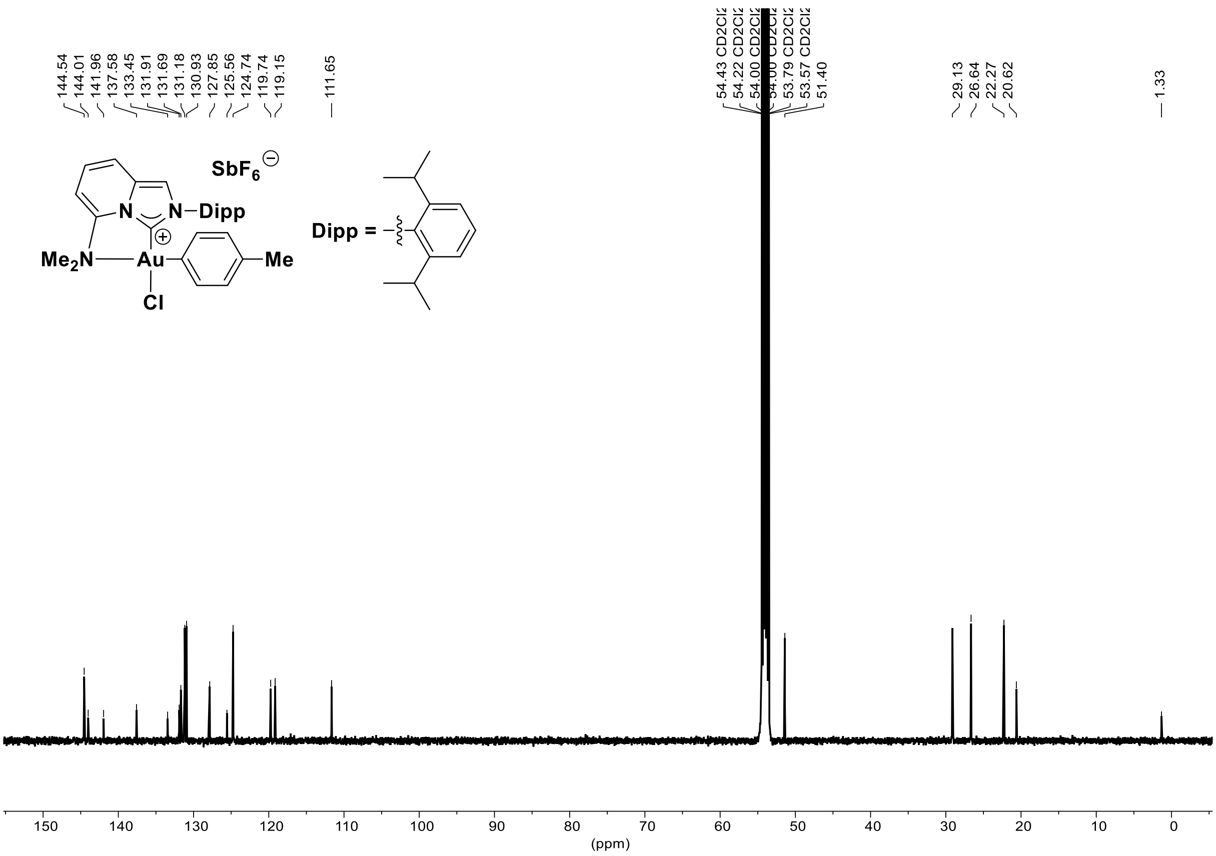

2d: ^{1}H, CD_2Cl_2, 500 MHz

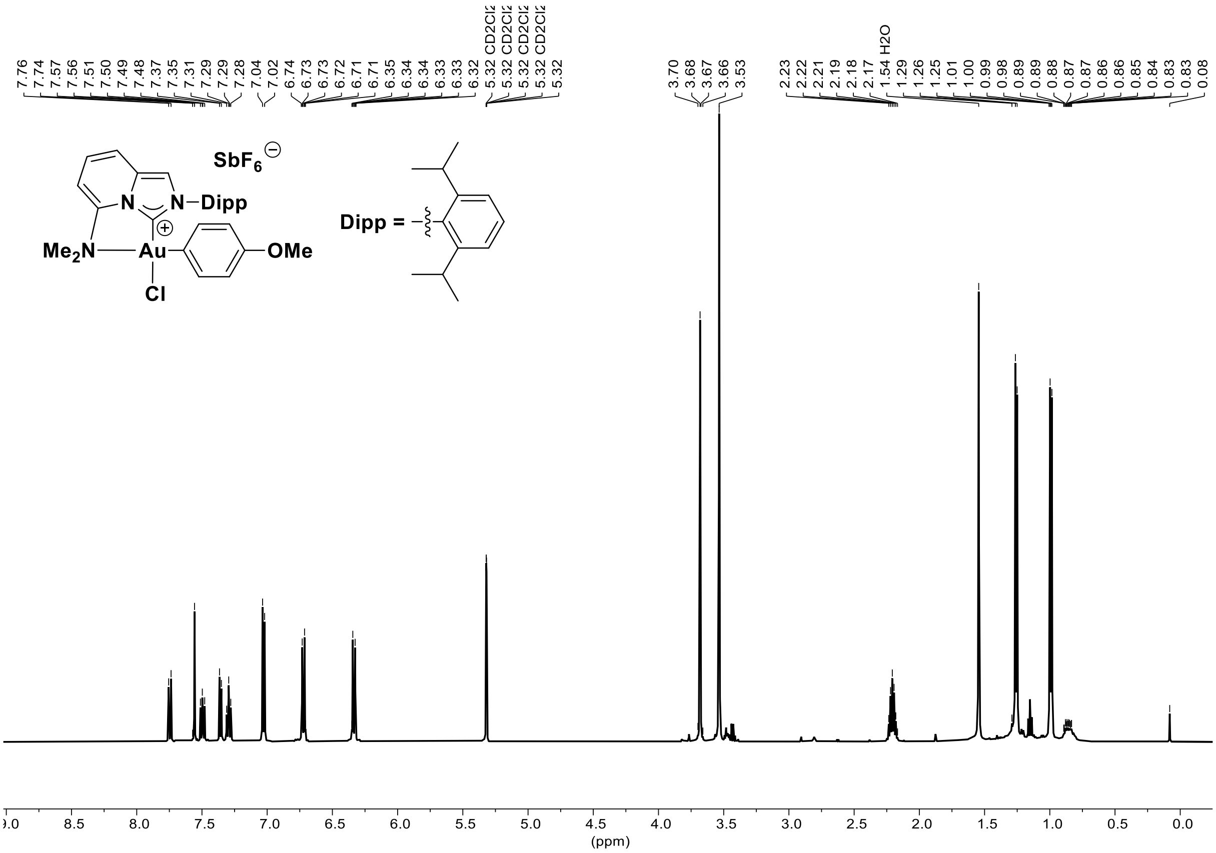

2d: ^{13}C, CD_2Cl_2, 126 MHz

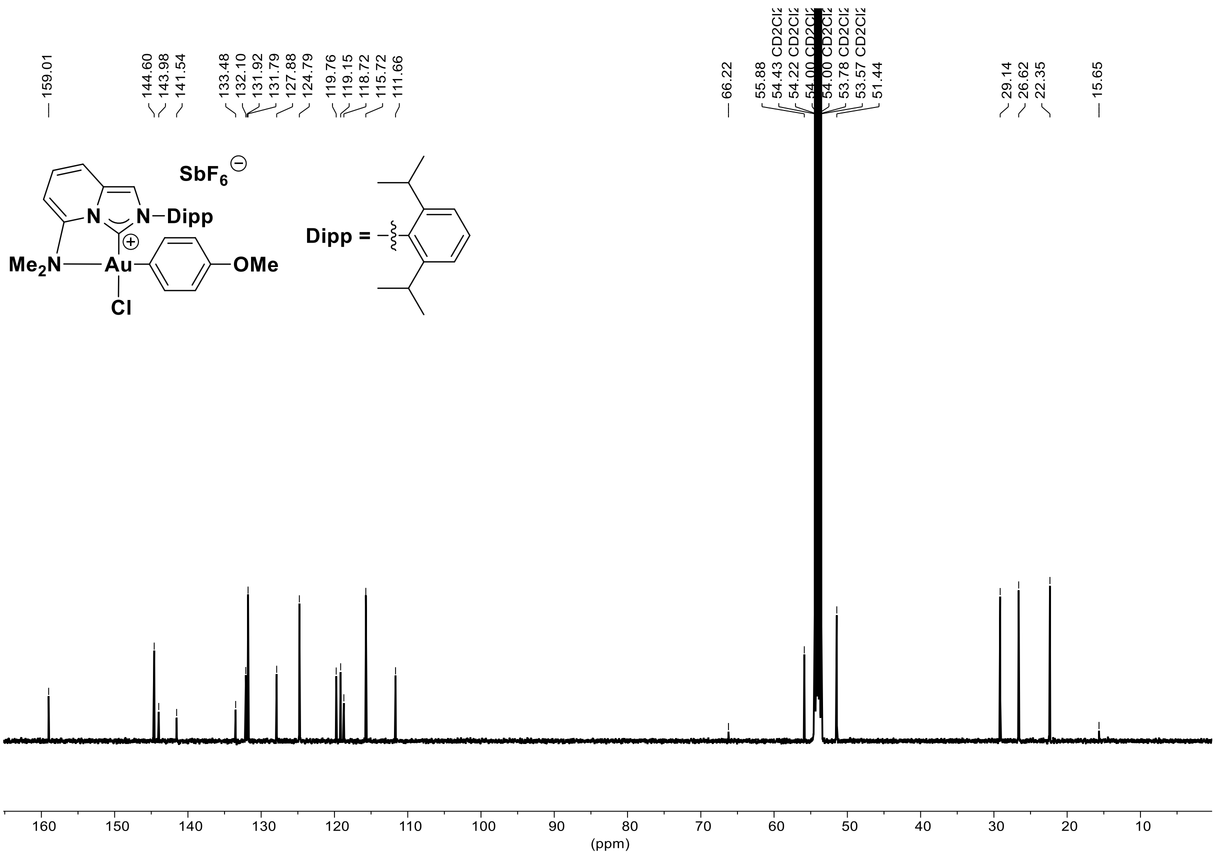

2e: ^{1}H, CD_2Cl_2, 500 MHz

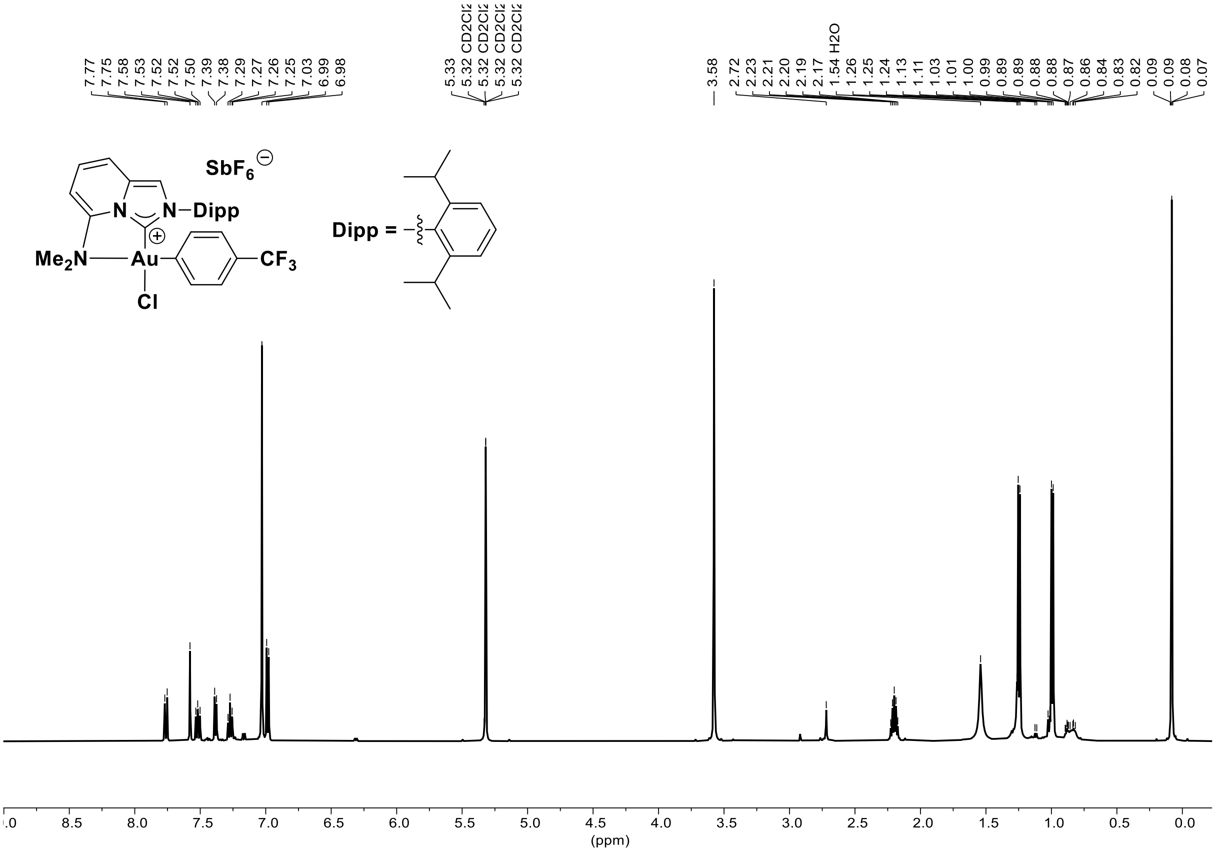

2e: ^{13}C, CD_2Cl_2, 126 MHz

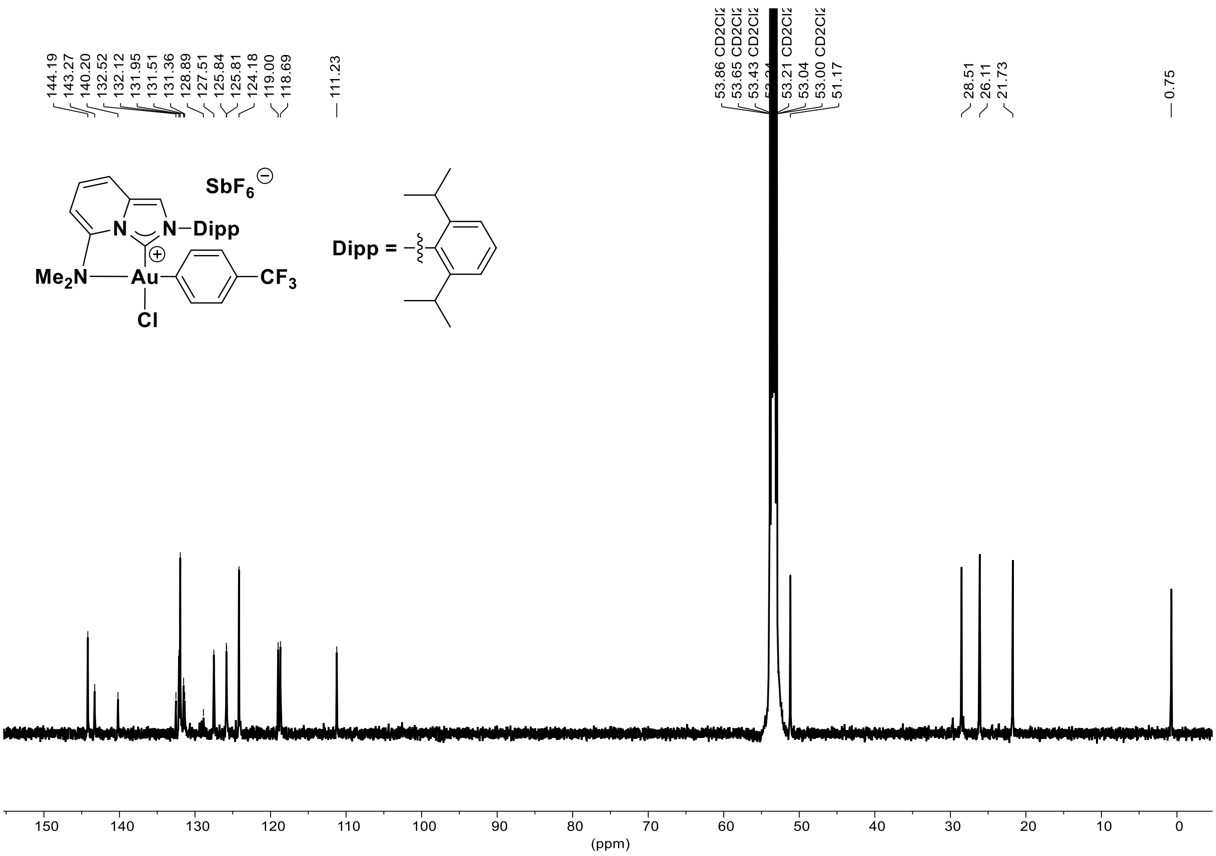

2e: ^{19}F, CD_2Cl_2, 377 MHz

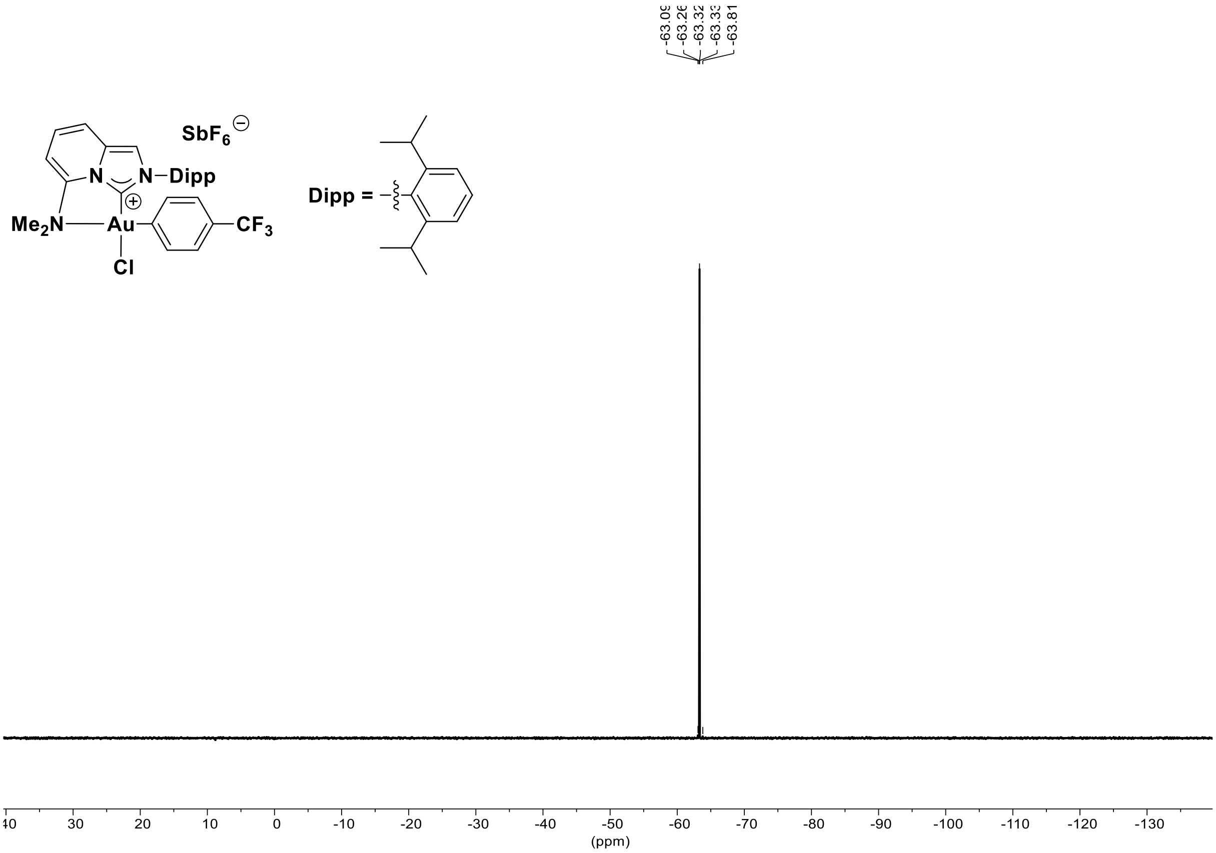

2f: ^{1}H, CD_2Cl_2, 500 MHz

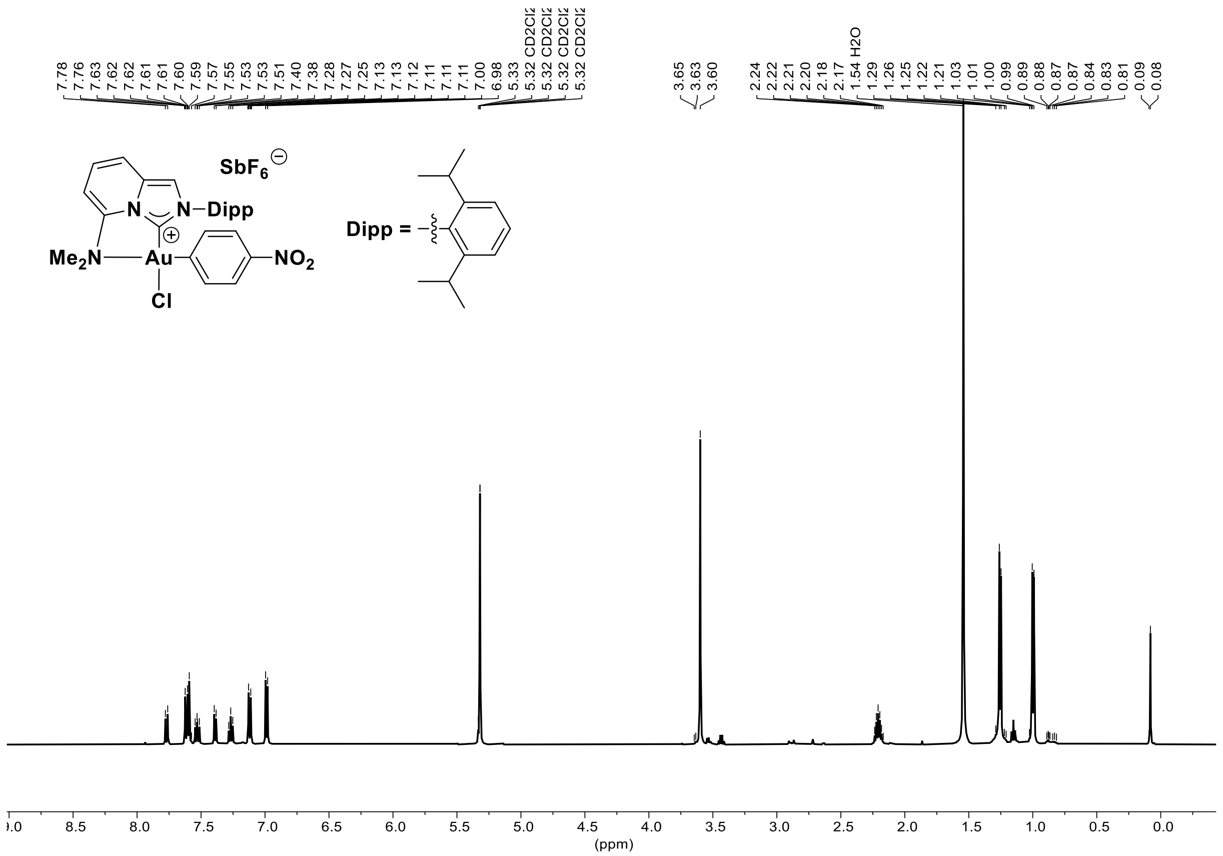

2f: ^{13}C, CD_2Cl_2, 126 MHz

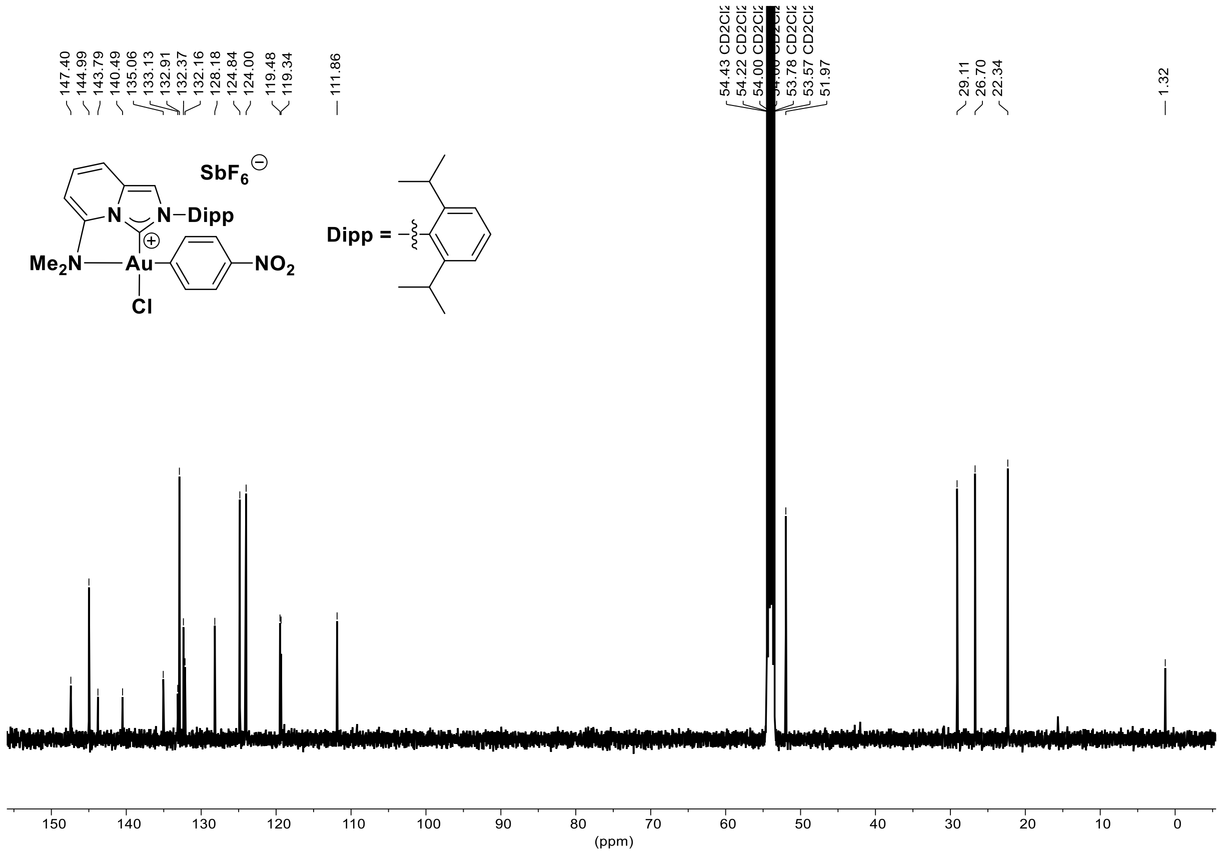

2g: ^{1}H, CD_2Cl_2, 500 MHz

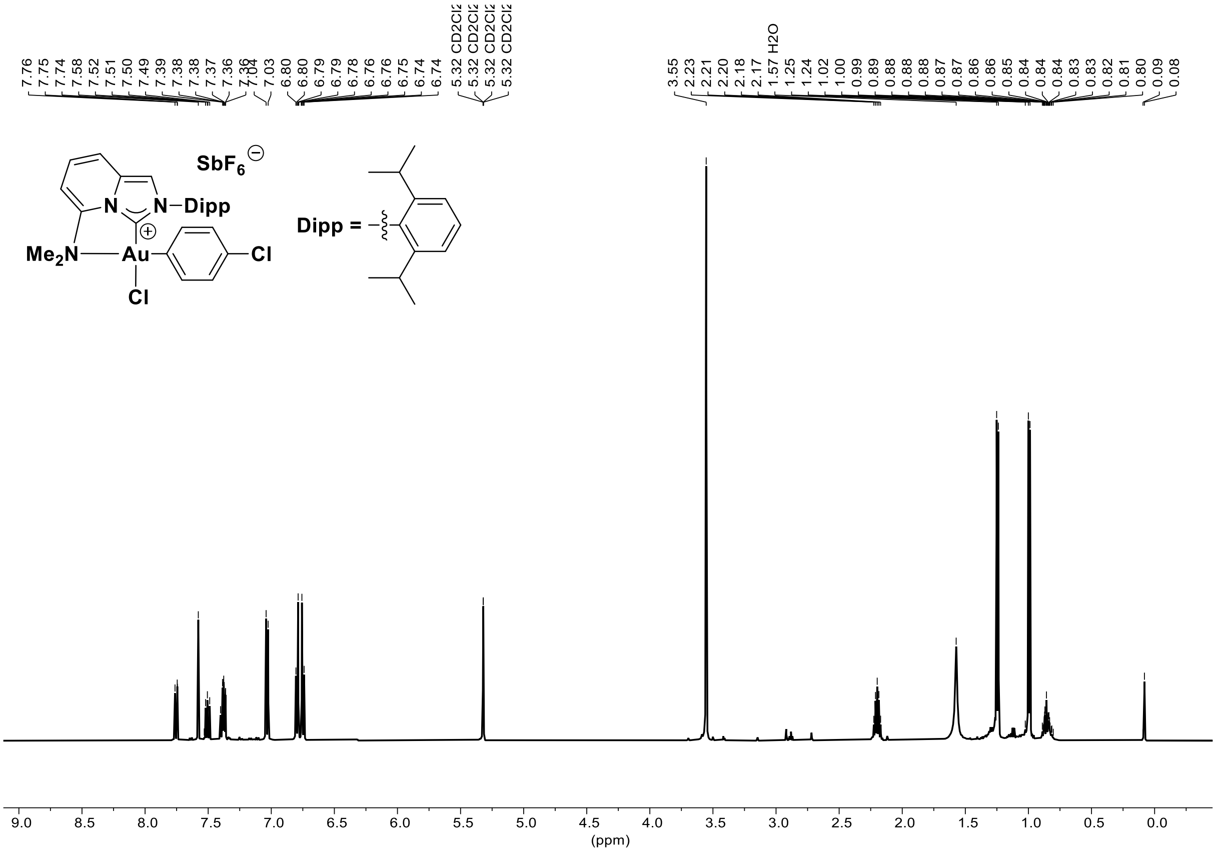

2g: ^{13}C, CD_2Cl_2, 126 MHz

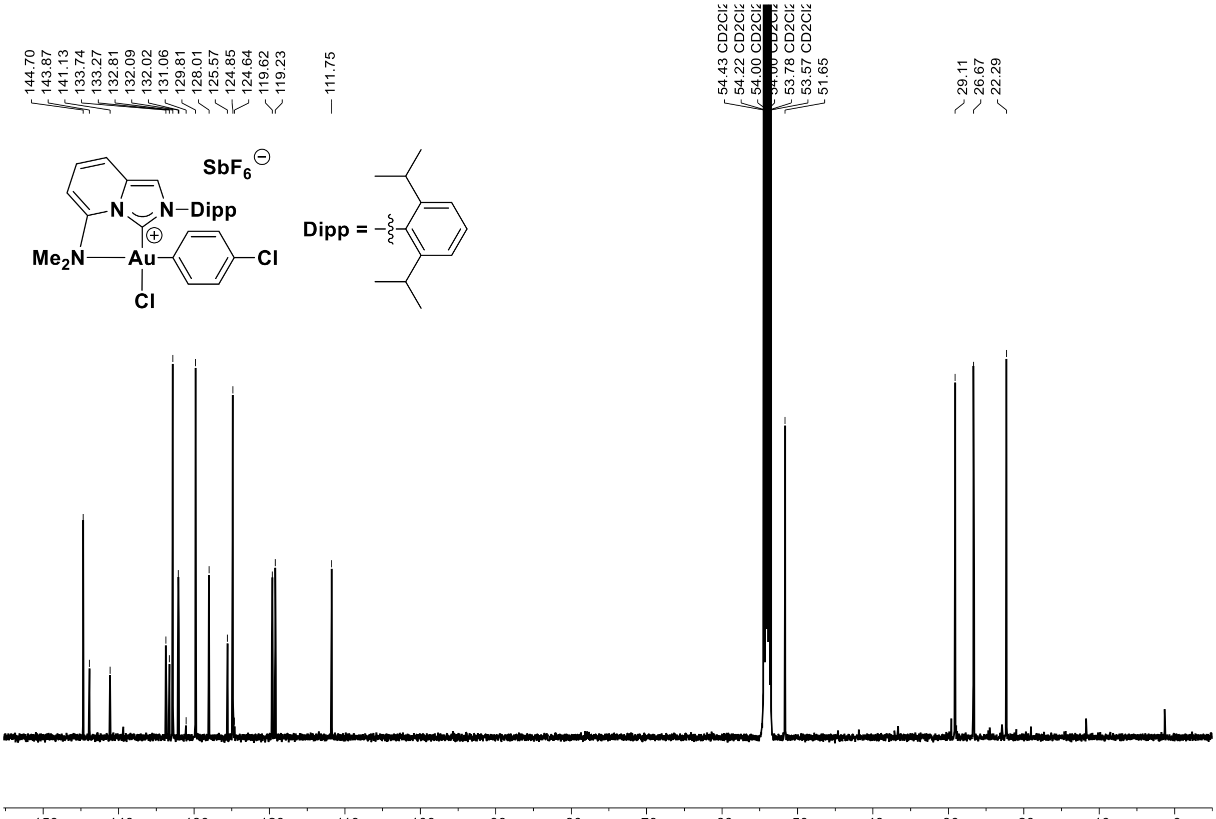

2h: ^{1}H, CD_2Cl_2, 500 MHz

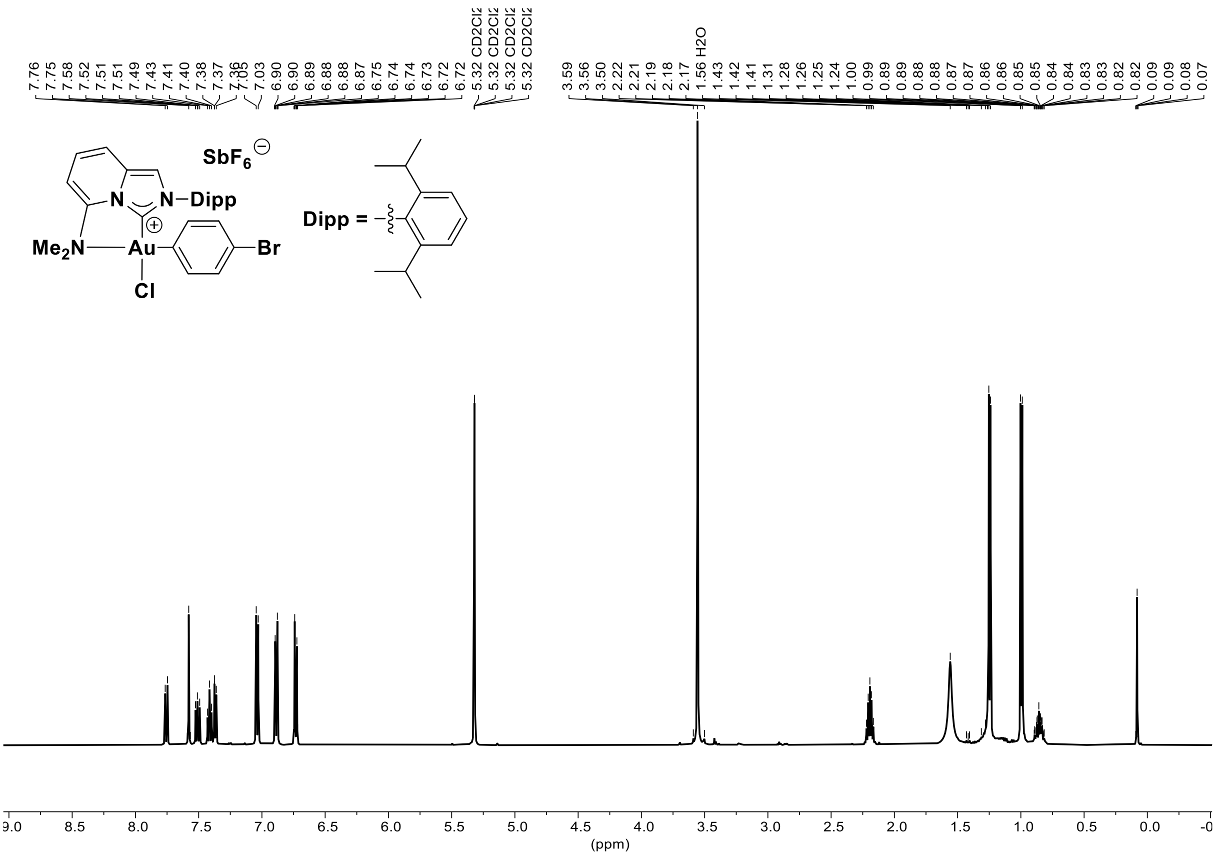

2h: ^{13}C, CD_2Cl_2, 126 MHz

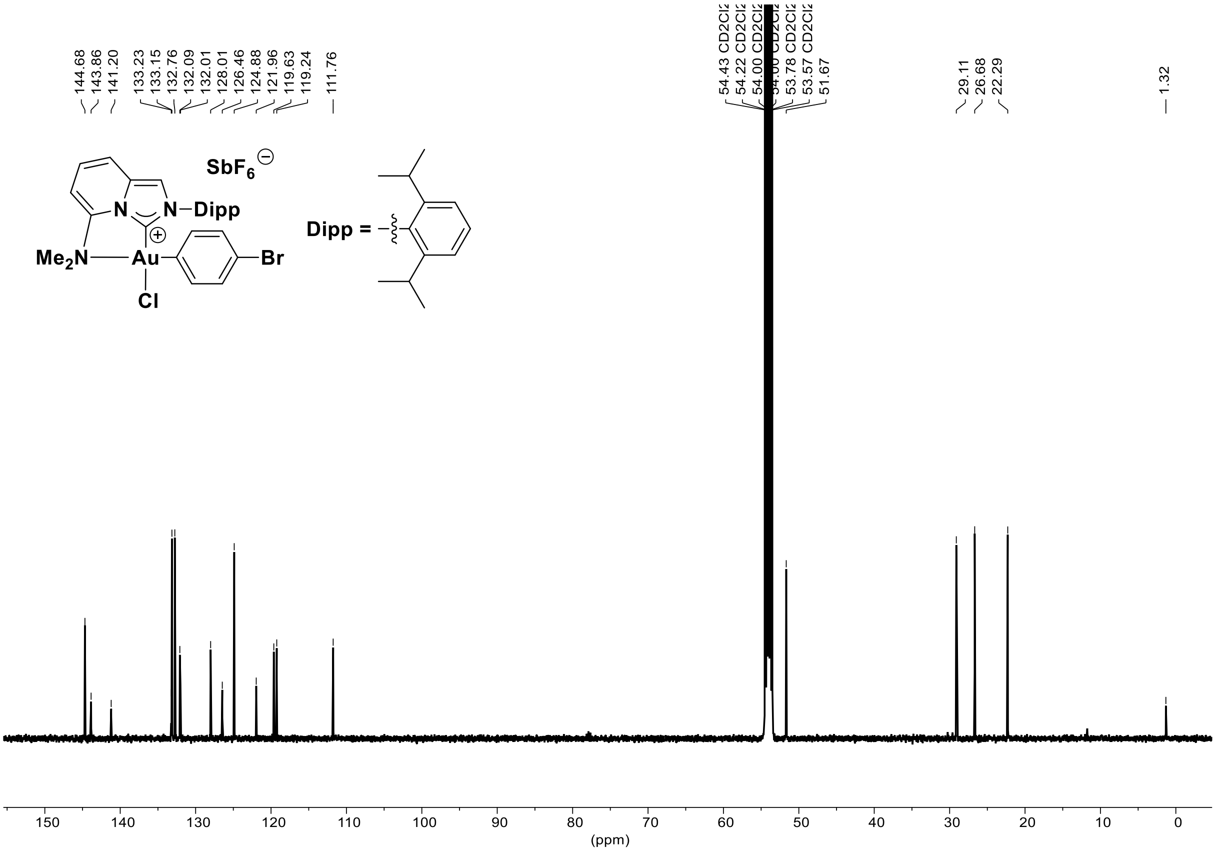

2i: ^{1}H, CD_2Cl_2, 500 MHz

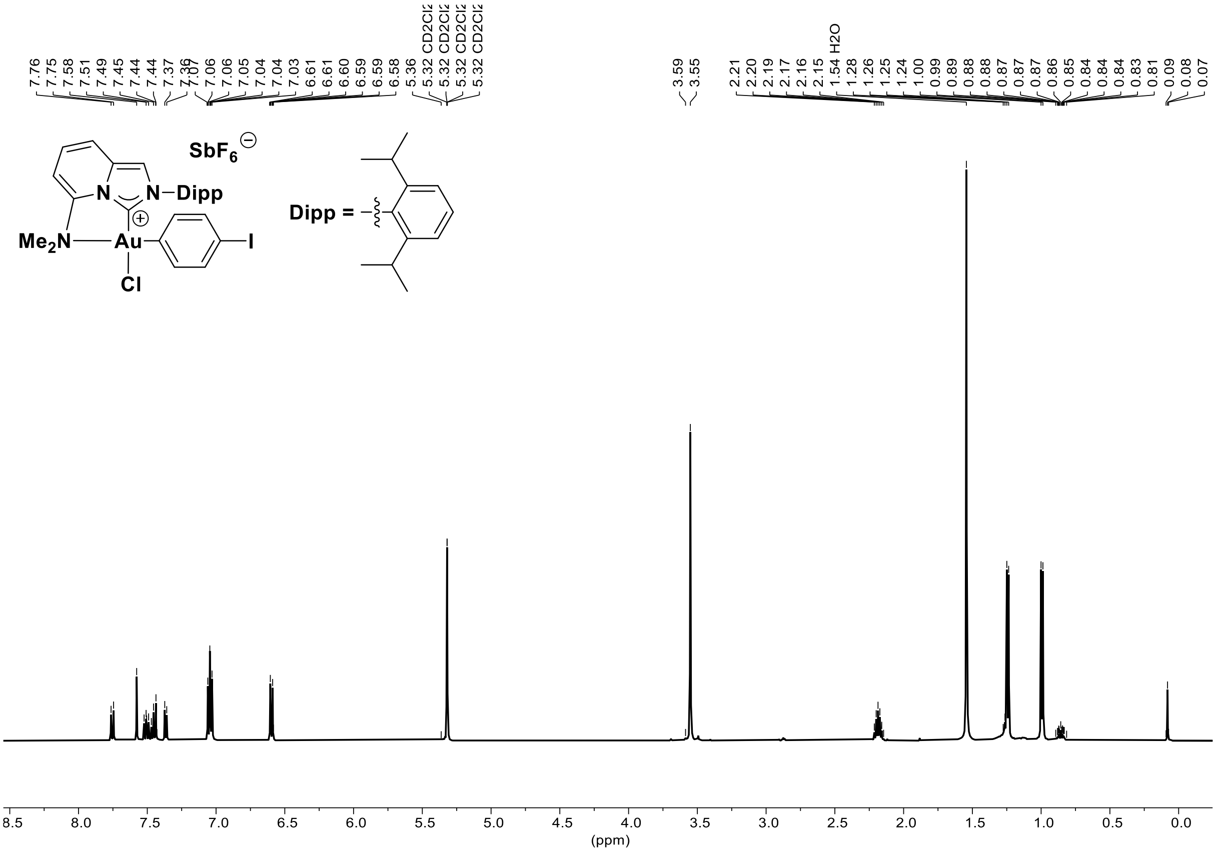

2i: ^{13}C, CD_2Cl_2, 126 MHz

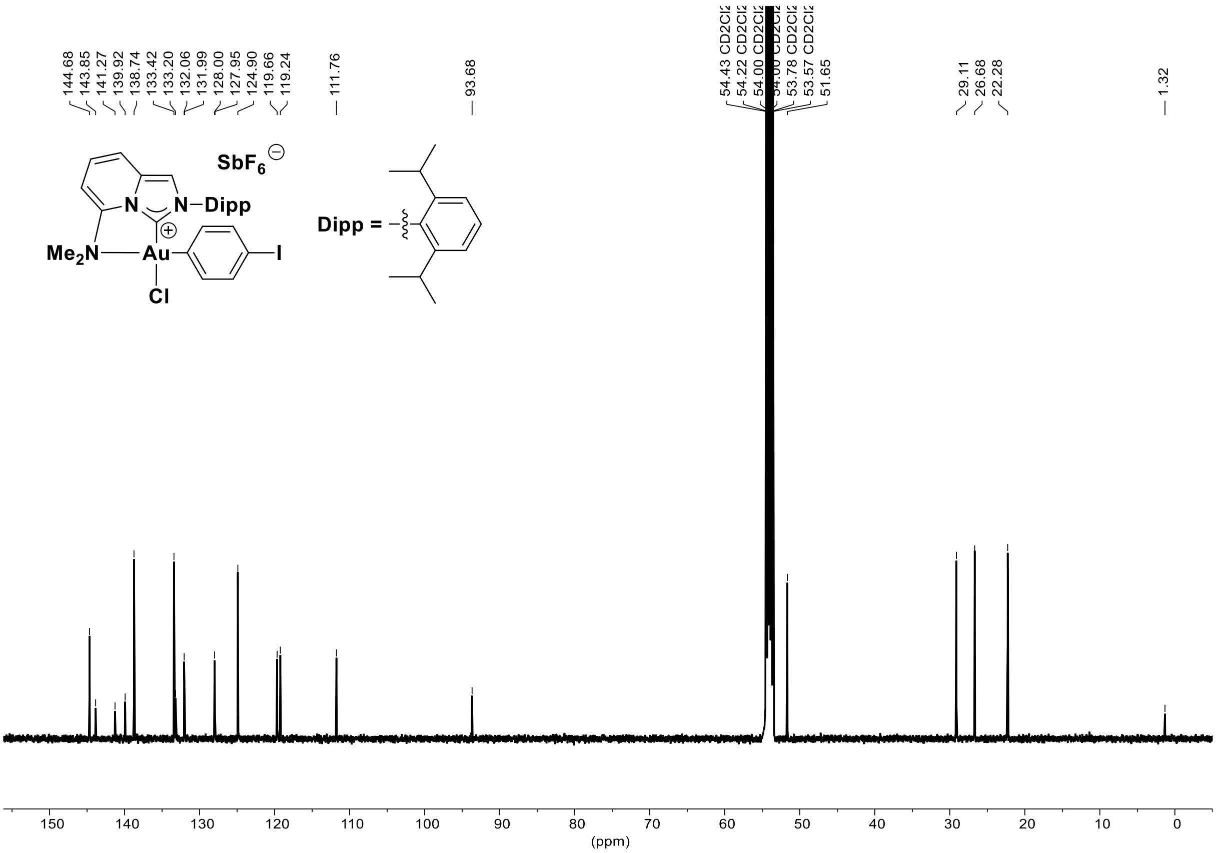

2j: ^{1}H, CD_2Cl_2, 500 MHz

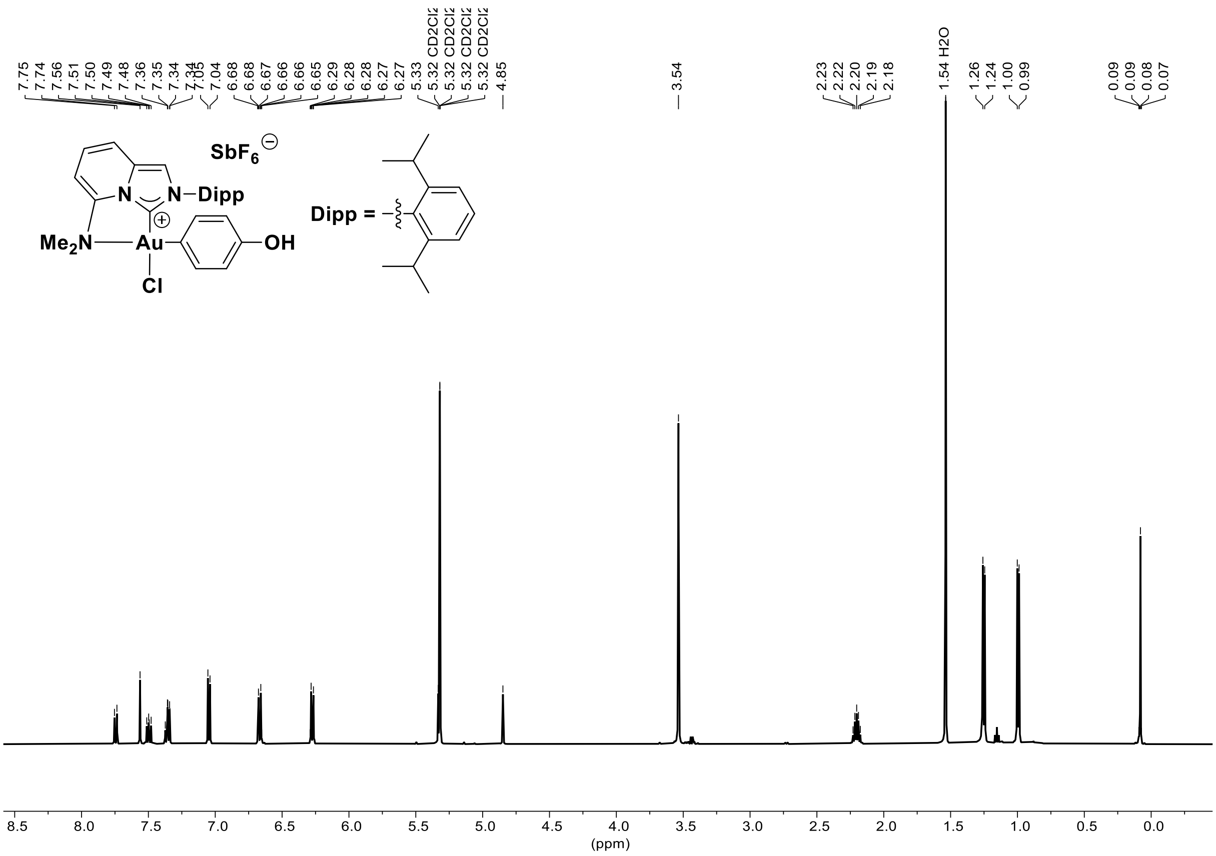

2j: ^{13}C, CD_2Cl_2, 126 MHz

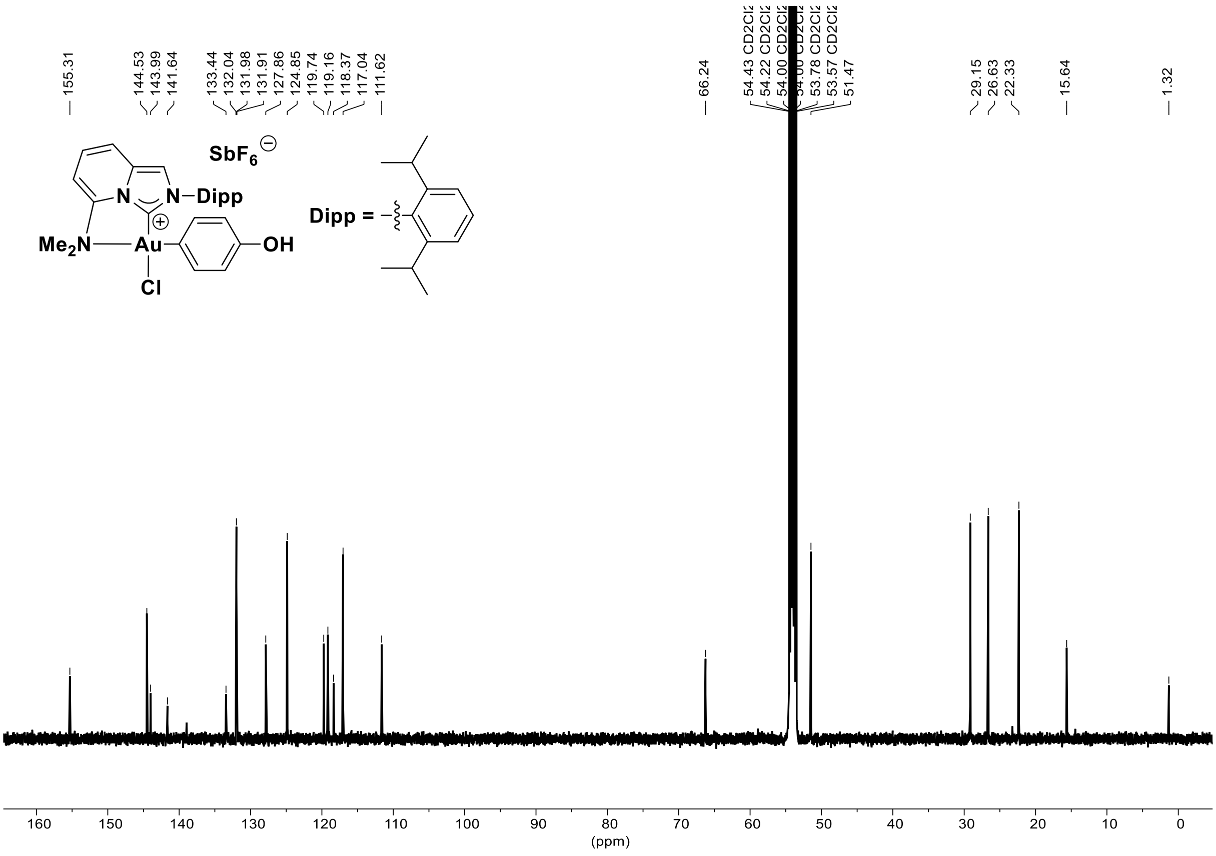

2k: ^{1}H, CD_2Cl_2, 500 MHz

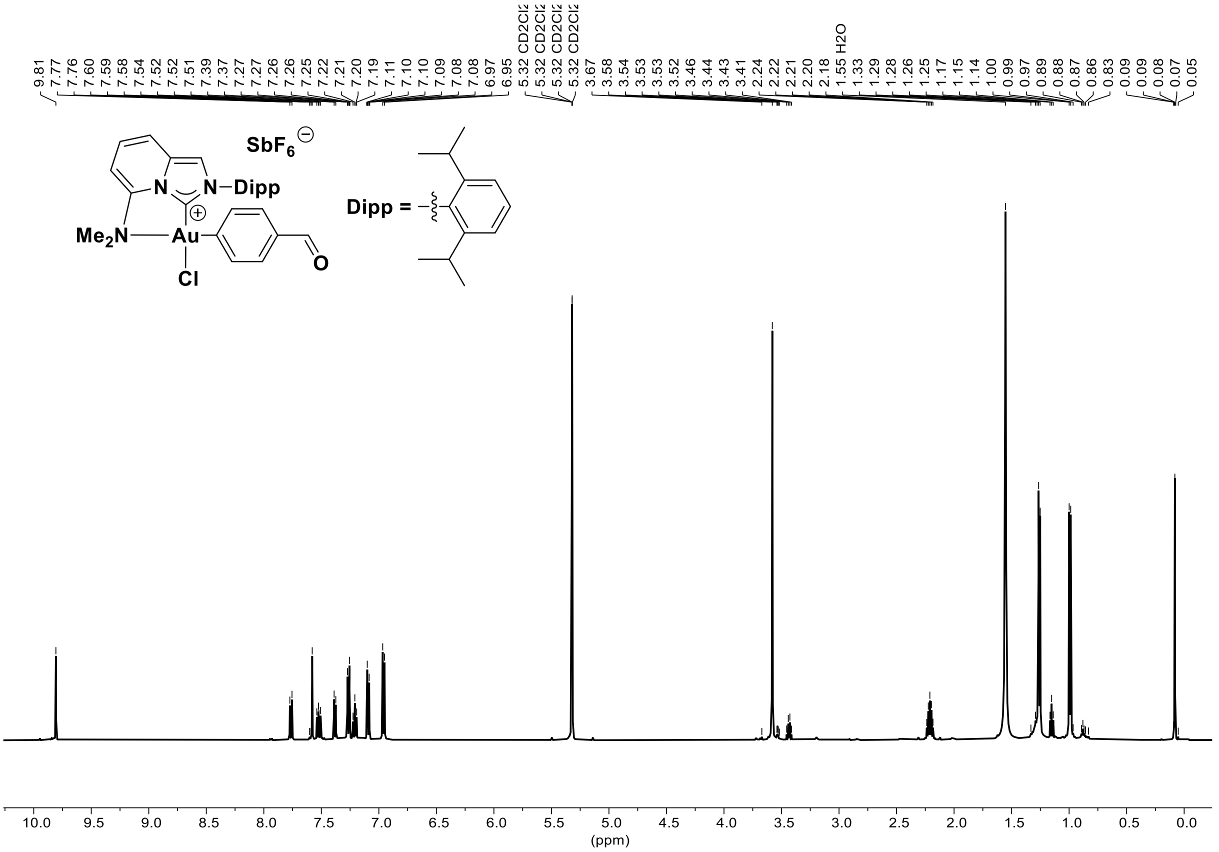

2k: ^{13}C, CD_2Cl_2, 126 MHz

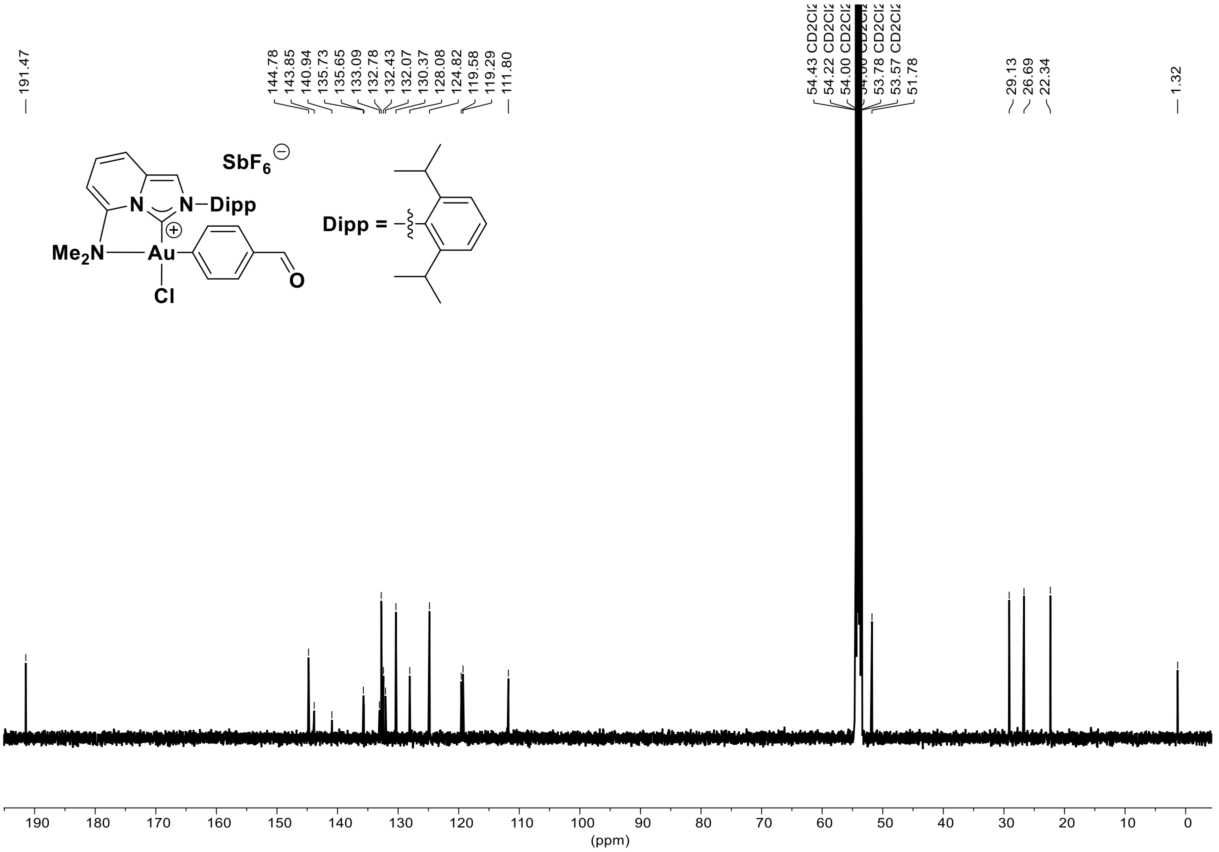

2l: ^{1}H, CD_2Cl_2, 500 MHz

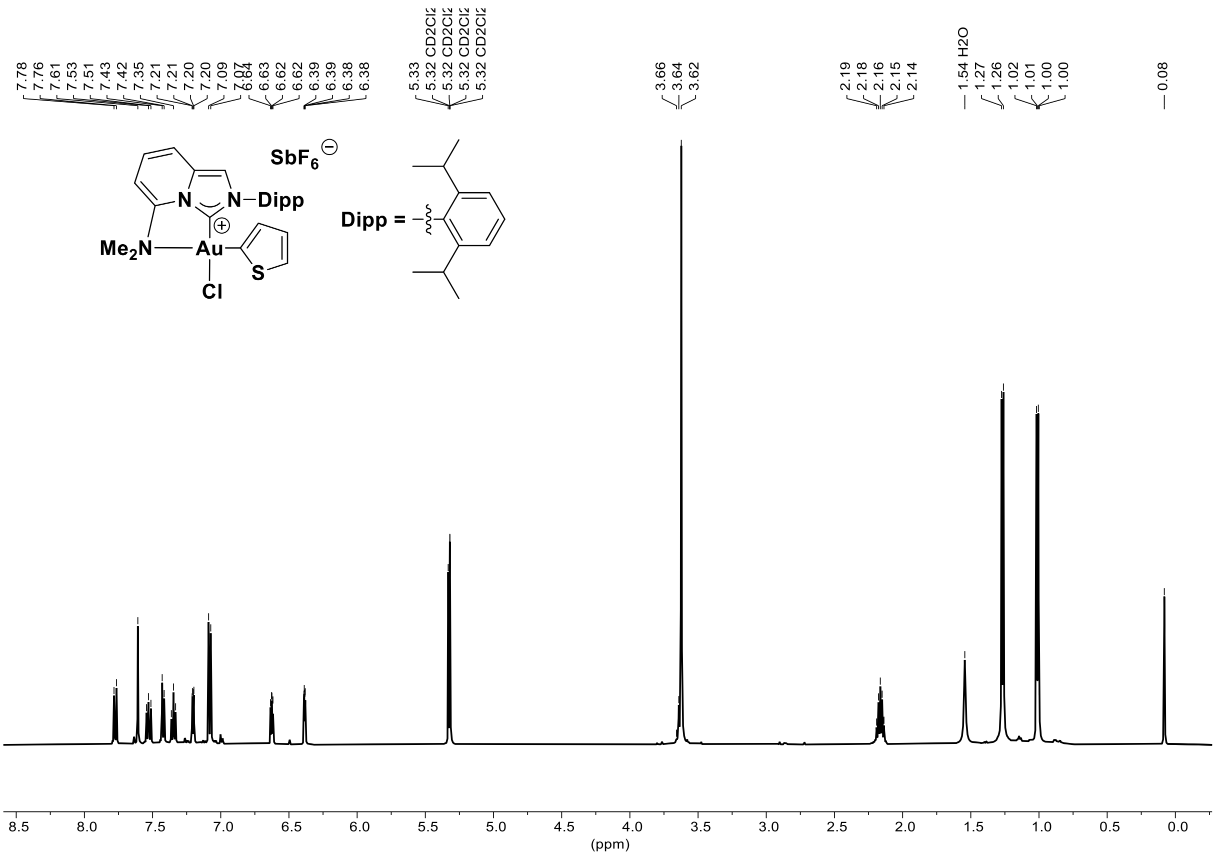

2l: ^{13}C, CD_2Cl_2, 126 MHz

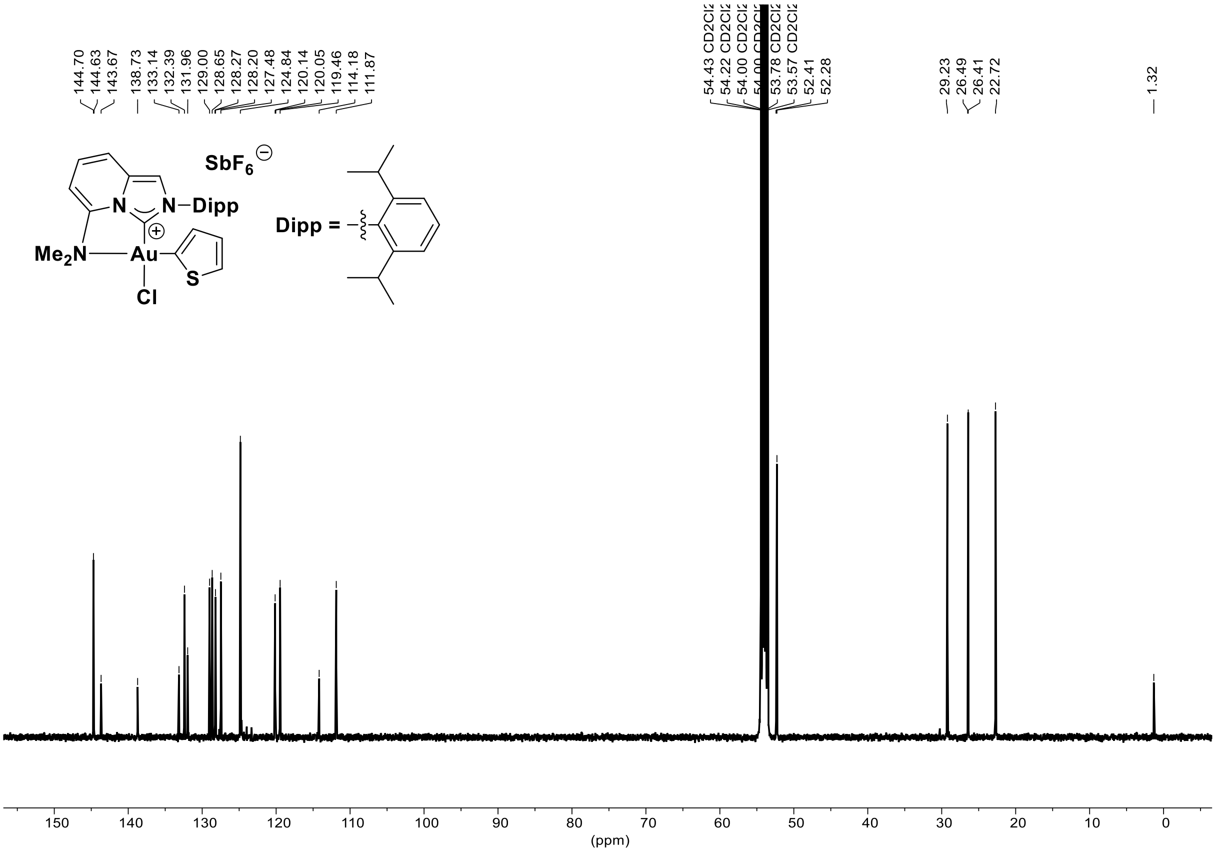

2m: ^{1}H, CD_2Cl_2, 500 MHz

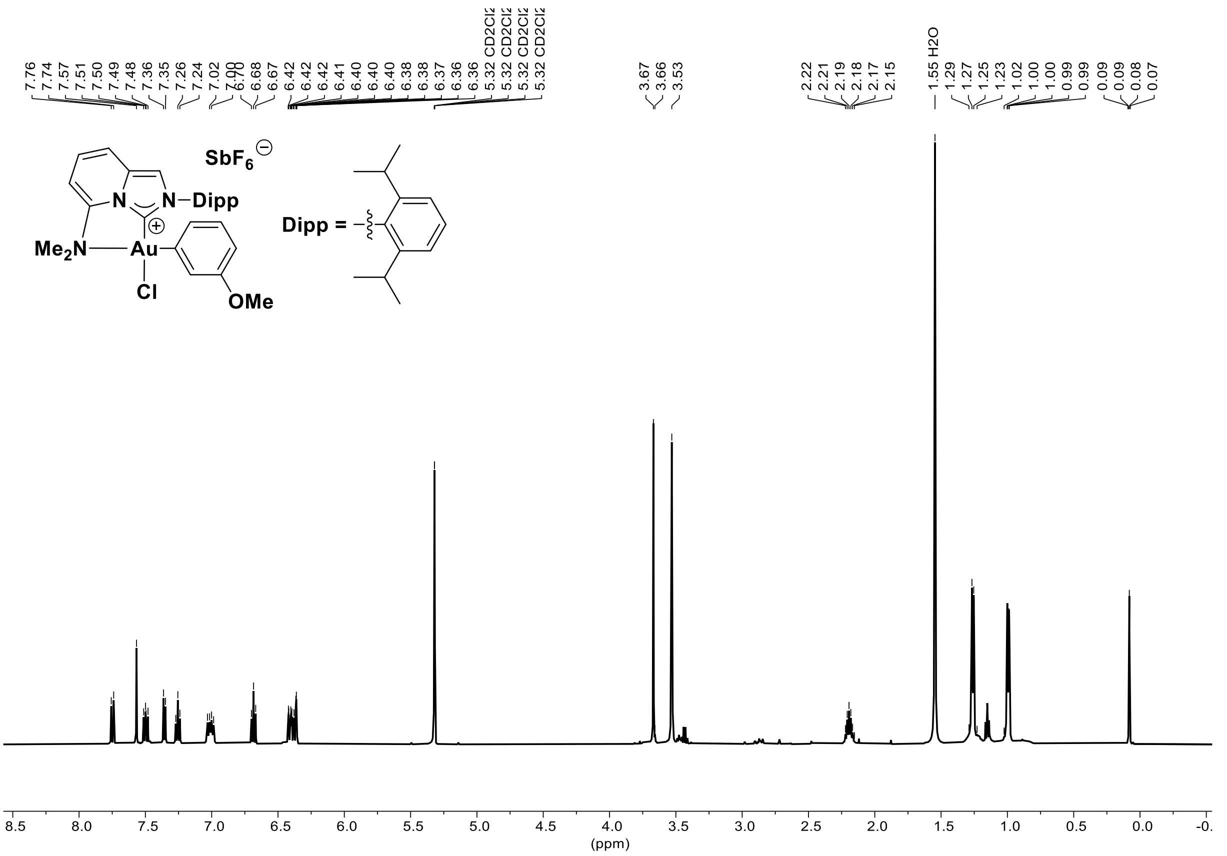

2m: ^{13}C, CD_2Cl_2, 126 MHz

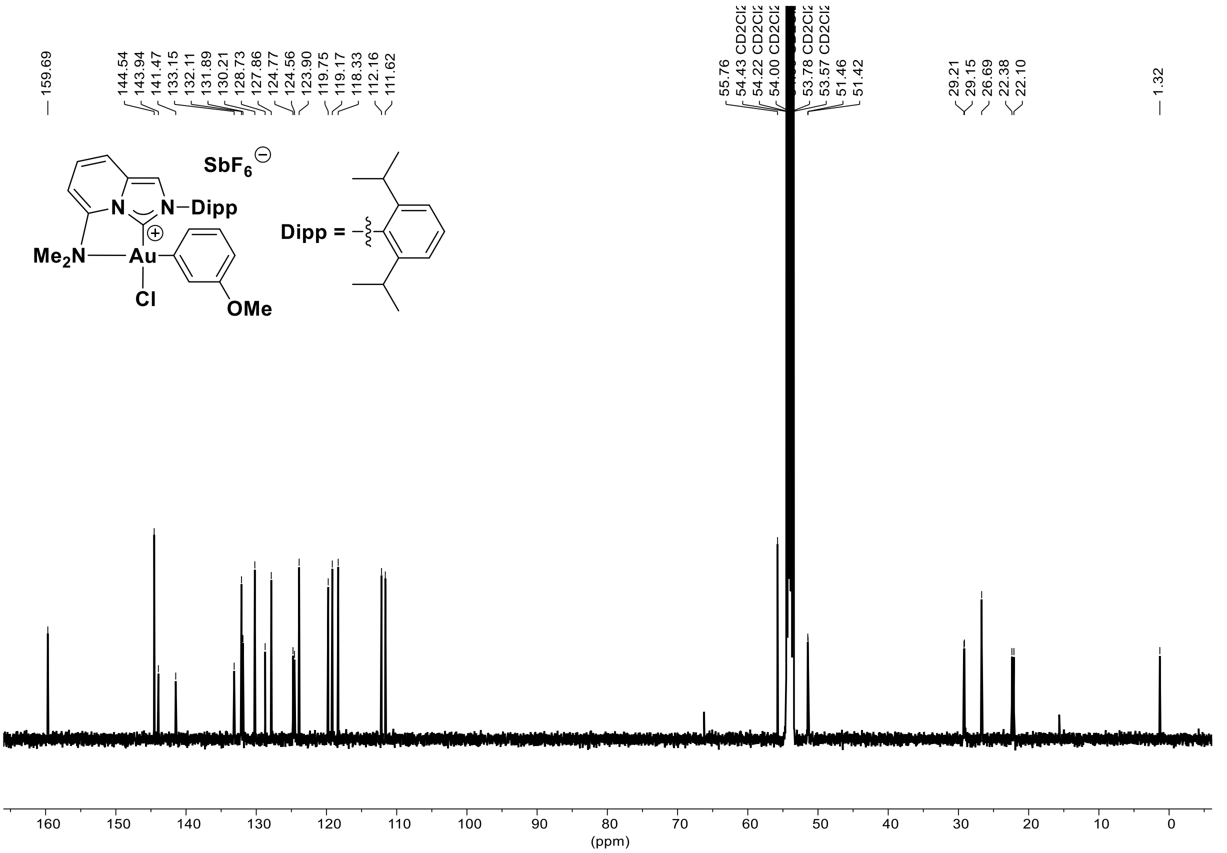

2p: ^{1}H, CD_2Cl_2, 500 MHz

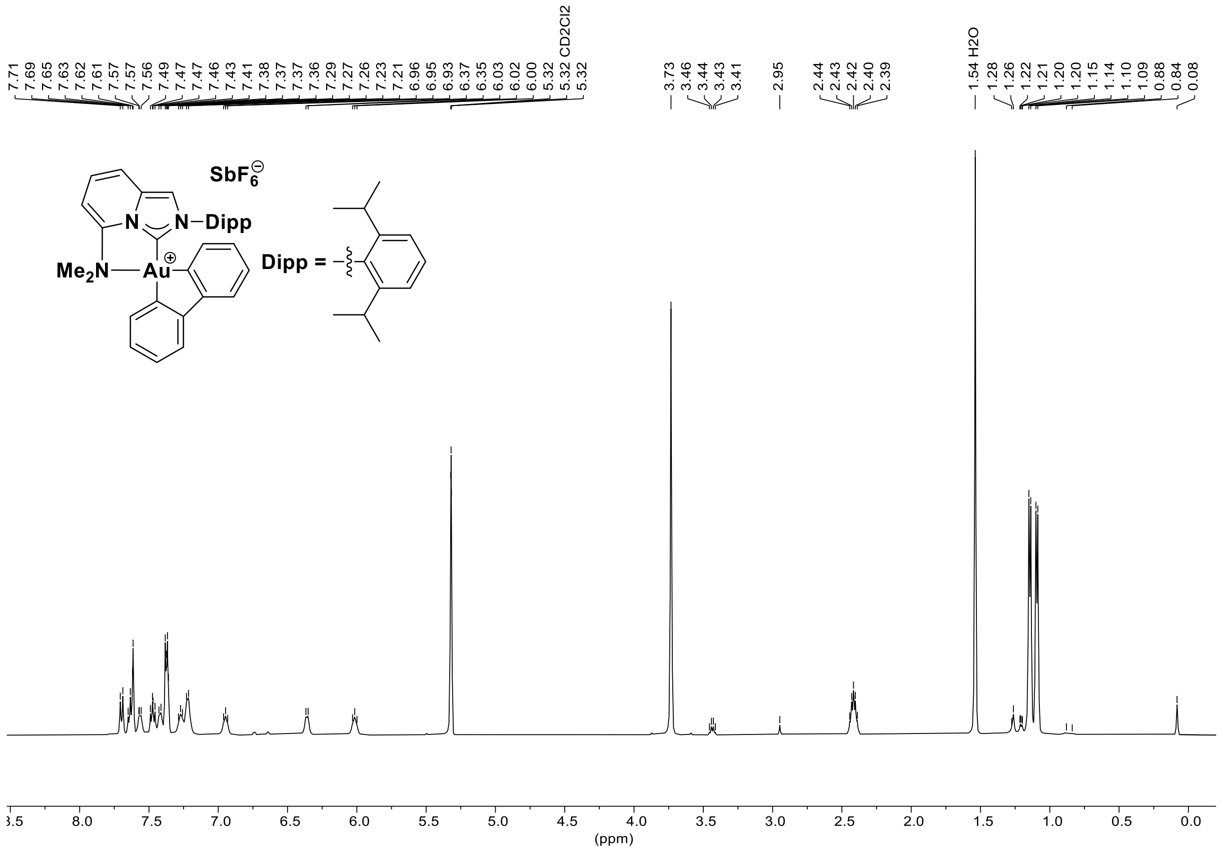

2p: ^{13}C, CD_2Cl_2, 126 MHz

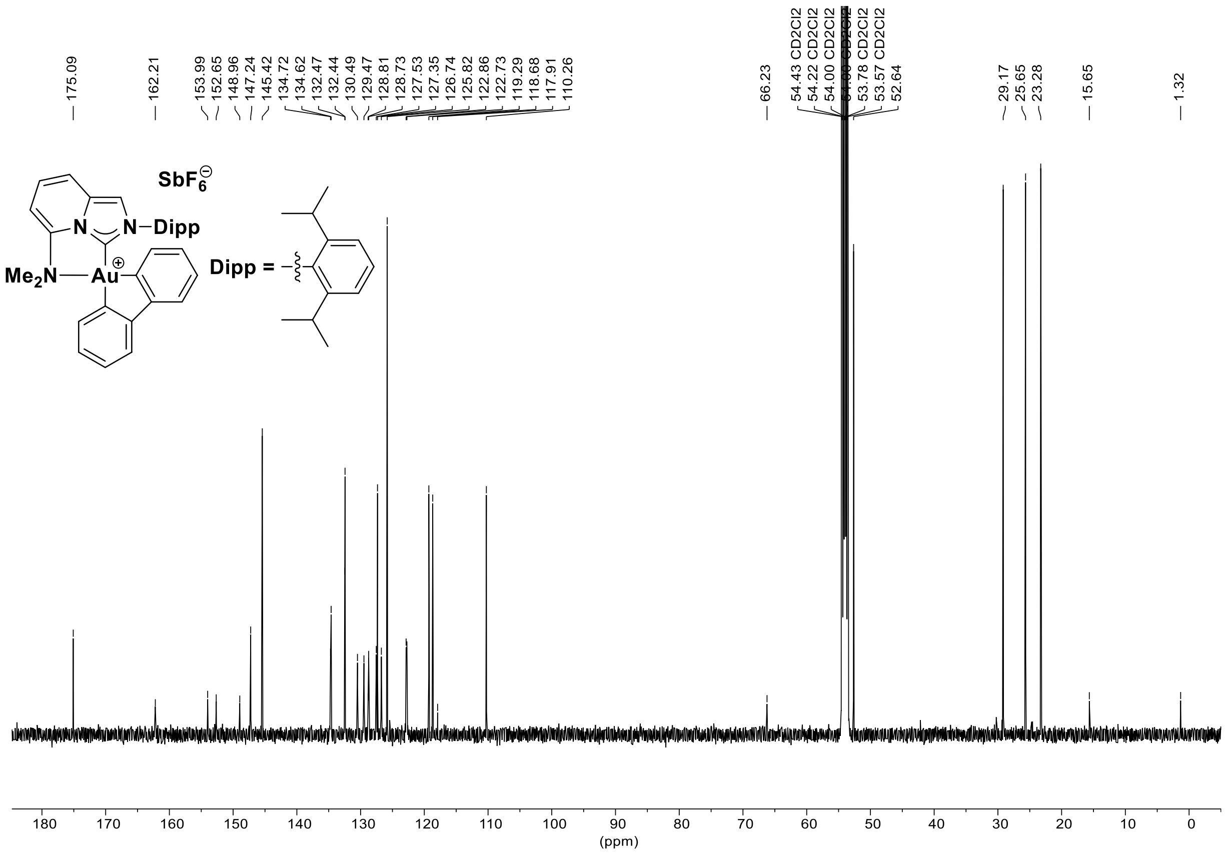

5f: ^{1}H, $CDCl_3$, 400 MHz

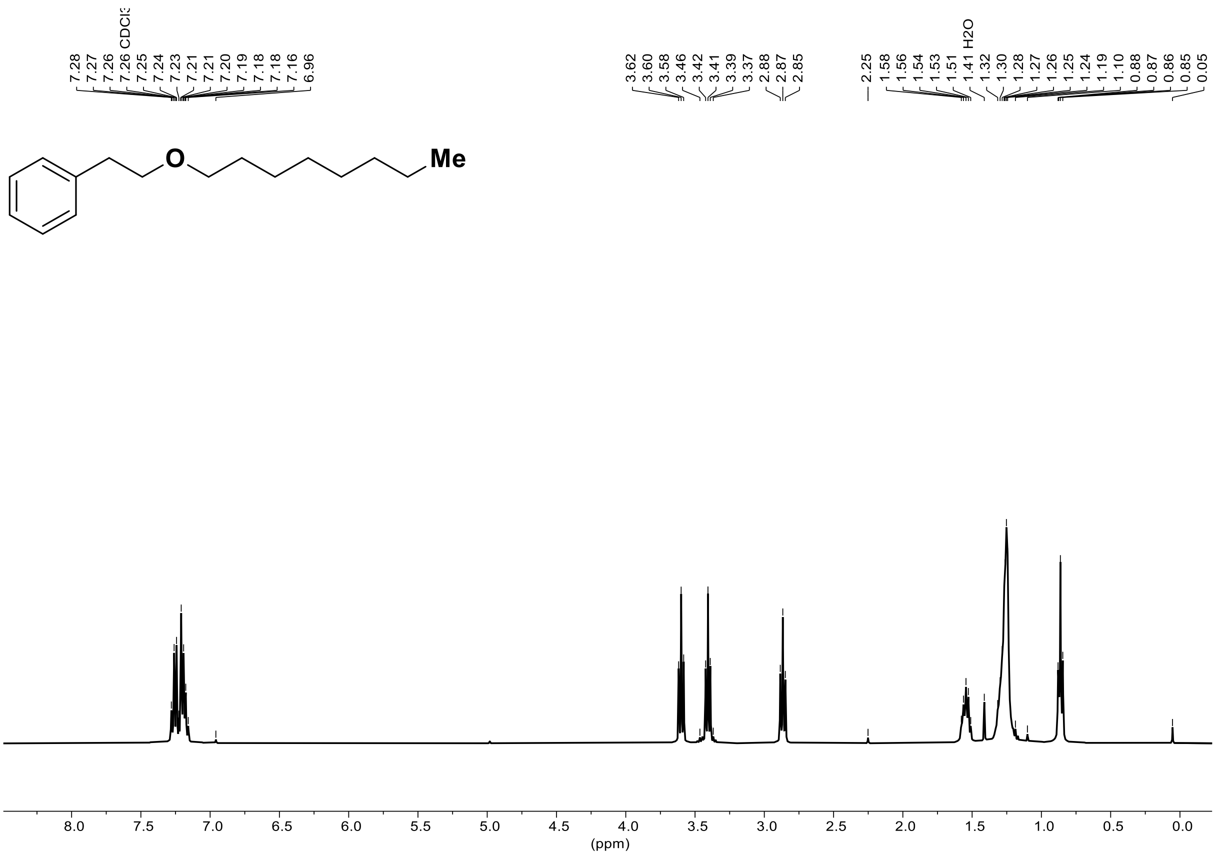

5f: ^{13}C, $CDCl_3$, 101 MHz

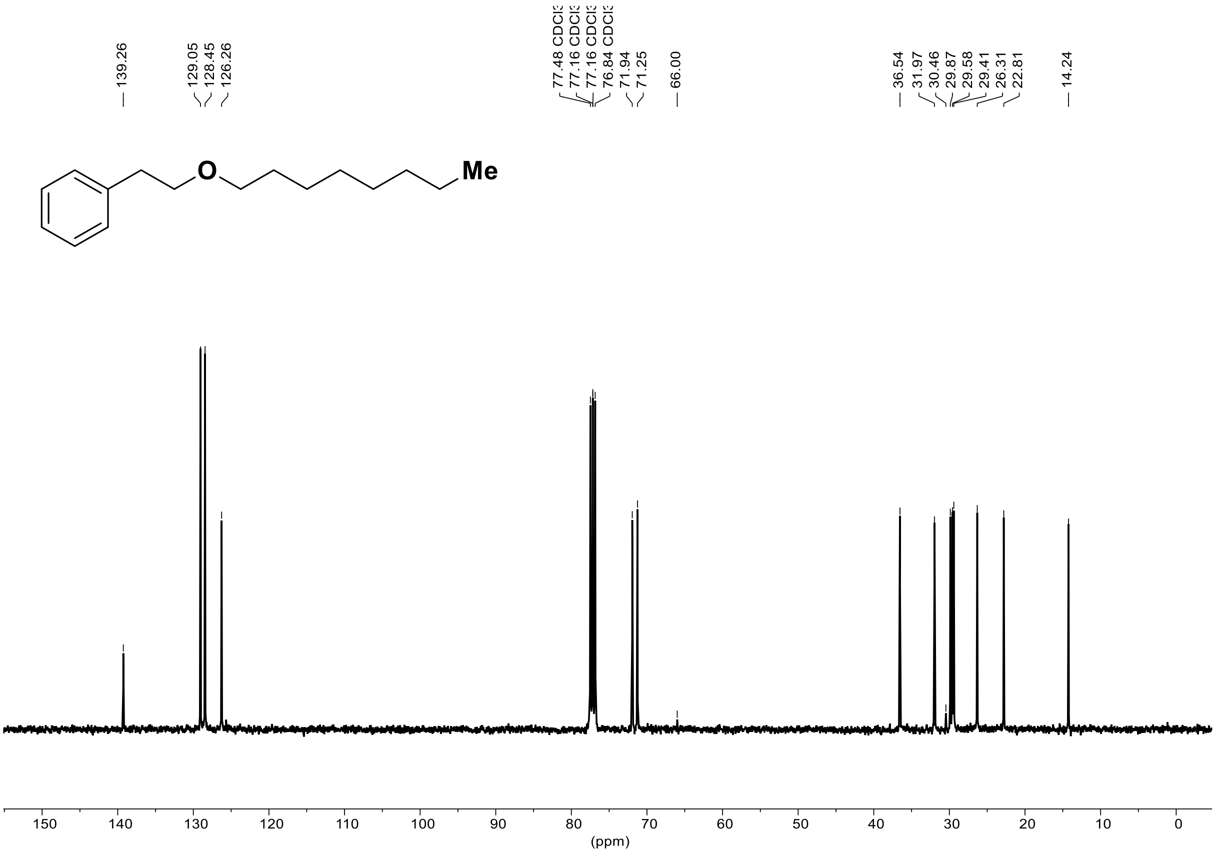

5h: ^{1}H, $CDCl_3$, 400 MHz

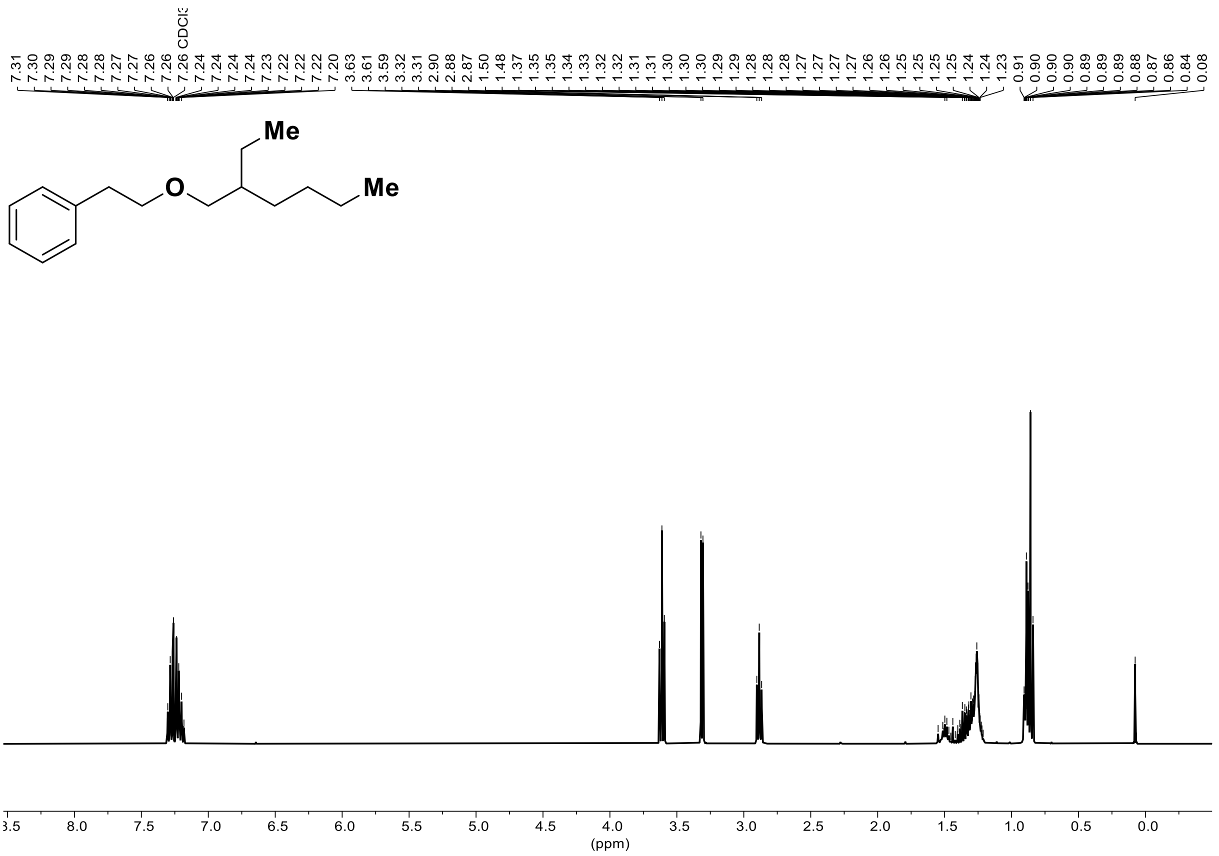

5h: ^{13}C, $CDCl_3$, 101 MHz

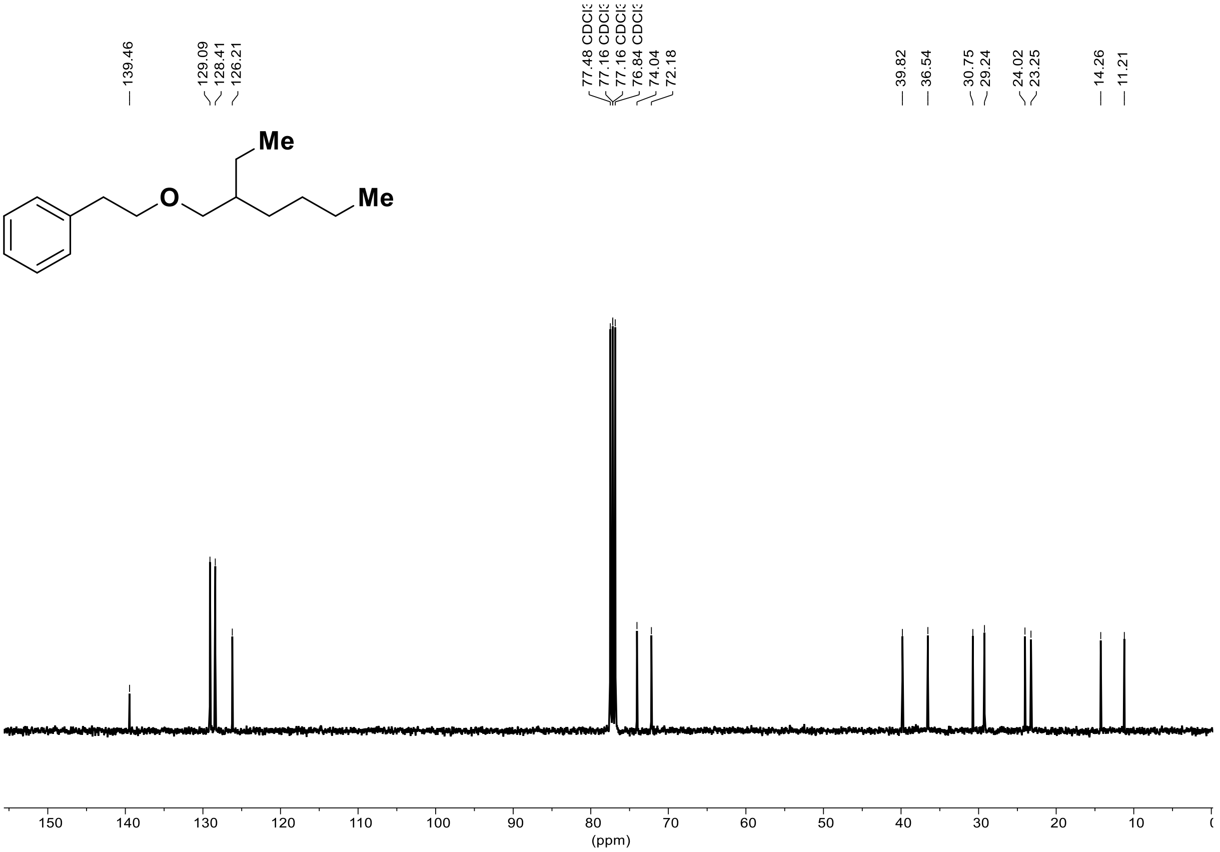

5m: ^{1}H, $CDCl_3$, 400 MHz

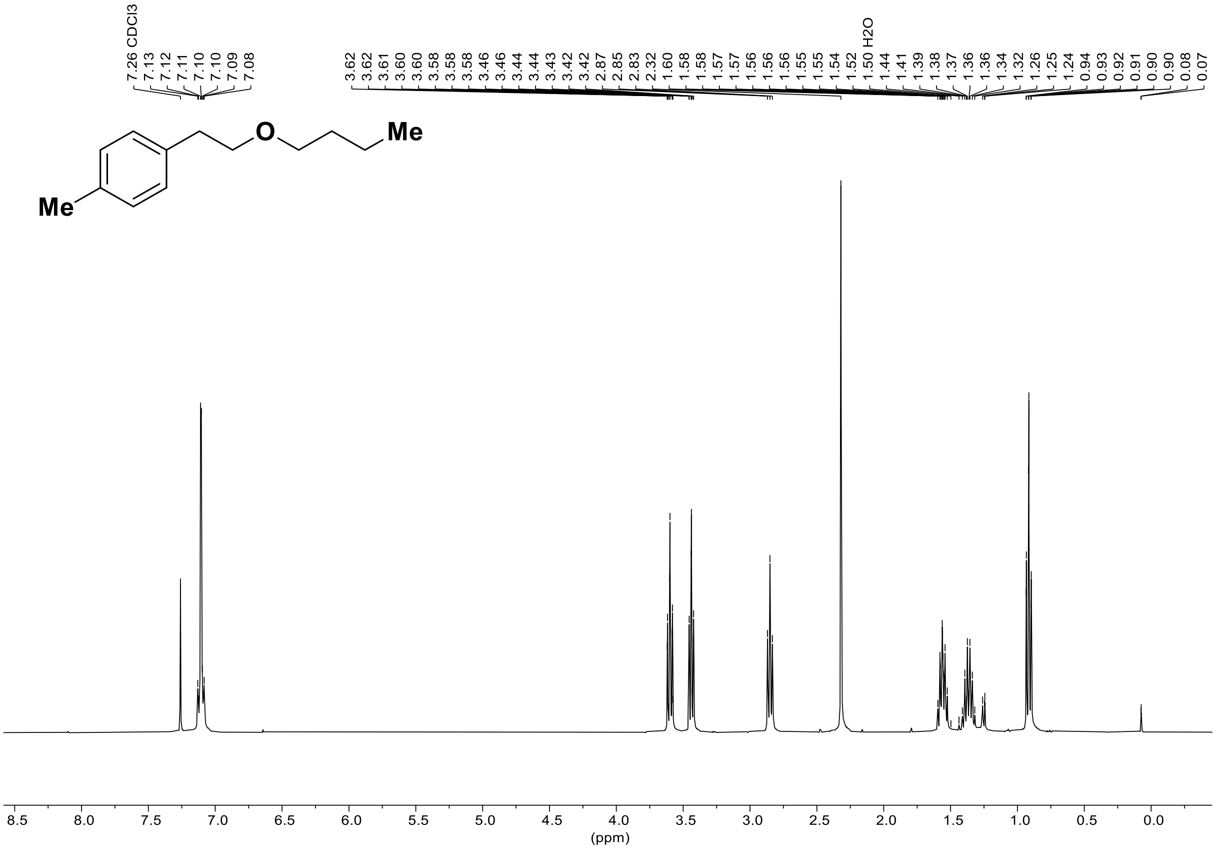

5m: ^{13}C, $CDCl_3$, 101 MHz

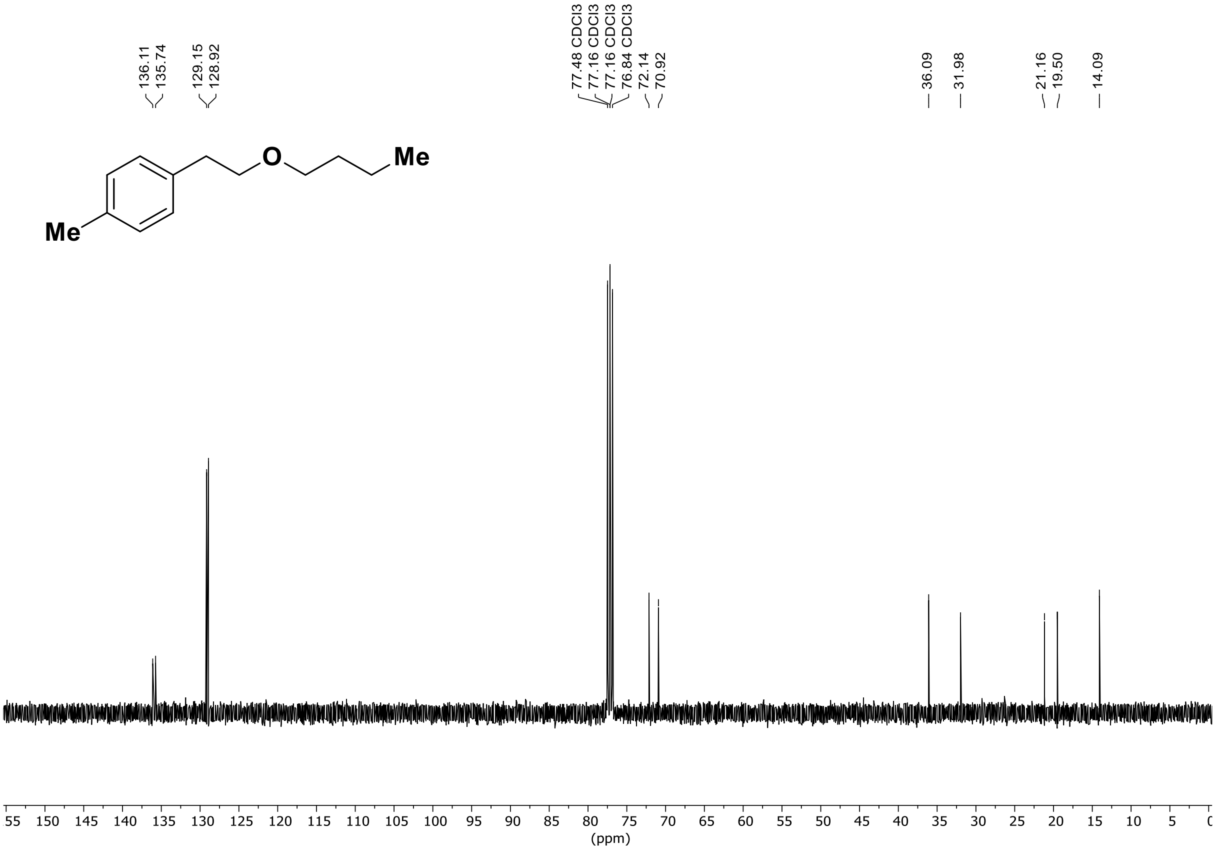

5n: ^{1}H, $CDCl_3$, 400 MHz

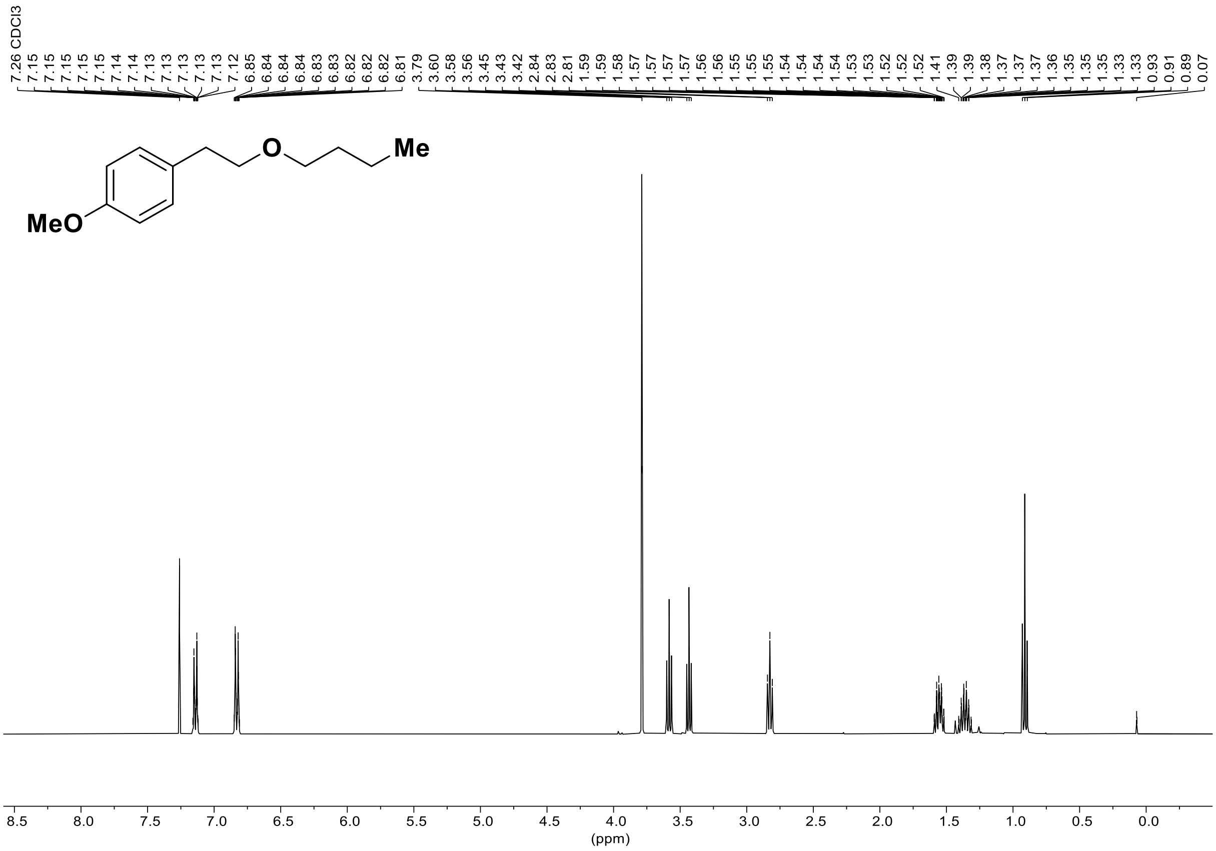

5n: ^{13}C, $CDCl_3$, 101 MHz

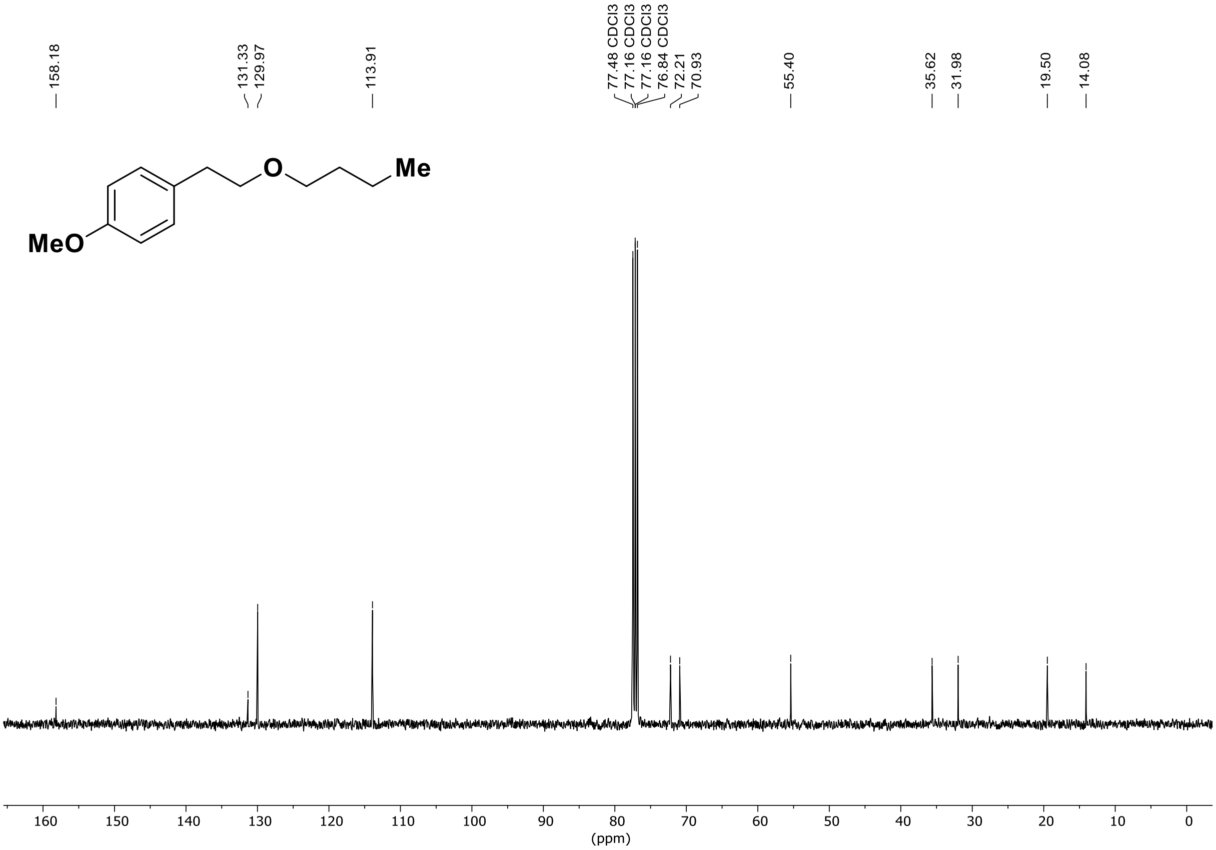

5o: ^{1}H, $CDCl_3$, 400 MHz

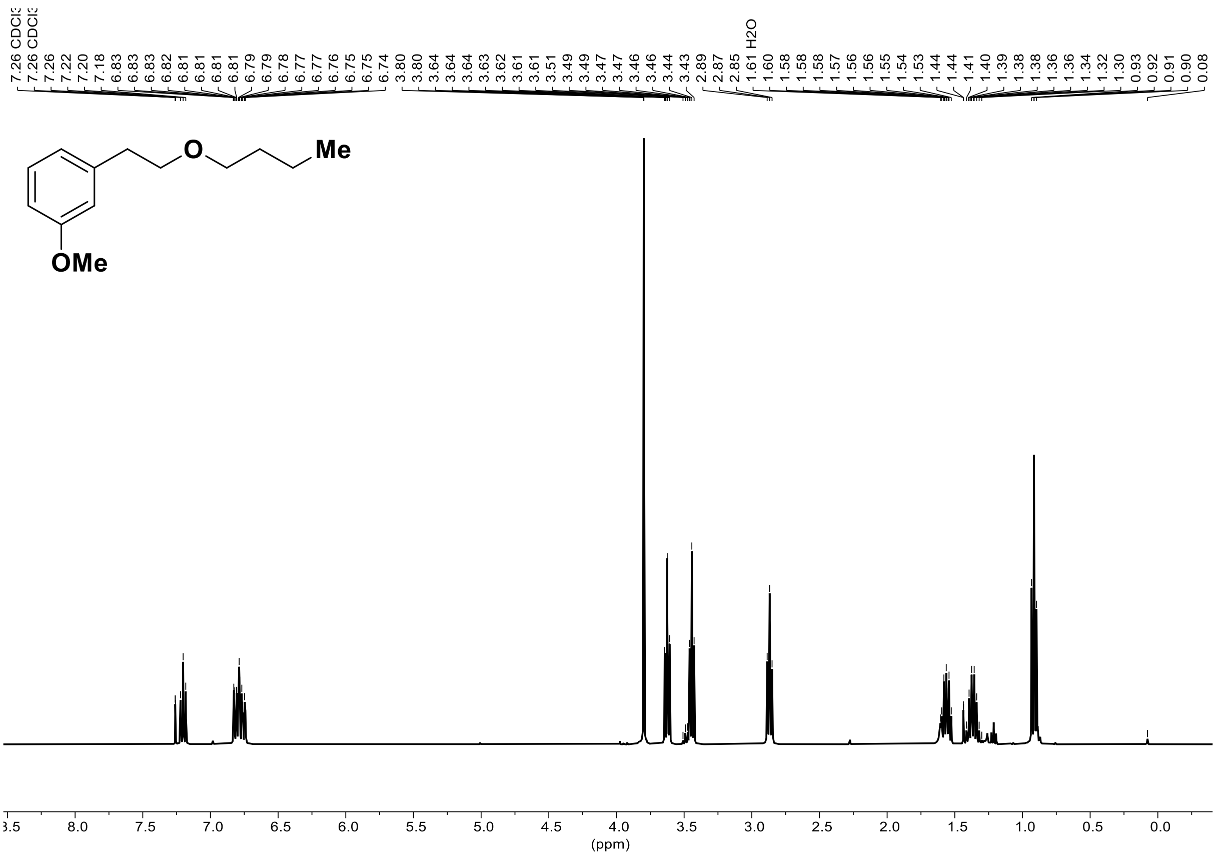

5o: ^{13}C, $CDCl_3$, 101 MHz

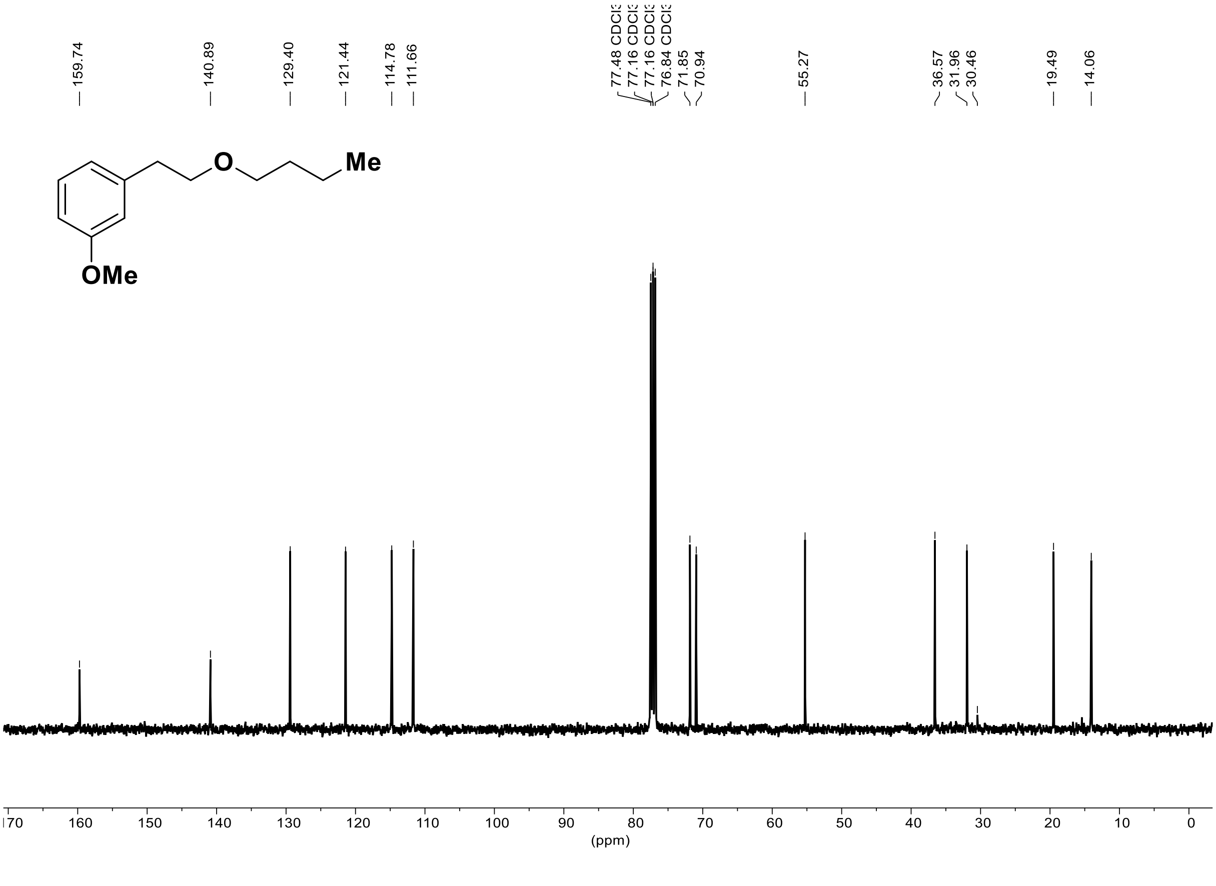

5p: ^{1}H, $CDCl_3$, 400 MHz

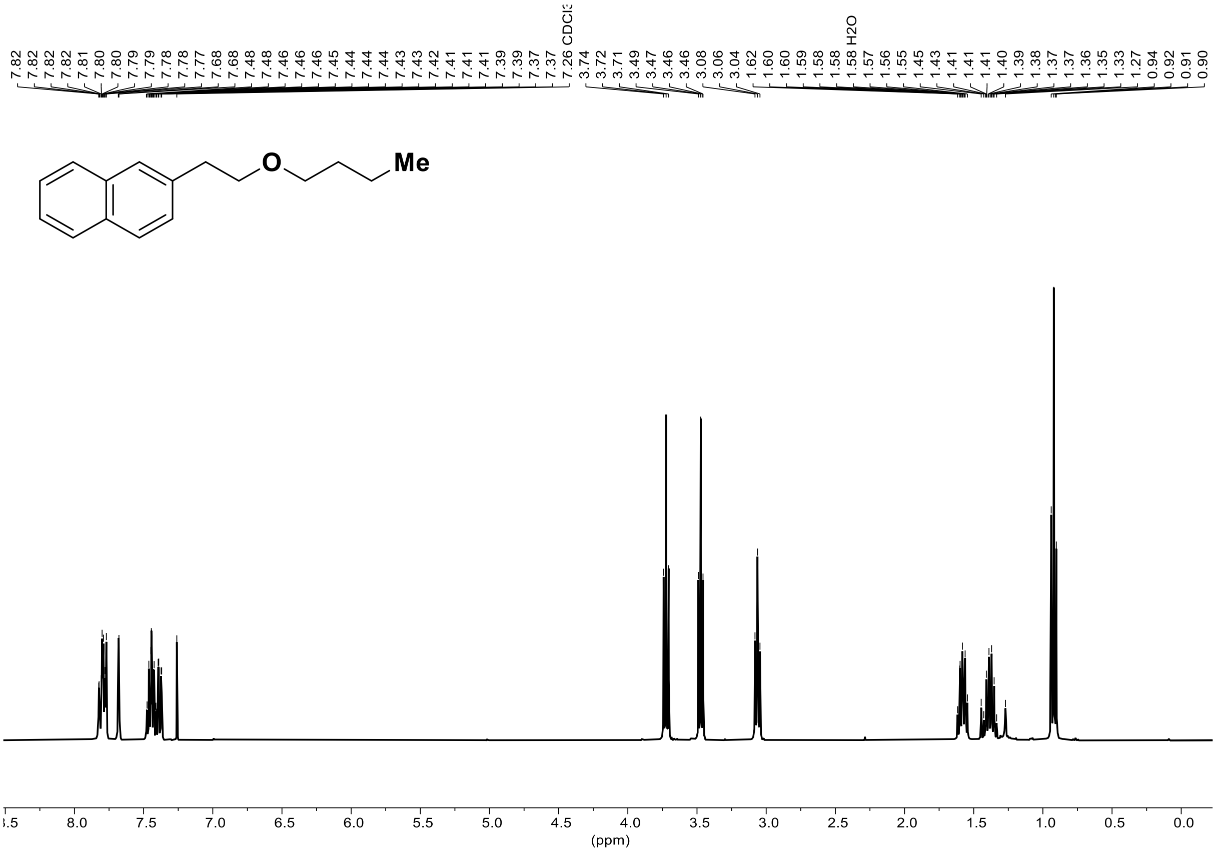

5p: ^{13}C, $CDCl_3$, 101 MHz

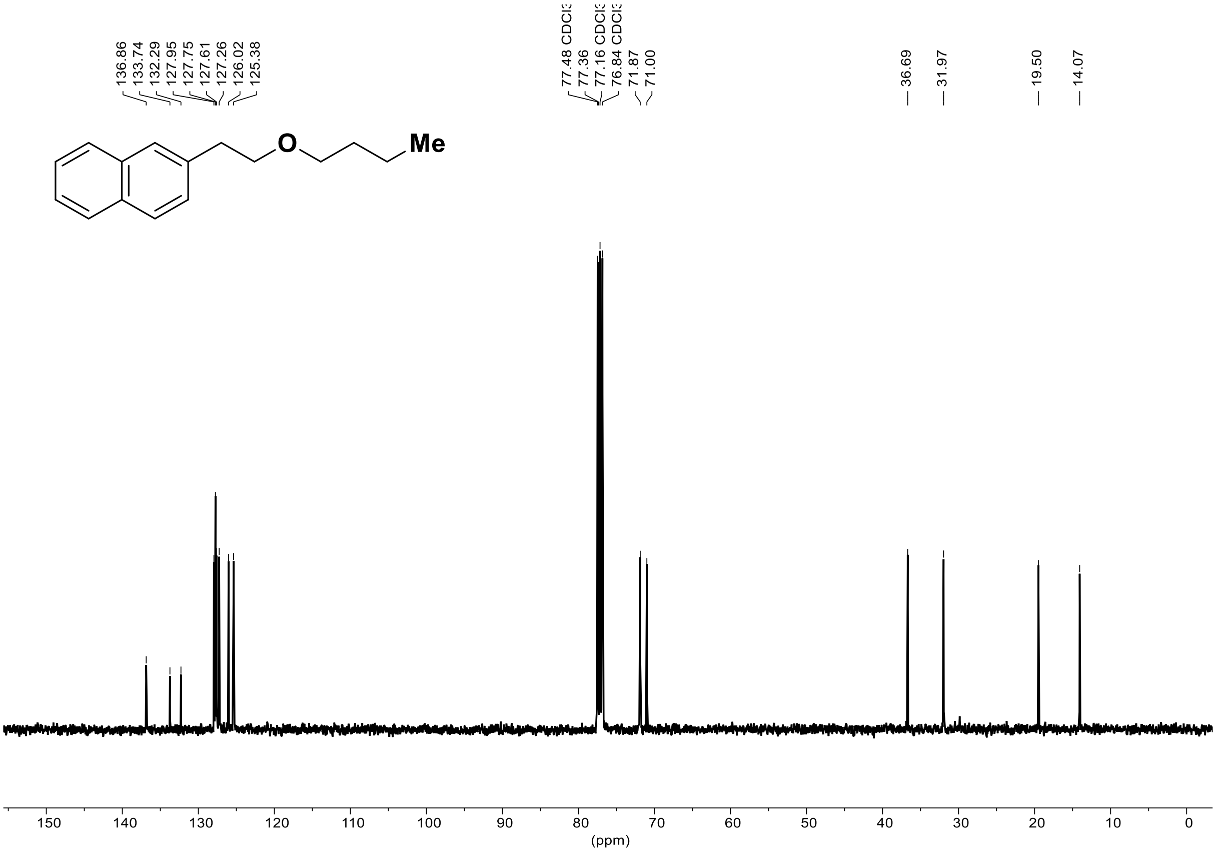

5q: ^{1}H, $CDCl_3$, 400 MHz

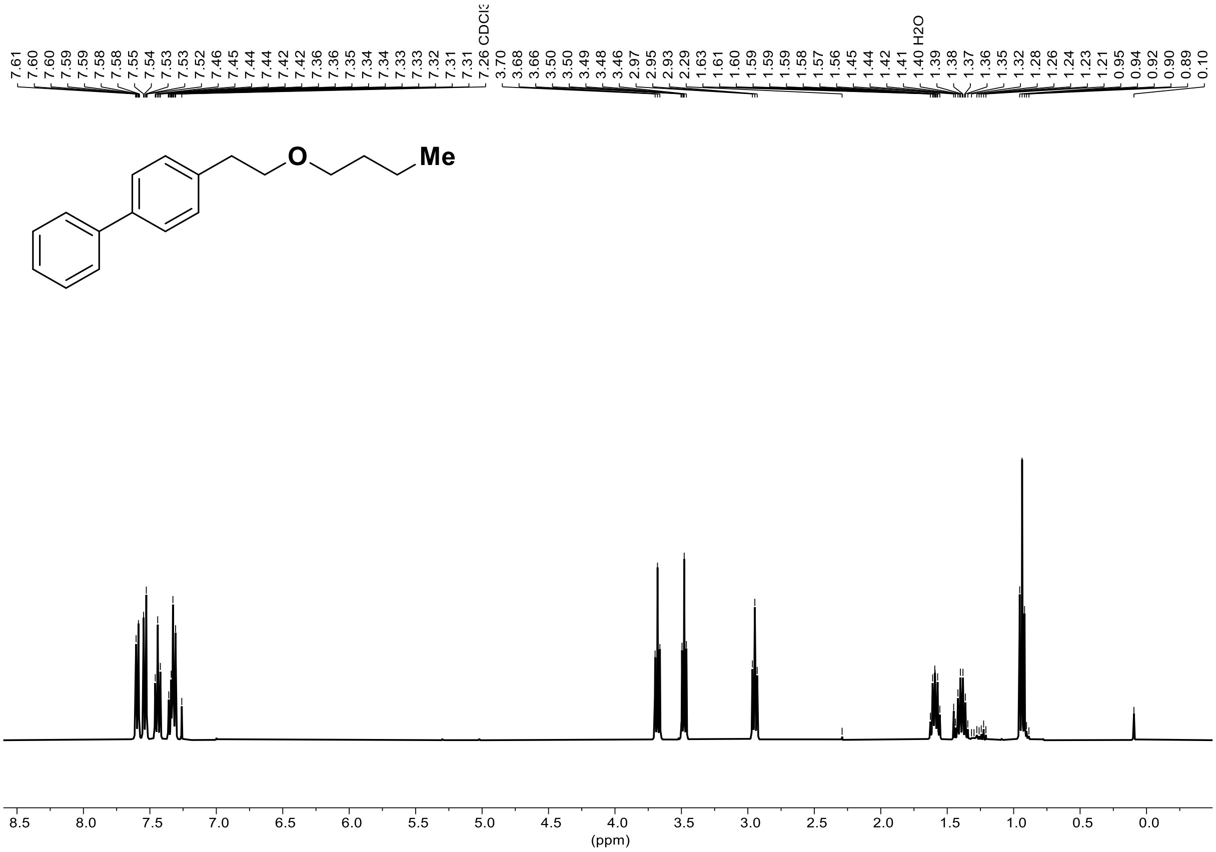

5q: ^{13}C, $CDCl_3$, 101 MHz

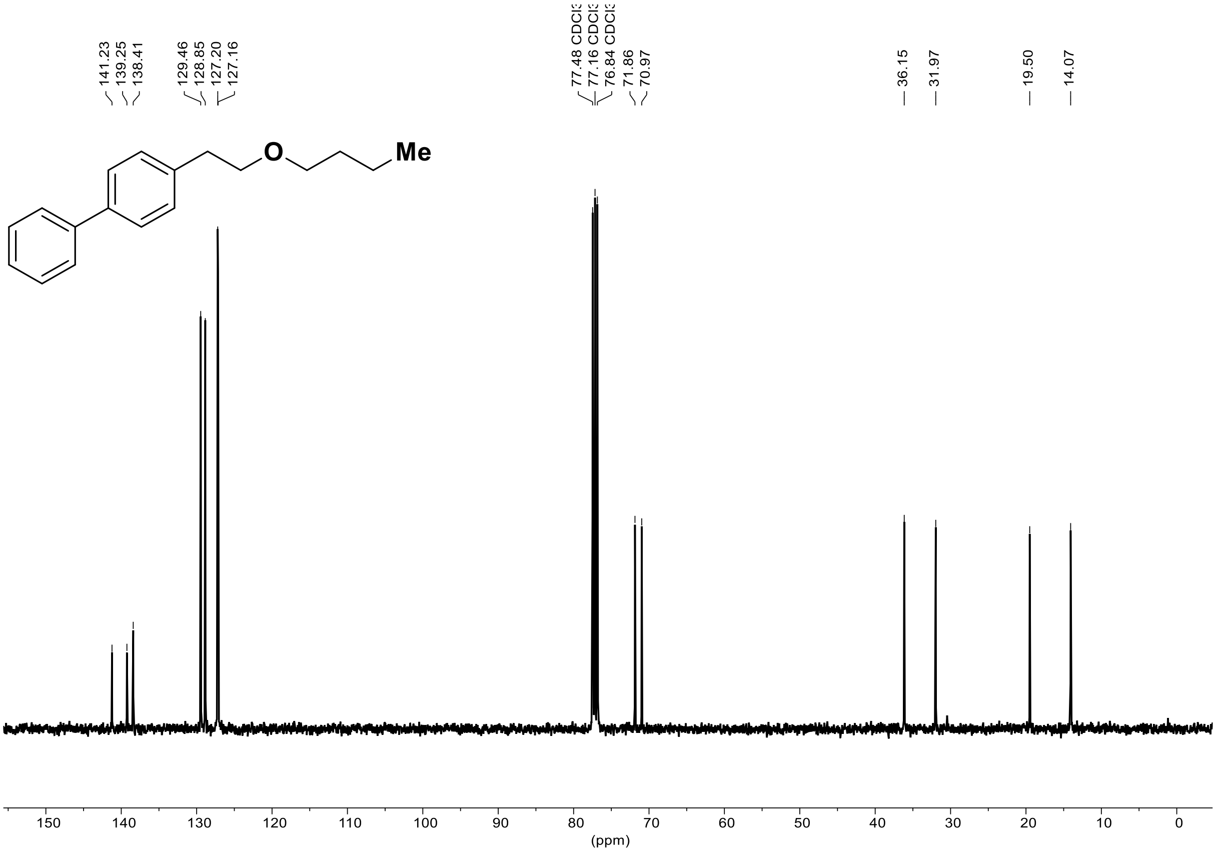

5r: ^{1}H, $CDCl_3$, 400 MHz

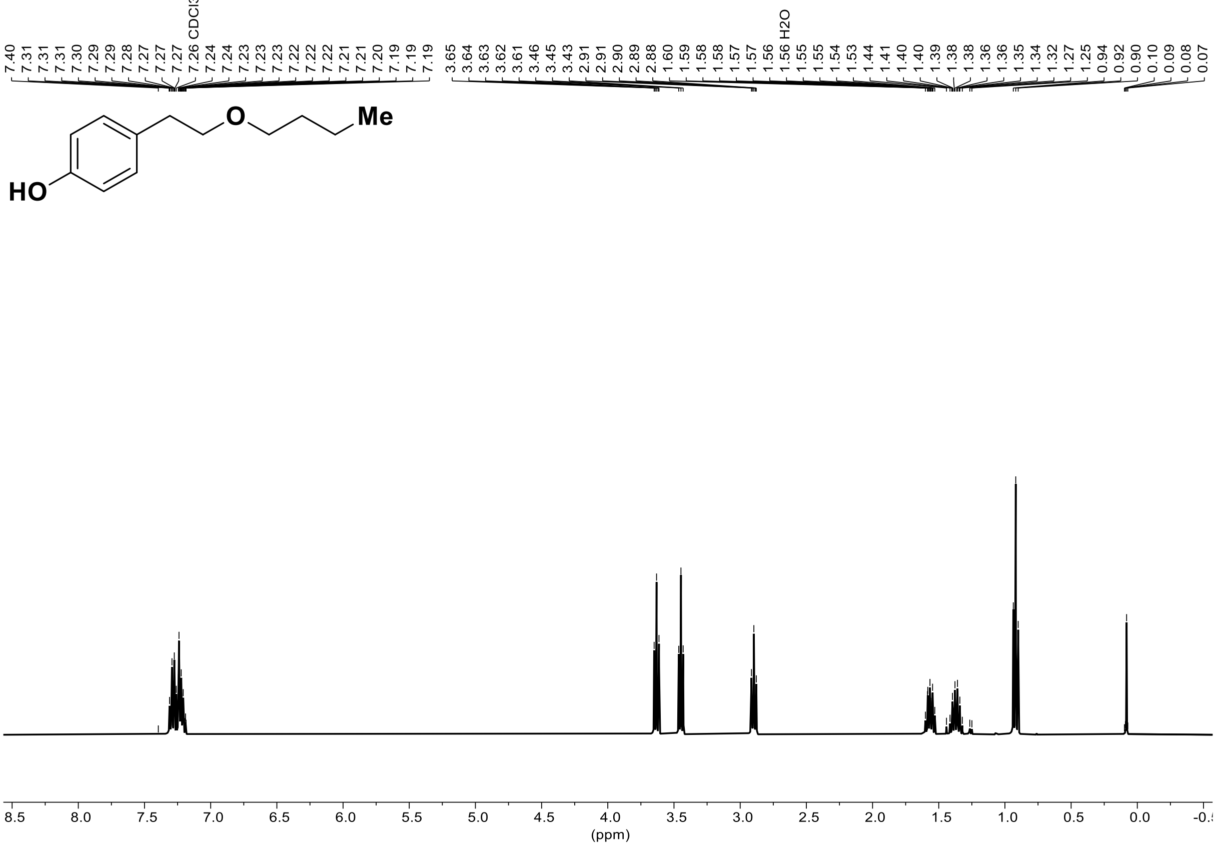

5r: ^{13}C, $CDCl_3$, 101 MHz

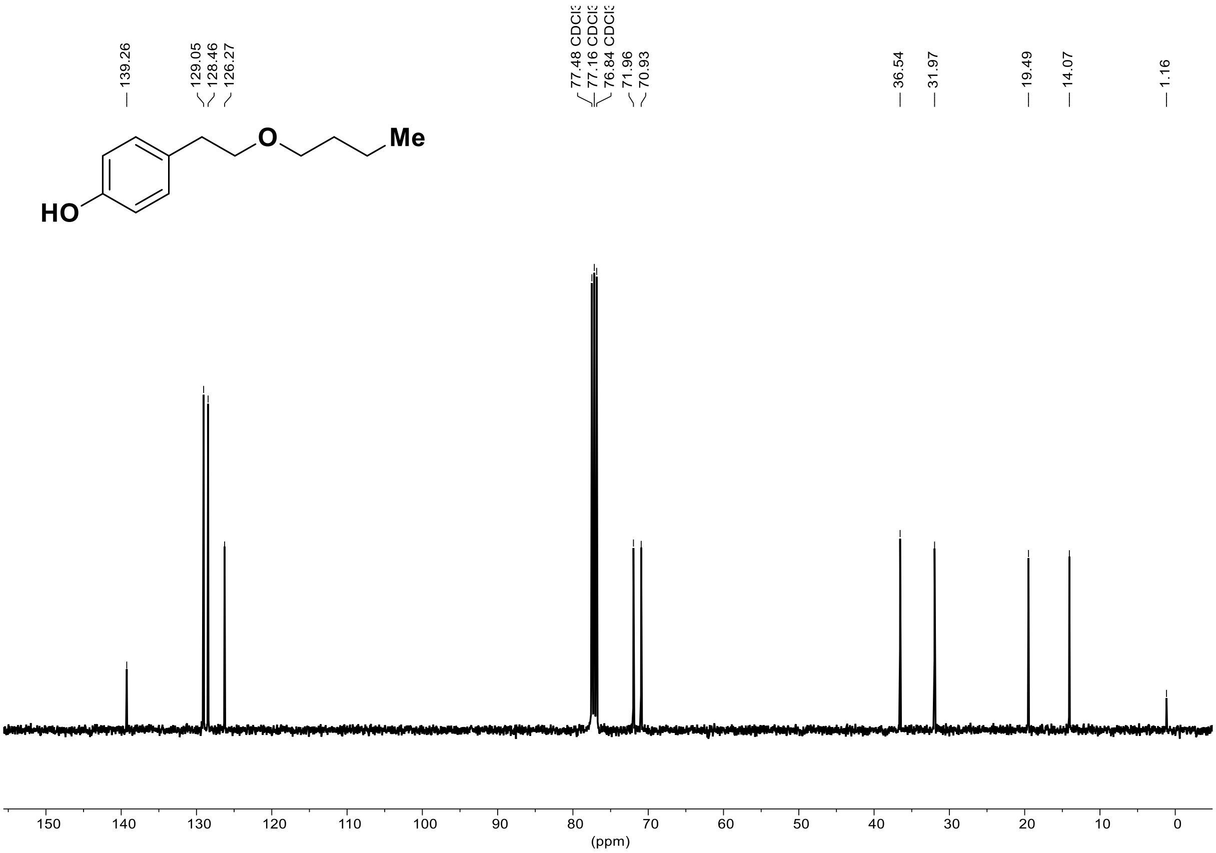

5s ^{1}H, $CDCl_3$, 400 MHz

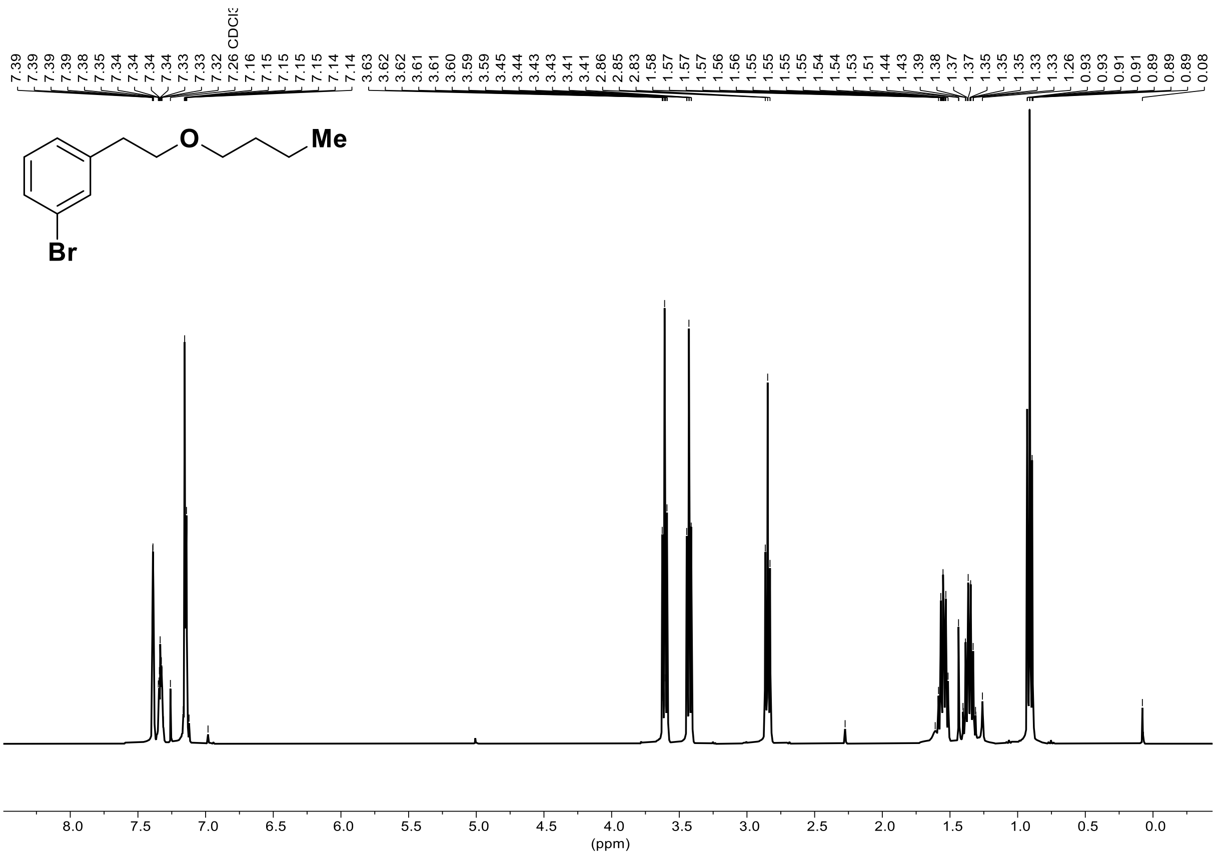

5s: ^{13}C, $CDCl_3$, 101 MHz

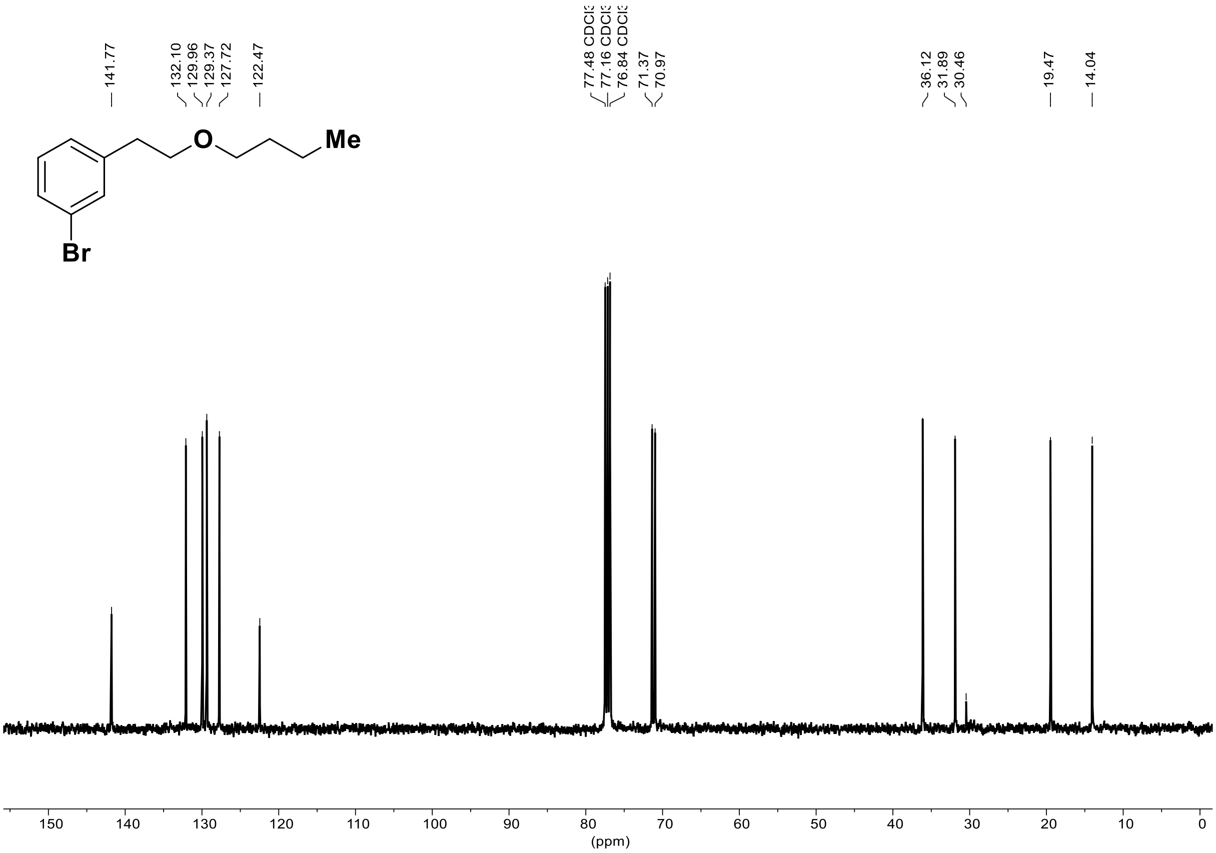

5t ^{1}H, $CDCl_3$, 400 MHz

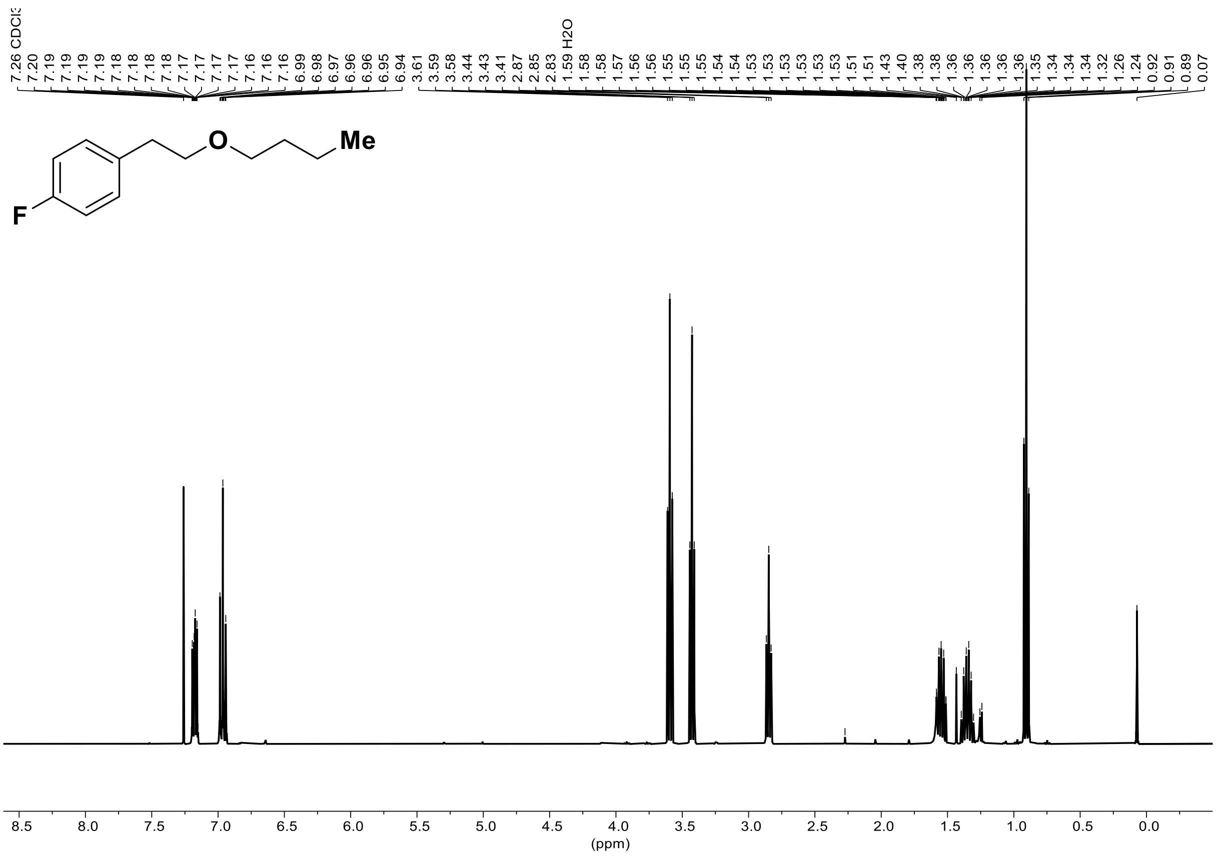

5t: ^{13}C, $CDCl_3$, 101 MHz

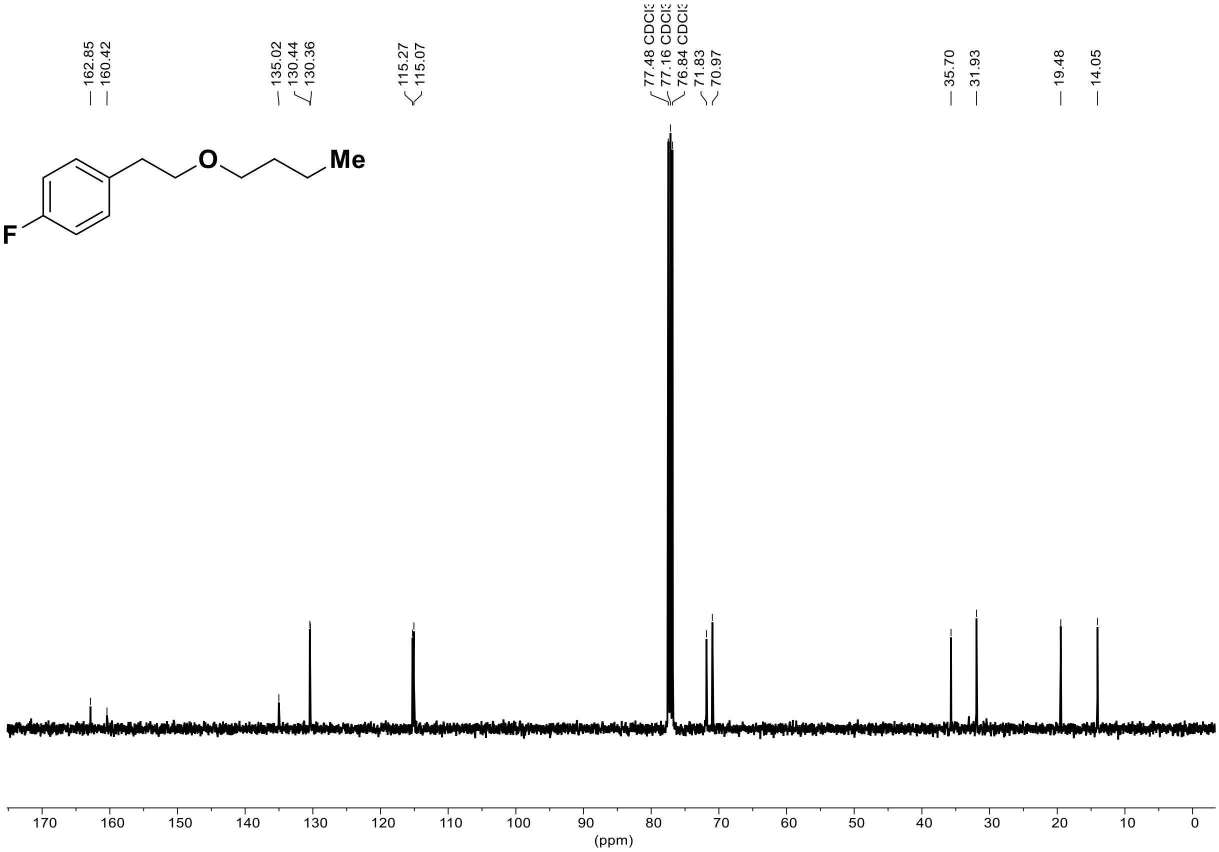

5t: ^{19}F, $CDCl_3$, 376 MHz

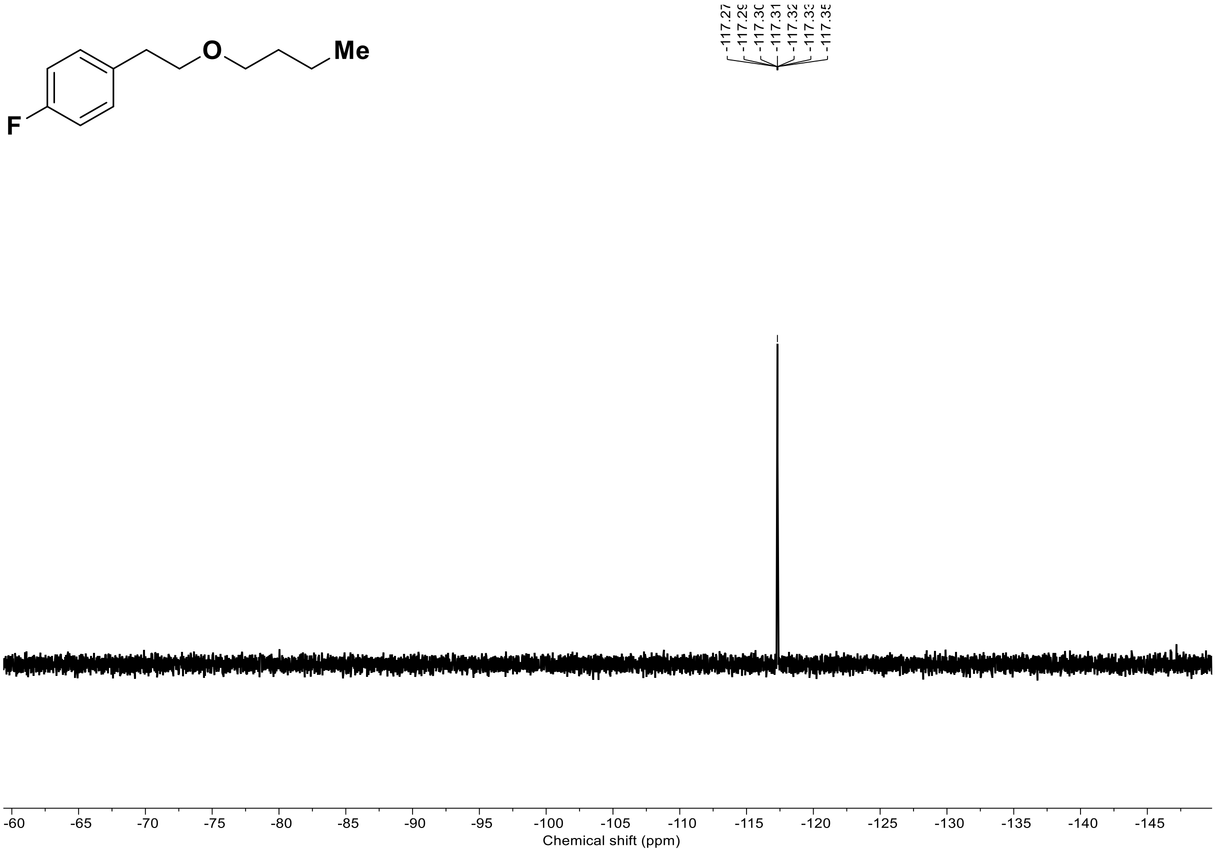

5u: ^{1}H, $CDCl_3$, 400 MHz

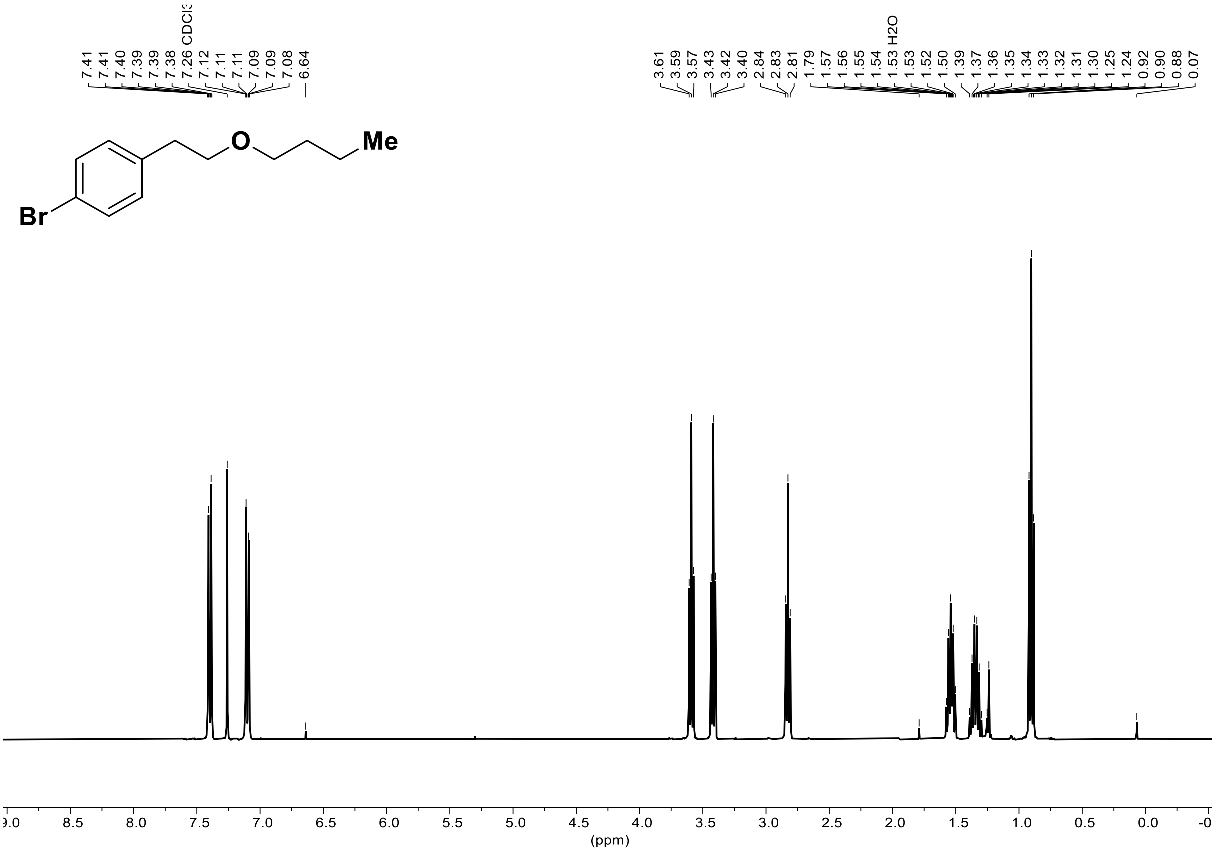

5u: ^{13}C, $CDCl_3$, 101 MHz

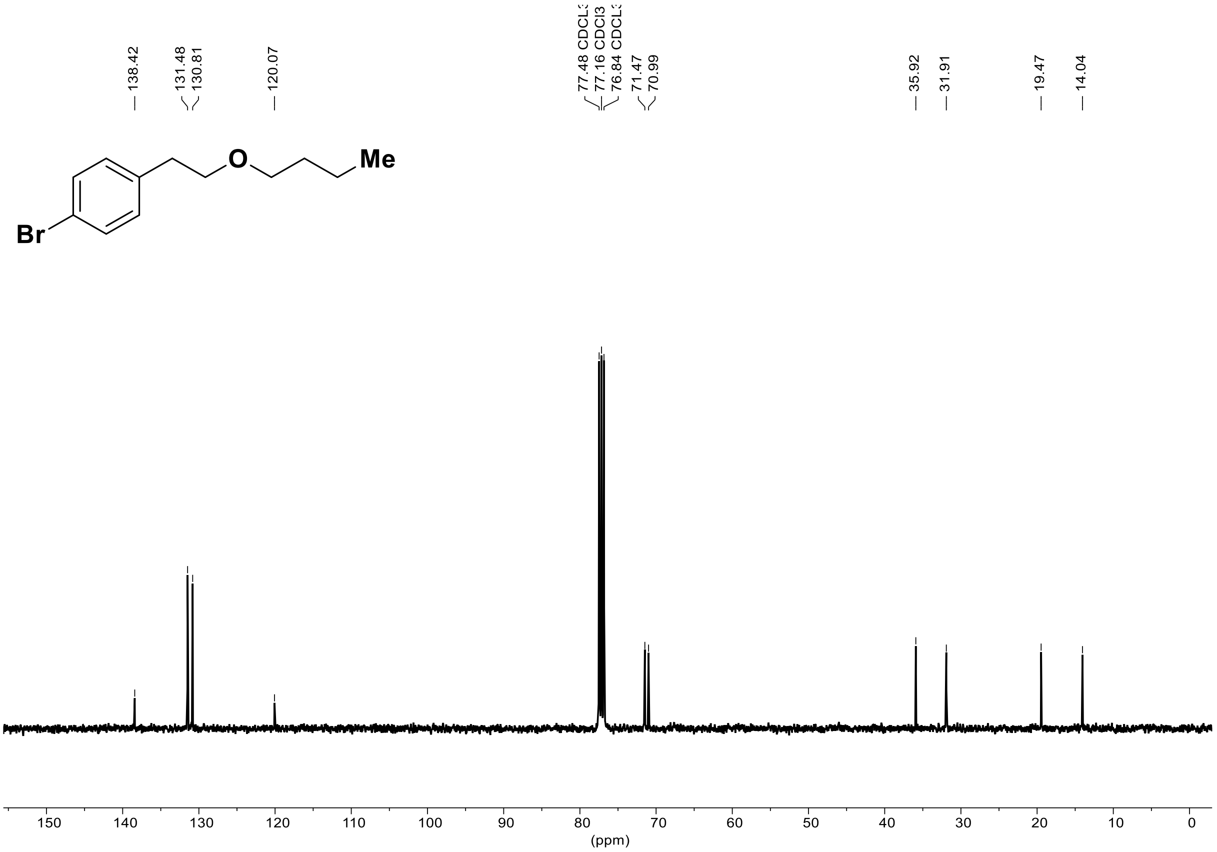

6a: ^{1}H, $CDCl_3$, 400 MHz

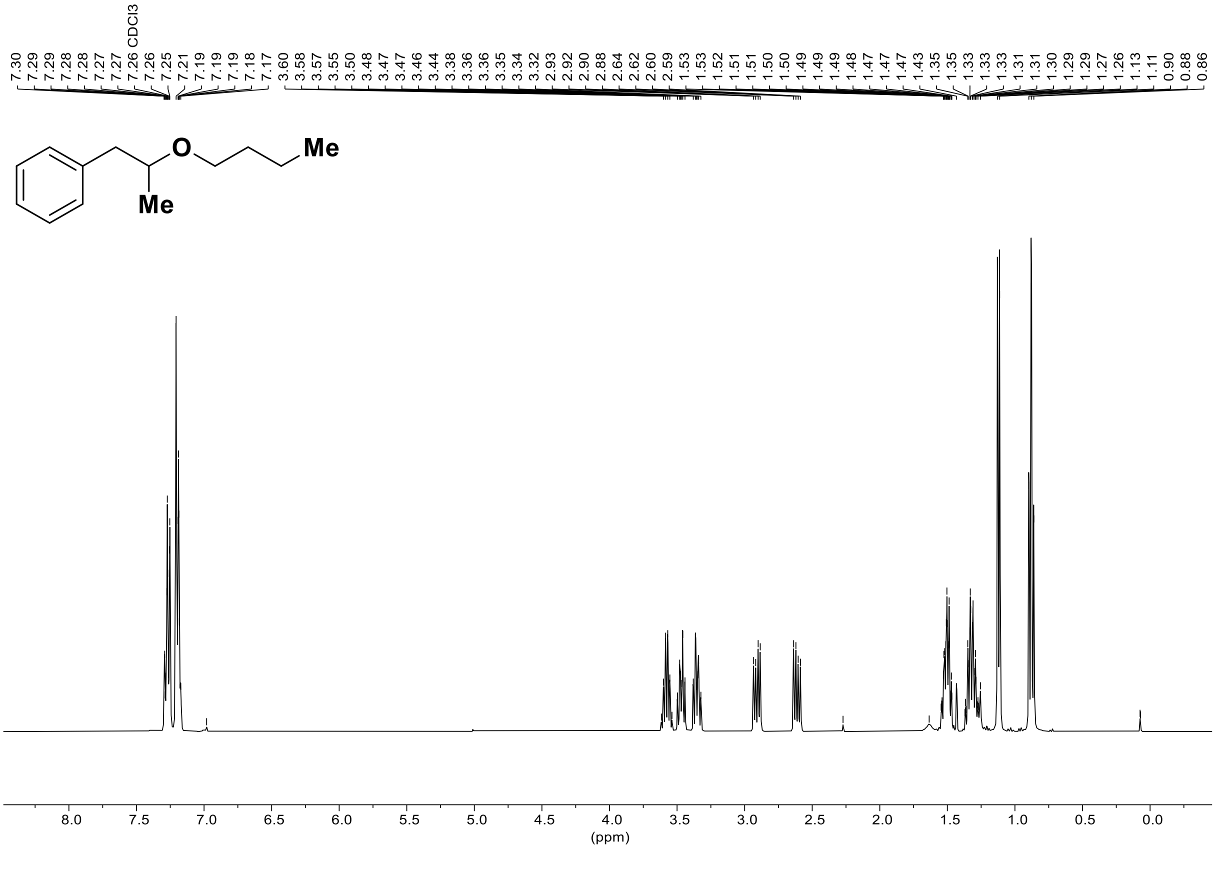

6a: ^{13}C, $CDCl_3$, 101 MHz

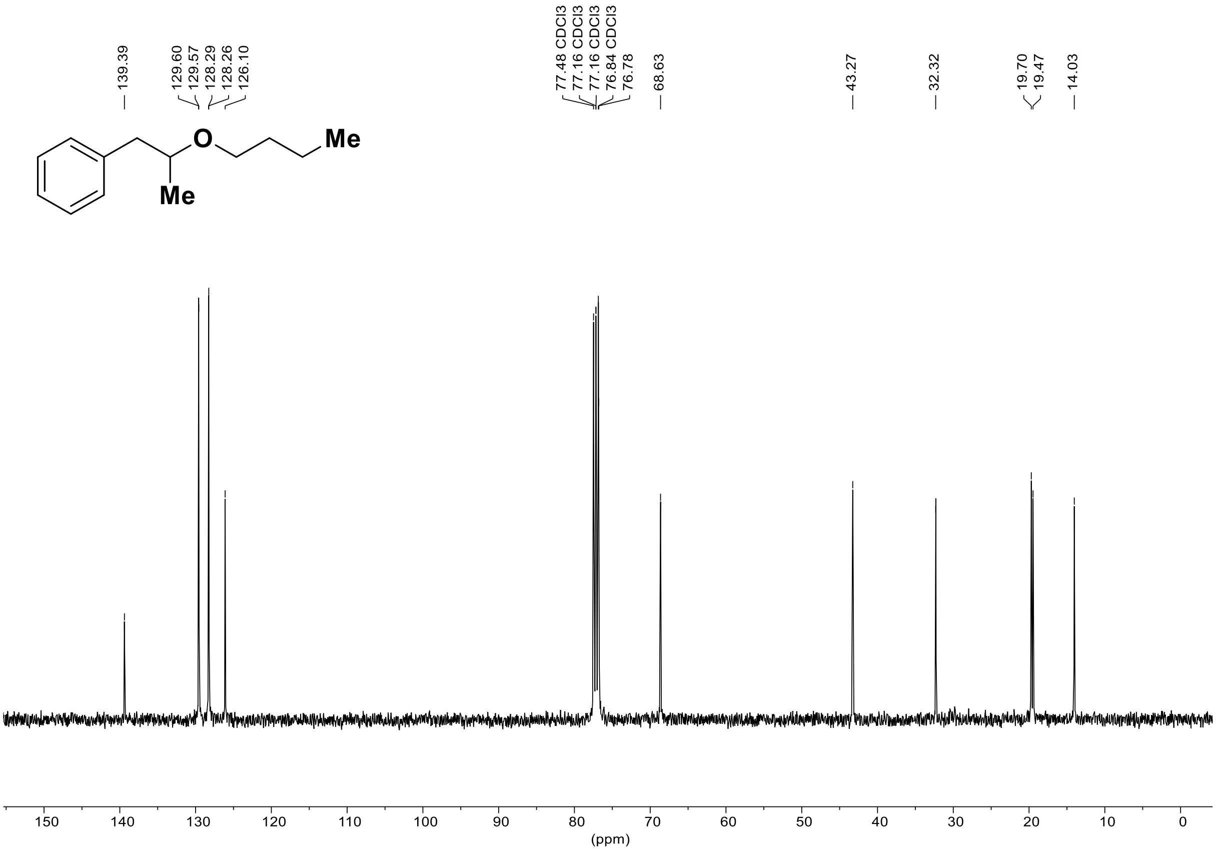

6b: ^{1}H, $CDCl_3$, 400 MHz

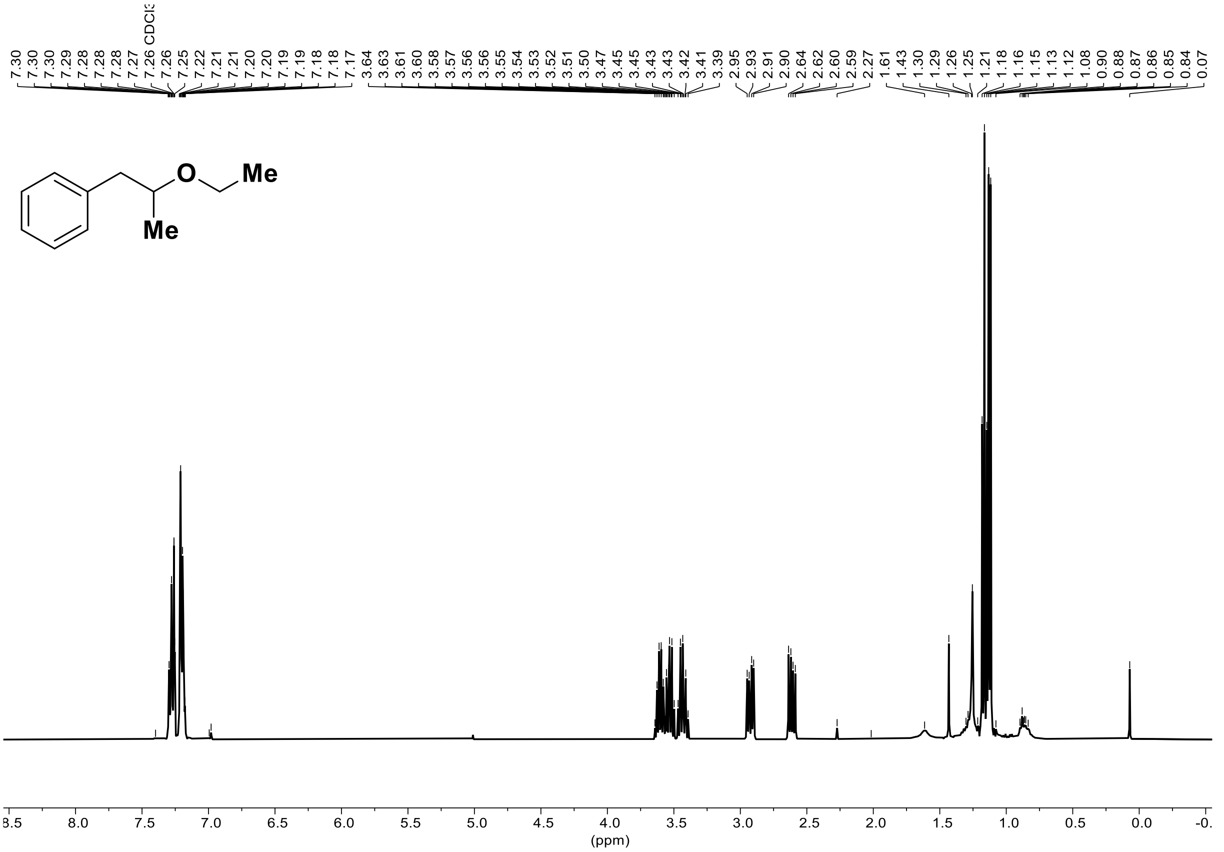

6b: ^{13}C, $CDCl_3$, 101 MHz

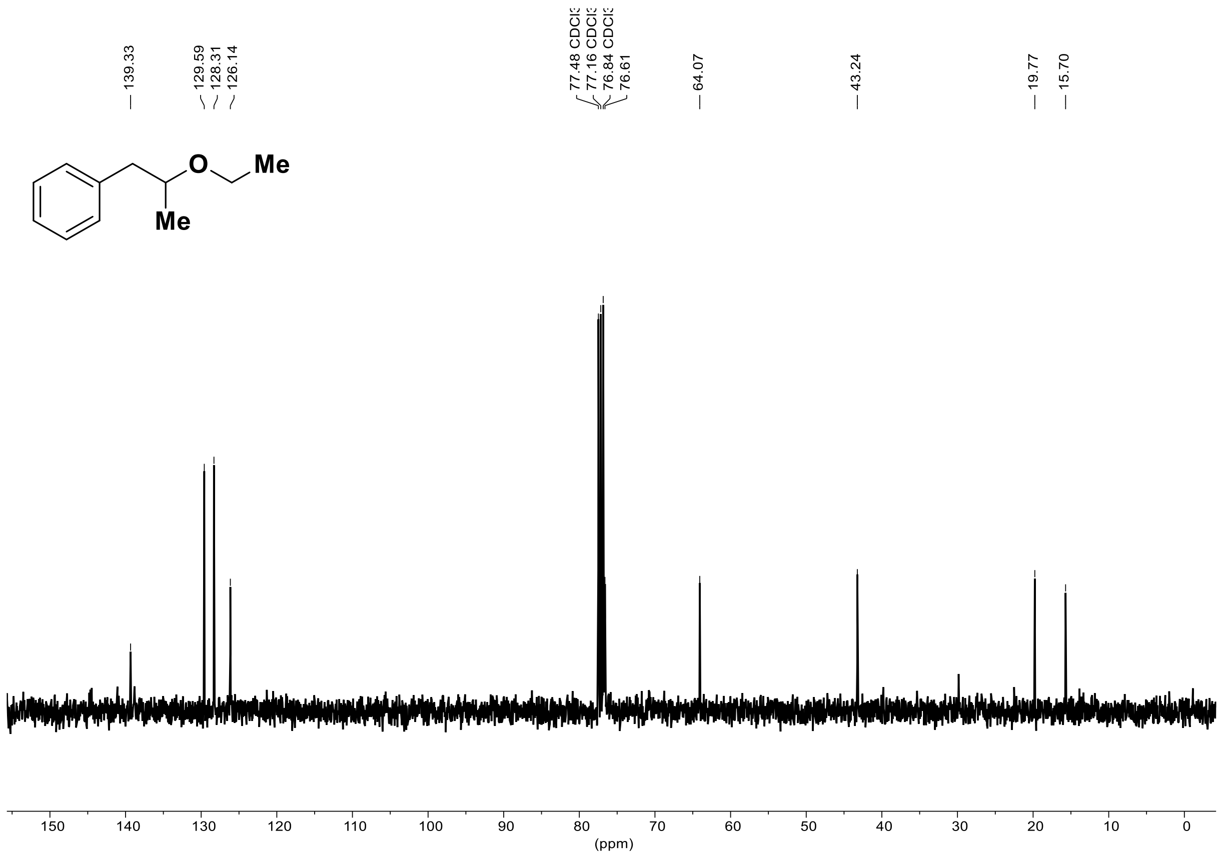

6c: ^{1}H, $CDCl_3$, 400 MHz

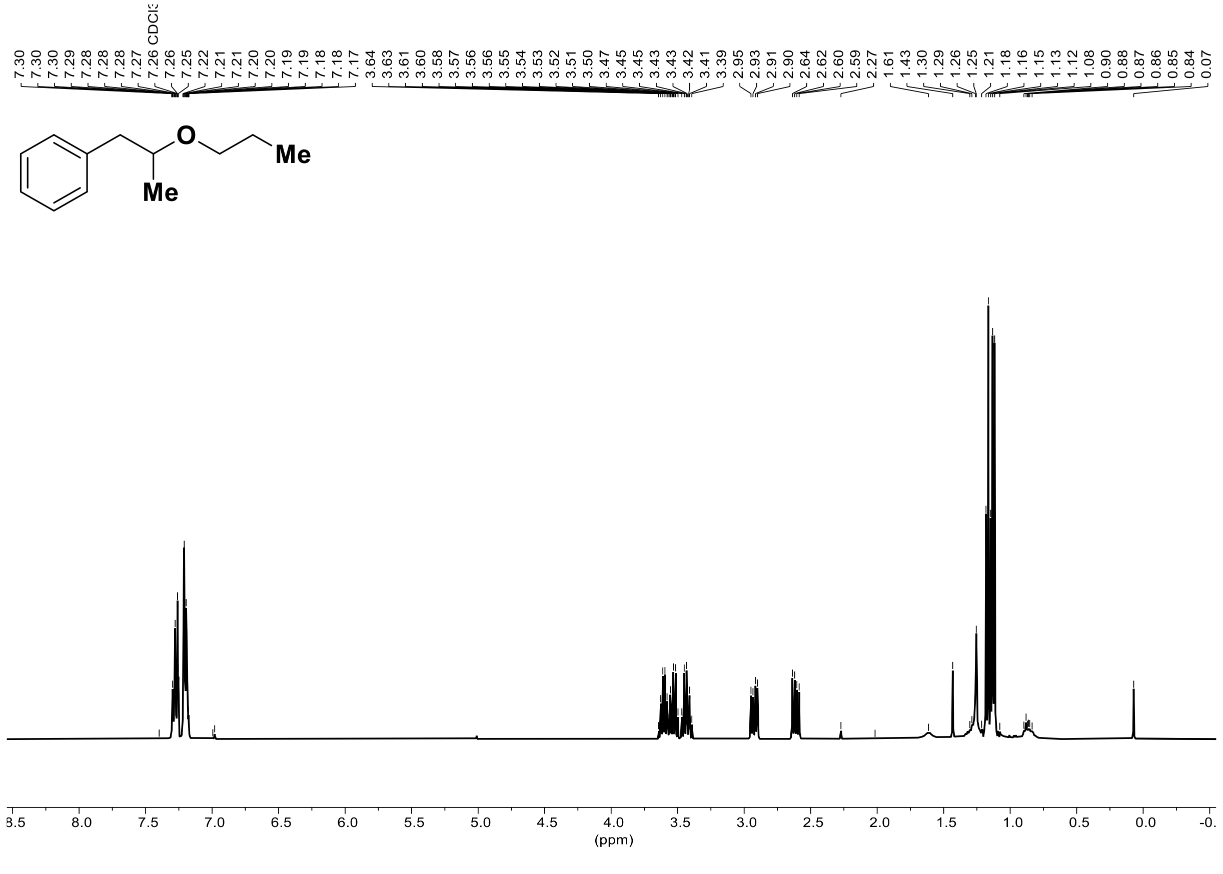

6c: ^{13}C, $CDCl_3$, 101 MHz

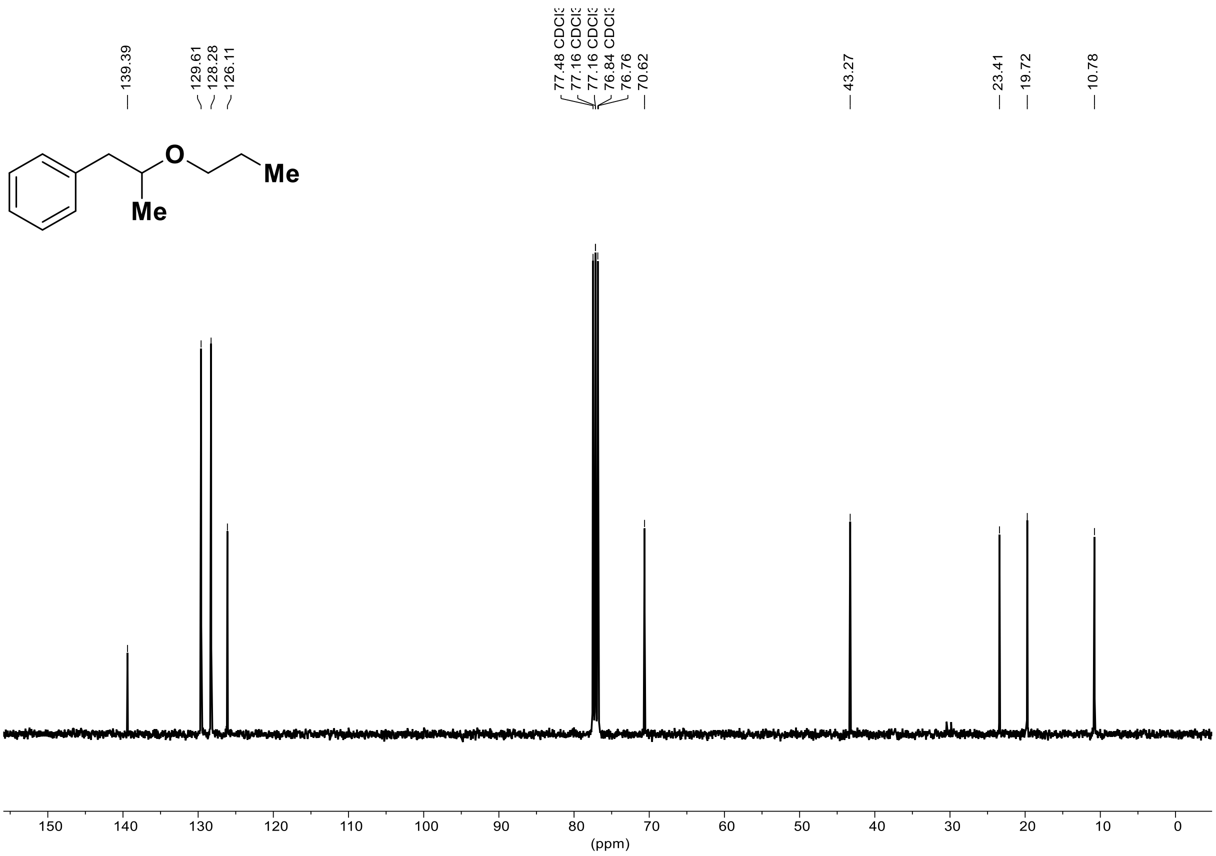

6d: ^{1}H, $CDCl_3$, 400 MHz

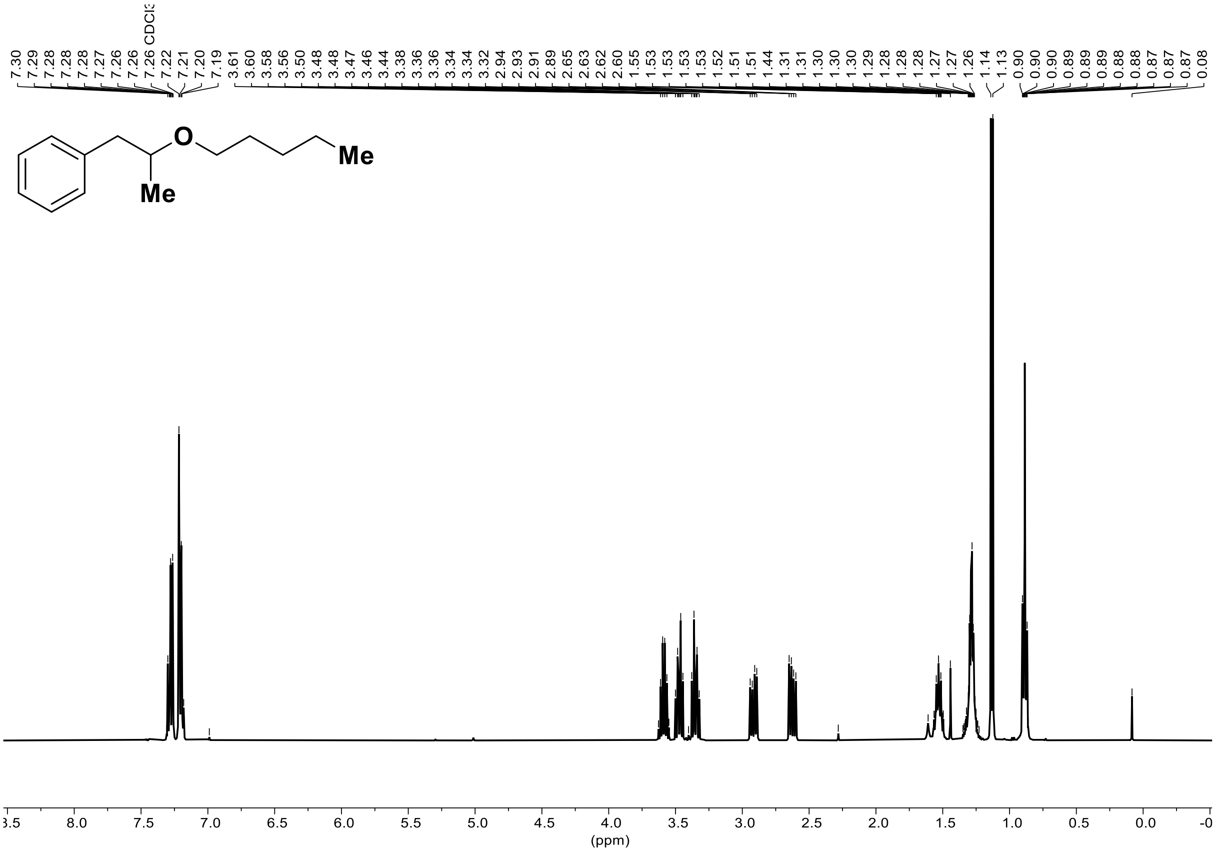

6d: ^{13}C, $CDCl_3$, 101 MHz

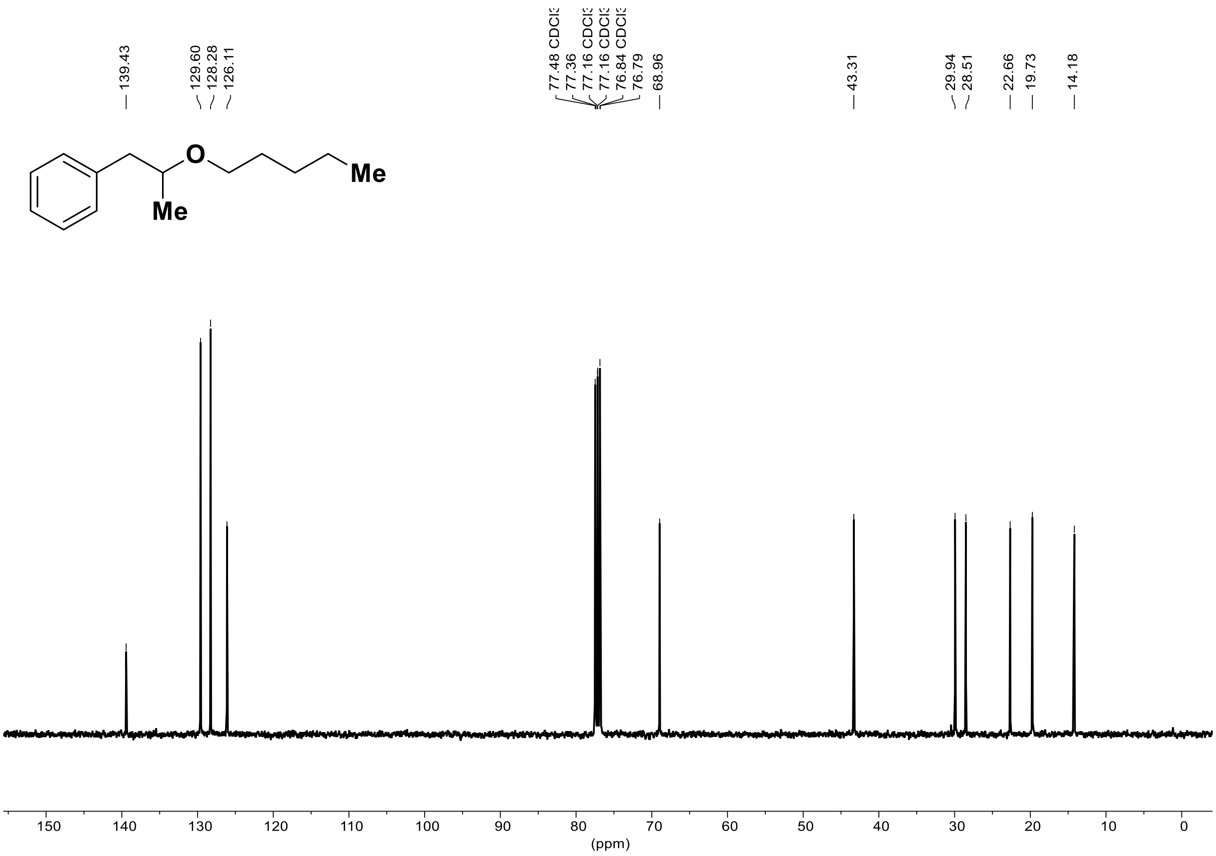

6f: ^{1}H, $CDCl_3$, 400 MHz

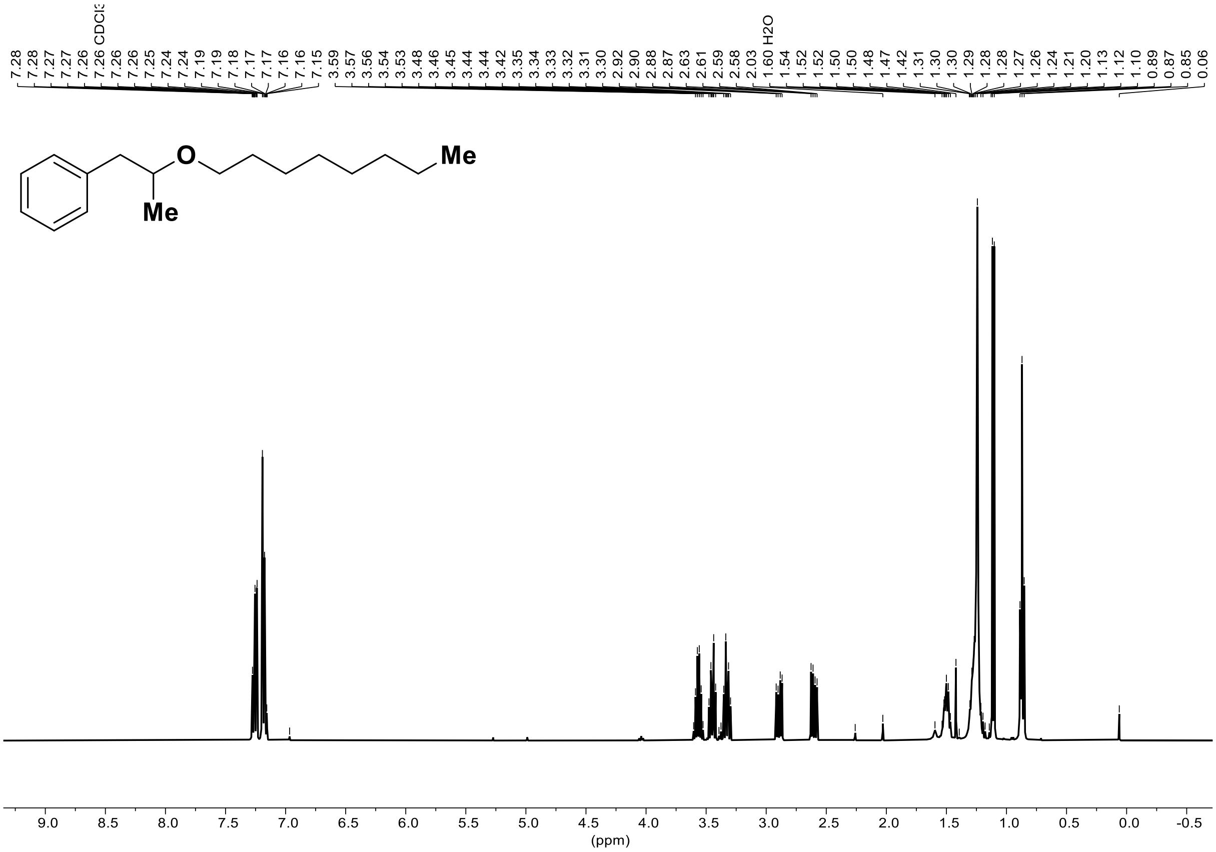

6f: ^{13}C, $CDCl_3$, 101 MHz

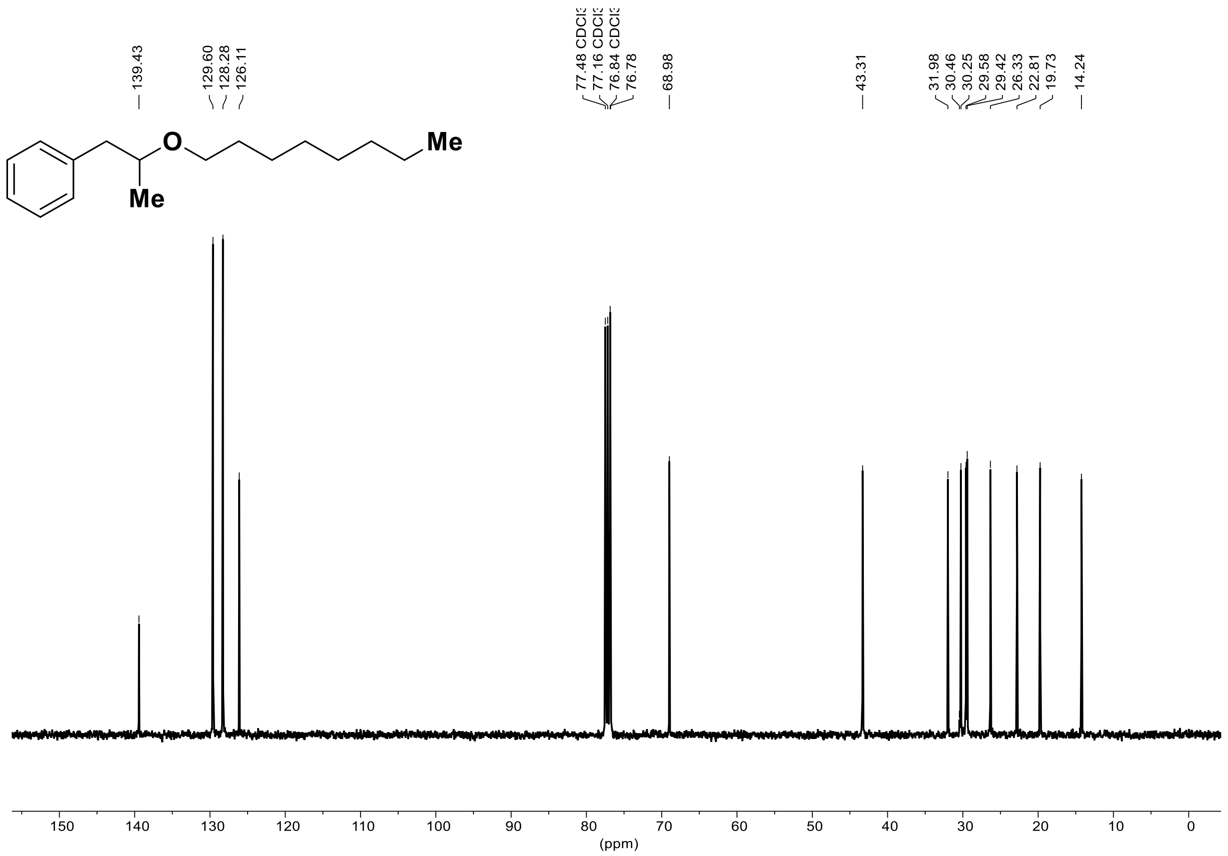

6h: ^{1}H, $CDCl_3$, 400 MHz

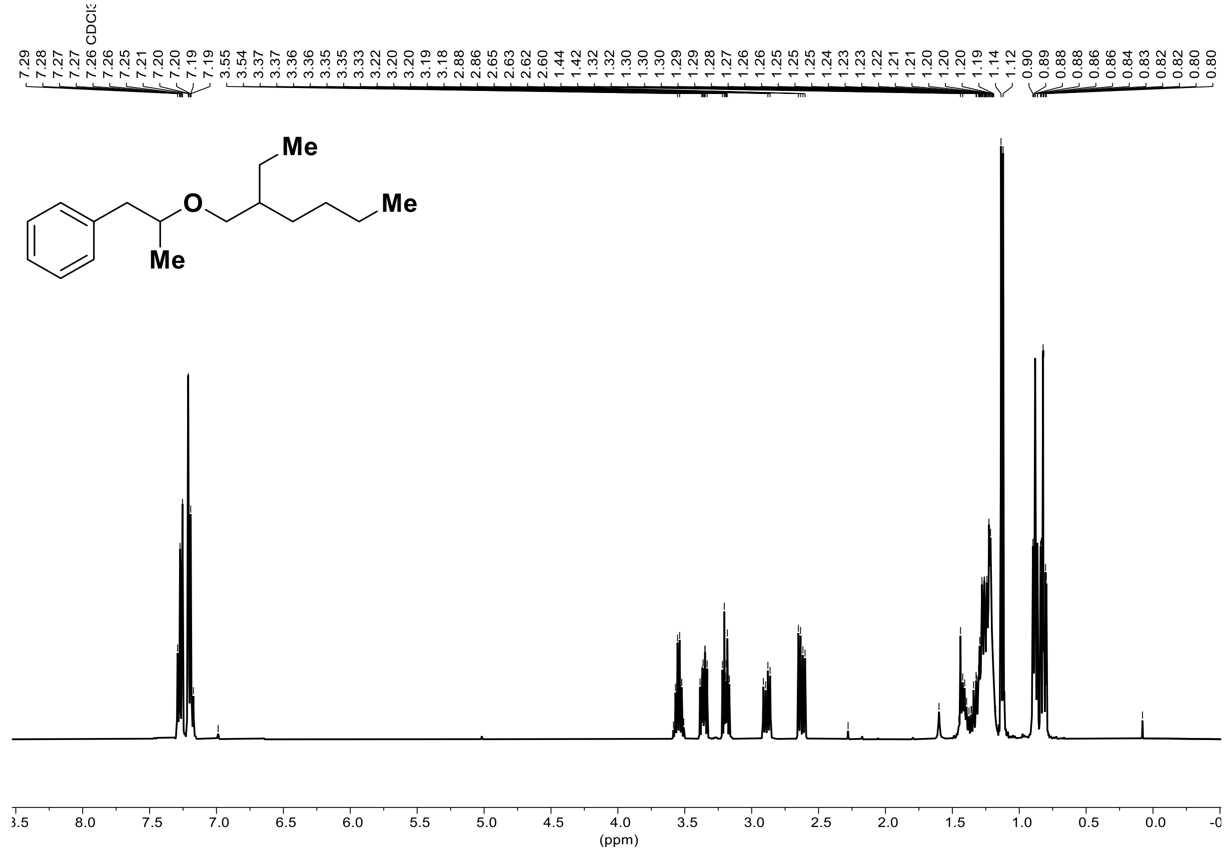

6h: ^{13}C, $CDCl_3$, 101 MHz

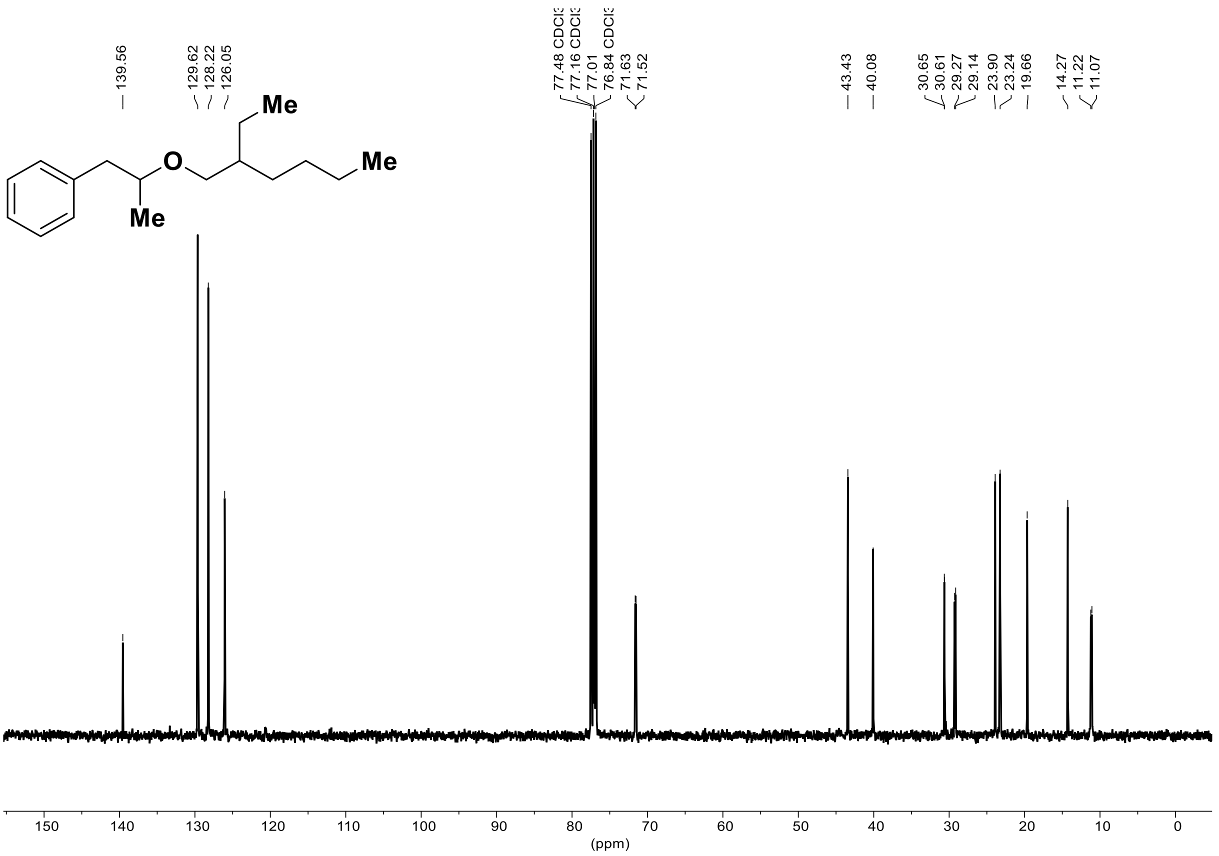

6i: ^{1}H, $CDCl_3$, 400 MHz

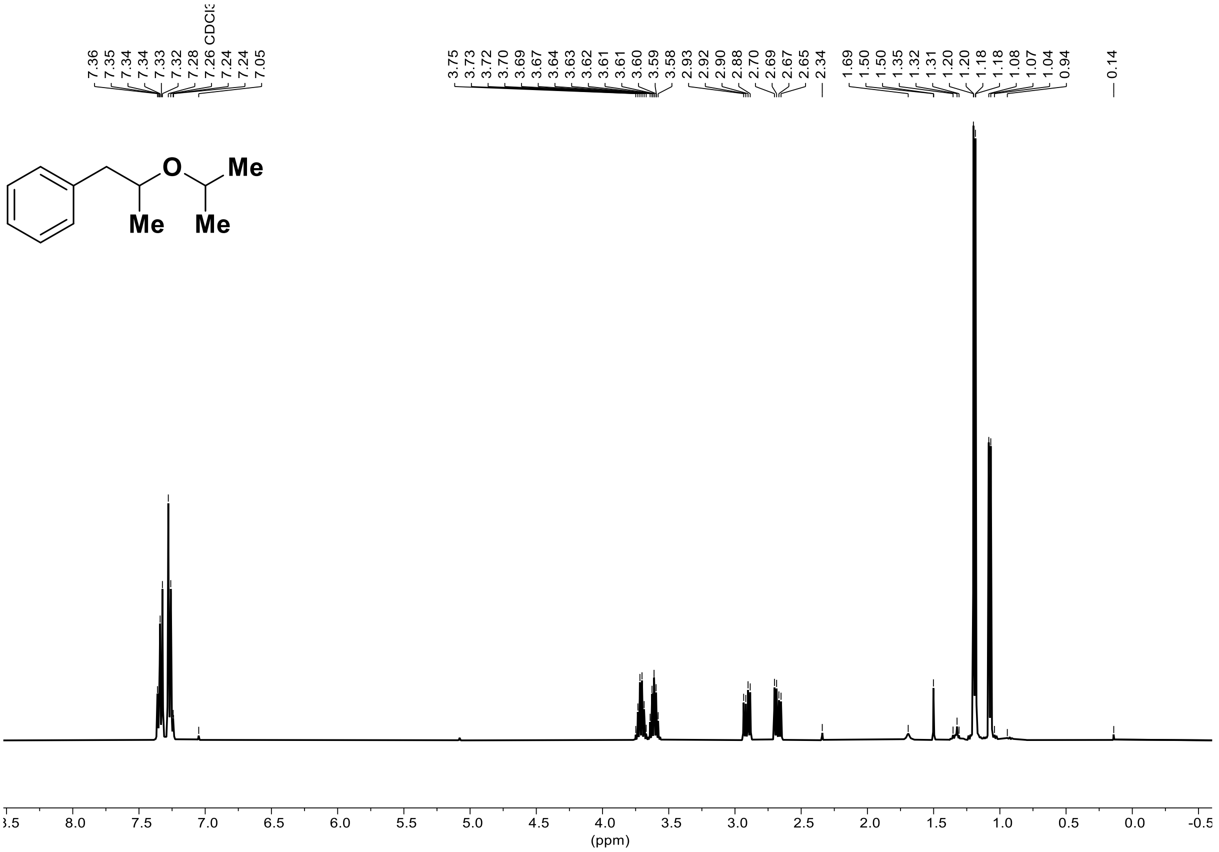

6i: ^{13}C, $CDCl_3$, 101 MHz

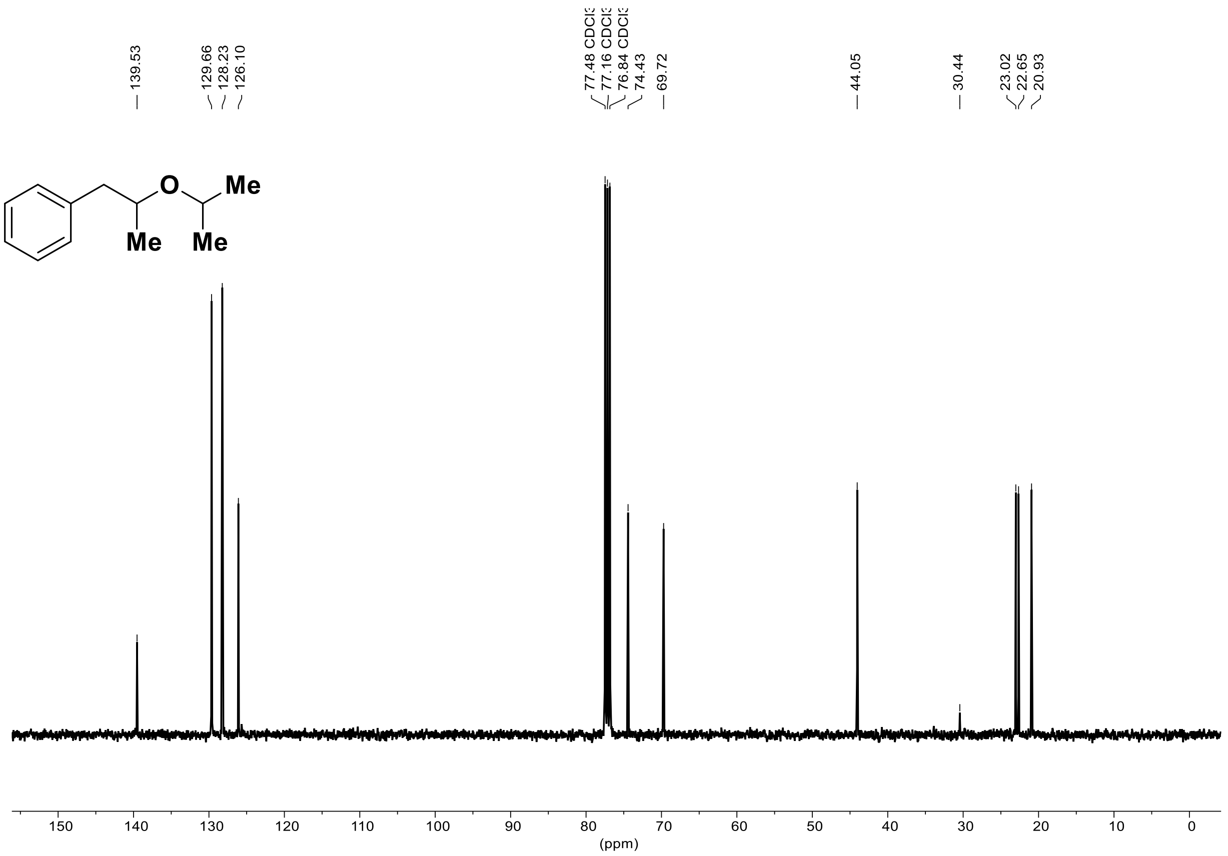

6m: ^{1}H, $CDCl_3$, 400 MHz

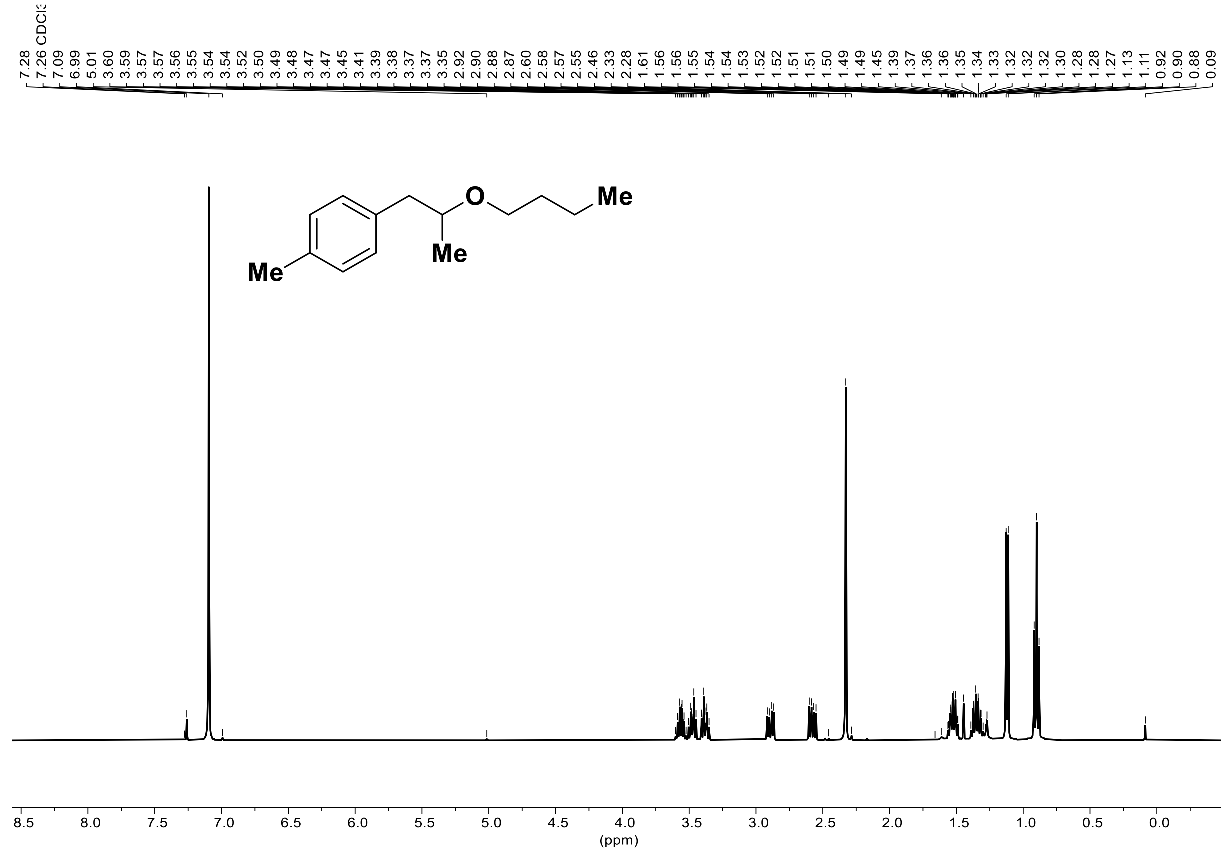

6m: ^{13}C, $CDCl_3$, 101 MHz

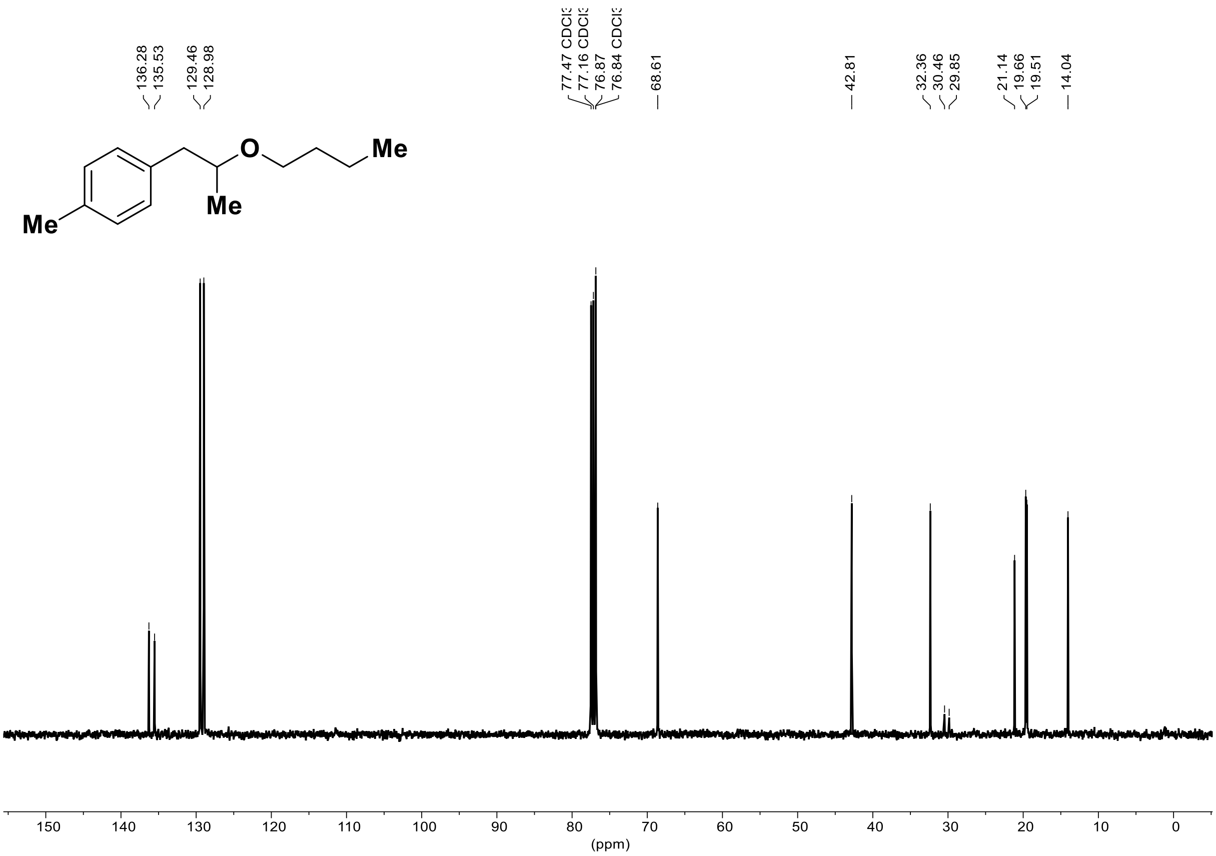

6n: ^{1}H, $CDCl_3$, 400 MHz

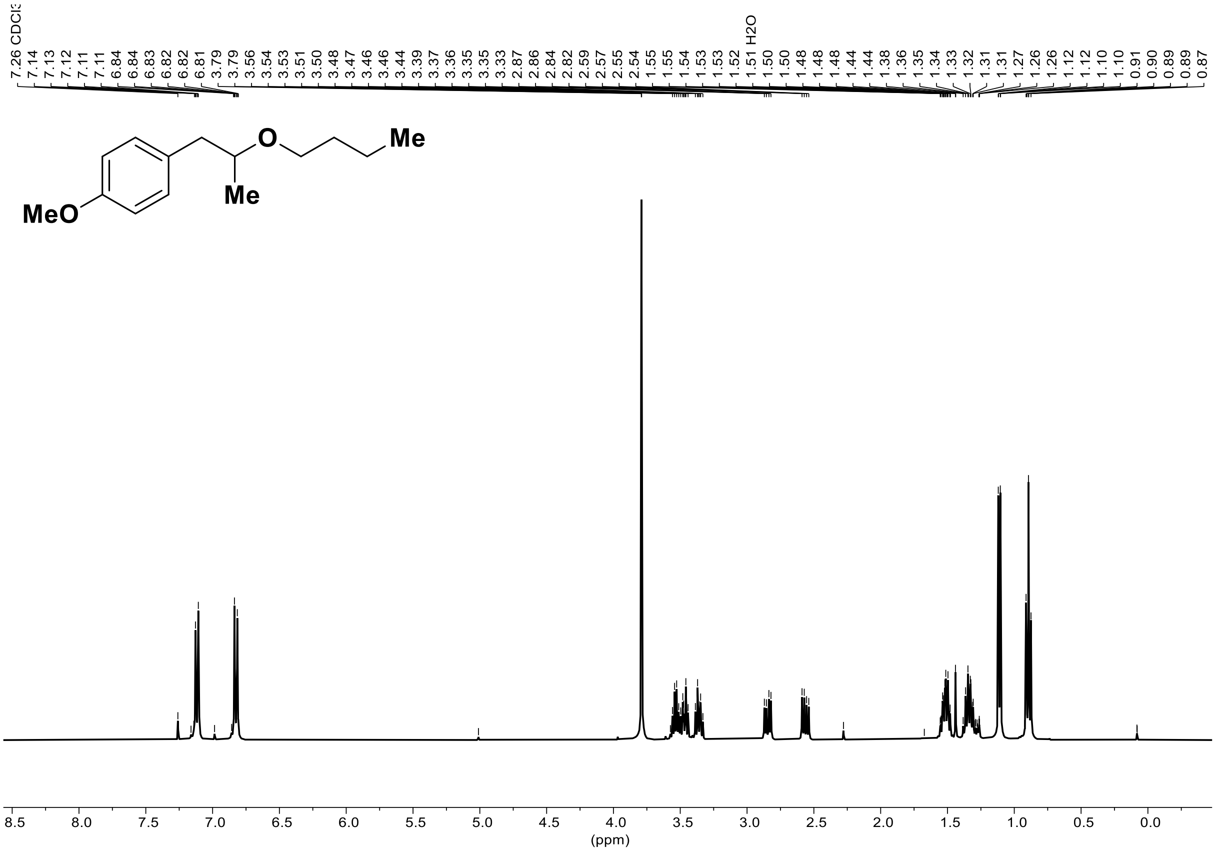

6n: ^{13}C, $CDCl_3$, 101 MHz

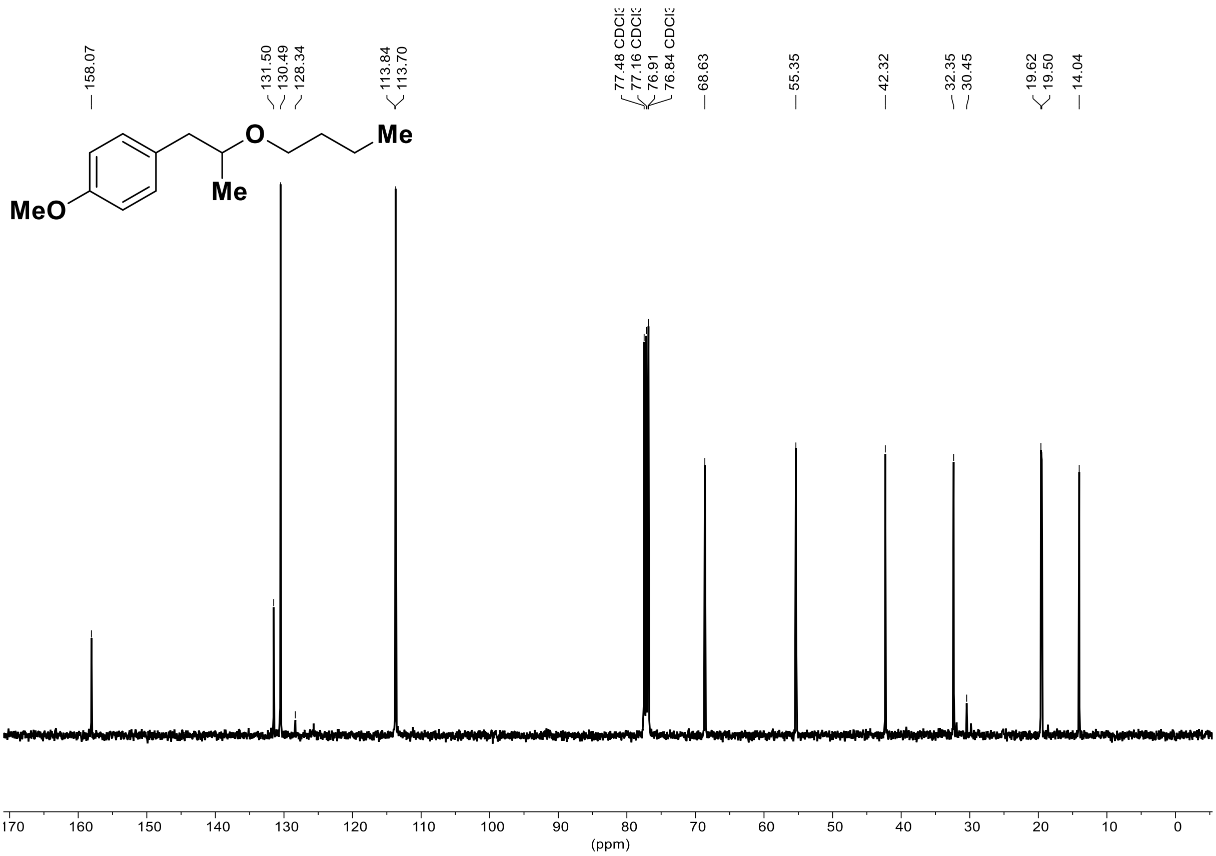

6o: ^{1}H, $CDCl_3$, 400 MHz

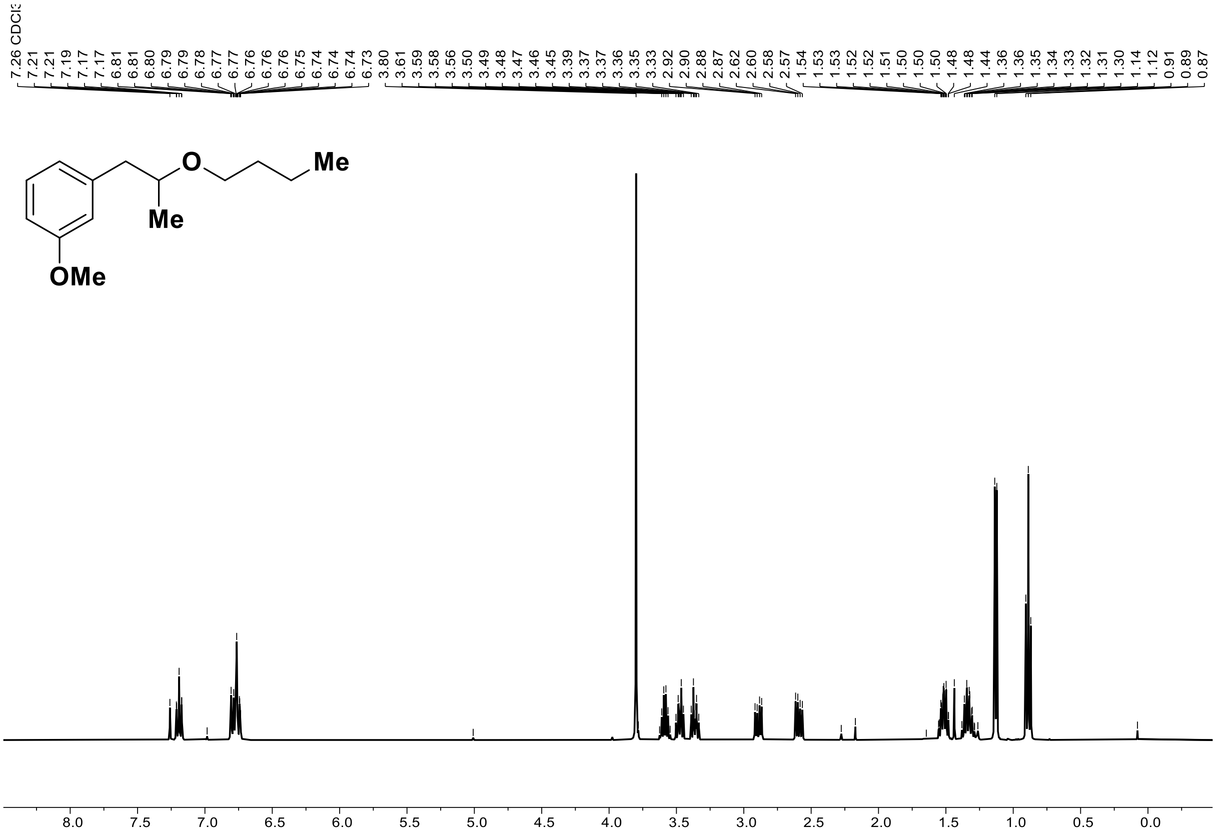

6o: ^{13}C, $CDCl_3$, 101 MHz

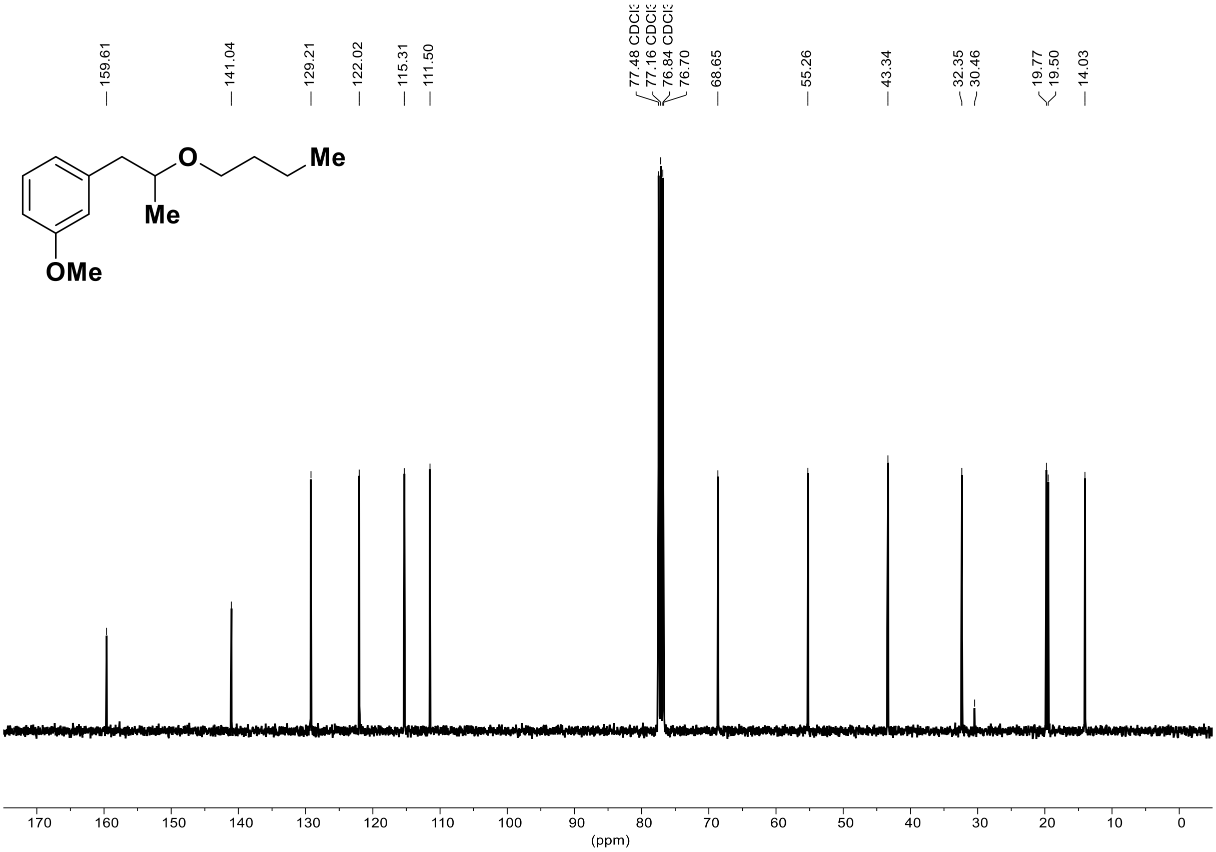

6p: ^{1}H, $CDCl_3$, 400 MHz

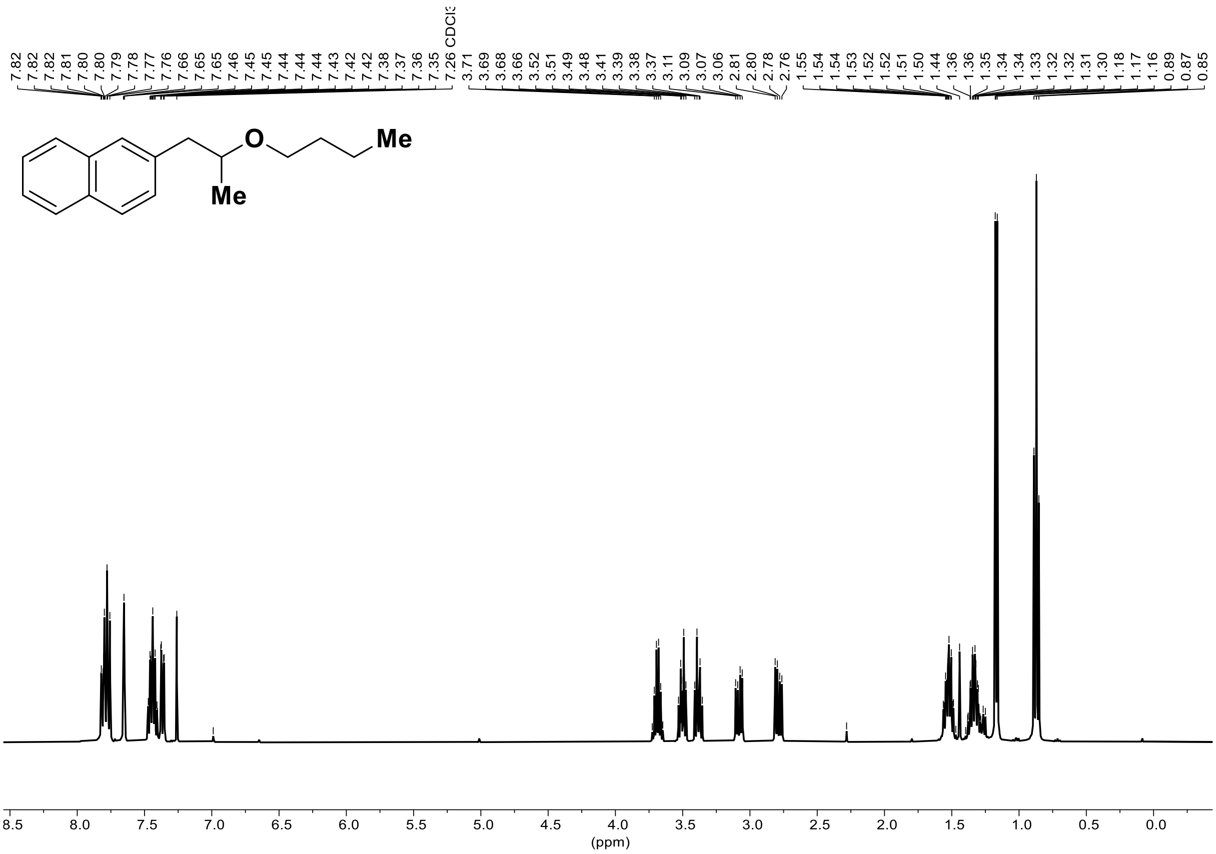

6p: ^{13}C, $CDCl_3$, 101 MHz

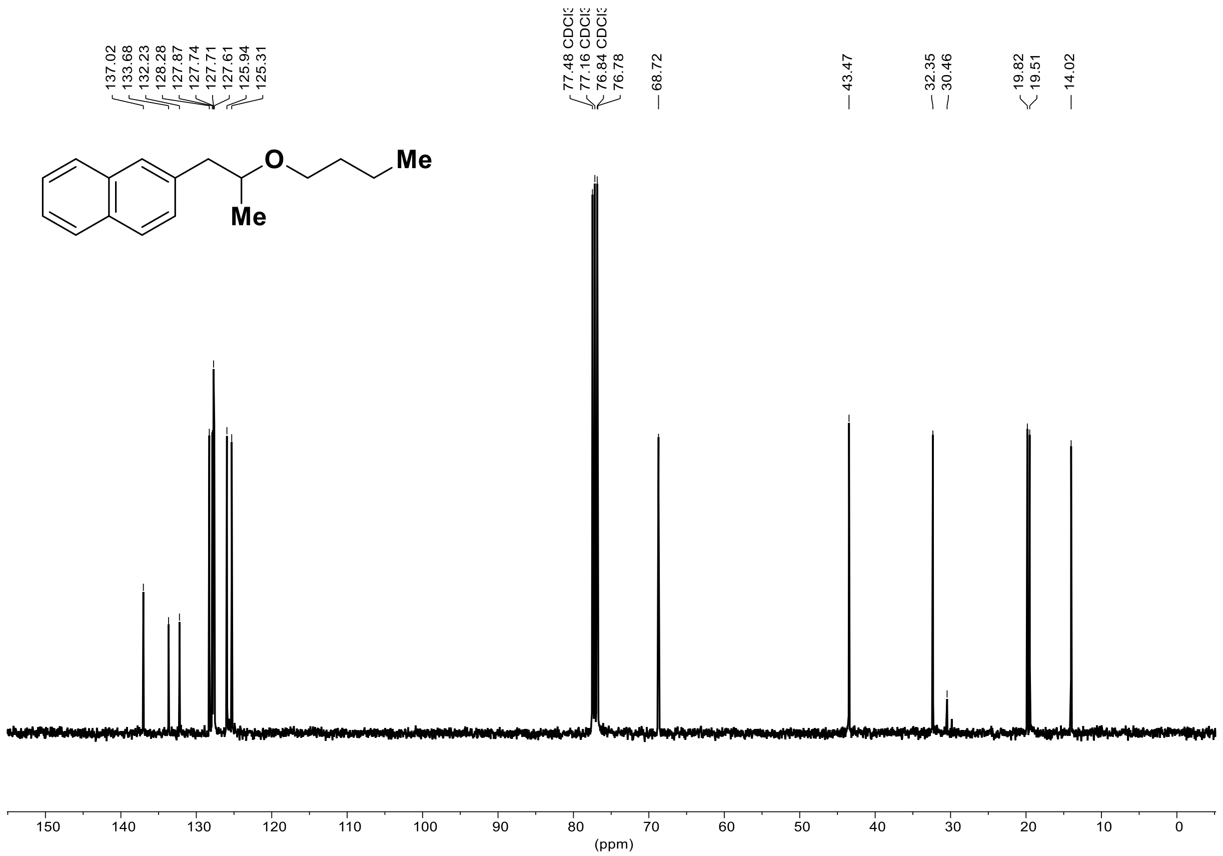

6q: ^{1}H, $CDCl_3$, 400 MHz

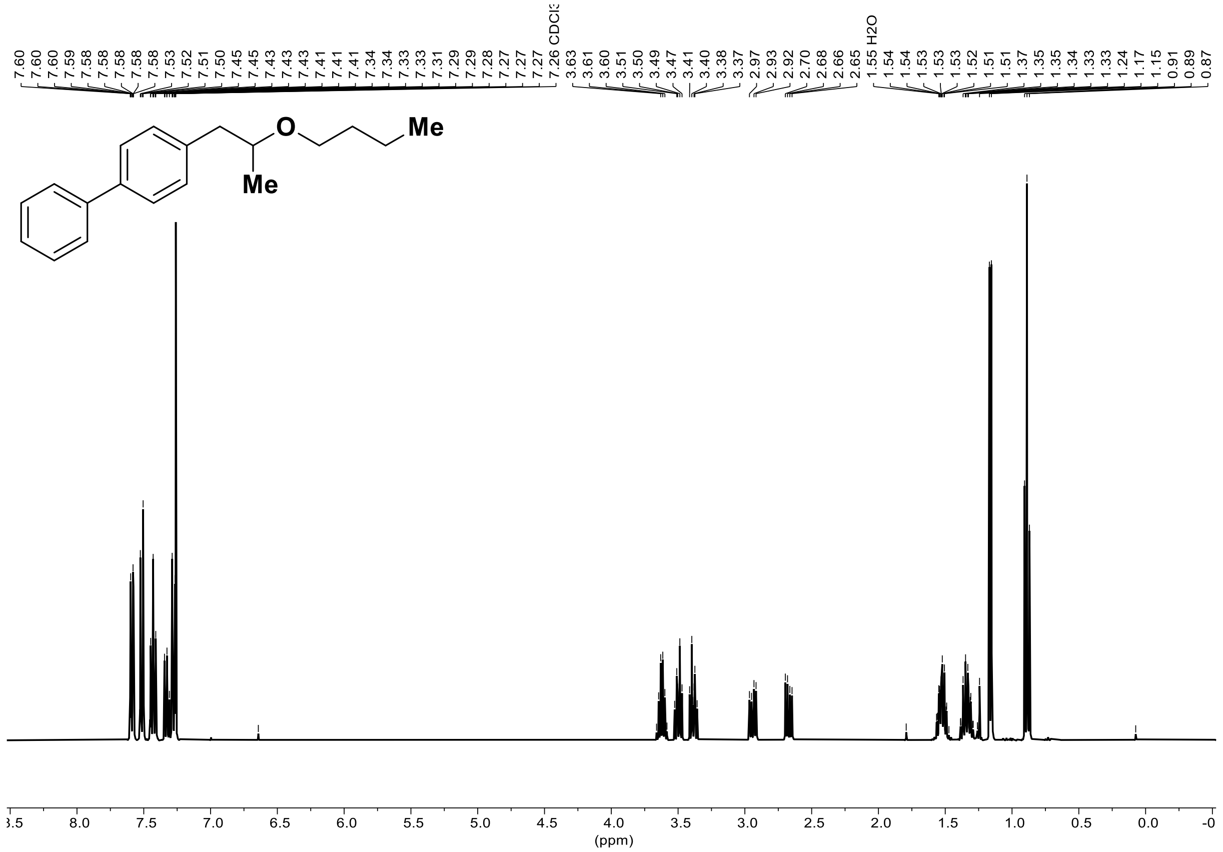

6q: ^{13}C, $CDCl_3$, 101 MHz

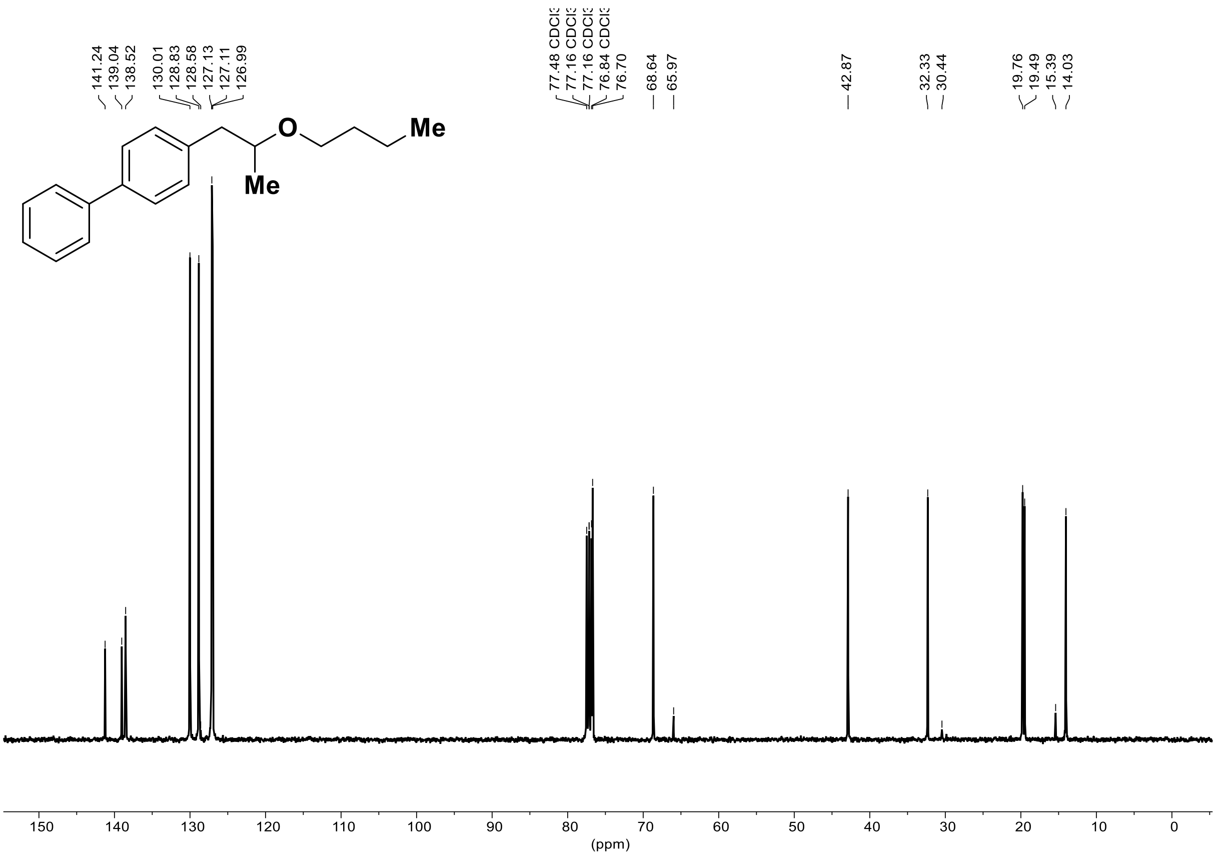

6r: ^{1}H, $CDCl_3$, 400 MHz

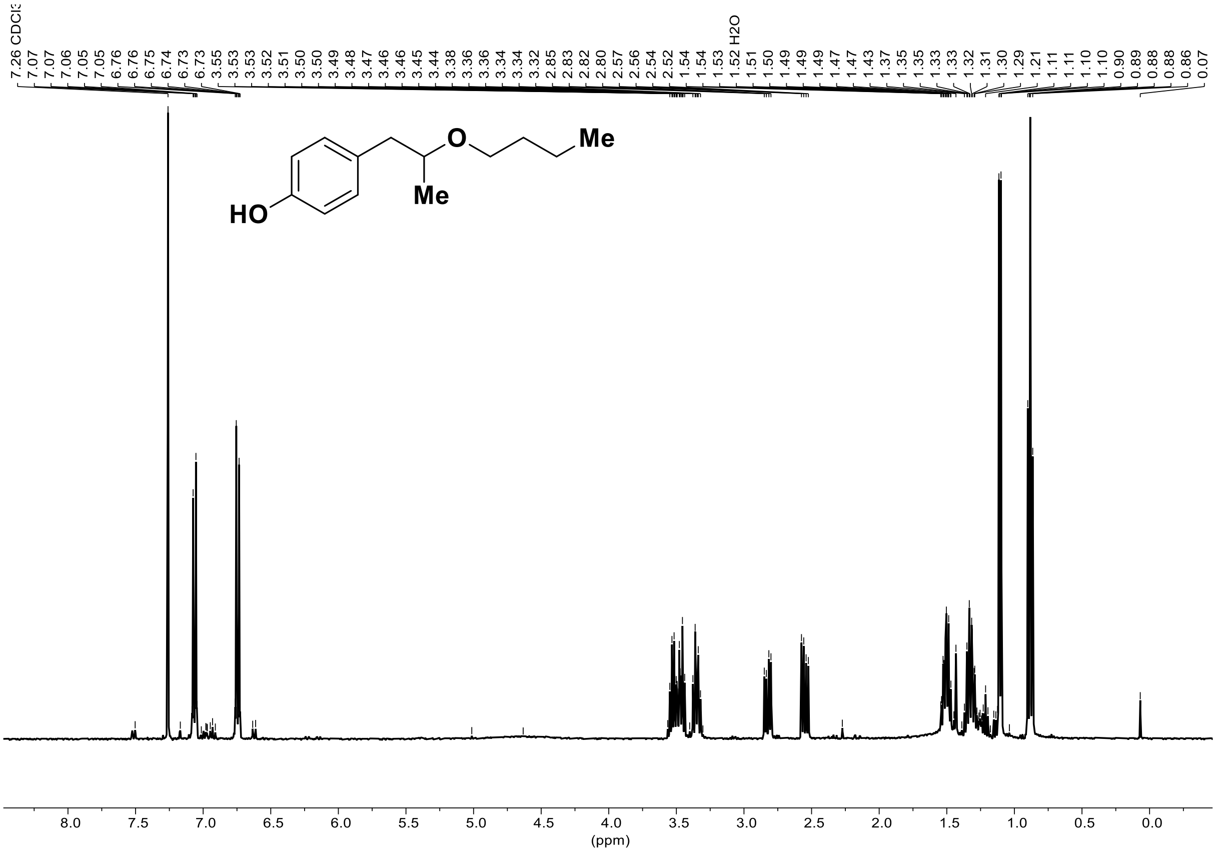

6r: ^{13}C, $CDCl_3$, 101 MHz

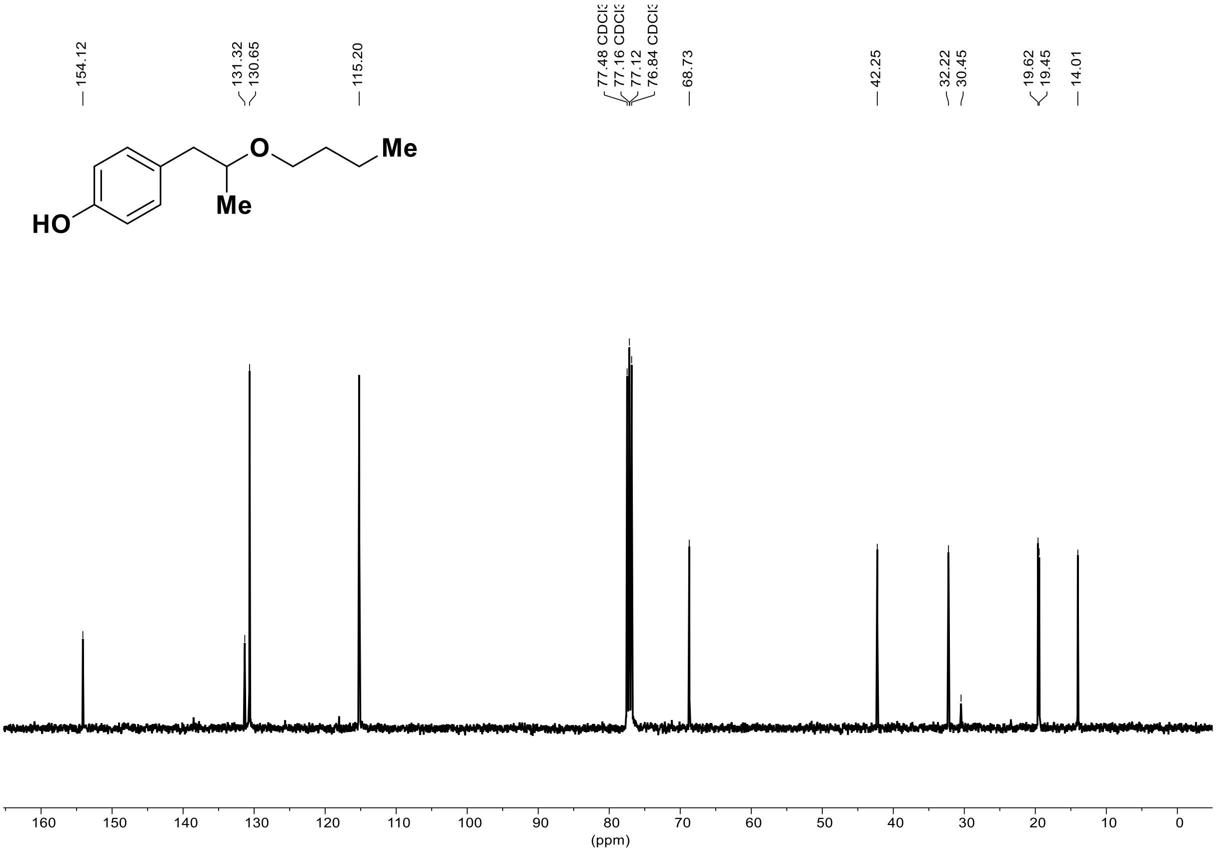

6s: ^{1}H, $CDCl_3$, 400 MHz

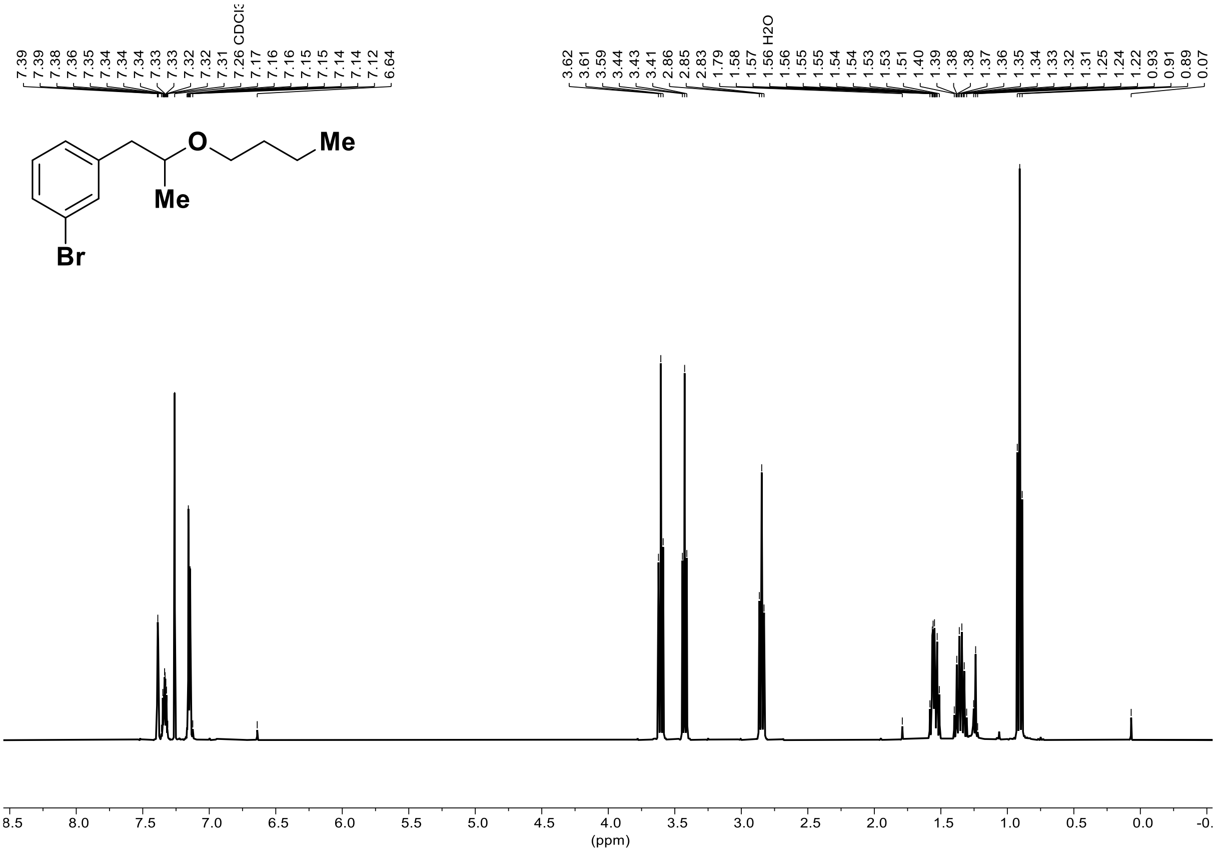

6s: ^{13}C, $CDCl_3$, 101 MHz

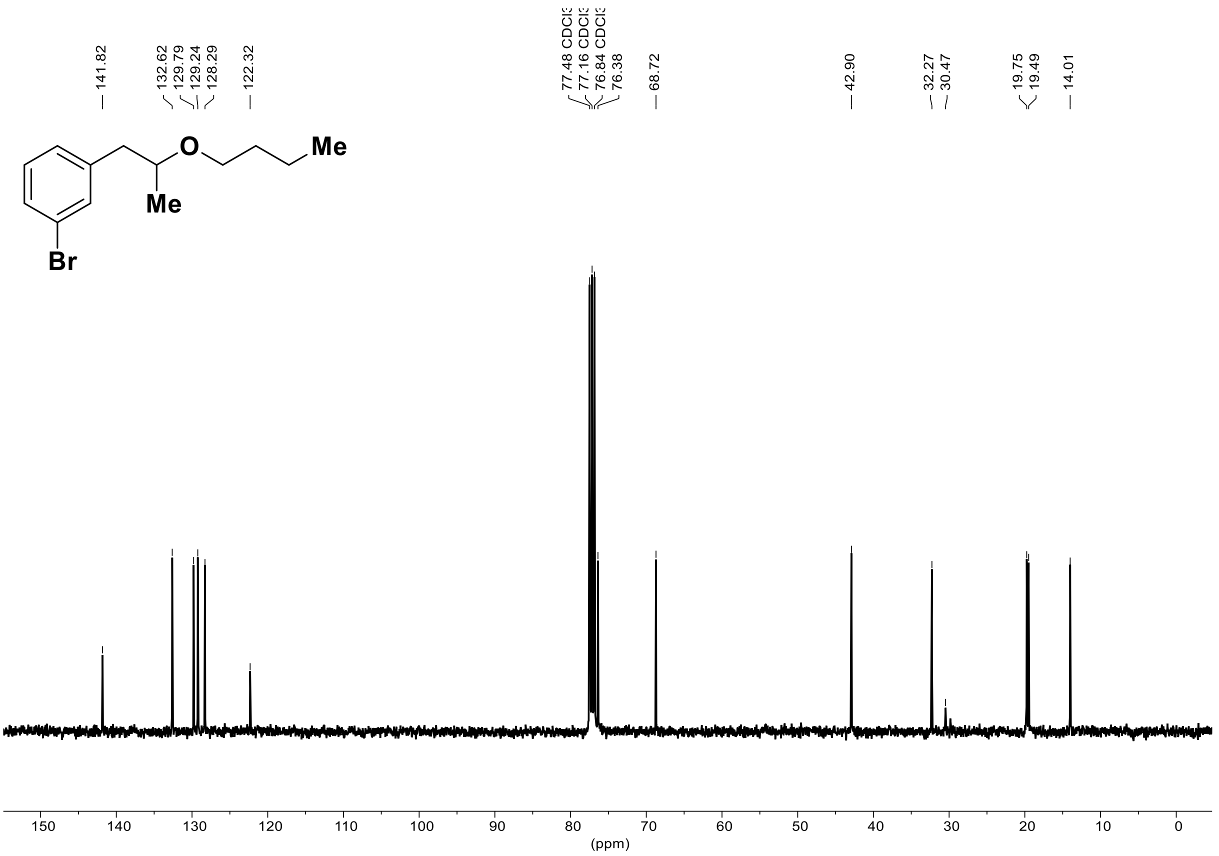

6t: ^{1}H, $CDCl_3$, 400 MHz

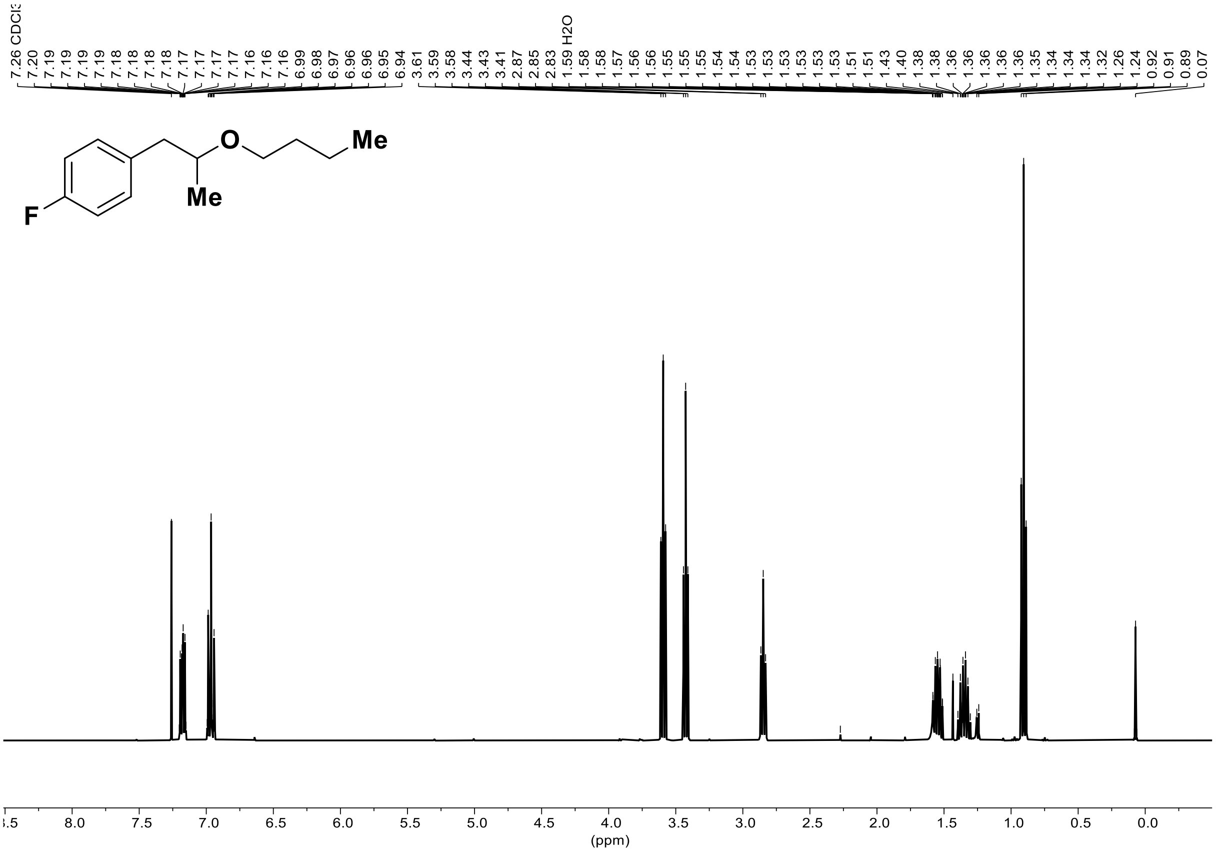

6t: ^{13}C, $CDCl_3$, 101 MHz

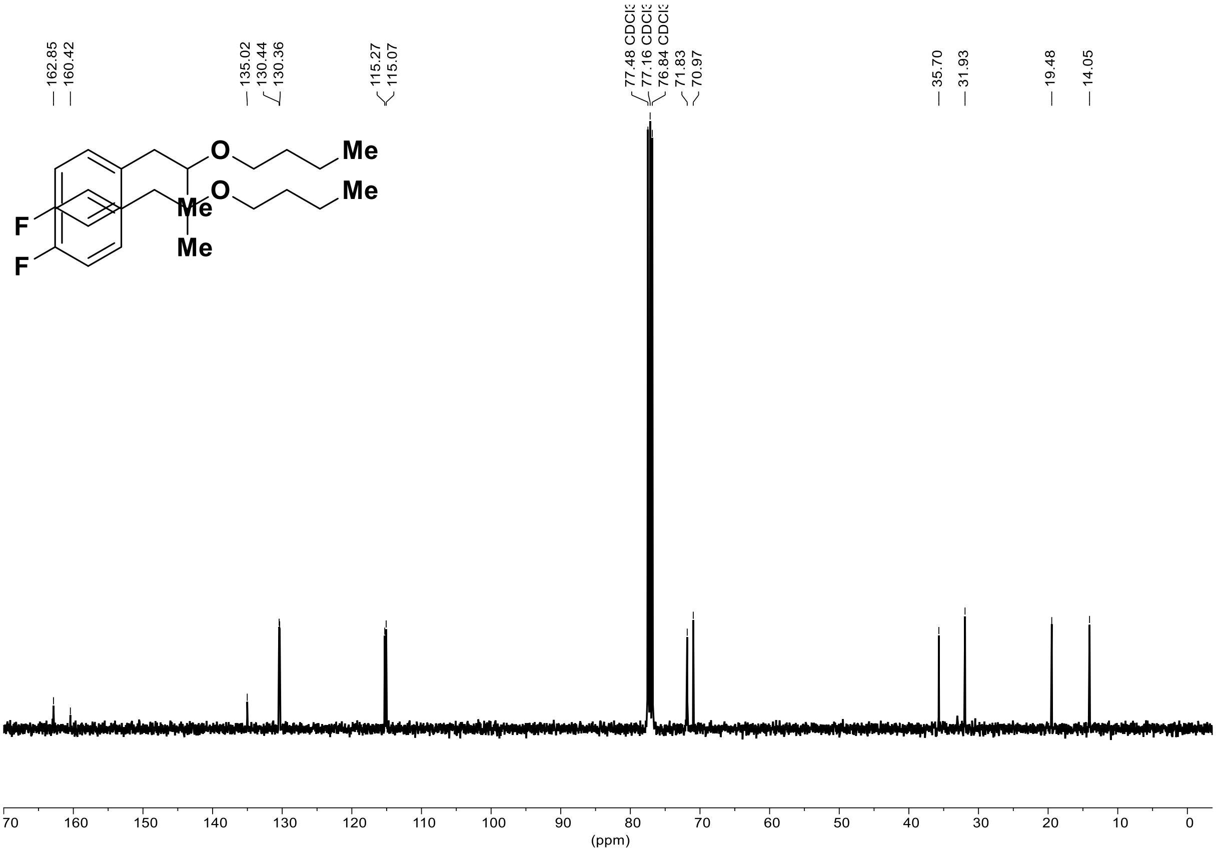

6t: ^{19}F, $CDCl_3$, 376 MHz

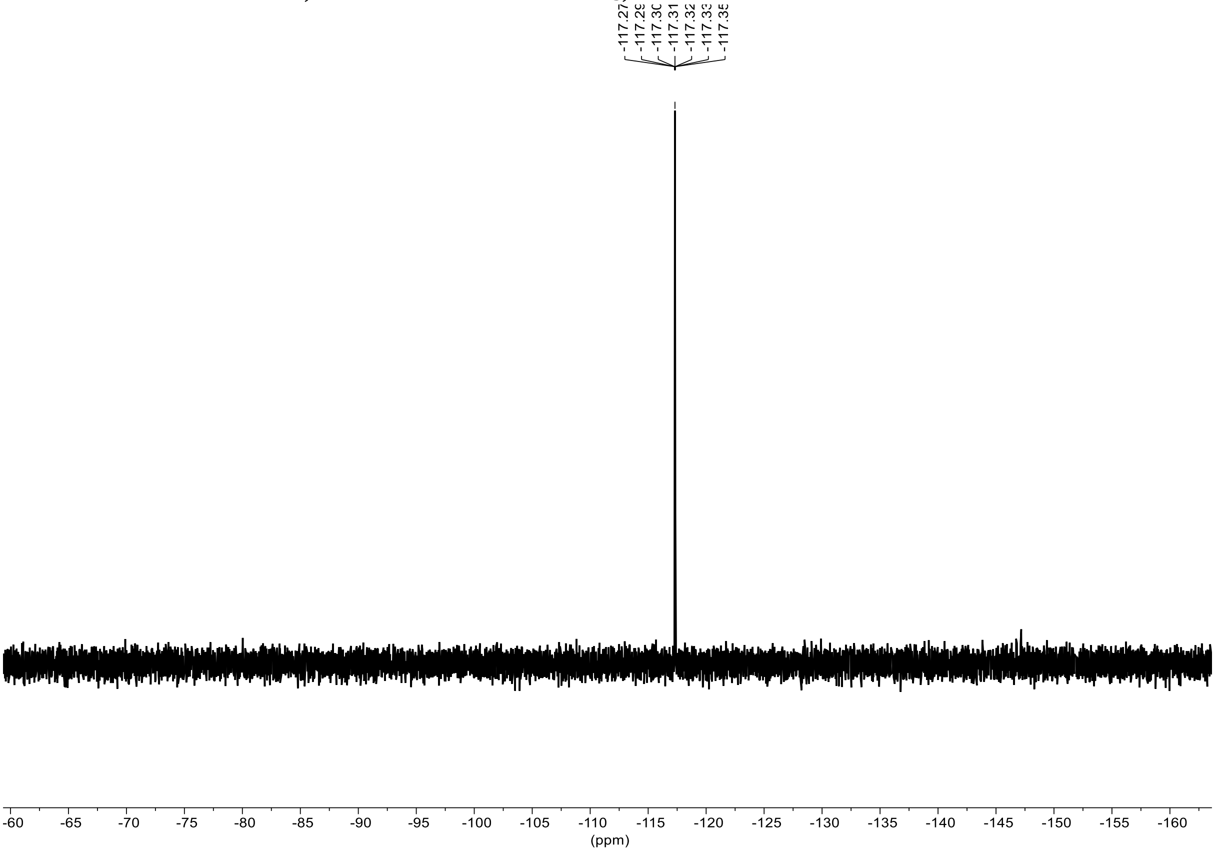

6u: ^{1}H, $CDCl_3$, 400 MHz

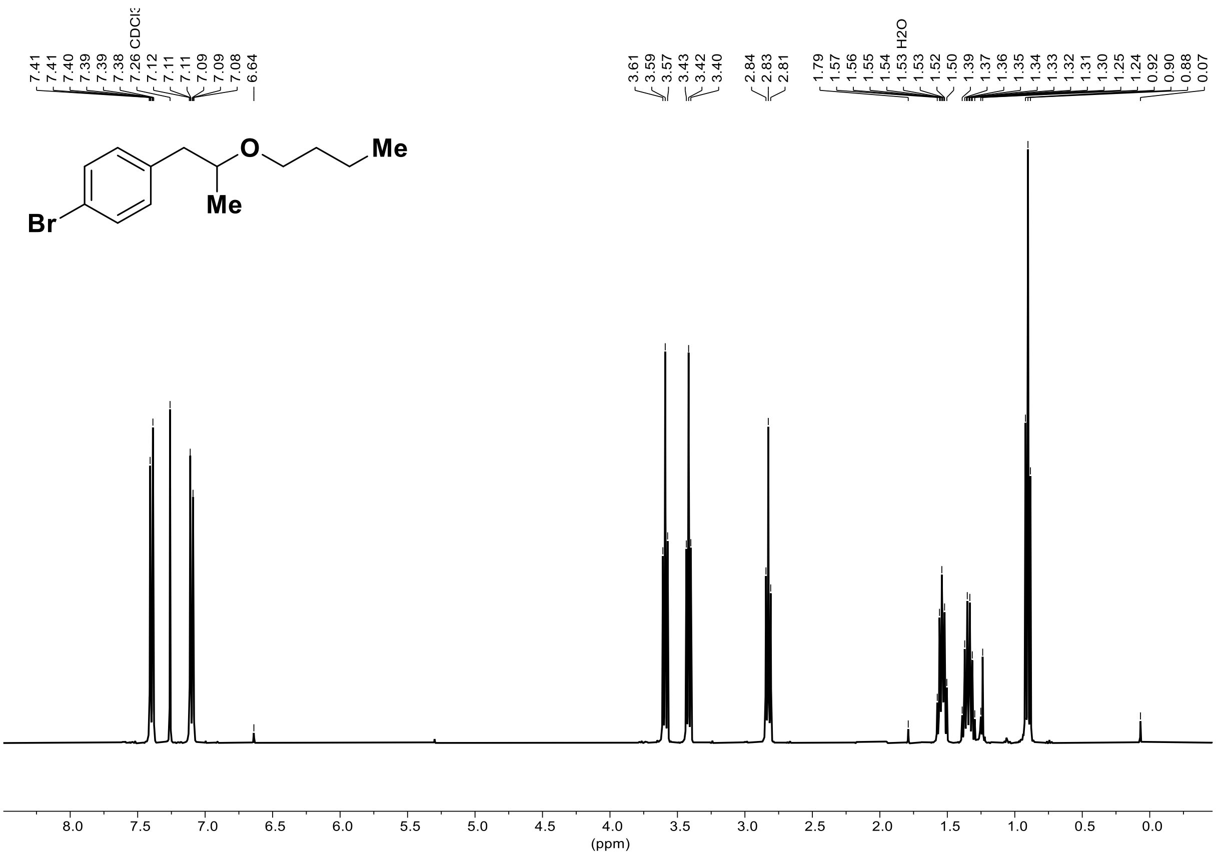

6u: ^{13}C, $CDCl_3$, 101 MHz

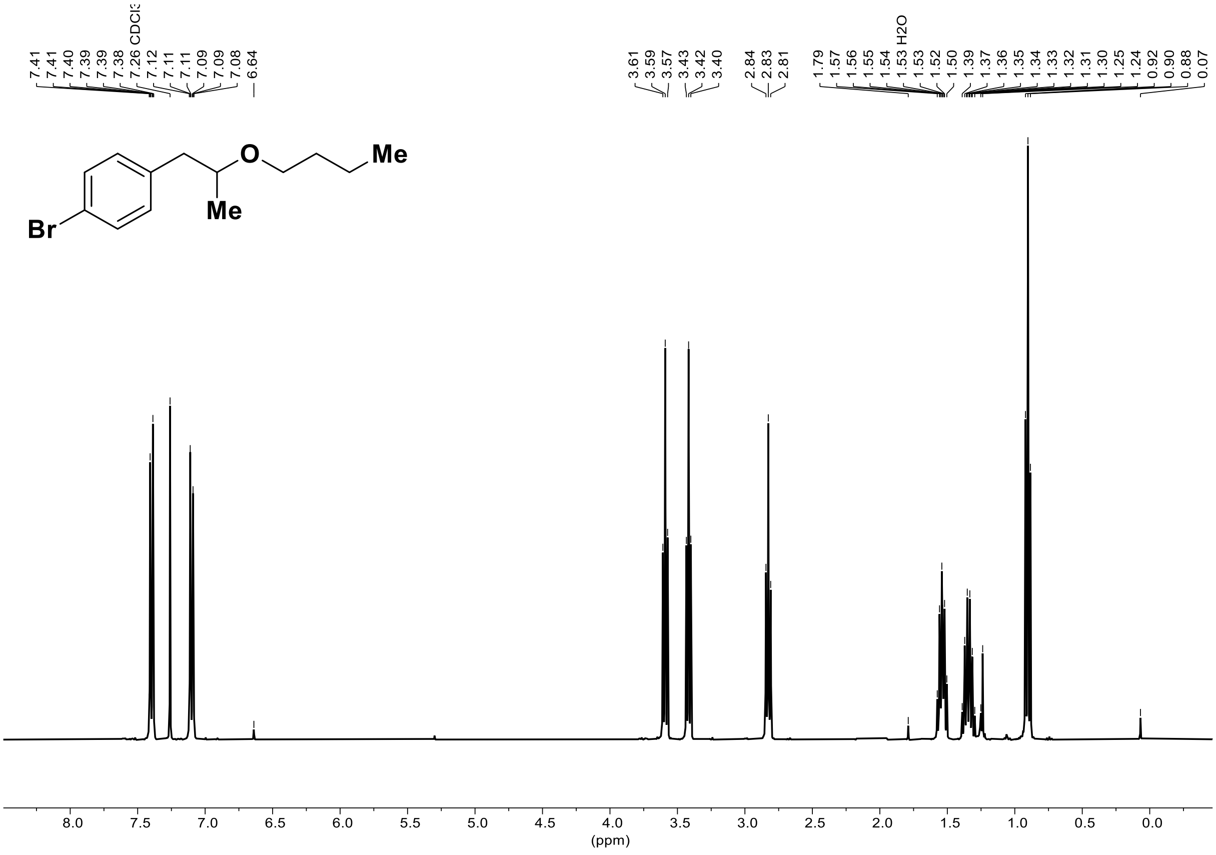

7: ^{1}H, $CDCl_3$, 400 MHz

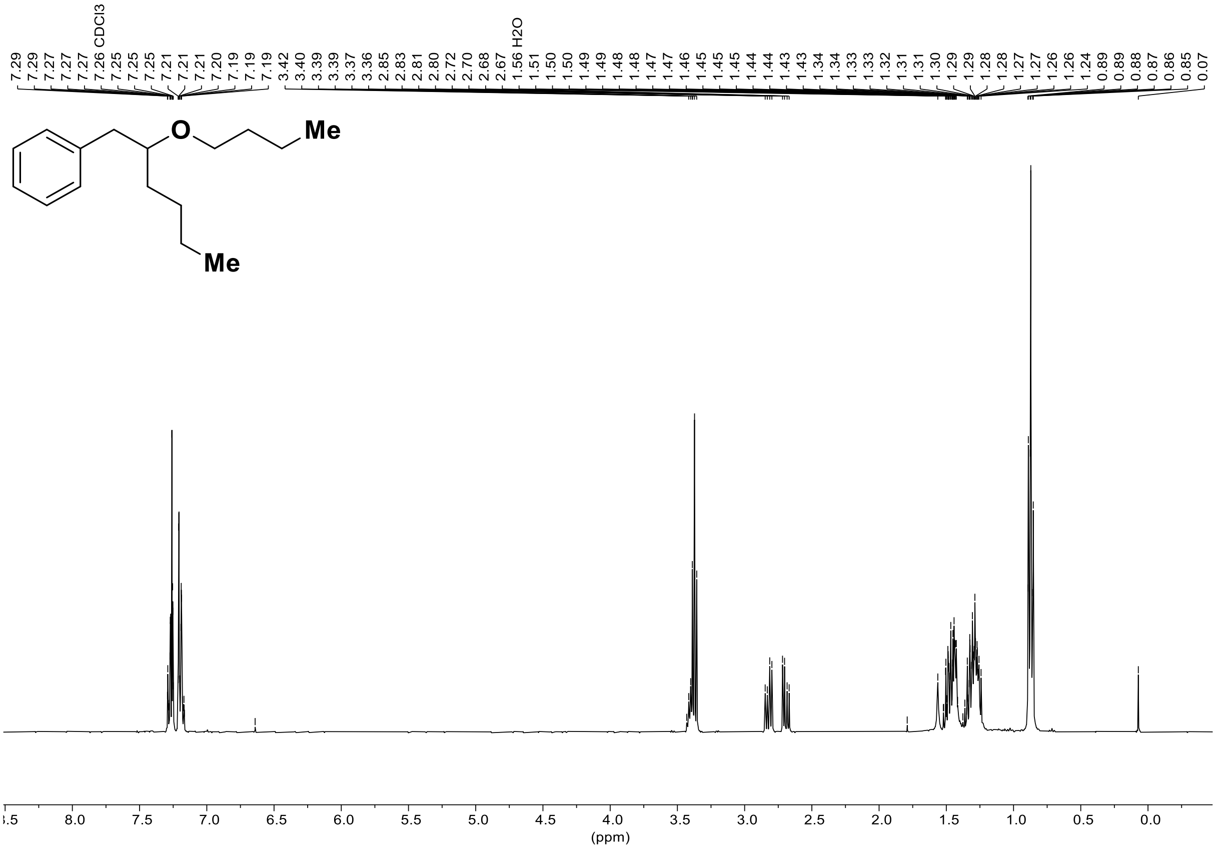

7: ^{13}C, $CDCl_3$, 101 MHz

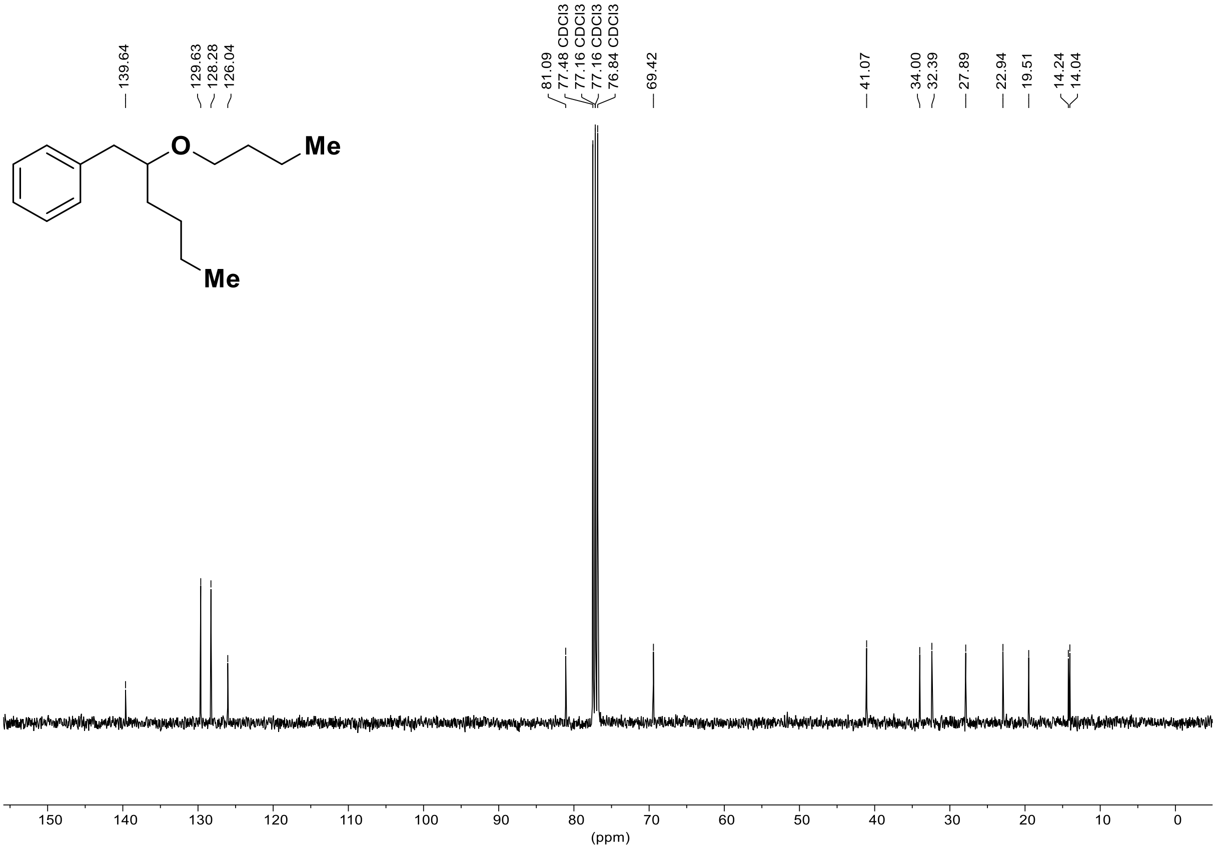

8. References

(1) Morozov, O. S.; Lunchev, A. V.; Bush, A. A.; Tukov, A. A.; Asachenko, A. F.; Khrustalev, V. N.; Zalesskiy, S. S.; Ananikov, V. P.; Nechaev, M. S., *Chem. Eur. J.* **2014**, *20*, 6162.

(2) (a) Catalano, V. J.; Malwitz, M. A.; Etogo, A. O., *Inorg. Chem.* **2004**, *43*, 5714; (b) Catalano, V. J.; Moore, A. L., *Inorg. Chem.* **2005**, *44*, 6558.

(3) Paloque, L.; Hemmert, C.; Valentin, A.; Gornitzka, H., *Eur. J. Med. Chem.* **2015**, *94*, 22.

(4) (a) Tsukamoto, I.; Koshio, H.; Kuramochi, T.; Saitoh, C.; Yanai-Inamura, H.; Kitada-Nozawa, C.; Yamamoto, E.; Yatsu, T.; Shimada, Y.; Sakamoto, S.; Tsukamoto, S., *Bioorg. Med. Chem.* **2009**, *17*, 3130; (b) Kriechbaum, M.; List, M.; R, J. F. B.; Patzschke, M.; Monkowius, U., *Chem. Eur. J.* **2012**, *18*, 5506.

(5) Raba, A.; Anneser, M. R.; Jantke, D.; Cokoja, M.; Herrmann, W. A.; Kühn, F. E., *Tetrahedron Lett.* **2013**, *54*, 3384.

(6) Riener, K.; Bitzer, M. J.; Pothig, A.; Raba, A.; Cokoja, M.; Herrmann, W. A.; Kuhn, F. E., *Inorg. Chem.* **2014**, *53*, 12767.

(7) Topf, C.; Hirtenlehner, C.; Fleck, M.; List, M.; Monkowius, U., *Z. Anorg. Allg. Chem.* **2011**, *637*, 2129.

(8) Liu, G.; Liu, C.; Han, F.; Wang, Z.; Wang, J., *Tetrahedron Lett.* **2017**, *58*, 726731.

(9) Sakai, N.; Moriya, T.; Konakahara, T., *J. Org. Chem.* **2007**, *72*, 5920.

(10) Corre, Y.; Rysak, V.; Capet, F.; Djukic, J. P.; Agbossou-Niedercorn, F.; Michon, C., *Chem. Eur. J.*, **2016**, *22*, 14036.

(11) Harper, M. J.; Emmett, E. J.; Bower, J. F.; Russell, C. A., *J. Am. Chem. Soc.* **2017**, *139*, 12386.

(12) Adamczyk-Wozniak, A.; Borys, K. M.; Czerwinska, K.; Gierczyk, B.; Jakubczyk, M.; Madura, I. D.; Sporzynski, A.; Tomecka, E., *Spectrochim. Acta. A. Mol. Biomol. Spectrosc.* **2013**, *116*, 616.

(13) Asao, N.; Aikawa, H.; Tago, S.; Umetsu, K., *Org. Lett.* **2007**, *9*, 4299-302.

(14) Colombel, V.; Rombouts, F.; Oehlrich, D.; Molander, G. A., *J. Org. Chem.* **2012**, *77*, 296670.

(15) Li, G.; Leow, D.; Wan, L.; Yu, J. Q., *Angew. Chem. Int. Ed.* **2013**, *52*, 1245.

(16) Frisch, M. J.; Trucks, G. W.; Schlegel, H. B.; Scuseria, G. E.; Robb, M. A.; Cheeseman, J. R.; Scalmani, G.; Barone, V.; Petersson, G. A.; Nakatsuji, H.; Li, X.; Caricato, M.; Marenich, A.; Bloino, J.; Janesko, B. G.; Gomperts, R.; Mennucci, B.; Hratchian, H. P.; Ortiz, J. V.; Izmaylov, A. F.; Sonnenberg, J. L.; Williams-Young, D.; Ding, F.; Lipparini, F.; Egidi, F.; Goings, J.; Peng, B.; Petrone, A.; Henderson, T.; Ranasinghe, D.; Zakrzewski, V. G.; Gao, J.; Rega, N.; Zheng, G.; Liang, W.; Hada, M.; Ehara, M.; Toyota, K.; Fukuda, R.; Hasegawa, J.; Ishida, M.; Nakajima, T.; Honda, Y.; Kitao, O.; Nakai, H.; Vreven, T.; Throssell, K.; J. A. Montgomery, J.; Peralta, J. E.; Ogliaro, F.; Bearpark, M.; Heyd, J. J.; Brothers, E.; Kudin, K. N.; Staroverov, V. N.; Keith, T.; Kobayashi, R.; Normand, J.; Raghavachari, K.; Rendell, A.; Burant, J. C.; Iyengar, S. S.; Tomasi, J.; Cossi, M.; Millam, J. M.; Klene, M.; Adamo, C.; Cammi, R.; Ochterski, J. W.; Martin, R. L.; Morokuma, K.; Farkas, O.; Foresman, J. B.; Fox, D. J. *Gaussian 09 rev. D.01*, Wallingford, CT, 2009.

(17) Chai, J.-D.; Head-Gordon, M., *Phys. Chem. Chem. Phys.* **2008**, *10*, 6615.

(18) Weigend, F.; Ahlrichs, R., *Phys. Chem. Chem. Phys.* **2005**, *7*, 3297-3305.

(19) (a) Andrae, D.; Häußermann, U.; Dolg, M.; Stoll, H.; Preuß, H., *Theor. Chim. Acta* **1990**, *77*, 123; (b) Peterson, K. A.; Figgen, D.; Goll, E.; Stoll, H.; Dolg, M., *J. Chem. Phys.* **2003**, *119*, 11113; (c) Pritchard, B. P.; Altarawy, D.; Didier, B.; Gibson, T. D.; Windus, T. L., *J. Chem. Inf. Model.* **2019**, *59*, 4814.

(20) Marenich, A. V.; Cramer, C. J.; Truhlar, D. G., *J. Phys. Chem. B* **2009**, *113*, 6378.

(21) (a) Foster, J. P.; Weinhold, F., *J. Am. Chem. Soc.* **1980**, *102*, 7211; (b) Reed, A. E.; Weinhold, F., *J. Chem. Phys.* **1983**, *78*, 4066; (c) Reed, A. E.; Weinstock, R. B.; Weinhold, F., *J. Chem. Phys.* **1985**, *83*, 735; (d) Reed, A. E.; Weinhold, F., *J. Chem. Phys.* **1985**, *83*, 1736; (e) Carpenter, J. E.; Weinhold, F., *J. Mol. Struct.: THEOCHEM* **1988**, *169*, 41; (f) Weinhold, F.; Carpenter, J. E., The natural bond orbital Lewis structure concept for molecules, radicals, and radical ions. In

The structure of small molecules and ions, Naaman, R.; Vager, Z., Eds. Springer: Boston, MA, 1988; pp 227-236.
(22) Glendening, E. D.; Reed, A. E.; Carpenter, J. E.; Weinhold, F., NBO Version 3.1. Gaussian Inc., Pittsburgh.
(23) Legault, C. Y., *CYLview20.* Université de Sherbrooke, 2020 (http://www.cylview.org).
(24) Zeineddine, A.; Estévez, L.; Mallet-Ladeira, S.; Miqueu, K.; Amgoune, A.; Bourissou, D., *Nat. Commun.* **2017**, *8*, 565.
(25) (a) Joost, M.; Zeineddine, A.; Estévez, L.; Mallet‒Ladeira, S.; Miqueu, K.; Amgoune, A.; Bourissou, D., *J. Am. Chem. Soc.* **2014**, *136*, 14654; (b) Harper, M. J.; Arthur, C. J.; Crosby, J.; Emmett, E. J.; Falconer, R. L.; Fensham-Smith, A. J.; Gates, P. J.; Leman, T.; McGrady, J. E.; Bower, J. F.; Russell, C. A., *J. Am. Chem. Soc.* **2018**, *140*, 4440; (c) Rodriguez, J.; Zeineddine, A.; Sosa Carrizo, E. D.; Miqueu, K.; Saffon-Merceron, N.; Amgoune, A.; Bourissou, D., *Chem. Sci.* **2019**, *10*, 7183; (d) Cadge, J. A.; Bower, J. F.; Russell, C. A., *Angew. Chem. Int. Ed.* **2021**, *60*, 24976.
(26) Fernández, I.; Wolters, L. P.; Bickelhaupt, F. M., *J. Comput. Chem.* **2014,** *35*, 2140.
(27) The equivalent donation from I to the Au–P σ* orbital was indicated as the largest stabilizing interaction in the oxidative addition transition state with a MeDalPhos-ligated Au complex, see ref 24.
(28) Bruker *SAINT+ v8.38A Integration Engine, Data Reduction Software*, Bruker Analytical X-ray Instruments Inc.: Madison, WI, U.S.A., 2015.
(29) Bruker *SADABS 2014/15, Bruker AXS area detector scaling and absorbtion correction*, Bruker Analytical X-ray Instruments Inc.: Madison, WI, U.S.A., 2014/15.
(30) (a) Palatinus, L.; Chapuis, G., *J. Appl. Crystallogr.* **2007**, *40*, 786; (b) Palatinus, L.; van der Lee, A., *J. Appl. Crystallogr.* **2008**, *41*, 975; (c) Palatinus, L.; Prathapa, S. J.; van Smaalen, S., *J. Appl. Crystallogr.* **2012**, *45*, 575.
(31) Sheldrick, G., *Acta Crystallogr., Sect. C* **2015**, *71*, 38.
(32) Dolomanov, O. V.; Bourhis, L. J.; Gildea, R. J.; Howard, J. A. K.; Puschmann, H., *J. Appl. Crystallogr.* **2009**, *42*, 339.
(33) Cadge, J. A.; Sparkes, H. A.; Bower, J. F.; Russell, C. A., *Angew. Chem. Int. Ed.* **2020**, *59,* 6617.
(34) Winston, M. S.; Wolf, W. J.; Toste, F. D., *J. Am. Chem. Soc.* **2015**, *137*, 7921.
